# Unveiling the Enigmatic nature of six neglected Amazonian *Leishmania* (*Viannia*) species using the hamster model: Virulence, Histopathology and prospection of LRV1

**DOI:** 10.1371/journal.pntd.0012333

**Published:** 2024-08-09

**Authors:** Rodrigo Pedro Soares, Igor Campos Fontes, Felipe Dutra-Rêgo, Jeronimo Nunes Rugani, Paulo Otávio L. Moreira, Vânia Lúcia Ribeiro da Matta, Gabriela Venícia Araujo Flores, Carmen Maria Sandoval Pacheco, Andrey José de Andrade, Magda Clara Vieira da Costa-Ribeiro, Jeffrey Jon Shaw, Márcia Dalastra Laurenti

**Affiliations:** 1 Grupo Biotecnologia Aplicada ao Estudo de Patógenos (BAP), Instituto René Rachou, Fundação Oswaldo Cruz (FIOCRUZ), Belo Horizonte, Minas Gerais, Brazil; 2 Laboratório de Patologia das Moléstias Infecciosas, Departamento de Patologia, Faculdade de Medicina, Universidade de São Paulo (USP), São Paulo, São Paulo, Brazil; 3 Laboratório de Parasitologia Molecular, Departamento de Patologia Básica, Setor de Ciências Biológicas, Universidade Federal do Paraná (UFPR), Curitiba, Paraná, Brazil; 4 Departamento de Parasitologia, Instituto de Ciências Biomédicas, Universidade de São Paulo (USP), São Paulo, São Paulo, Brazil; Federal University of Rio de Janeiro, BRAZIL

## Abstract

American tegumentary leishmaniasis (ATL) is highly endemic in the Amazon basin and occurs in all South American countries, except Chile and Uruguay. Most Brazilian ATL cases are due to *Leishmania (Viannia) braziliensis*, however other neglected Amazonian species are being increasingly reported. They belong to the subgenus *L*. *(Viannia)* and information on suitable models to understand immunopathology are scarce. Here, we explored the use of the golden hamster *Mesocricetus auratus* and its macrophages as a model for *L*. *(Viannia)* species. We also studied the interaction of parasite glycoconjugates (LPGs and GIPLs) in murine macrophages. The following strains were used: *L*. *(V*.*) braziliensis* (MHOM/BR/2001/BA788), *L*. *(V*.*) guyanensis* (MHOM/BR/85/M9945), *L*. *(V*.*) shawi* (MHOM/BR/96/M15789), *L*. *(V*.*) lindenbergi* (MHOM/BR/98/M15733) and *L*. *(V*.*) naiffi* (MDAS/BR/79/M5533). *In vivo* infections were initiated by injecting parasites into the footpad and were followed up at 20- and 40-days PI. Parasites were mixed with salivary gland extract (SGE) from wild-captured *Nyssomyia neivai* prior to *in vivo* infections. Animals were euthanized for histopathological evaluation of the footpads, spleen, and liver. The parasite burden was evaluated in the skin and draining lymph nodes. *In vitro* infections used resident peritoneal macrophages and THP-1 monocytes infected with all species using a MOI (1:10). For biochemical studies, glycoconjugates (LPGs and GIPLs) were extracted, purified, and biochemically characterized using fluorophore-assisted carbohydrate electrophoresis (FACE). They were functionally evaluated after incubation with macrophages from C57BL/6 mice and knockouts (TLR2-/- and TLR4-/-) for nitric oxide (NO) and cytokine/chemokine production. All species, except *L*. *(V*.*) guyanensis*, failed to generate evident macroscopic lesions 40 days PI. The *L*. *(V*.*) guyanensis* lesions were swollen but did not ulcerate and microscopically were characterized by an intense inflammatory exudate. Despite the fact the other species did not produce visible skin lesions there was no or mild pro-inflammatory infiltration at the inoculation site and parasites survived in the hamster skin/lymph nodes and even visceralized. Although none of the species caused severe disease in the hamster, they differentially infected peritoneal macrophages *in vitro*. LPGs and GIPLs were able to differentially trigger NO and cytokine production via TLR2/TLR4 and TLR4, respectively. The presence of a sidechain in *L*. *(V*.*) lainsoni* LPG (type II) may be responsible for its higher proinflammatory activity. After Principal Component analyses using all phenotypic features, the clustering of *L*. *(V*.*) lainsoni* was separated from all the other *L*. *(Viannia)* species. We conclude that *M*. *auratus* was a suitable *in vivo* model for at least four dermotropic *L*. *(Viannia)* species. However, *in vitro* studies using peritoneal cells are a suitable alternative for understanding interactions of the six *L*. *(Viannia)* species used here. LRV1 presence was found in *L*. *(V*.*) guyanensis* and *L*. (*V*.) *shawi* with no apparent correlation with virulence *in vitro* and *in vivo*. Finally, parasite glycoconjugates were able to functionally trigger various innate immune responses in murine macrophages *via* TLRs consistent with their inflammatory profile *in vivo*.

## Author summary

American Tegumentary Leishmaniasis (ATL) is one of the major neglected diseases occurring in Brazil and the Amazon contributes with a wide range of species that are not completely known. These little-known species are transmitted to rural workers entering the forest during lumbering, mining, and latex extraction activities and rarely in peri domestic situation. Infection occurs by the bite of infected sand flies in different environments and biomes. One of the major constraints in studying *L*. (*Viannia*) species is the difficulty of finding suitable animal and cellular models to investigate their biology. The mouse model is not a suitable model for most *L*. (*Viannia*) species. However, hamsters were considered a good model for *L*. (*V*.) *braziliensis*. In the search for a better model for some neglected dermotropic Amazonian *Leishmania*, we evaluated interactions *in vivo* in the hamster and *in vitro* in its macrophages. All species infected cells *in vitro*, but hamsters could only be considered as a suitable *in vivo* model for *L*. (*V*.) *guyanensis*. Importantly the glycoconjugates of the six *L*. (*Viannia*) species were very proinflammatory, showing various levels of macrophage activation. This paper opens possibilities of determining more accurately the taxonomic position of *L*. (*V*.) *lainsoni* within the subgenus *L*. (*Viannia*).

## Introduction

*Leishmania* species from *L*. (*Viannia*) subgenus have been identified from patients exhibiting American Tegumentary leishmaniasis (ATL) [1]. Most Brazilian ATL cases are caused by *Leishmania* (*Viannia*) *braziliensis*, and in past years [2] there has been an increase in the number of cases. Eight species are considered proven vectors including *Nyssomyia intermedia* and *Ny*. *whitmani* [3]. Several wild and synanthropic reservoirs have been identified including rodents [4–6] and marsupials [7,8]. Recently, urbanization of ATL was reported in the Southeast Brazil, where *L*. (*V*.) *braziliensis* proven vectors *Migonemyia migonei*, *Ny*. *intermedia* and *Ny*. *whitmani* were captured [9,10]. This novel finding sparked concern in the disease epidemiology since urbanization is associated to visceral leishmaniasis (VL) due to *L*. (*Leishmania*) *infantum* [11].

*L*. *(V*.) *braziliensis* and *L*. (*V*.) *guyanensis* are epidemiologically the most important ATL species. Most cases are in the north and northeast of Brazil, where *L*. *(Viannia)* parasites are endemic, the disease being a zoonosis and occupational hazard [12]. *Nyssomyia umbratilis*, found in the Amazon region and other Brazilian states, transmits *L*. (*V*.) *guyanensis* above the Negro River [13,14]. The frequent infection of this species by *Leishmania* RNA virus 1 (LRV1), a *Totiviridae* virus known to increase disease severity, is a distinguished characteristic of *L*. *guyanensis* [15,16]. Other dermotropic species also contribute to ATL increase in Latin America. These include *L*. (*V*.) *naiffi*, *L*. (*V*.) *shawi*, *L*. (*V*.) *lindenbergi* and *L*. (*V*.) *lainsoni*. *L*. (*V*.) *naiffi* was first isolated from an armadillo (*Dasypus novemcinctus*) in the Brazilian state of Pará, Colombia and French Guyana [17,18]. The suspected vectors were *Psychodopygus paraensis* and *Ps*. *ayrozai* [19]. Recently, *L*. (*V*.) *naiffi* DNA was detected in several sandfly species including *Ps*. *hirsutus*, *Ny*. *antunesi* and *Ny*. *shawi* in the Brazilian state of Rondônia [20]. Recently, *L*. (*V*.) *lainsoni* has also been detected in French Guyana, Peru and Bolivia [18], but its first description was from a human case in Pará state, Brazil [21], where *Cuniculus paca* (Rodentia: Cuniculidae) was incriminated as the wild reservoir [22]. Later, field-captured *Trichophoromyia ubiquitalis* and *Trichophoromyia brachipyga* were incriminated as suspected and proven vectors of *L*. (*V*.) *lainsoni*, respectively [23]. A distinguished feature of *L*. (*V*.) *lainsoni* compared to the other *L*. (*Viannia*) species is that it possesses several biological, biochemical, and molecular characteristics that differ from the others of the same subgenus [24]. *L*. (*V*.) *shawi* was also reported in Pará state, Brazil, whose wild reservoirs include *Cebus apella*, *Chiropotes satanas*, *Choloepus didactylus*, *Bradypus tridactylus*, and *Nasua nasua* [25]. Later, this species was isolated from human cases and the suspected vector was *Ny*. *whitmani* [26]. Finally, *L*. (*V*.) *lindenbergi* was first reported in an ATL outbreak among eight soldiers in Pará state, Brazil. Clinically it is very similar to *L*. (*V*.) *naiffi*, whose suspected vector is *Ny*. *antunesi* and sylvatic reservoirs are unknown [27]. Recently, *L*. (*V*.) *lindenbergi* was also reported in another Brazilian state of Rondonia where *Ny*. *antunesi* occurs [20,28]. Several factors are responsible for ATL increase in the Americas including climate, economic, environmental and others [29]. Those are neglected species and even though some aspects of their biology are known, information on suitable animal models is still scarce.

For over 60 years [30] the hamster has been a model for several pathogens. This model is suitable for both viscerotropic and dermotropic *Leishmania* and its clinicopathologic features and immunopathologic mechanisms are like those found in humans, but quite different from mice [31–33]. Hamsters have been considered an acceptable model for *L*. (*V*.) *braziliensis* infection [34] but we do not know their susceptibility to infection with other less common dermotropic *L*. (*Viannia*) species. In this work, in the search for a better model and to understand immunopathology, we evaluated the interaction of *L*. (*Viannia*) species and their glycoconjugates (LPGs and GIPLs) with *M*. *auratus* and human/rodent macrophages.

## 2. Material and methods

### Ethics statement

Animals (*Mesocricetus auratus*) were kept in the Animal Facility of the Faculdade de Medicina da Universidade de São Paulo (FMUSP). *Mus musculus* (C57BL/6, TLR2 -/- and TLR4 -/-) were kept in the Animal Facility of Instituto René Rachou, Oswaldo Cruz Foundation. Animal handling was performed according to the guidelines of the Brazilian College for Experiments with Animals (COBEA-Law 11.794/2008). Our protocols were approved by our Ethics Committee in Animal Experimentation/Comitê de Ética em Experimentação Animal (ECAE/CEUA) (Protocol 1659/2021 and LW-32/16).

### Parasite culture and molecular typing

Three isolates and three World Health Organization Reference *L*. (*Viannia*) species were evaluated (Table 1). Promastigotes were cultured in M199 medium and/or Schneider supplemented with 10% fetal bovine serum (FBS) (Invitrogen/ Thermo Fisher Scientific (Carlsbad, USA), penicillin (200 u/mL), and streptomycin (200 μg/mL) (all Merck KGaA, Darmstadt, Germany), at 25°C [35]. To confirm parasite identity, molecular typing (*hsp*70 gene) was performed [36]. Confirmed sequences were deposited in the GenBank database (accession numbers PP331240, PP331241, PP331242, PP331243, PP331244 and PP331245).

**Table 1:** *Leishmania* strains used in this study.

Strain	WHO reference code	Nomenclature in the text	Host	Origin
*Leishmania (V*.*) guyanensis*	MHOM/BR/75/M4147	M4147	*Homo sapiens*	Pará/BR
*Leishmania (V*.*) guyanensis*	IUMB/BR/85/M9945	M9945	*Ny*. *umbratilis*	Pará/BR
*Leishmania (V*.*) braziliensis*	MHOM/BR/01/BA788	BA788	*Homo sapiens*	Bahia/BR
*Leishmania (V*.*) shawi*	MHOM/BR/96/M15789	M15789	*Homo sapiens*	Bahia/BR
*Leishmania (V*.*) lainsoni*	MHOM/BR/81/M6426	M6426	*Homo sapiens*	Bahia/BR
*Leishmania (V*.*) lindenbergi*	MHOM/BR/1996/M15733	M15733	*Homo sapiens*	Bahia/BR
*Leishmania (V*.*) naiffi*	MDAS/BR/1979/M5533	M5533	*Dasypus* sp	Minas Gerais/BR
*Leishmania (L*.*) infantum*	MCAN/BR/89/Ba-262	BA262	*Canis familiaria*	Bahia/BR

### Extraction and purification of glycoconjugates

Lipophosphoglycans (LPGs) and glycoinositolphospholipids (GIPLs) were extracted and purified using Solvent E in a hydrophobic interaction column as previously reported [37]. Purified LPGs and GIPLs were depolymerized using mild and strong acid hydrolysis, respectively. Poly- (repeat units) and monosaccharides were resolved by fluorophore-assisted carbohydrate electrophoresis (FACE) and the profiles were visualized under UV [38].

### *In vitro* assays

Hamster resident intraperitoneal macrophages were recovered by sequential washing with 10 mL of cold PBS. Cell viability was checked by trypan blue. THP-1 cells were differentiated by addition of 20 nM phorbol 12-myristate 13-acetate as previously reported [39]. Cells (10^5^/well) were seeded into 24-well culture plates for adhesion (1 h, 35°C, 5% CO_2_) with RPMI supplemented with 2 mM glutamine, 50 U/mL of penicillin, 50 μg/mL streptomycin and 10% FBS. Cells were exposed to *L*. *Viannia* species (10^6^/well, 4 h, MOI 1:10). After 48 hours, 13-mm round coverslips were recovered and stained with Panoptic. For functional studies with glycoconjugates, thioglycolate-elicited macrophages were removed from mice peritoneal cavity (C57BL/6) and respective knockouts (TLR2 -/- and TLR4 -/-). Macrophages were attached to 96-well plates and primed with IFN-γ (3U/mL) for 16 h prior to incubation with LPG and GIPLs (10 μg/mL) for 48 h (37°C, 5% CO_2_). Negative control was RPMI medium only. Positive controls were primed with IFN-γ (3U/mL) and included LPS (100 ng/mL, TLR4 agonist) and *Staphylococcus aureus* extract (100 ng/mL, TLR2 agonist) [40]. Supernatants were assayed for nitric oxide and cytokines using Griess method and CBA flex Inflammation kit (Beckton and Dickinson) according to manufacturer’s instructions. The following cytokines/chemokine were assayed TNF-α, IL-6, IL-12, and MCP-1. Two parallel experiments were performed in triplicate totalizing 6 samples for each glycoconjugate exposure. These experiments were performed in the Flow Cytometry platform in the Rene Rachou Institute, Oswaldo Cruz Foundation.

### Sand flies and salivary gland dissection

*Nyssomyia neivai* is a suspected vector of *L*. (*V*.) *braziliensis* in the South of Brazil [41]. The ability of this sand fly to acquire infection from *L*. (*V*.) *braziliensis*-infected hamsters has already been reported [42]. Salivary glands extracts (SGEs) are important modulators of early and late infection [43]. For this reason, we chose this species´ salivary glands for our studies with *L*. (*Viannia*) isolates. Sand flies found resting on the wall of a residence located in Castro municipality (24°59’31.2” S, 49°37’40.8” W), Paraná, Brazil were captured with an aspirator. They were placed in a bottle with pieces of cotton soaked in a 10% sucrose solution prior to dissection in the laboratory. *Ny*. *neivai* females’ SGEs were dissected in PBS under a stereomicroscope and stored. *Vouchers* were deposited in the “Coleção de Parasitologia do Departamento de Patologia Básica” (ColPar/DPAT/UFPR).

### *In vivo* assays

For each species, 8 hamsters were inoculated subcutaneously (SC) in the right rear footpad with 1 X 10^5^ of stationary parasites in a volume of 100 μL of PBS and salivary gland extract (0.5 gland) of *Ny*. *neivai*. Negative controls were inoculated with PBS only. Animals were followed by 20- and 40-days PI. At the two time-points, hamsters were anaesthetized and euthanized. Skin, liver, and spleen fragments were collected for histopathology (hemotoxylin-eosin-HE) and immunohistochemistry (IHC) as previously reported [43]. To evaluate the inflammation in the skin, a semi-quantitative comparative analysis was performed [44]. The results were scored based on the intensity of cellularity as follows: (−) negative, 1–50 cells (+) discrete, 50–100 cells (++) moderate and higher than 100 cells (+++) intense. To determine parasite load, limiting dilution was performed in the skin and draining lymph nodes as reported elsewhere [45].

### LRV1 detection

Attempts to detect LRV1 were performed using RNA extracted from cultured parasites (1 x 10^8^ cells) using the Trizol method (Life Technologies). Samples were treated with DNase I before cDNA synthesis as previously reported, using the Super Script III- First Strand Synthesis Kit (Invitrogen). Specific primers (SMB2109 and SMB2110) were used to amplify a 496 bp LRV1 fragment and β-tubulin (396 bp) was used as quality control. Amplified fragments were resolved in 1% agarose gels [46,47].

### Statistical and Principal Component analyses (PCA)

For morphometric semi-quantitative analysis, 25 fields of each slide were randomly photographed. Quantification of parasitism and mononuclear cells for each species were performed in KS300 software. Data were analyzed using GraphPad Prism 5.0. Statistical analyses used One-way ANOVA followed by Bonferroni´s and Dunn´s Multiple Comparison tests. For NO and cytokines/chemokine, the Shapiro Wilk test was conducted to test the null hypothesis that data were sampled from a Gaussian distribution [48]. When data did not deviate from a Normal distribution, Student´s “t” test were performed. *P*-values <0.05 were considered statistically significant. Principal Component Analysis (PCA) and hierarchical clustering by squared-Euclidean distance [33] were performed using the software IBM SPSS Statistics 22. This approach used all biological, biochemical, and immunological parameters summarized in Tables 2 and 3 to generate an integrative description of the different characteristics of the *L*. (*Viannia*) species.

**Table 2:** Biological parameters assessed in *Leishmania* strains used in this study.

Strain	*In vitro* infection for macrophages		*In vivo* infection in *M*. *auratus*				LRV1
	**Hamster**	**THP-1**	**HE**	**IHC**	**LD 20/40 days PI**		
	**infection/amastigotes per cell**	**infection/amastigotes per cell**	**20/40 days PI**	**20/40 days PI**	**Skin**	**Lymph node**	
*L*. *(V*.*) guyanensis* (M9945)	**+++/+++**	**++/++**	**+++/-**	**+/+++**	**+++/+++**	**++/+++**	**+++**
*L*. *(V*.*) shawi* (M15789)	**+++/++**	**+/+**	**-/-**	**+/++**	**-/+++**	**+/+++**	**++**
*L*. *(V*.*) lainsoni* (M6426)	**++/++**	**++/++**	**+/-**	**++/++**	**+++/++**	**+++/+++**	**-**
*L*. *(V*.*) braziliensis* (BA788)	**++/++**	**+/+**	**+++/++**	**+/+++**	**++/+++**	**+++/+++**	**-**
*L*. *(V*.*) naiffi* (M5533)	**+/+**	**+/+**	**-/-**	**+/-**	**-/-**	**-/-**	**-**
*L*. *(V*.*) lindenbergi* (M15733)	**+/+**	**++/+**	**-/-**	**+/+**	**-/-**	**-/-**	**-**

+++, higher infection; ++, moderate infection; +, lower infection; -, not detectable; HE, hematoxylin-eosin staining; IHC, immunohistochemistry; LD, limiting dilution; PI, post-infections and, LRV1, *LeishmaniaRNAvirus* 1.

**Table 3:** Biochemical and Functional properties of *Leishmania* glycoconjugates used in this study.

Parameter	Strains/Glycoconjugates											
	L.guy (M9945)		L.sha (M15789)		L.lai (M6426)		L.braz (BA788)		L.nai (M5533)		L.lin (M15733)	
	LPG	GIPL	LPG	GIPL	LPG	GIPL	LPG	GIPL	LPG	GIPL	LPG	GIPL
**Type**	**I**	**II/Hybrid**	**I**	**I**	**I**	**I**	**II**	**I**	**I**	**I**	**I**	**I**
**NO/TLR2**	**+**	**+**	**++**	**++**	**++**	**+**	**+**	**+**	**+**	**+**	**++**	**+**
**NO/TLR4**	**++**	**++**	**++**	**++**	**+++**	**+++**	**++**	**++**	**++**	**++**	**++**	**++**
**IL-6/TLR2**	**-**	**+**	***+***	**++**	**-**	**+++**	**-**	**+**	**-**	**+**	**+++**	**+**
**IL-6/TLR4**	**+**	**-**	***+***	**+**	**+++**	**+++**	**+**	**+**	**+**	**+**	**+**	**+**
**IL-12/TLR2**	**-**	**+**	***-***	**-**	**-**	**++**	**-**		**-**	**-**	**-**	**-**
**IL-12/TLR4**	**++**	**+**	***+***	**++**	**+**	**-**	**+**	**++**	**+**	**-**	**+**	**-**
**TNF-**α**/TLR2**	**-**	**-**	**++**	**++**	**+++**	**+++**	**-**	**-**	**-**	**-**	**-**	**-**
**TNF-**α**/TLR4**	**+**	**-**	**++**	**++**	**+**	**+++**	**+**	**+**	**+**	**+**	**+**	**+**
**MCP-1/TLR2**	**+**	**-**	**+**	**-**	**+**	**-**	**+**	**-**	**+**	**-**	**+**	**-**
**MCP-1/TLR4**	**+**	**+**	**+**	**+**	**+++**	**+**	**++**	**+**	**++**	**+**	**++**	**+**

**Legend:** LPG, Lipophosphoglycan; GIPLs, glycoinositolphospholipids; TLR2, toll-like receptor 2; TLR4, toll-like receptor 4; NO, nitric oxide; IL-6, interleukin 6; IL-12, interleukin 12; TNF-α, tumor necroses factor alpha and, MCP-1, monocyte chemoattractant protein-1.

## Results

### LRV1 presence did not seem to affect infection in hamster resident peritoneal macrophages and human THP-1 cells

**Fig 1:**
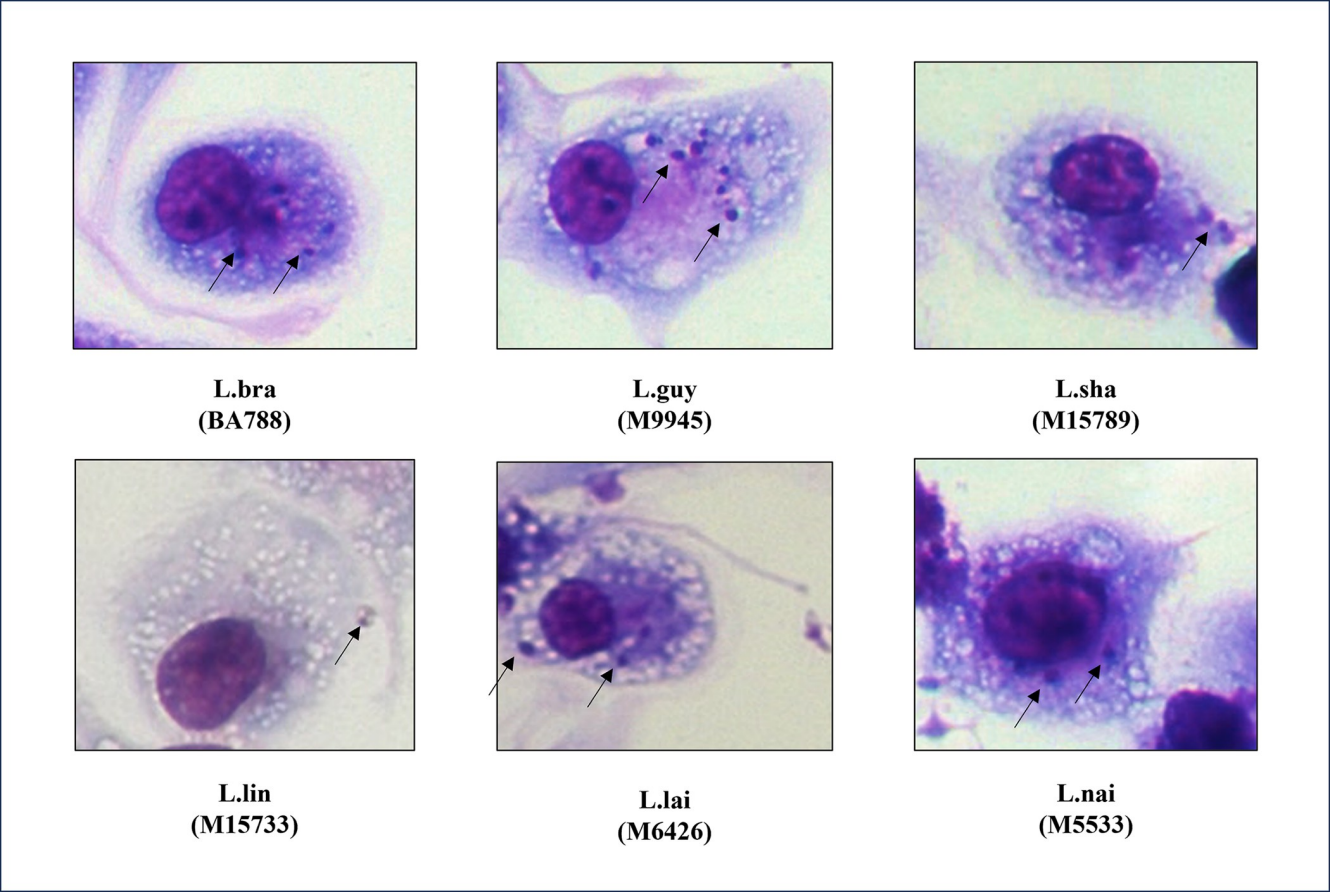
Resident peritoneal macrophages from *Mesocricetus auratus* are susceptible to *in vitro Leishmania* (*Viannia*) infection. Arrows indicate intracellular amastigote forms. Legend: L.braz, *L*. *braziliensis*; L.guy, *L*. *guyanensis*; L.sha, *L*. *shawi*, L.lind, *L*. *linderbergi*, L.lai, *L*. *lainsoni* and L.nai, *L*. *naiffi*. Magnification of 1000x.

**Fig 2:**
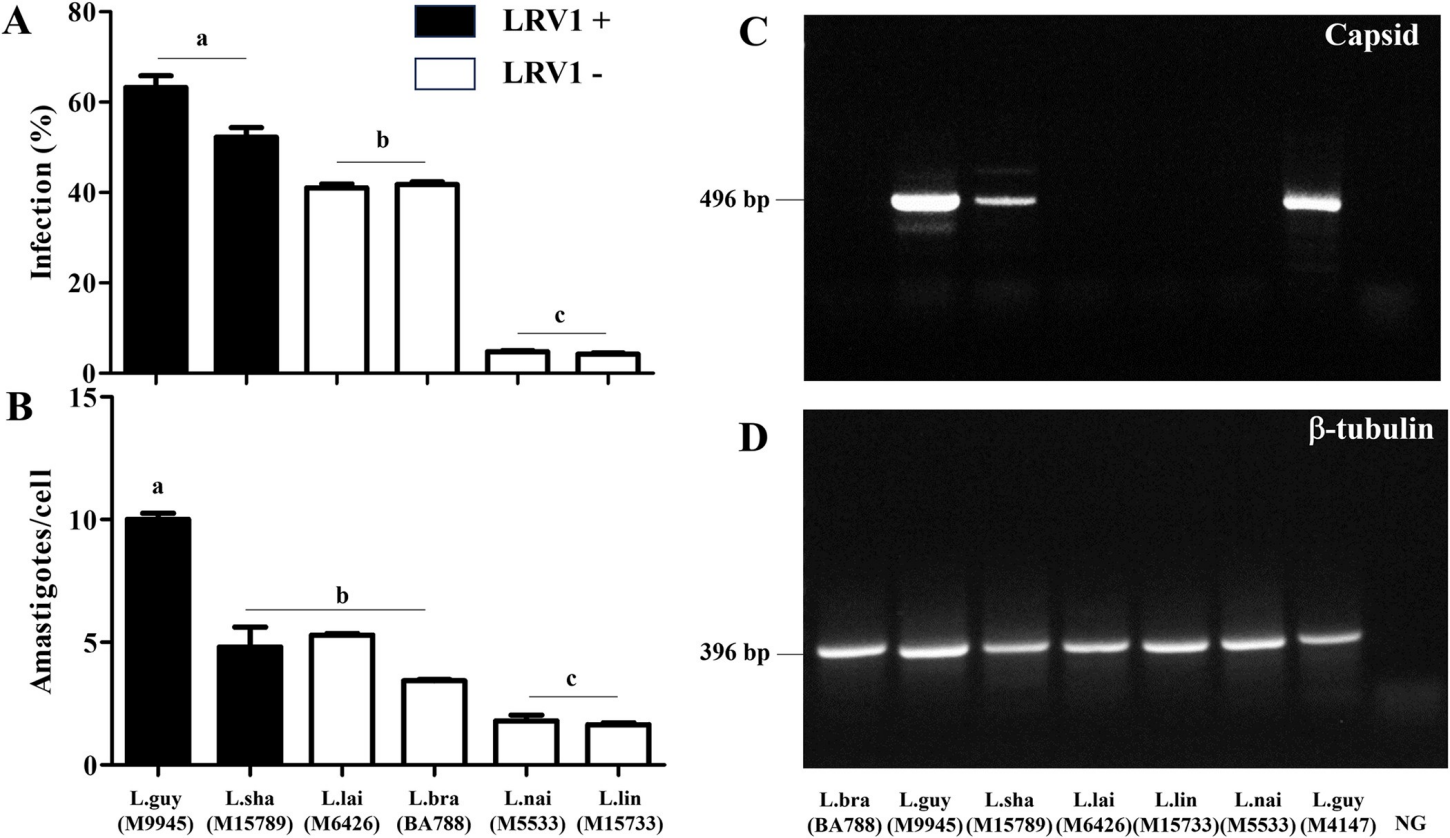
*Leishmania* species shows different levels of infection in resident peritoneal macrophages of *M*. *auratus* in the presence/absence of *Leishmania RNA virus 1* (LRV1). (A) Macrophage infection (%), (B) number of amastigotes per macrophage, (C) LRV1 detection based on amplification of capsid gene and (D) DNA integrity control based on amplification of β-tubulin gene. Legend: L.braz, *L*. *braziliensis*; L.guy, *L*. *guyanensis*; L.sha, *L*. *shawi*, L.lind, *L*. *linderbergi*, L.lai, *L*. *lainsoni*, L.nai, *L*. *naiffi* and NG, negative control. Positive control is represented by *L*. *guyanensis* (L.guy M4147). Dark and white bars indicate LRV1 presence and absence, respectively. The same letters above bars do not indicate statistical differences (P>0.05). Different letters indicate statistical differences (P<0.05).

All strains had variable levels of infectivity with resident macrophages of *M*. *auratus* (Fig 1). *L*. (*V*.) *guyanensis* and *L*. (*V*.) *shawi* had the highest infection levels of macrophages (>50%). *L*. (*V*.) *braziliensis* and *L*. (*V*.) *lainsoni* had moderate infection levels (~40%) whereas *L*. (*V*.) *lindenbergi*/*L*. (*V*.) *naiffi* were the lowest (<6%) (Fig 2A, P<0.05). In respect to amastigote intracellular parasitism *L*. (*V*.) *guyanensis* had the highest number of parasites (~10/cell) (P<0.05). The remaining species exhibited lower numbers of amastigotes ranging from 1.6–1.8 in *L*. (*V*.) *lindenbergi*/*L*. (*V*.) *naiffi* (P>0.05) to 3.4–5.3 for *L*. (*V*.) *shawi*/*L*. (*V*.) *braziliensis*/*L*. (*V*.) *lainsoni* (Fig 2B) (P>0.05). All strains were searched for *Leishmania* RNA virus 1 (LRV1), because its presence was related with *Leishmania* virulence. The reference strain of *L*. (*V*.) *guyanensis* (M4147) was positive for LRV1. LRV1 was detected in *L*. (*V*.) *guyanensis* (M9945) and in *L*. (*V*.) *shawi* (M15789) (Fig 2C) (Table 2). β-tubulin gene confirmed DNA integrity of all strains (Fig 2D). THP-1 cells had a lower infection percentage (11–25%) and number of intracellular amastigotes per cell (1–2) in comparison to hamster resident macrophages (S1A–S1C Fig).

### *Leishmania* (*Viannia*) *guyanensis* caused macroscopical lesions in hamsters

**Fig 3:**
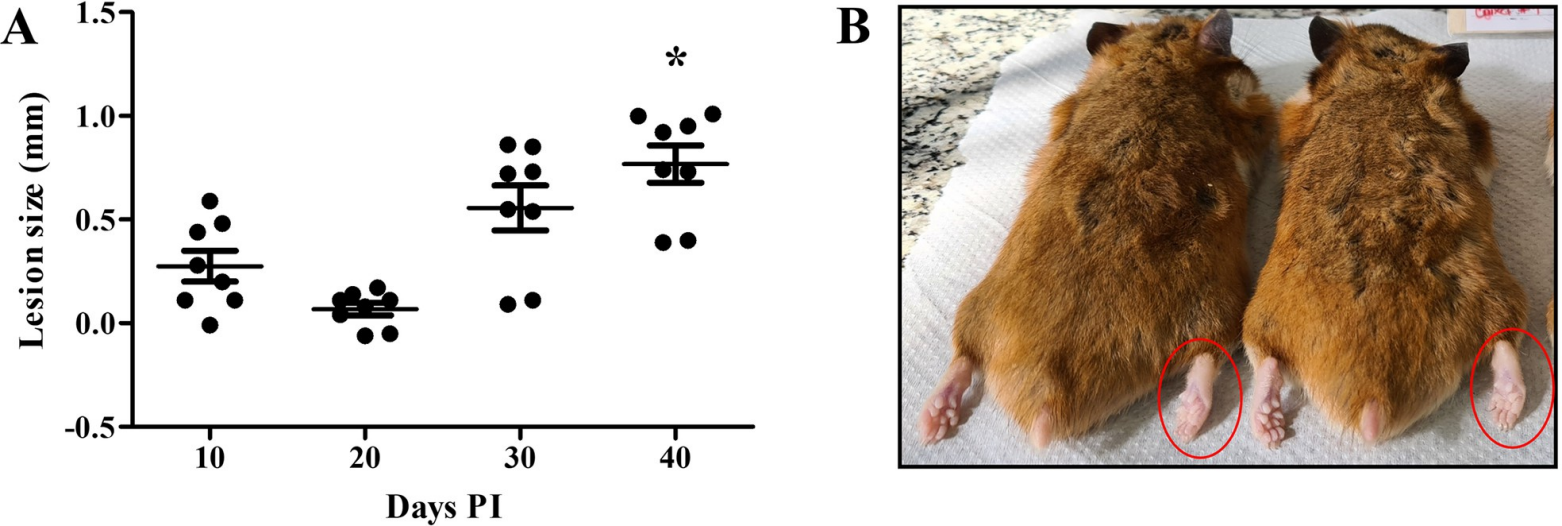
*Leishmania guyanensis* (M9945) in the presence of salivary gland extract (SGE) caused macroscopic lesion in *M*. *auratus* foot pads. (A) Size of the lesion (mm) and (B) macroscopic evaluation of swollen right foot pads (red circles). Asterisk indicates statistical difference in the lesion size between 40- and 10/20- days PI.

Since all strains infected hamster macrophages *in vitro*, the next step was to perform *in vivo* infections of the parasites in the presence of SGEs of *Ny*. *neivai*. Except for *L*. (*V*.) *guyanensis*, all strains failed to cause macroscopic lesions in the foot pad of the hamsters (Fig 3). Although no ulceration was seen, an increase in the swelling of the foot pad was observed after 20 days PI reaching its peak at 40 days PI (Fig 3A) (P<0.05). A representative image of two animals shows right feet pads swollen in comparison to left ones (Fig 3B).

### *L*. (*Viannia*) species exhibited different levels of pathological severity

**Fig 4:**
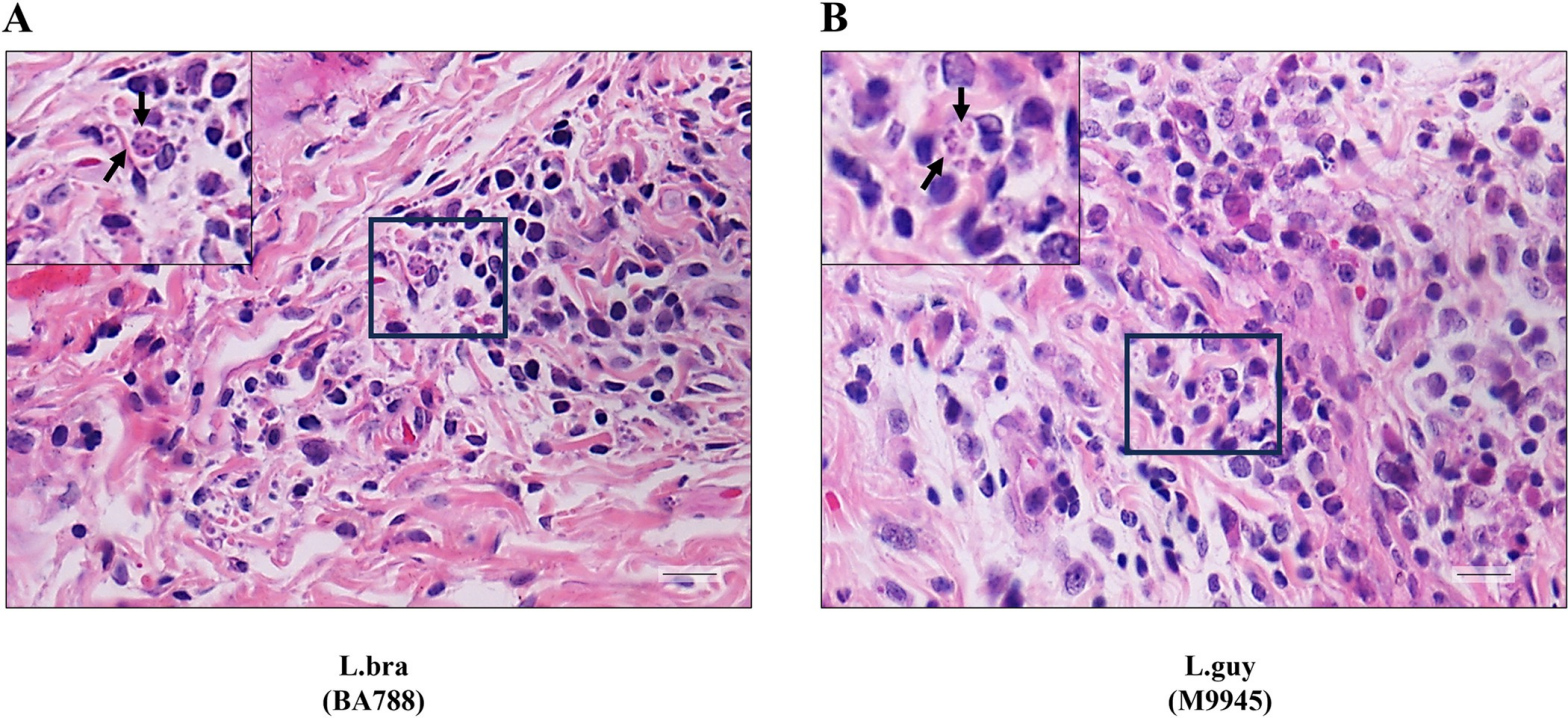
*L*. *braziliensis* (A) and *L*. *guyanensis* (B) had higher levels of mononuclear infection in the dermis of *M*. *auratus* 20 days PI. Left upper sections in higher magnification showing macrophages infected with amastigote forms (dark arrows). Legend: L.braz, *L*. *braziliensis* and L.guy, *L*. *guyanensis*. Scale bar = 20 um.

**Fig 5:**
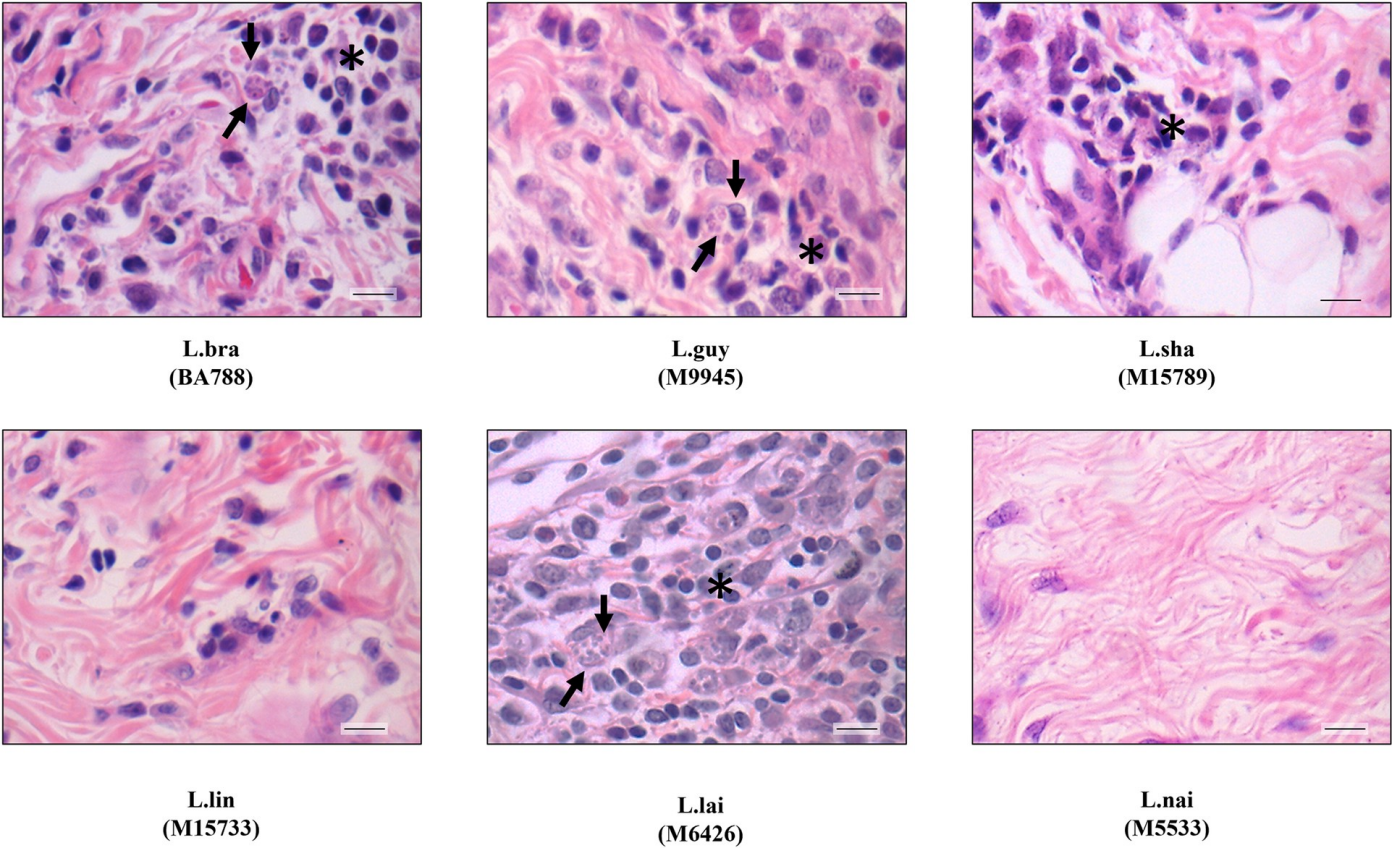
*Leishmania (Viannia*) species show different levels of pathology in the dermis of *M*. *auratus* after 20 days PI. Legend: L.braz, *L*. *braziliensis*; L.guy, *L*. *guyanensis*; L.sha, *L*. *shawi*, L.lind, *L*. *linderbergi*, L.lai, *L*. *lainsoni*, L.nai, *L*. *naiffi*. Scale bar = 20 μm. Dark arrows indicate suggestive intracellular amastigote forms. Asterisks indicate a region with inflammatory infiltrate.

**Fig 6:**
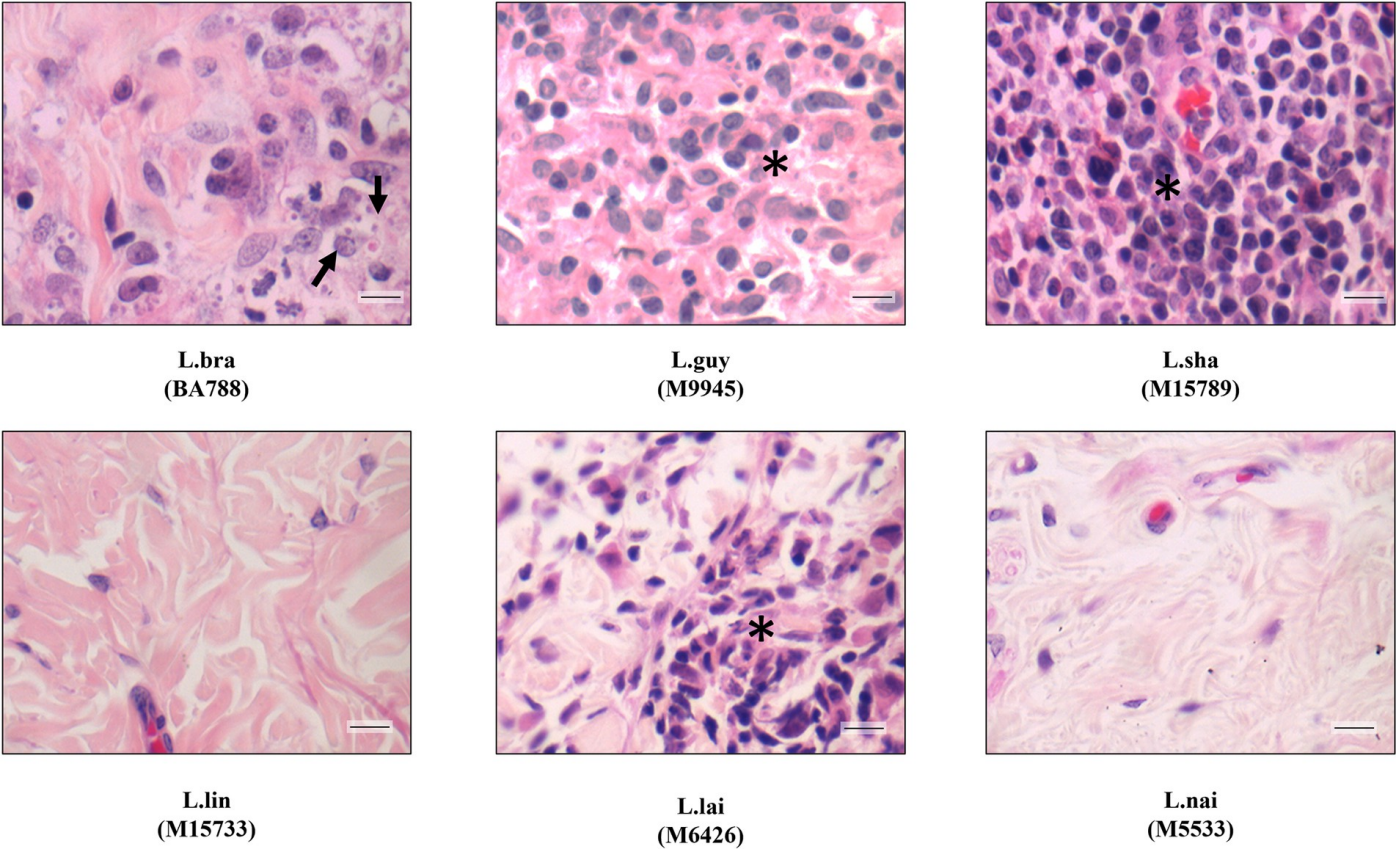
*In vivo* infection by *Leishmania* (*Viannia*) in the dermis of *M*. *auratus* shows decrease in parasitism after 40 days PI. Legend: L.braz, *L*. *braziliensis*; L.guy, *L*. *guyanensis*; L.sha, *L*. *shawi*, L.lind, *L*. *linderbergi*, L.lai, *L*. *lainsoni*, L.nai, *L*. *naiffi*. Scale bar = 20 um. Dark arrows indicate suggestive intracellular amastigote forms. Asterisks indicate a region with inflammatory infiltrate.

**Fig 7:**
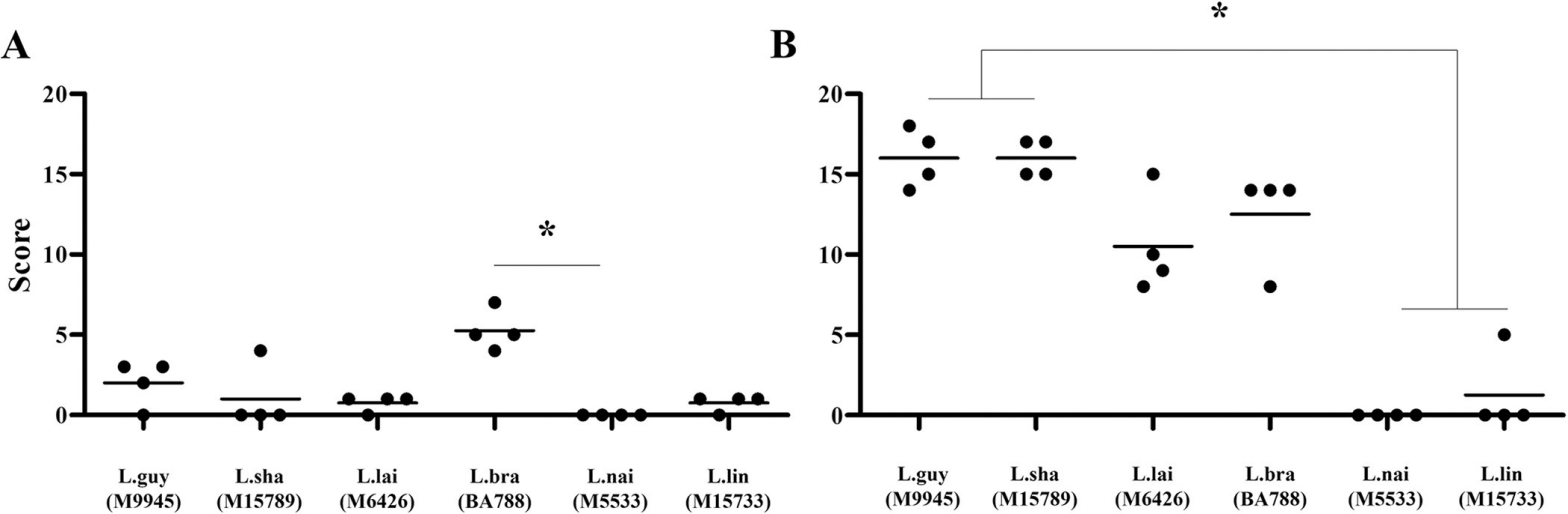
*Leishmania* (*Viannia*) species display different inflammatory scores in the dermis of *M*. *auratus*. (A) 20 days PI and (B) 40 days PI. Legend: L.braz, *L*. *braziliensis*; L.guy, *L*. *guyanensis*; L.sha, *L*. *shawi*, L.lind, *L*. *linderbergi*, L.lai, *L*. *lainsoni*, L.nai, *L*. *naiffi*. Asterisk indicates statistical differences (P<0.05).

After 20- and 40-days PI, hamsters were anesthetized and euthanized prior to foot pad, liver, and spleen removal. Although no external macroscopical ulceration was detected in skin, an inflammatory infiltrate was triggered by some species at variable levels. After 20 days PI, this feature was more perceptible for *L*. (*V*.) *braziliensis*, *L*. (*V*.) *guyanensis* and *L*. (*V*.) *lainsoni* in HE stained section. They produced a strong inflammatory infiltration in the presence of infected mononuclear cells containing amastigotes (black arrows, Figs 4A and 4B, and 5). Although *L*. (*V*.) *shawi* also induced areas of proinflammatory infiltration indicated by the asterisks, no amastigotes were detected after 20 days PI with HE (Fig 5). Finally, *L*. (*V*.) *lindenbergi* and *L*. (*V*.) *naiffi* showed low/absent inflammation, with no detected parasites by HE (Fig 5). After 40 days PI, the proinflammatory infiltrate decreased in *L*. (*V*.) *braziliensis*, although amastigotes were still seen inside some cells. For *L*. (*V*.) *guyanensis*, *L*. (*V*.) *shawi* and *L*. (*V*.) *lainsoni*, the inflammatory infiltrate remained but with no detectable amastigotes by HE (Fig 6). Like previous results *L*. (*V*.) *lindenbergi* and *L*. (*V*.) *naiffi* did not show any signs of inflammation in the dermis 40 days PI. As expected, negative controls did not show histopathological changes in the skin, liver, and spleen (S2A–S2C Fig). The quantification of those dermis infiltrates/parasites was performed by morphometry and inflammatory scores were provided (Fig 7). Here, *L*. (*V*.) *braziliensis* had higher proinflammatory scores compared to the other species after 20 days PI. However, statistical difference was only achieved in comparison to *L*. (*V*.) *naiffi* (Fig 7A, P<0.05). However, this pattern changed after 40 days PI, where *L*. (*V*.) *shawi* and *L*. (*V*.) *guyanensis* reached higher scores compared to *L*. (*V*.) *lindenbergi* and *L*. (*V*.) *naiffi* (Fig 7B, P<0.05).

### *Leishmania* (*Viannia*) *braziliensis* visceralized and induced granuloma development in the liver

**Fig 8:**
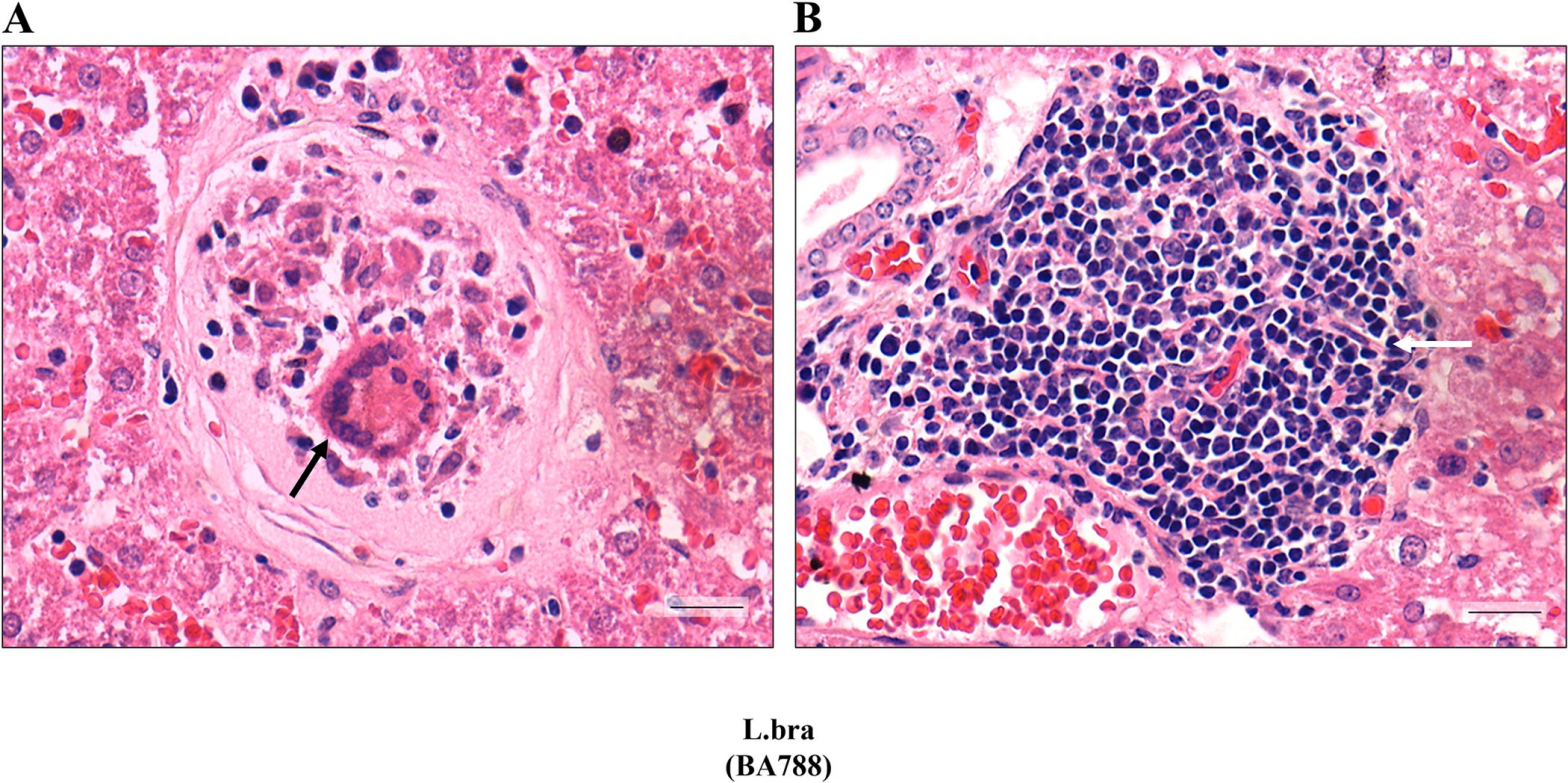
Visceralization of *L*. *braziliensis* in *M*. *auratus* resulted in granuloma-like structures in the liver. (A) Fibrotic granuloma composed of epithelioid macrophages and giant cells (dark arrow) after 20 days PI. (B) Granuloma composed of lymphoid cells and fibroblasts (white arrow) after 40 days PI. Scale bar = 20 um.

Together with skin, liver and spleen were also removed for histopathological analysis. We did not find any major alterations in the viscera of all strains, except for *L*. (*V*.) *braziliensis*. This species caused two types of granulomas in the liver (Fig 8). One was composed of epithelioid macrophages and giant cells surrounded by a dense capsule of fibrotic tissue after 20 days PI (Fig 8A). The other had a nodular arrangement of inflammatory cells containing lymphocytes and fibroblasts (white arrow) after 40 days PI (Fig 8B). However, no parasites were seen in the surrounding areas of those structures in both periods of PI.

### *L*. (*Viannia*) species had different levels of parasitism in the dermis of *M*. *auratus*

**Fig 9:**
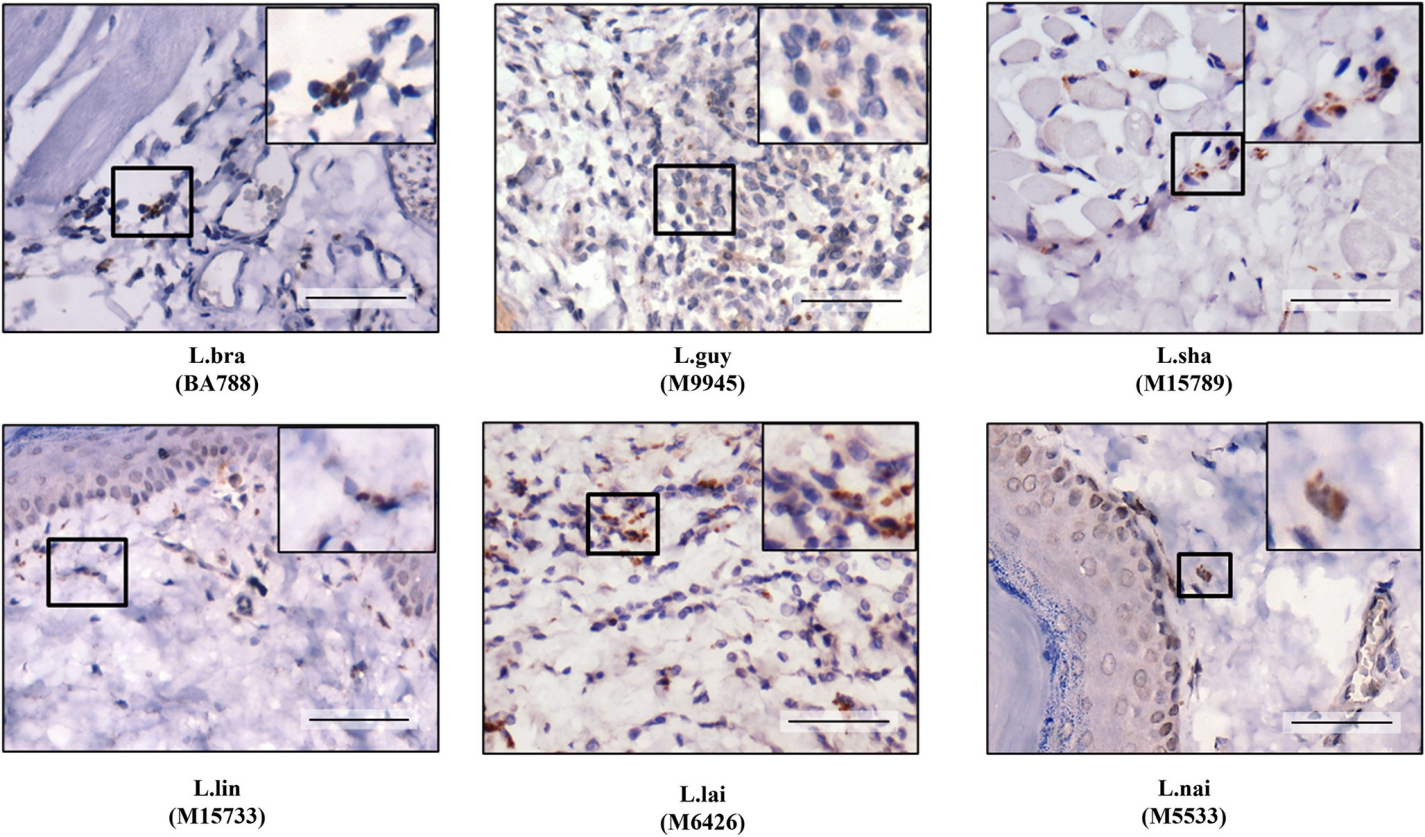
*Leishmania* (*Viannia*) species had different parasite load in the dermis of *M*. *auratus* 20 days PI by immunohistochemistry (IHC). Upper sections in higher magnification showing amastigote forms. Legend: L.braz, *L*. *braziliensis*; L.guy, *L*. *guyanensis*; L.sha, *L*. *shawi*, L.lind, *L*. *linderbergi*, L.lai, *L*. *lainsoni*, L.nai, *L*. *naiffi*. Scale bar = 20 um.

**Fig 10:**
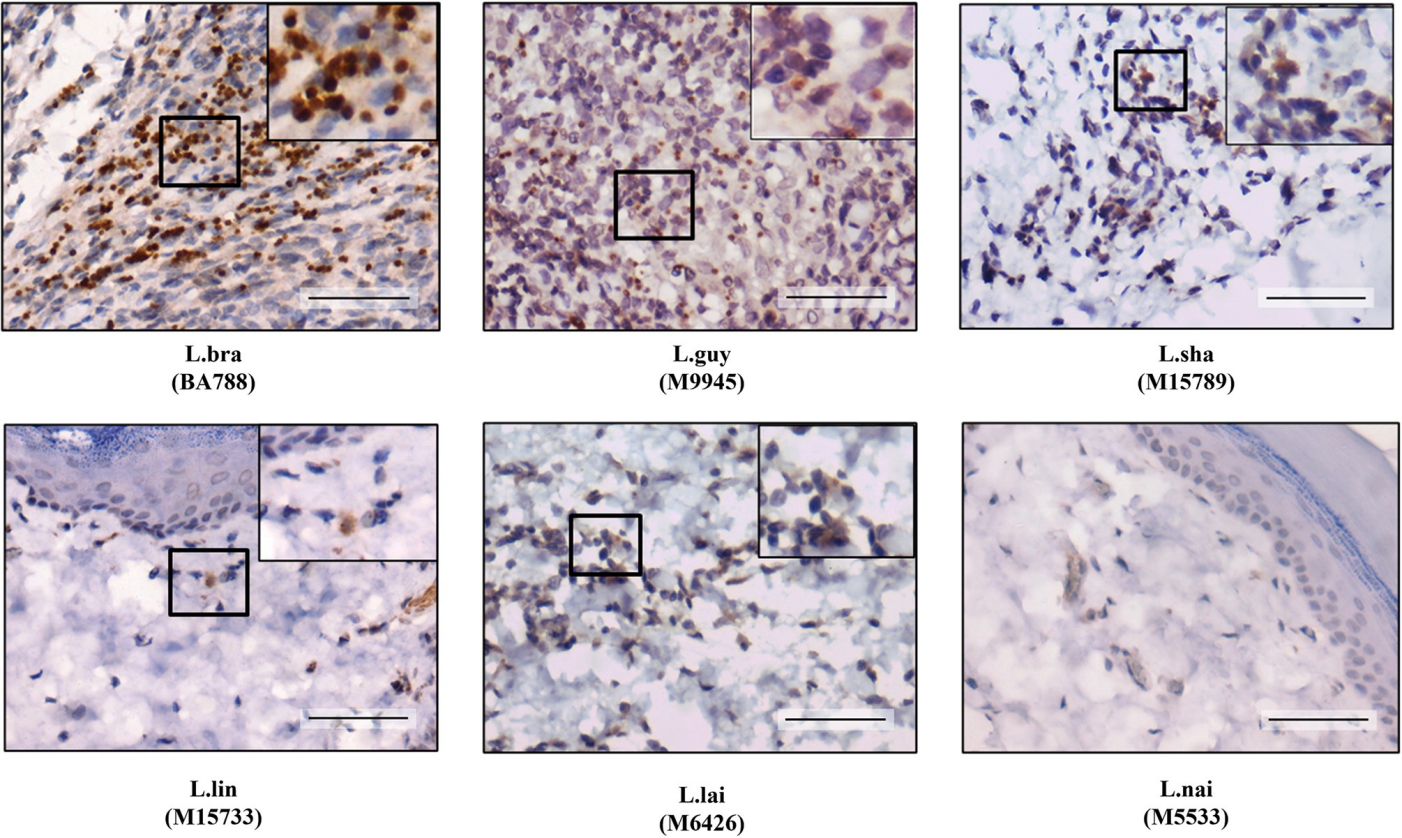
Imunohistochemistry (IHC) of *Leishmania* (*Viannia*) species in the dermis of *M*. *auratus* after 40 days PI shows clearance of *L*. *naiffi* parasites. Upper sections in higher magnification showing amastigote forms. Legend: L.braz, *L*. *braziliensis*; L.guy, *L*. *guyanensis*; L.sha, *L*. *shawi*, L.lind, *L*. *linderbergi*, L.lai, *L*. *lainsoni*, L.nai, *L*. *naiffi*. Scale bar = 20 μm.

Since visible amastigotes were only detected by HE in *L*. (*V*.) *braziliensis*, *L*. (*V*.) *guyanensis* and *L*. (*V*.) *lainsoni* infections, a more sensitive technique, IHC, was used. Unlike HE (Figs 4–6), IHC detected amastigotes in the dermis of animals infected with all species after 20- days PI (Fig 9). After 40 days PI (Fig 10), an increase in the density of parasites was noticed in the dermis of hamsters infected with *L*. (*V*.) *braziliensis*/*L*. *guyanensis* followed by *L*. (*V*.) *shawi*/*L*. (*V*.) *lainsoni*. A few amastigotes were seen in *L*. (*V*.) *lindenbergi* infected hamster, but none were detected in *L*. (*V*.) *naiffi* 40 days PI.

**Fig 11:**
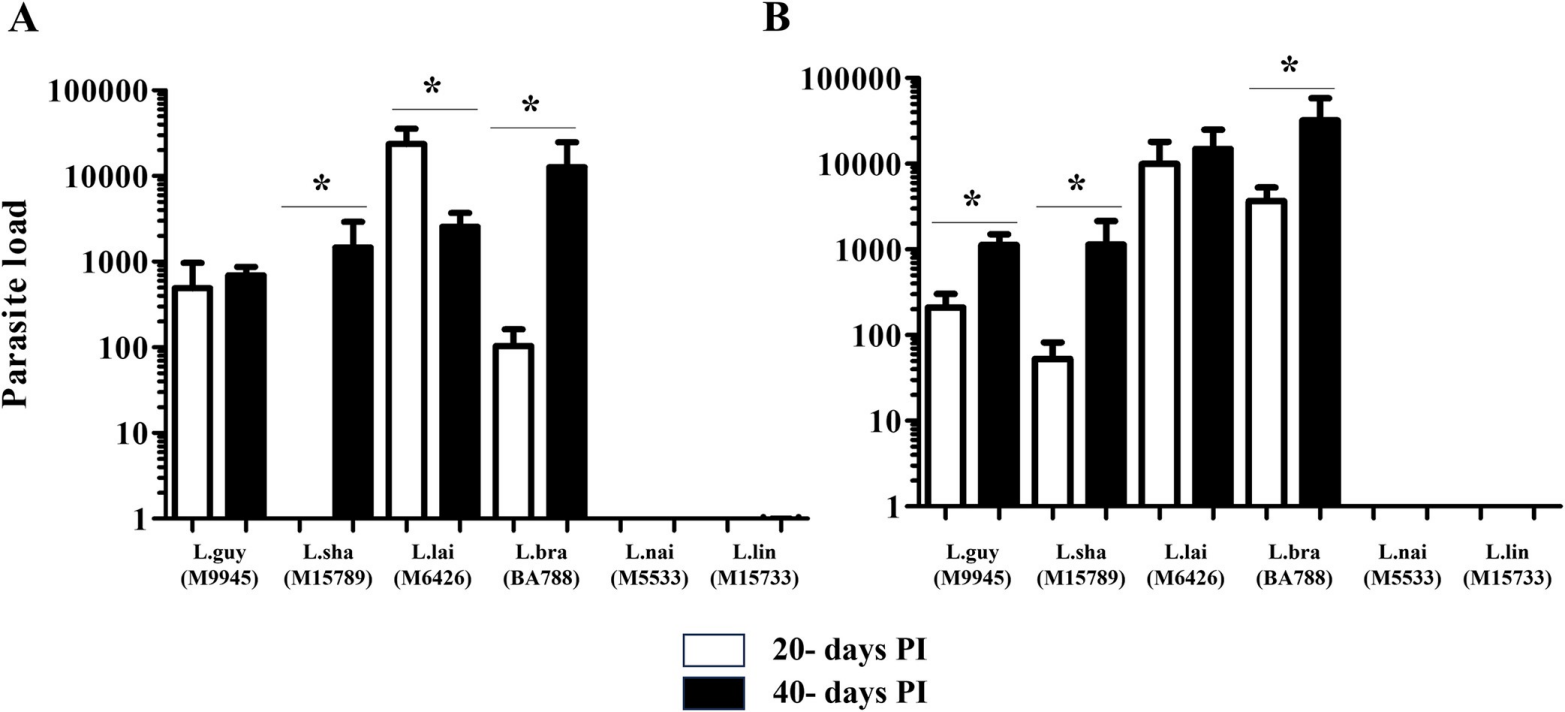
*Leishmania naiffi* and *L*. *lindenbergi* had the lowest parasite load in *M*. *auratus* tissues. Parasite load (parasites/mg tissue) recovered using the limiting dilution method from the dermis (A) and lymph nodes (B) of *M*. *auratus* infected with different *Leishmania*. Legend: L.braz, *L*. *braziliensis*; L.guy, *L*. *guyanensis*; L.sha, *L*. *shawi*, L.lind, *L*. *linderbergi*, L.lai, *L*. *lainsoni*, L.nai, *L*. *naiffi*. White and dark bars indicate 20- and 40- days PI, respectively. Asterisks indicate statistical differences (P<0.05).

We used the limiting dilution assay to improve parasite quantification loads in the foot pad skin and draining lymph nodes. Confirming previous results with HE (Figs 4–6) and IHC (Figs 9–10), we were able to recover parasites from the infected tissues with higher parasite load at both time points (Fig 11A and 11B). In the skin, after 20 days PI, we recovered *L*. (*V*.) *braziliensis*, *L*. (*V*.) *guyanensis* and *L*. (*V*.) *lainsoni*. After 40 days PI, in addition to these same species, we also recovered *L*. (*V*.) *shawi*. (Fig 11A). The same recovery pattern exhibited by the skin was repeated in the lymph nodes, except for *L*. *shawi*, that was also recovered 20 days PI (Fig 11B). In general, parasite load increased with time in both skin and lymph nodes for *L*. (*V*.) *braziliensis*/*L*. (*V*.) *shawi* and *L*. (*V*.) *guyanensis/L*. (*V*.) *shawi/L*. (*V*.) *braziliensis*, respectively (P<0.05). No *L*. (*V*.) *lindenbergi*/*L*. (*V*.) *naiffi* parasites were recovered from any tissue 20- and 40-days PI. All data are summarized in Table 2.

### Glycoconjugates (LPGs and GIPLs) were able to differentially activate murine macrophages *via* TLR4/TLR2

**Fig 12:**
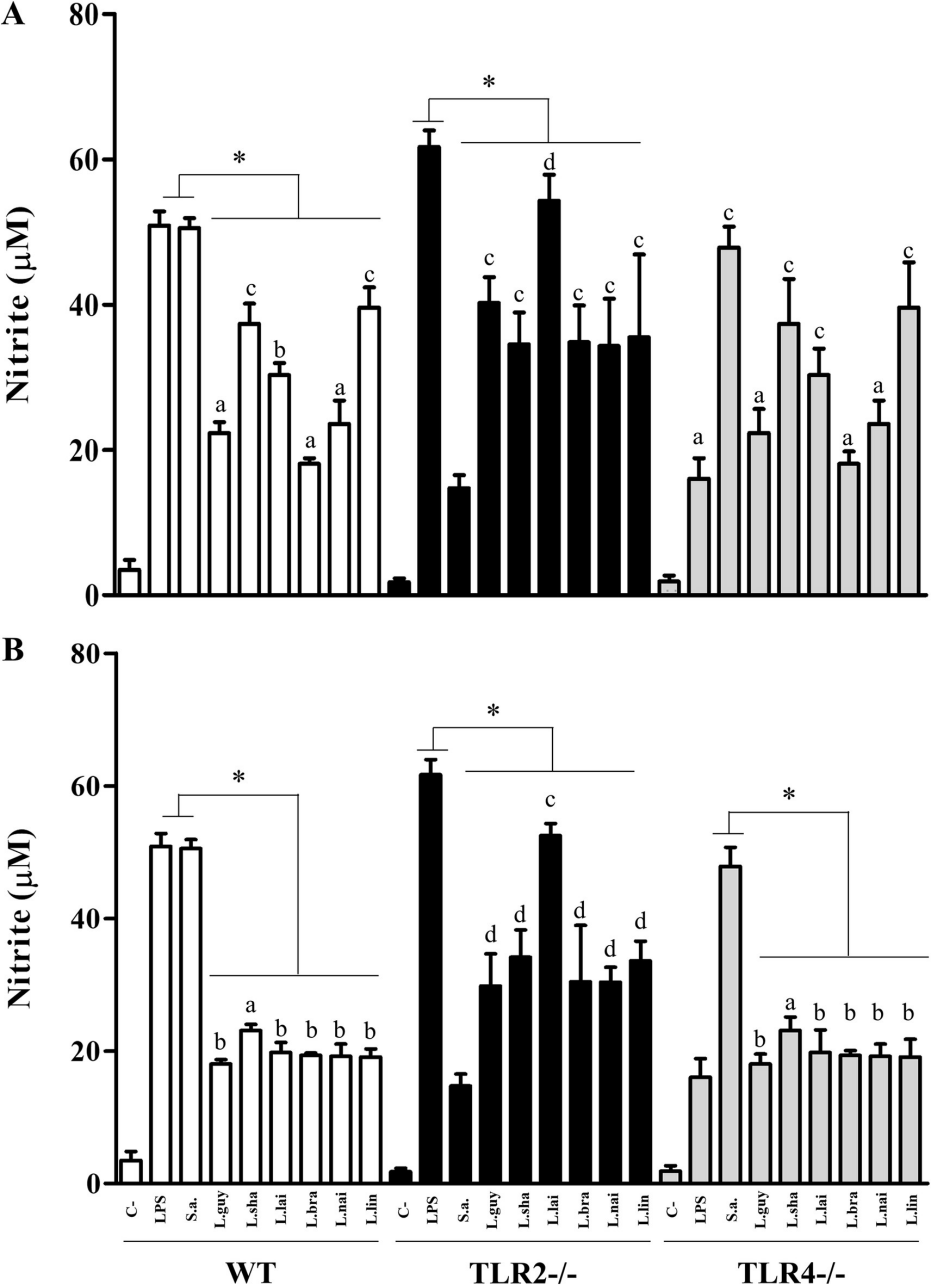
Glycoconjugates (LPGs and GIPLs) from *Leishmania* (*Viannia*) species induced variable levels of Nitric oxide (μM) by C57BL/6 murine macrophages *via* TLR4/TLR2. Wild-type (WT, white bars), TLR2 (-/-) (dark bars) and TLR4 (-/-) (gray bars) knockouts. Macrophages were exposed to lipophosphoglycans (LPGs) (A) and glycoinositolphospholipids (GIPLs) (B) (10 μg/mL) from different *Leishmania*. Legend: C-, negative control (RPMI medium); LPS, lipopolysaccharide (TLR4 agonist, 100 ng/mL); S.a., *Staphylococcus aureus* extract (TLR2 agonist, 100 ng/mL); L.braz, *L*. *braziliensis*; L.guy, *L*. *guyanensis*; L.sha, *L*. *shawi*, L.lind, *L*. *linderbergi*, L.lai, *L*. *lainsoni*, L.nai, *L*. *naiffi*. Asterisks indicate statistical differences within the same mice lineage (P<0.05). The same letters above bars do not indicate statistical differences (P>0.05). Different letters indicate statistical differences (P<0.05).

**Fig 13:**
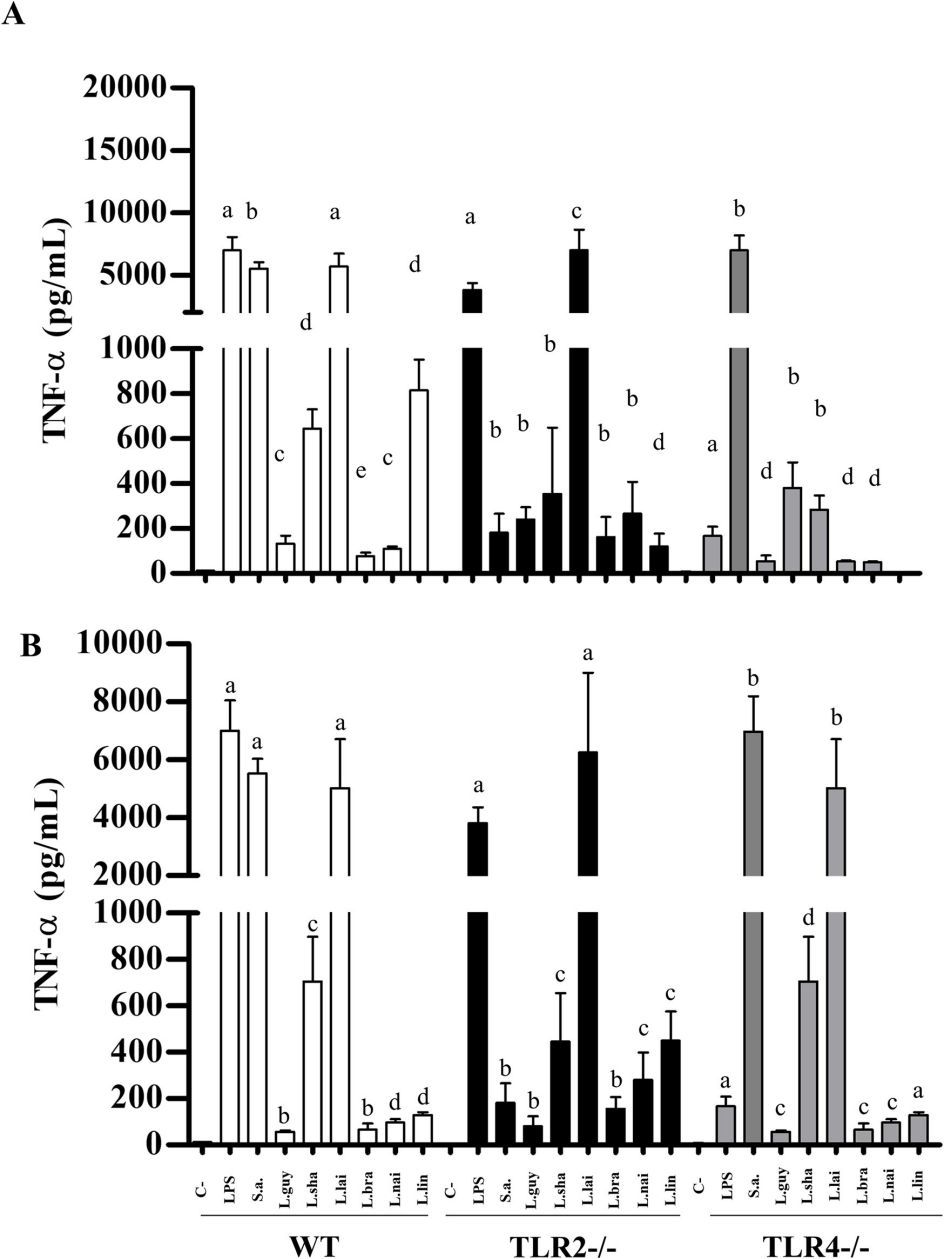
*Leishmania lainsoni* glycoconjugates induced higher TNF-α production by C57BL/6 murine macrophages *via* TLR4/TLR2. Wild-type (WT, white bars), TLR2 (-/-) (dark bars) and TLR4 (-/-) (gray bars) knockouts. Macrophages were exposed to lipophosphoglycans (LPGs) (A) and glycoinositolphospholipids (GIPLs) (B) (10 μg/mL) from different *Leishmania*. Legend: C-, negative control (RPMI medium); LPS, lipopolysaccharide (TLR4 agonist, 100 ng/mL); S.a., *Staphylococcus aureus* extract (TLR2 agonist, 100 ng/mL); L.braz, *L*. *braziliensis*; L.guy, *L*. *guyanensis*; L.sha, *L*. *shawi*, L.lind, *L*. *linderbergi*, L.lai, *L*. *lainsoni*, L.nai, *L*. *naiffi*. Asterisks indicate statistical differences within the same mice lineage (P<0.05). The same letters above bars do not indicate statistical differences (P>0.05). Different letters indicate statistical differences (P<0.05).

**Fig 14:**
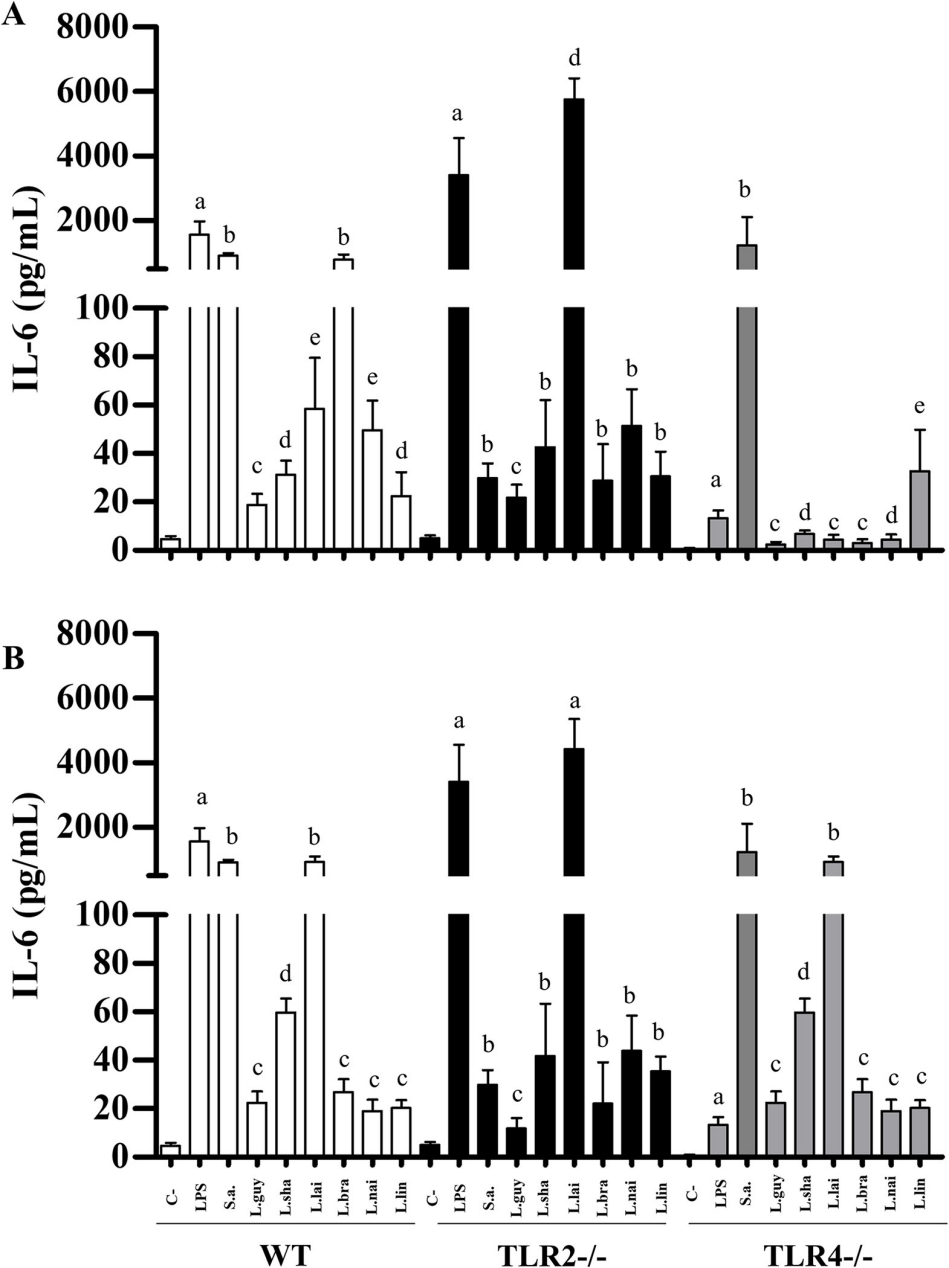
*Leishmania lainsoni* glycoconjugates induced higher IL-6 production by C57BL/6 murine macrophages *via* TLR4/TLR2. Wild-type (WT, white bars), TLR2 (-/-) (dark bars) and TLR4 (-/-) (gray bars) knockouts. Macrophages were exposed to lipophosphoglycans (LPGs) (A) and glycoinositolphospholipids (GIPLs) (B) (10 μg/mL) from different *Leishmania*. Legend: C-, negative control (RPMI medium); LPS, lipopolysaccharide (TLR4 agonist, 100 ng/mL); S.a., *Staphylococcus aureus* extract (TLR2 agonist, 100 ng/mL); L.braz, *L*. *braziliensis*; L.guy, *L*. *guyanensis*; L.sha, *L*. *shawi*, L.lind, *L*. *linderbergi*, L.lai, *L*. *lainsoni*, L.nai, *L*. *naiffi*. Asterisks indicate statistical differences within the same mice lineage (P<0.05). The same letters above bars do not indicate statistical differences (P>0.05). Different letters indicate statistical differences (P<0.05).

**Fig 15:**
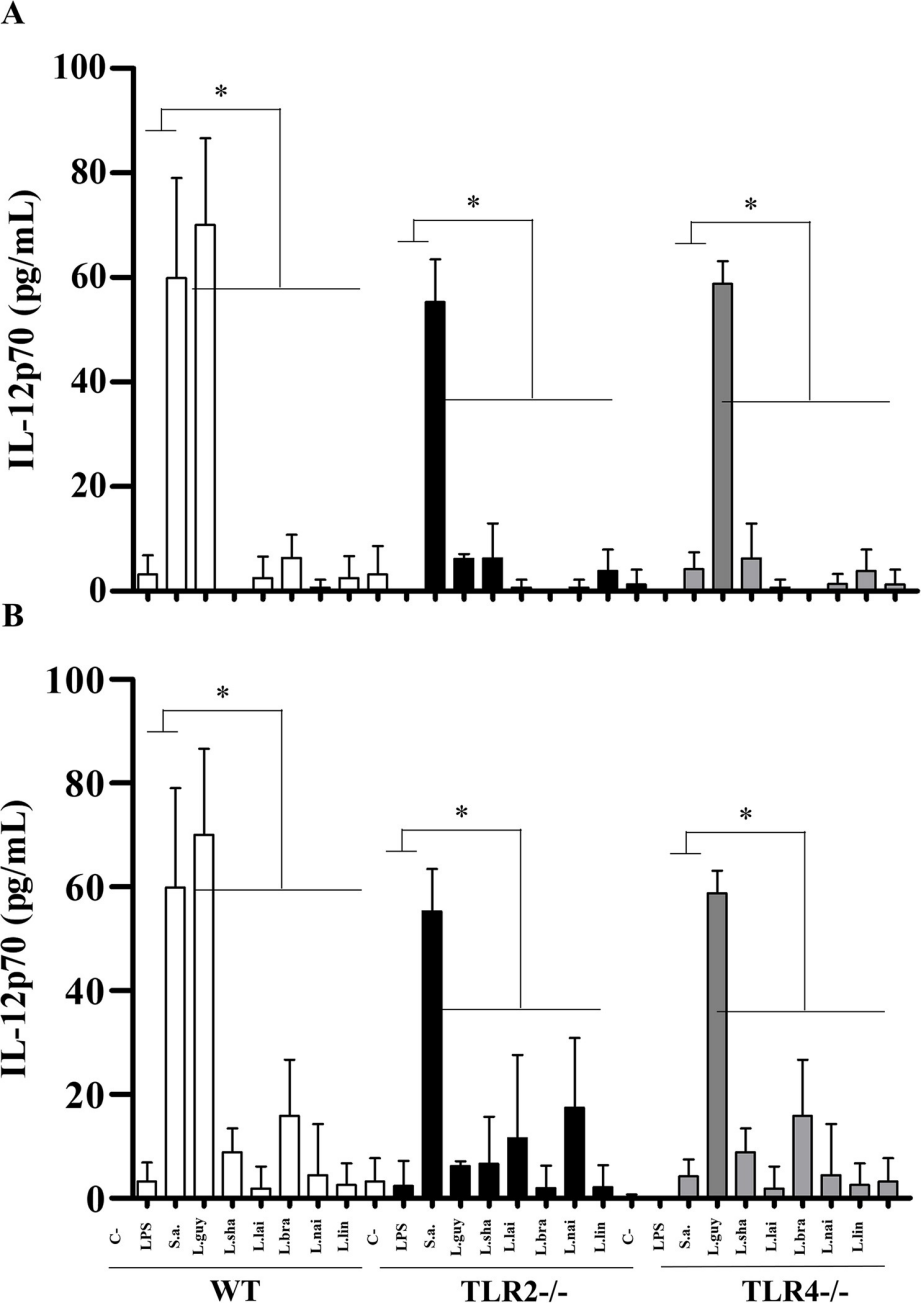
*Leishmania* (*Viannia*) species did not induce considerable levels of IL-12 by C57BL/6 murine macrophages. Wild-type (WT, white bars), TLR2 (-/-) (dark bars) and TLR4 (-/-) (gray bars) knockouts. Macrophages were exposed to lipophosphoglycans (LPGs) (A) and glycoinositolphospholipids (GIPLs) (B) (10 μg/mL) from different *Leishmania*. Legend: C-, negative control (RPMI medium); LPS, lipopolysaccharide (TLR4 agonist, 100 ng/mL); S.a., *Staphylococcus aureus* extract (TLR2 agonist, 100 ng/mL); L.braz, *L*. *braziliensis*; L.guy, *L*. *guyanensis*; L.sha, *L*. *shawi*, L.lind, *L*. *linderbergi*, L.lai, *L*. *lainsoni*, L.nai, *L*. *naiffi*. Asterisks indicate statistical differences within the same mice lineage (P<0.05).

**Fig 16:**
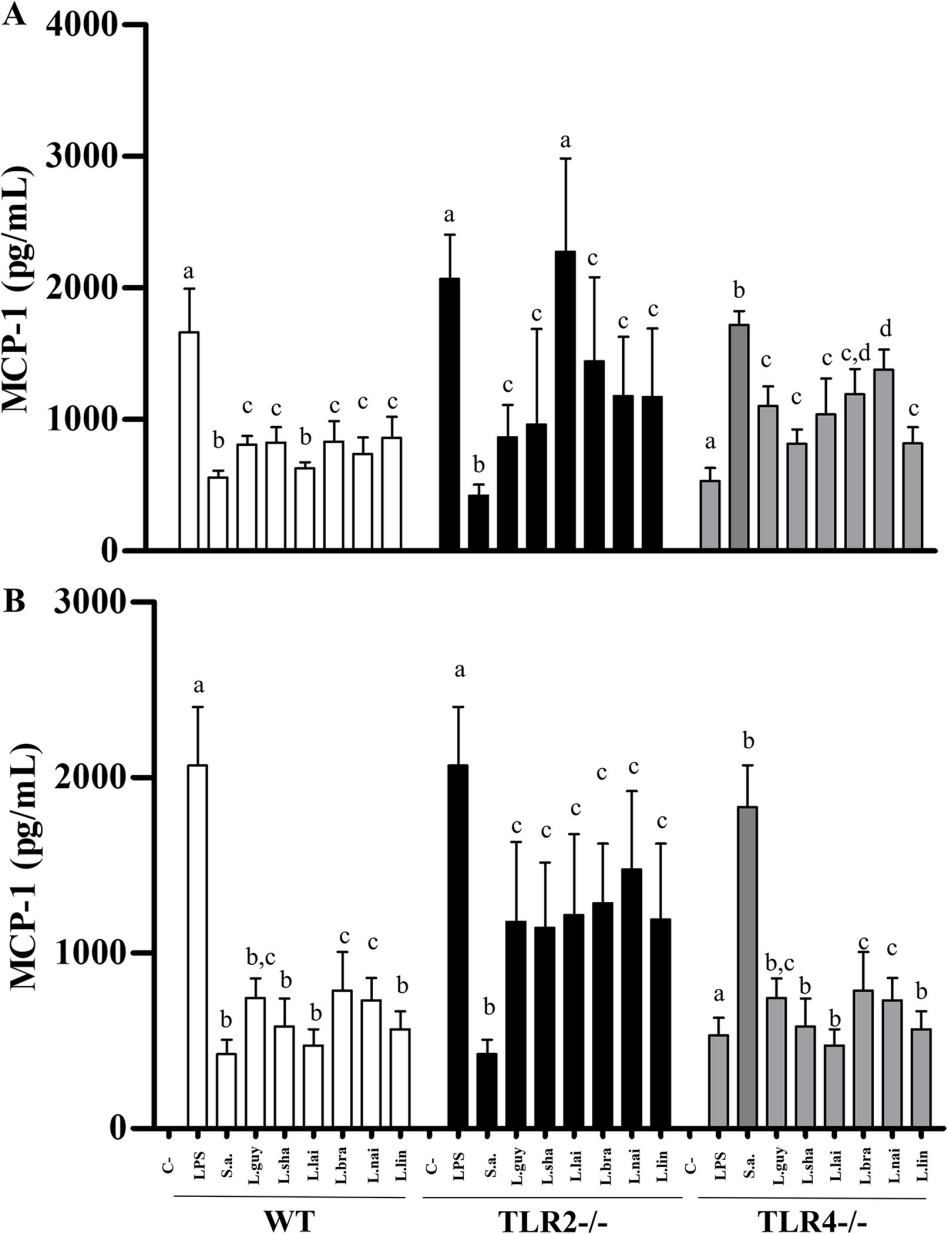
MCP-1 production by C57BL/6 murine macrophages differ in LPG-stimulated but not in GIPLs-stimulated from *Leishmania* (*Viannia*) species. Wild-type (WT, white bars), TLR2 (-/-) (dark bars) and TLR4 (-/-) (gray bars) knockouts. Macrophages were exposed to lipophosphoglycans (LPGs) (A) and glycoinositolphospholipids (GIPLs) (B) (10 μg/mL) from different *Leishmania*. Legend: C-, negative control (RPMI medium); LPS, lipopolysaccharide (TLR4 agonist, 100 ng/mL); S.a., *Staphylococcus aureus* extract (TLR2 agonist, 100 ng/mL); L.braz, *L*. *braziliensis*; L.guy, *L*. *guyanensis*; L.sha, *L*. *shawi*, L.lind, *L*. *linderbergi*, L.lai, *L*. *lainsoni*, L.nai, *L*. *naiffi*. Asterisks indicate statistical differences within the same mice lineage (P<0.05). The same letters above bars do not indicate statistical differences (P>0.05). Different letters indicate statistical differences (P<0.05).

LPG and GIPLs differentially stimulated murine macrophages. Functionally, they were able to induce NO and cytokines/chemokine showing variations in their agonistic proinflammatory activities. LPGs from all *L*. (*Viannia*) species induced NO *via* TLR4/TLR2, and this ability varied within the three mice lineages. In WT and TLR4 (-/-), LPGs from *L*. (*V*.) *shawi* and *L*. (*V*.) *lindenbergi* induced higher levels of NO, whereas for TLR2 (-/-), *L*. (*V*.) *lainsoni* had higher pro-inflammatory activity (Fig 12A, P<0.05). For GIPLs, a similar pattern was observed (Fig 12B). GIPLs from *L*. (*V*.) *shawi* induced a significant level of NO compared to all species in WT and TLR4 (-/-) (P<0.05). However, for TLR2 (-/-), higher levels of NO were detected in all species, but the highest production was *L*. (*V*.) *lainsoni* (Fig 12, P<0.05). As expected, for WT, both positive controls (LPS and *S*. *aureus* extract) induced higher levels of NO and cytokines/chemokine (P<0.05). For TLR2 (-/-), LPS induced higher levels of NO, whereas *S*. *aureus* extract did not. On the other hand, for TLR4 (-/-), LPS induced lower levels of NO compared to *S*. *aureus* extract (Figs 12–16, P<0.05). For the cytokines/chemokine, in general, LPGs had an agonistic activity *via* TRL4 and secondarily *via* TLR2. On the other hand, GIPLs had a more pro-inflammatory activity *via* TLR4. Sometimes, glycoconjugates frow *L*. (*V*.) *shawi* also had considerable proinflammatory activity, especially for TNF-α (Fig 13, p<0.05) and IL-6 (Fig 14, p<0.05). The remaining four species had lower proinflammatory activity either for LPGs or GIPLs (Figs 13–16, P<0.05). IL-12 production was very low for all *Viannia* glycoconjugates (Fig 15, P<0.05). All data are summarized in Table 3.

### Type II LPG in *L*. (*V*.) *lainsoni* had higher pro-inflammatory activity in murine macrophages

**Fig 17:**
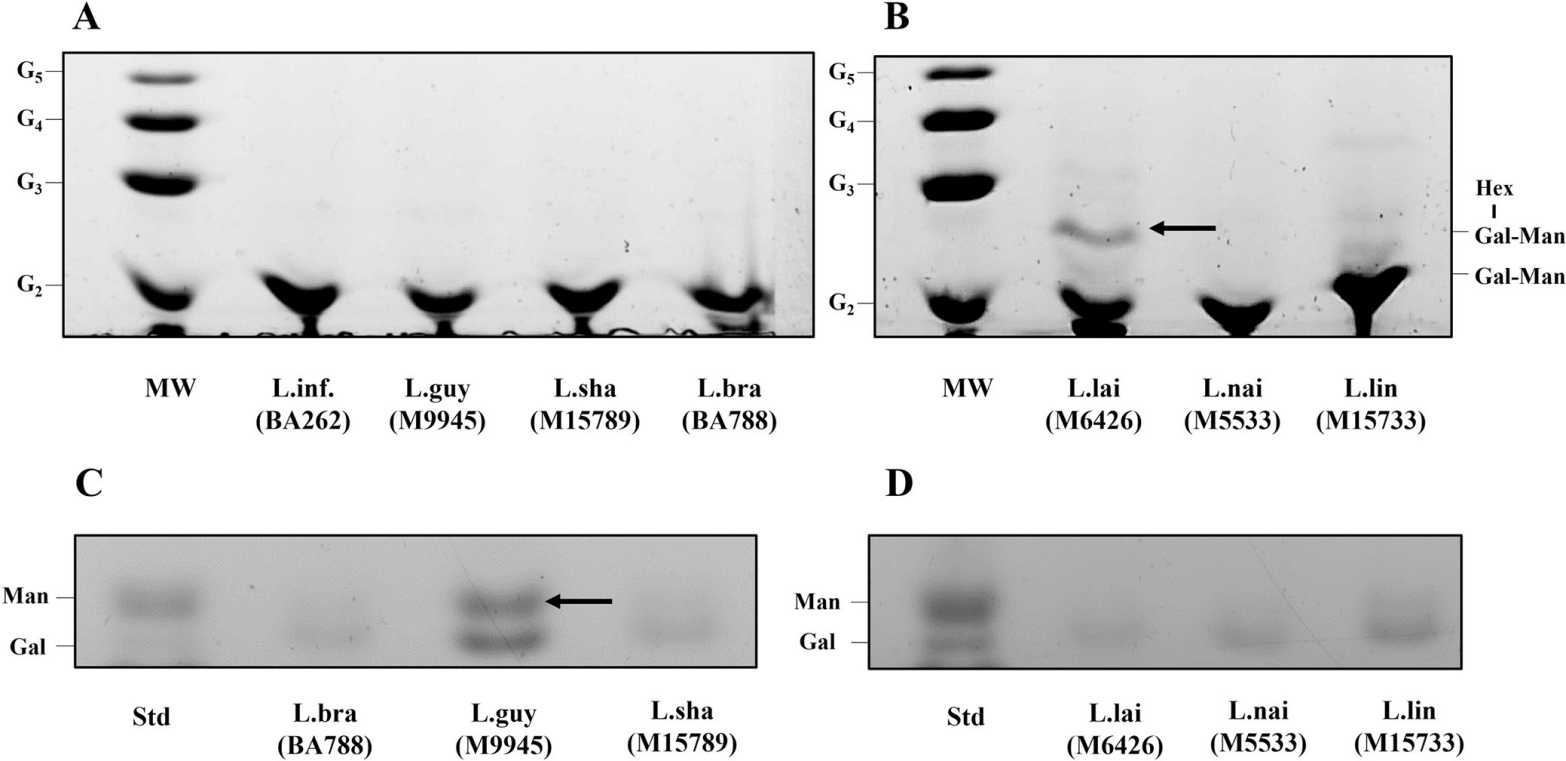
Glycoconjugates of *Leishmania* (*Viannia*) species display interspecies polymorphisms in their carbohydrate’s motifs. Fluorophore-assisted carbohydrate electrophoresis (FACE) analysis of depolymerized glycoconjugates (lipophosphoglycans and glycoinositolphospholipids) from *Leishmania*. A) and B), polysaccharide profile of dephosphorylated repeat units of LPGs; C) and D), monosaccharide profile of GIPLs. Legend: L.inf, *L*. *infantum* (type I LPG—control), L.braz, *L*. *braziliensis*; L.guy, *L*. *guyanensis*; L.sha, *L*. *shawi*; L.lind, *L*. *linderbergi*; L.lai, *L*. *lainsoni*; L.nai, *L*. *naiffi*; MW, molecular weight represented by oligoglucose ladder G_2_-G_5_; Std, monosaccharide standard represented by galactose and mannose; Gal, galactose; Man, Mannose and Hex–Hexose.

To investigate whether the variations in the functional properties of glycoconjugates were due to biochemical polymorphisms, they were depolymerized for biochemical analysis (Fig 17). Lipophosphoglycan repeat units were purified and subjected to FACE analysis. Most of repeat units co-migrated with oligosaccharide standard G_2_ and type I control from *L*. (*L*.) *infantum* (BA262) confirming the Gal(β1,4)Man structure common to all LPGs (Fig 17A and 17B). All species, except *L*. (*V*.) *lainsoni*, had LPG devoid of sidechains (type I) (Fig 17A and 17B). *L*. (*V*.) *lainsoni* had one side-chain co-migrating close to oligo-glucose ladder G_3_, suggestive of a hexose sidechain (Type II LPG) (Fig 17B, dark arrow). Like LPGs, most GIPLs structures were not polymorphic (Fig 17C and 17D). Most GIPLs were rich in galactose (type II) and only *L*. (*V*.) *guyanensis* was rich in galactose/mannose (type I or hybrid) (Fig 17C, dark arrow). All data are summarized in Table 3.

### *Leishmania* (V*iannia*) *lainsoni* clustered differently in comparison to other *L*. (*Viannia*) species

**Fig 18:**
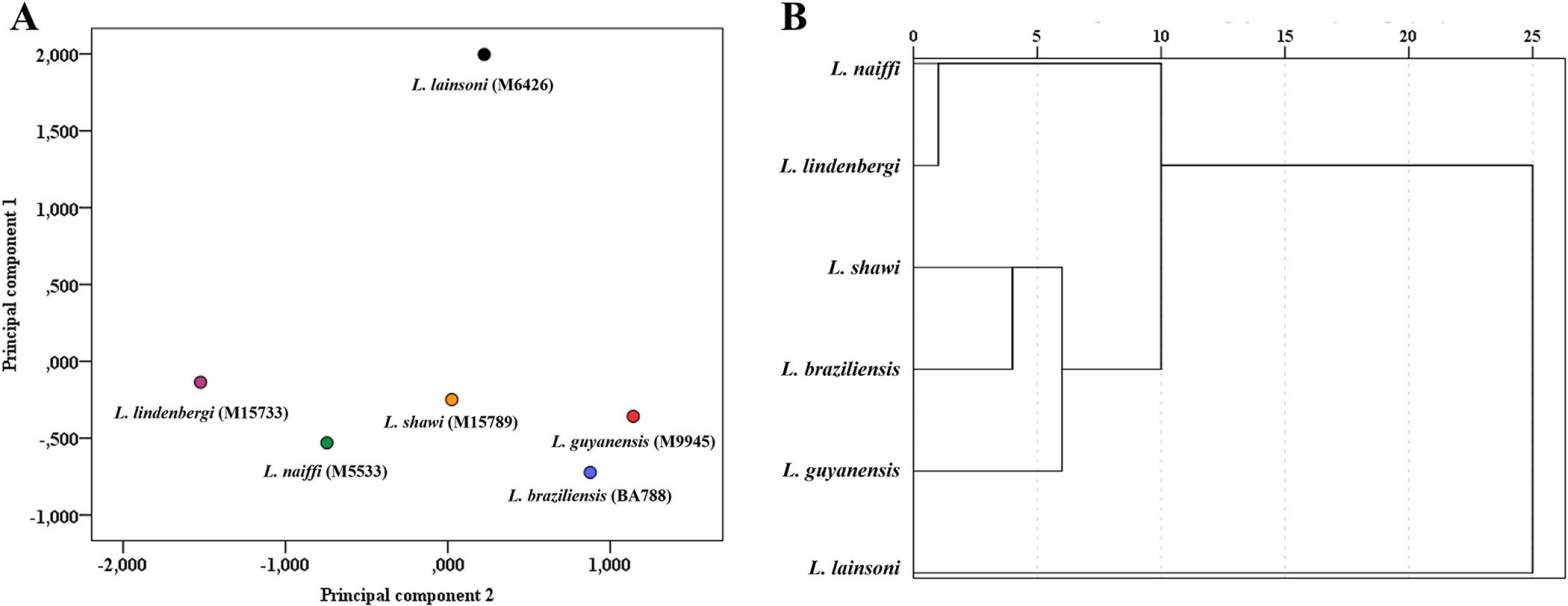
*Leishmania lainsoni* is clustered separately from other members within the *Viannia* subgenus. Principal component analysis (PCA) derived from 33 variables (Tables 2 and 3). A) Dotplot graphic showing distinct biological patterns among the different *Viannia* species. B) Hierarchical cluster by squared-Euclidean distance using the first two PCs indicating the proximity of the six *Viannia* species and the formation of an isolated cluster composed of *L*. *lainsoni*.

The biological, biochemical, and immunological parameters studied in this paper were collected and summarized in Tables 2 and 3. To provide a better understanding of the taxonomic relationships among the *L. (Viannia)* species, Principal Component Analysis (PCA) was performed, and we found that they clustered into two groups (Fig 18A). One was composed by *L. (V.) lainsoni* (cluster 1) and another by the five remaining species (cluster 2). After hierarchical cluster by squared-Euclidean distance, cluster 2 was splitted into two subdivisions: *L. (V.) lindenbergi/L. (V.) naiffi* and *L. (V.) guyanensis/L. (V.) shawi/L. (V.) braziliensis* (Fig 18B).

## Discussion

Several factors affect infectivity and/or pathogenicity amongst *Leishmania* strains/species. In our search for a model to study those species, we used different *L*. (*Viannia*) strains evaluating their ability to infect two types of macrophages *in vitro* including resident peritoneal (hamster) and continuous cell line THP-1 (human). Previous studies with other species/strains of *Leishmania* demonstrated that *L*. (*L*.) *infantum* (dermotropic/viscerotropic), *L*. (*L*.) *amazonensis* and *L*. (*V*.) *braziliensis* were able to successfully infect hamsters and THP-1 macrophages [32,39,40]. Here, all species were able to infect both cell types displaying variability in their intracellular parasitism. Resident peritoneal macrophages were more susceptible to infection than THP-1 cells exhibiting higher parasitism depending on the *Leishmania* species. In resident peritoneal macrophages, *L*. (*V*.) *guyanensis* and *L*. (*V*.) *shawi* were the most infective. *L*. (*V*.) *naiffi* and *L*. (*V*.) *lindenbergi* were the least infective species for both cell types. Regarding intracellular development, *L*. (*V*.) *guyanensis* had a higher amastigote number (~2-3-fold) than the other species. These data advocate the use of resident peritoneal macrophages from hamsters instead of THP-1 cells. The two less infective species, *L*. (*V*.) *naiffi* and *L*. (*V*.) *lindenbergi* still warrant a better model of cellular infection. In nature, *L*. (*V*.) *guyanensis* strains are often infected with the *Leishmania RNA virus*, whereas *L*. (*V*.) *braziliensis* and *L*. (*L*.) *infantum* are not [46,47,49–51]. The literature suggests a correlation between LRV1 presence and severity of disease leading to mucosal development for humans [16]. We found this virus in *L*. (*V*.) *guyanensis* and *L*. (*V*.) *shawi*. The former had a higher viral load and intracellular infection than the latter. Although it is tempting to suggest that LRV1 presence may increase infectivity/intracellular parasitism, at least for *L*. (*V*.) *guyanensis*, the present study did not find a coherent correlation between LRV1 presence and invasion/intracellular replication in our *in vitro* models. Our observations that these *L*. (*Viannia*) species developed more efficiently in hamster cells, led us to use *M*. *auratus* for our *in vivo* experiments.

The novelty of our work was to use for the first time SGE from *Ny*. *neivai* since saliva compounds have several immunomodulatory activities [43,52–54]. All strains had low ability to develop macroscopic ulcerated lesions in hamsters´ footpads. The only species that caused external visible swelling was *L*. (*V*.) *guyanensis* that increased over time. Like *in vitro* experiments, in our experimental *in vivo* conditions, LRV1 could not solely explain variations in pathology among species/strains, since LRV1-positive *L*. (*V*.) *shawi* did not cause external lesion either. However, while analyzing the microscopic dermis of infected footpads this picture changed. At both PI intervals, very productive inflammatory reactions in the dermis for some *L*. (*Viannia*) species were noticed by HE and IHC. We observed a strong inflammatory exudate for at least four species including *L*. (*V*.) *braziliensis*, *L*. (*V*.) *guyanensis*, *L*. (*V*.) *shawi* and *L*. (*V*.) *lainsoni*. Those species were even recovered after limiting dilution method from skin and draining lymph nodes. No inflammatory infiltration was seen in *L*. (*V*.) *naiffi*/*L*. (*V*.) *lindenbergi* after 40 days PI suggesting clearance of those parasites. Altogether, that data confirmed that for at least four *L*. (*Viannia*) species were able to trigger important immunopathological responses in the dermis of *M*. *auratus* in the absence of macroscopical external lesions. A distinguished feature of *L*. (*V*.) *braziliensis* strain used here was its ability to visceralize. It is already known that depending on the inoculum, that dermotropic species possess different abilities to colonize animal viscera. Several reports already demonstrate this phenomenon for other strains of *L*. (*V*.) *braziliensis* [33], *L*. (*M*.) *enriettii* [55], *L*. (*L*.) *amazonensis* [56] and *L*. (*L*.) *infantum* [45]. Here, we have shown that although most *L*. (*Viannia*) strains had variable levels of pathology in the dermis, only *L*. (*V*.) *braziliensis* visceralized to the liver evoking two types of lesions with presence of giant cells, lymphocytes, and fibroblasts. Consistent with previous observations, in the liver, a certain degree of infection control is detected due to the development of granulomas, this may justify the complete absence of parasites in those lesions [32,57,58]. However, no visible dermotropic lesions appeared in this species during the time proposed (up to 7 weeks PI). We therefore suggest that for this strain (BA788) a longer period of 8–10 weeks should be performed to circumvent the possible lack of apparent lesions.

Like *in vitro* data, *in vivo* assays confirmed that all six *L*. (*Viannia*) species varied in their ability to cause infection in hamsters. *L*. (*V*.) *lindenbergi* and *L*. (*V*.) *naiffi*) had low ability to sustain infection in those animals and there is still the need to find alternative hosts. Regarding the other species, except for *L*. (*V*.) *guyanensis*, that caused apparent macroscopic swelling, the hamster model showed that parasites were microscopically viable even at 40-days PI. For those remaining four species, the hamster model could provide information on inflammatory events, cellular attraction and even visceralization. Since variable levels of infectivity and proinflammatory activity were reported in the previous findings, our next step was to investigate how parasite virulence factors could modulate such events.

*Leishmania* species possess a complex array of surface molecules acting as Pathogen-Associated Molecular Patterns (PAMPs). They include glycoprotein 63 (GP-63), lipophosphoglycans (LPGs) and glycoinositolphospholipids (GIPLs) [59–61]. TLR2 and TLR4 are the main receptors recognized by LPGs and GIPLs and the ability to induce proinflammatory responses by several species was dependent on the species strains [37,38,62–65]. Here, glycoconjugates from all *L*. (*Viannia*) species demonstrated the ability to trigger cytokine/chemokine and NO production like other dermotropic species strains of *Leishmania*. Confirming previous data with New World *L*. (*Viannia*) and *L*. (*Leishmania*) species that *L*. (*Viannia*) LPGs were also able to activate TLR4 and secondarily TLR2 [37,40,65]. Interestingly, this activation pattern was more homogeneous for the GIPLs, that were primarily TLR4 agonists like those from *L*. (*L*.) *infantum*/*L*. (*V*.) *braziliensis* and *Trypanosoma cruzi* [62,66]. However, both glycoconjugates were not potent IL-12 inducers confirming the role of this important cytokine during *Leishmania* interaction with the host. The low ability of *Leishmania* glycoconjugates in inducing this cytokine may suggest an escape mechanism of those parasites during early events in the innate immune compartment [67]. For the other mediators, in general, a higher agonistic activity was observed for *L*. (*V*.) *lainsoni* and sometimes for *L*. (*V*.) *shawi*/*L*. (*V*.) *lindenbergi* glycoconjugates, especially for NO and TNF-α. The variable potential of *L*. (*Viannia*) glycoconjugates corroborates the immunopathology caused by these dermotropic species. They often cause ulcerated lesions in the patient’s skin and some of them can even lead to mucosal involvement [16,68]. This is different from the dermotropic *L*. (*L*.) *amazonensis* LPG that can trigger strong proinflammatory responses but does not translocate NF-kB [64]. Here, even though we could not observe productive macroscopical lesions in the hamsters’ skin, we detected microscopical exudates and inflammatory infiltration suggesting the involvement of macrophages and several mediators during infection. However, our next step was to biochemically characterize those glycoconjugates to establish a possible functional relationship between structure and activity.

LPG and GIPLs showed variations in structures of *L*. (*V*.) *lainsoni* LPG and *L*. (*V*.) *guyanensis* GIPLs. Of all *L*. (*Viannia*) species reported here, only *L*. (*V*.) *braziliensis* had its LPG/GIPL characterized. LPGs from this species are from three types (I-III) depending on the sidechains bearing the conserved Gal(β1,4) Man(α1)-PO_4_ repeat unit motif [37,69]. The GIPLs from *L*. (*V*.) *braziliensis* are mostly galactosylated and considered type II [62]. Here, we provide for the first time the structures of the repeat units of other *L*. (*Viannia*) species. Most of the LPGs are devoid of side chains (type I), except for that of *L*. (*V*.) *lainsoni* that showed a polymorphism having a hexose sidechain (type II). Type I LPG is the most common and reported for in several species/strains of *Leishmania* [37,38,62,64,69]. The *L*. (*V*.) *lainsoni* LPG is very similar to those reported for *L*. (*L*.) *mexicana* [70] and *L*. (*L*.) *infantum* (strain PP75) [35], who also possess a hexose (glucose) as sidechains. With respect to GIPLs, except for *L*. (*V*.) *guyanensis*, most of them were galactosylated (Type II). A prominent mannose band detected by FACE analysis in addition to the galactose band is suggestive of a Type I or hybrid GIPL commonly observed previously for *L*. (*L*.) *infantum/L*. (*L*.) *donovani* [62] and *L*. (*L*.) *tropica/L*. (*L*.) *aethiopica* [71]. Overall, the present data indicates that the polymorphisms detected in the carbohydrate motifs were very low. Except for *L*. (*V*.) *lainsoni* LPG whose polymorphism in the carbohydrate motif resulted in higher proinflammatory activity, this was not very clear for *L*. (*V*.) *guyanensis* type I/hybrid GIPL. This is in accordance with previous literature on glycobiology suggesting that other parts of the glycoconjugates, e.g. lipids, also have proinflammatory activity [62,72]. However, it is very clear that *L*. (*Viannia*) glycoconjugates have a pivotal role during early events of *Leishmania* infection in the innate immune compartment.

The present multidisciplinary study used several biological, biochemical, and immunological parameters to elucidate the parasite/mammalian-host interface of the less common *L*. (*Viannia*) species. All data were collected, summarized, and subjected to a PCA analysis to understand possible taxonomical relationships among *L*. (*Viannia*) strains. Confirming previous questions in literature [24,73] the PCA analysis, based on the parameters evaluated in the present study, showed that *L*. (*V*.) *lainsoni* is separated from the other *L*. (*Viannia*) species. We conclude that phenotypic and intrinsic characters show that *L*. (*V*.) *lainsoni* belongs to the viannian clade but is distinct from all other *L*. (*Viannia*) species. This opens the possibility for WGS analysis for a better understanding of its taxonomic position within the *L*. (*Viannia*) subgenus.

In conclusion, at least four *L*. (*Viannia*) species display polymorphisms in their abilities to infect and sustain infection in *M*. *auratus* upon needle injection in the presence of sandfly SGE. This advocates the use of this model for four *L*. (*Viannia*) species specially to understand their pathological mechanisms in the dermis. Their glycoconjugates display low polymorphisms but possess high proinflammatory agonistic activity *via* TLR4/TLR2. The establishment of a good animal model would be important not only to understand host-parasite relationships but also to provide *in vivo* testing of new drugs for chemotherapy. Besides, the ability of the glycoconjugates from all *Leishmania Viannia* species in inducing proinflammatory responses in macrophages suggest their possible use as vaccines adjuvants.

## Supporting information

S1 Fig *Leishmania* (*Viannia*) species display lower levels of infection in THP-1 macrophages and lack correlation with LRV1 presence. (A) Macrophage infection (%), (B) number of amastigotes per macrophage and (C) Intracellular amastigote forms in THP-1 macrophages. Legend: L.braz, *L*. *braziliensis*; L.guy, *L*. *guyanensis*; L.sha, *L*. *shawi*, L.lind, *L*. *linderbergi*, L.lai, *L*. *lainsoni* and L.nai, *L*. *naiffi*. Magnification of 1000x. Dark and white bars indicate LRV1 presence and absence, respectively. Letters above bars indicate statistical differences (P<0.05).
(TIF)

S2 Fig **Representative negative controls of skin (A), liver (B) and spleen (C) of uninfected *M*. *auratus* stained with hematoxylin-eosin (HE).** Scale bar = 20 μm.
(TIF)

S1 File Raw data and descriptive statistics. (DOCX)

## References

[1] Maia-Elkhoury ANS E. Yadón Z, Idali Saboyá Díaz M, de Fátima de Araújo Lucena F, Gerardo Castellanos L et al. Exploring spatial and temporal distribution of cutaneous leishmaniasis in the Americas, 2001–2011. PLoS Negl Trop Dis. 2016;10: e0005086. doi: 10.1371/journal.pntd.0005086 27824881 PMC5100938

[2] Alvar J, Vélez ID, Bern C, Herrero M, Desjeux P, Cano J, et al. Leishmaniasis worldwide and global estimates of its incidence. PLoS One. 2012;7: e35671. doi: 10.1371/journal.pone.0035671 22693548 PMC3365071

[3] Rangel EF, Lainson R, Carvalho BM, Costa SM, Shaw JJ. Sand fly vectors of American cutaneous leishmaniasis in Brazil. Brazilian Sand Flies: biology, taxonomy, medical importance and control. 2018; 341–380.

[4] Brandão-Filho SP, Carvalho FG de, de Brito MEF, Almeida F de A, Nascimento LA. American cutaneous leishmaniasis in Pernambuco, Brazil: eco-epidemiological aspects in" Zona da Mata" region. Mem Inst Oswaldo Cruz. 1994;89: 445–449. doi: 10.1590/s0074-02761994000300028 7476230

[5] Tonelli GB, Tanure A, Rego FD, Carvalho GM de L, Stumpp R, Assimos GR, et al. *Leishmania* (*Viannia*) *braziliensis* infection in wild small mammals in ecotourism area of Brazil. PLoS One. 2017;12: e0190315. 29284049 10.1371/journal.pone.0190315 PMC5746269

[6] Shaw JJ, Marinho-Júnior JF, Courtenay O, Brandão-Filho SP. Assessing reservoir host status in leishmaniasis with special reference to the infectiousness of *Leishmania* (*Viannia*) braziliensis infections in wild rodents. Rev Soc Bras Med Trop. 2023;56: e0503–2023. 38126376 10.1590/0037-8682-0503-2023 PMC10726968

[7] Quaresma PF, Rêgo FD, Botelho HA, da Silva SR, Moura AJ, Neto RGT, et al. Wild, synanthropic and domestic hosts of Leishmania in an endemic area of cutaneous leishmaniasis in Minas Gerais State, Brazil. Trans R Soc Trop Med Hyg. 2011;105: 579–585. doi: 10.1016/j.trstmh.2011.07.005 21890159

[8] Brandão-Filho SP, Dantas-Torres F, Tolezano JE, Shaw JJ. Hospedeiros reservatórios de Leishmania spp. associados à leishmaniose tegumentar americana, com especial ênfase no Brasil. Leishmanias e a leishmaniose tegumentar Americana. 2011;1: 1–236.

[9] Neves RL, Cardoso DT, Rêgo FD, Gontijo CMF, Barbosa DS, Soares RP. A follow-up study (2007–2018) on American Tegumentary Leishmaniasis in the municipality of Caratinga, Minas Gerais State, Brazil: Spatial analyses and sand fly collection. PLoS Negl Trop Dis. 2021;15: e0009429. doi: 10.1371/journal.pntd.0009429 34003866 PMC8162678

[10] Neves RL, Ker FTO, Dutra-Rêgo F, Rugani JMN, Andrade Filho JD, Soares RP, et al. Increased Risk of American Tegumentary Leishmaniasis in an Urban and Rural Area of Caratinga, Brazil between 2016 and 2021. Am J Trop Med Hyg. 2023;109: 791. doi: 10.4269/ajtmh.23-0017 37640295 PMC10551075

[11] Barbosa DS, Belo VS, Rangel MES, Werneck GL. Spatial analysis for identification of priority areas for surveillance and control in a visceral leishmaniasis endemic area in Brazil. Acta Trop. 2014;131: 56–62. doi: 10.1016/j.actatropica.2013.12.002 24342506

[12] Camara Coelho LI, Paes M, Guerra JA, Barbosa M das G, Coelho C, Lima B, et al. Characterization of *Leishmania* spp. causing cutaneous leishmaniasis in Manaus, Amazonas, Brazil. Parasitol Res. 2011;108: 671–677. 21072540 10.1007/s00436-010-2139-9 PMC3040821

[13] Ready PD, Arias JR, Freitas RA. A pilot study to control Lutzomyia *umbratilis* (Diptera: Psychodidae), the major vector of *Leishmania brazileiensis guyanensis*, in a peri-urban rainforest of Manaus, Amazonas State, Brazil. Mem Inst Oswaldo Cruz. 1985;80: 27–36. 4088044 10.1590/s0074-02761985000100005

[14] Soares RP, Nogueira PM, Secundino NF, Marialva EF, Ríos-Velásquez CM, Pessoa FAC. *Lutzomyia umbratilis* from an area south of the Negro River is refractory to in vitro interaction with *Leishmania guyanensis*. Mem Inst Oswaldo Cruz. 2018;113: 202–205. 29412360 10.1590/0074-02760170425 PMC5804313

[15] Ives A, Ronet C, Prevel F, Ruzzante G, Fuertes-Marraco S, Schutz F, et al. Leishmania RNA virus controls the severity of mucocutaneous leishmaniasis. Science (1979). 2011;331: 775–778. doi: 10.1126/science.1199326 21311023 PMC3253482

[16] Cantanhêde LM, da Silva Júnior CF, Ito MM, Felipin KP, Nicolete R, Salcedo JMV, et al. Further evidence of an association between the presence of Leishmania RNA virus 1 and the mucosal manifestations in tegumentary leishmaniasis patients. PLoS Negl Trop Dis. 2015;9: e0004079. doi: 10.1371/journal.pntd.0004079 26372217 PMC4570810

[17] Correa-Cárdenas CA, Pérez J, Patino LH, Ramírez JD, Duque MC, Romero Y, et al. Distribution, treatment outcome and genetic diversity of *Leishmania* species in military personnel from Colombia with cutaneous leishmaniasis. BMC Infect Dis. 2020;20: 1–11. doi: 10.1186/s12879-020-05529-y 33297972 PMC7724885

[18] Ducharme O, Simon S, Ginouves M, Prévot G, Couppie P, Demar M, et al. *Leishmania naiffi* and *lainsoni* in French Guiana: Clinical features and phylogenetic variability. PLoS Negl Trop Dis. 2020;14: e0008380. 32797078 10.1371/journal.pntd.0008380 PMC7449503

[19] Lainson R, Shaw JJ. *Leishmania* (*Viannia*) *naiffi* sp. n., a parasite of the armadillo, *Dasypus novemcinctus* (L.) in Amazonian Brazil. Ann Parasitol Hum Comp. 1989;64: 3–9. doi: 10.1051/parasite/19896413 2930120

[20] Silva ANR, Júnior AMP, de Paulo PFM, da Silva MS, Castro TS, da Silva Costa G, et al. Detection of *Leishmania* species (Kinetoplastida, Trypanosomatidae) in phlebotomine sand flies (Diptera, Psychodidae) from Porto Velho, Northern Brazil. Acta Trop. 2021;213: 105757. 33189711 10.1016/j.actatropica.2020.105757

[21] Silveira FT, Shaw JJ, Braga RR, Ishikawa E. Dermal leishmaniasis in the Amazon region of Brazil: *Leishmania* (*Viannia*) *lainsoni* sp. n., a new parasite from the State of Pará. Mem Inst Oswaldo Cruz. 1987;82: 289–291. doi: 10.1590/s0074-02761987000200018 3506634

[22] Silveira FT, Lainson R, Shaw JJ, Braga RR, Ishikawa EEA, Souza AAA. Leishmaniose cutânea na Amazônia: isolamento de *Leishmania* (*Viannia*) lainsoni do roedor *Agouti paca* (Rodentia: Dasyproctidae), no Estado do Pará, Brasil. Rev Inst Med Trop Sao Paulo. 1991;33: 18–22. 1843391 10.1590/s0036-46651991000100004

[23] Lainson R, Shaw JJ, Souza AAA, Silveira FT, Falqueto A. Further observations on *Lutzomyia ubiquitalis* (Psychodidae: Phlebotominae), the sandfly vector of *Leishmania* (*Viannia*) *lainsoni*. Mem Inst Oswaldo Cruz. 1992;87: 437–439. 1343653 10.1590/s0074-02761992000300016

[24] Corrêa JR, Brazil RP, Soares MJ. *Leishmania* (*Viannia*) *lainsoni* (Kinetoplastida: Trypanosomatidae), a divergent *Leishmania* of the *Viannia* subgenus: a mini review. Mem Inst Oswaldo Cruz. 2005;100: 587–592. 16302071 10.1590/s0074-02762005000600014

[25] Lainson R, Braga RR, De Souza AAA, Povoa MM, Ishikawa EAY, Silveira FT. *Leishmania* (*Viannia*) shawi sp. n., a parasite of monkeys, sloths and procyonids in Amazonian Brazil. Ann Parasitol Hum Comp. 1989;64: 200–207. 2504099 10.1051/parasite/1989643200

[26] Shaw JJ, Ishikawa EAY, Lainson R, Braga RR, Silveira FT. Cutaneous leishmaniasis of man due to *Leishmania* (*Viannia*) *shawi* Lainson, de Souza, Pόvoa, Ishikawa & Silveira, in Pará State, Brazil. Ann Parasitol Hum Comp. 1991;66: 243–246. 1822654 10.1051/parasite/1991666243

[27] Silveira FT, Ishikawa EAY, De Souza AAA, Lainson R. An outbreak of cutaneous leishmaniasis among soldiers in Belém, Pará State, Brazil, caused by *Leishmania* (*Viannia*) *lindenbergi* n. sp.-a new leishmanial parasite of man in the Amazon region. Parasite. 2002;9: 43–50. 11938695 10.1051/parasite/200209143

[28] Cantanhêde LM, Mattos CB, de Souza Ronconi C, Filgueira CPB, da Silva Júnior CF, Limeira C, et al. First report of *Leishmania* (*Viannia*) *lindenbergi* causing tegumentary leishmaniasis in the Brazilian western Amazon region. Parasite. 2019;26. 10.1051/parasite/2019030 PMC6532396 31120019

[29] Organization WH. Report of a meeting of the WHO Expert Committee on the Control of Leishmaniases, Geneva, Switzerland, 22–26 March 2010. WHO technical report series. 2010.

[30] Miao J, Chard LS, Wang Z, Wang Y. Syrian hamster as an animal model for the study on infectious diseases. Front Immunol. 2019;10: 457882. 10.3389/fimmu.2019.02329 PMC6781508 31632404

[31] Melby PC, Chandrasekar B, Zhao W, Coe JE. The hamster as a model of human visceral leishmaniasis: progressive disease and impaired generation of nitric oxide in the face of a prominent Th1-like cytokine response. The Journal of Immunology. 2001;166: 1912–1920. doi: 10.4049/jimmunol.166.3.1912 11160239

[32] Laurenti MD, Sotto MN, Corbett CE, Da Matta VL, Duarte M. Experimental visceral leishmaniasis: sequential events of granuloma formation at subcutaneous inoculation site. Int J Exp Pathol. 1990;71: 791. 2278823 PMC2002383

[33] Rêgo FD, da Rocha Lima ACVM, Pereira AAS, Quaresma PF, Pascoal-Xavier MA, Shaw JJ, et al. Genetic variant strains of *Leishmania* (*Viannia*) *braziliensis* exhibit distinct biological behaviors. Parasitol Res. 2018;117: 3157–3168. 30022292 10.1007/s00436-018-6014-4

[34] Silva AG, Valverde JG, Romão RPR, Pereira RMP, Cruz AM da. Golden hamster (*Mesocricetus auratus*) as an experimental model for Leishmania (Viannia) braziliensis infection. Parasitology. 2013;140: 771–779. 23369503 10.1017/S0031182012002156

[35] Soares RPP, Macedo ME, Ropert C, Gontijo NF, Almeida IC, Gazzinelli RT, et al. *Leishmania chagasi*: lipophosphoglycan characterization and binding to the midgut of the sand fly vector *Lutzomyia longipalpis*. Mol Biochem Parasitol. 2002;121: 213–224. 12034455 10.1016/s0166-6851(02)00033-6

[36] Garcia L, Kindt A, Bermudez H, Llanos-Cuentas A, De Doncker S, Arevalo J, et al. Culture-independent species typing of neotropical *Leishmania* for clinical validation of a PCR-based assay targeting heat shock protein 70 genes. J Clin Microbiol. 2004;42: 2294–2297. 15131217 10.1128/JCM.42.5.2294-2297.2004 PMC404633

[37] Vieira T da S, Rugani JN, Nogueira PM, Torrecilhas AC, Gontijo CMF, Descoteaux A, et al. Intraspecies polymorphisms in the lipophosphoglycan of *L*. *braziliensis* differentially modulate macrophage activation via TLR4. Front Cell Infect Microbiol. 2019;9: 240. 31355149 10.3389/fcimb.2019.00240 PMC6636203

[38] Coelho-Finamore JM, Freitas VC, Assis RR, Melo MN, Novozhilova N, Secundino NF, et al. *Leishmania infantum*: Lipophosphoglycan intraspecific variation and interaction with vertebrate and invertebrate hosts. Int J Parasitol. 2011;41: 333–342. 21118695 10.1016/j.ijpara.2010.10.004

[39] Rugani JN, Quaresma PF, Gontijo CF, Soares RP, Monte-Neto RL. Intraspecies susceptibility of *Leishmania* (*Viannia*) *braziliensis* to antileishmanial drugs: antimony resistance in human isolates from atypical lesions. Biomedicine & Pharmacotherapy. 2018;108: 1170–1180. 30372818 10.1016/j.biopha.2018.09.149

[40] Rêgo FD, Cardoso C d A, Moreira POL, Nogueira PM, Araújo MS, Borges VM, et al. *Leishmania amazonensis* from distinct clinical forms/hosts has polymorphisms in Lipophosphoglycans, displays variations in immunomodulatory properties and, susceptibility to antileishmanial drugs. Cell Biol Int. 2022;46: 1947–1958. 35998255 10.1002/cbin.11880 PMC9804363

[41] Thomaz-Soccol V, Goncalves AL, Piechnik CA, Baggio RA, Boeger WA, Buchman TL, et al. Hidden danger: Unexpected scenario in the vector-parasite dynamics of leishmaniases in the Brazil side of triple border (Argentina, Brazil and Paraguay). PLoS Negl Trop Dis. 2018;12: e0006336. doi: 10.1371/journal.pntd.0006336 29624586 PMC5906028

[42] Diniz MMC de SL, Galvis Ovallos F, de Castro Gomes CM, de Oliveira Lavitschka C, Bianchi Galati EA. Host-biting rate and susceptibility of some suspected vectors to *Leishmania braziliensis*. Parasit Vectors. 2014;7: 1–11. 24684943 10.1186/1756-3305-7-139 PMC3976554

[43] Pinheiro LJ, Paranaíba LF, Alves AF, Parreiras PM, Gontijo NF, Soares RP, et al. Salivary gland extract modulates the infection of two *Leishmania enriettii* strains by interfering with macrophage differentiation in the model of Cavia porcellus. Front Microbiol. 2018;9: 350959. 10.3389/fmicb.2018.00969 PMC5986888 29896161

[44] Ridley DS, Ridley MJ. The evolution of the lesion in cutaneous leishmaniasis. J Pathol. 1983;141: 83–96. doi: 10.1002/path.1711410109 6620010

[45] Flores GVA, Pacheco CMS, Ferreira AF, Tomokane TY, Nunes JB, Colombo FA, et al. *Leishmania* (*L*.) *infantum chagasi* isolated from skin lesions of patients affected by non-ulcerated cutaneous leishmaniasis lead to visceral lesion in hamsters. Parasitol Int. 2022;93: 9. 10.1016/j.parint.2022.102723 36566911

[46] Macedo DH, Menezes-Neto A, Rugani JM, Rocha AC, Silva SO, Melo MN, et al. Low frequency of LRV1 in *Leishmania braziliensis* strains isolated from typical and atypical lesions in the State of Minas Gerais, Brazil. Mol Biochem Parasitol. 2016;210: 50–54. 27546549 10.1016/j.molbiopara.2016.08.005 PMC5125831

[47] Rêgo FD, da Silva ES, Lopes VV, Teixeira-Neto RG, Belo VS, Fonseca Júnior AA, et al. First report of putative Leishmania RNA virus 2 (LRV2) in *Leishmania infantum* strains from canine and human visceral leishmaniasis cases in the southeast of Brazil. Mem Inst Oswaldo Cruz. 2023;118: e230071. 37729273 10.1590/0074-02760230071 PMC10511063

[48] Shapiro SS, Wilk MB. An analysis of variance test for normality (complete samples). Biometrika. 1965;52: 591–611.

[49] Ogg MM, Carrion R, de Carvalho Botelho AC, Mayrink W, Correa-Oliveira R, Patterson JL. Quantification of leishmaniavirus RNA in clinical samples and its possible role in pathogenesis. American Journal of Tropical Medicine and Hygiene. 2003;69: 309–313. 14628949

[50] Pereira L de OR, Maretti-Mira AC, Rodrigues KM, Lima RB, Oliveira-Neto MP de, Cupolillo E, et al. Severity of tegumentary leishmaniasis is not exclusively associated with Leishmania RNA virus 1 infection in Brazil. Mem Inst Oswaldo Cruz. 2013;108: 665–667. doi: 10.1590/0074-0276108052013021 23903986 PMC3970596

[51] Zabala-Peñafiel A, Fantinatti M, Dias-Lopes G, da Silva JL, Miranda L de FC, Lyra MR, et al. First report of Leishmania RNA virus 1 in *Leishmania* (*Viannia*) *braziliensis* clinical isolates from Rio de Janeiro State-Brazil. Mem Inst Oswaldo Cruz. 2022;117: e210107. 36000673 10.1590/0074-02760210107 PMC9395166

[52] Pita-Pereira D, Souza GD, Zwetsch A, Alves CR, Britto C, Rangel EF. First report of *Lutzomyia* (*Nyssomyia*) *neivai* (Diptera: Psychodidae: Phlebotominae) naturally infected by *Leishmania* (*Viannia*) *braziliensis* in a periurban area of south Brazil using a multiplex polymerase chain reaction assay. American Journal of Tropical Medicine and Hygiene. 2009;80: 593. 19346382

[53] Ribeiro JMC, Francischetti IMB. Role of arthropod saliva in blood feeding: sialome and post-sialome perspectives. Annu Rev Entomol. 2003;48: 73–88. doi: 10.1146/annurev.ento.48.060402.102812 12194906

[54] Espinosa OA, Serrano MG, Camargo EP, Teixeira MMG, Shaw JJ. An appraisal of the taxonomy and nomenclature of trypanosomatids presently classified as *Leishmania* and *Endotrypanum*. Parasitology. 2018;145: 430–442. 27976601 10.1017/S0031182016002092

[55] Alves-Sobrinho EV, Pinheiro L de J, Paranaíba LF, Fontes IC, Parreiras PM, Gontijo NF, et al. *Leishmania enriettii* visceralises in the trachea, lungs, and spleen of Cavia porcellus. Mem Inst Oswaldo Cruz. 2022;117: e220065. 35920504 10.1590/0074-02760220065 PMC9343011

[56] Valdivia HO, Almeida L V, Roatt BM, Reis-Cunha JL, Pereira AAS, Gontijo C, et al. Comparative genomics of canine-isolated *Leishmania* (*Leishmania*) *amazonensis* from an endemic focus of visceral leishmaniasis in Governador Valadares, southeastern Brazil. Sci Rep. 2017;7: 40804. 28091623 10.1038/srep40804 PMC5238499

[57] Duarte MIS, Laurenti MD, Andrade HF Jr, Corbett CEP. Estudo comparativo do comportamento biológico de dois isolados de *Leishmania* caracterizados respectivamente como *L*. *major*-like e *L*. *donovani*, em hamster. Rev Inst Med Trop Sao Paulo. 1988;30: 21–27.3222614

[58] Corbett CE, Paes RA, Laurenti MD, Júnior HFA, Duarte MI. Histopathology of lymphoid organs in experimental leishmaniasis. Int J Exp Pathol. 1992;73: 417. 1390190 PMC2002360

[59] de Assis RR, Ibraim IC, Nogueira PM, Soares RP, Turco SJ. Glycoconjugates in New World species of *Leishmania*: polymorphisms in lipophosphoglycan and glycoinositolphospholipids and interaction with hosts. Biochimica et Biophysica Acta (BBA)-General Subjects. 2012;1820: 1354–1365. 22093608 10.1016/j.bbagen.2011.11.001

[60] Kamhawi S, Ramalho-Ortigao M, Pham VM, Kumar S, Lawyer PG, Turco SJ, et al. A role for insect galectins in parasite survival. Cell. 2004;119: 329–341. doi: 10.1016/j.cell.2004.10.009 15543683

[61] Rogers ME, Ilg T, Nikolaev A V, Ferguson MAJ, Bates PA. Transmission of cutaneous leishmaniasis by sand flies is enhanced by regurgitation of fPPG. Nature. 2004;430: 463–467. doi: 10.1038/nature02675 15269771 PMC2835460

[62] Assis RR, Ibraim IC, Noronha FS, Turco SJ, Soares RP. Glycoinositolphospholipids from *Leishmania braziliensis* and *L*. *infantum*: modulation of innate immune system and variations in carbohydrate structure. PLoS Negl Trop Dis. 2012;6: e1543. 22389743 10.1371/journal.pntd.0001543 PMC3289616

[63] Ibraim IC, de Assis RR, Pessoa NL, Campos MA, Melo MN, Turco SJ, et al. Two biochemically distinct lipophosphoglycans from *Leishmania braziliensis* and *Leishmania infantum* trigger different innate immune responses in murine macrophages. Parasit Vectors. 2013;6: 1–11. 23497381 10.1186/1756-3305-6-54 PMC3606350

[64] Nogueira PM, Assis RR, Torrecilhas AC, Saraiva EM, Pessoa NL, Campos MA, et al. Lipophosphoglycans from *Leishmania amazonensis* strains display immunomodulatory properties via TLR4 and do not affect sand fly infection. PLoS Negl Trop Dis. 2016;10: e0004848. 27508930 10.1371/journal.pntd.0004848 PMC4980043

[65] Cardoso CA, Araujo G V, Sandoval CM, Nogueira PM, Zúniga C, Sosa-Ochoa WH, et al. Lipophosphoglycans from dermotropic *Leishmania infantum* are more pro-inflammatory than those from viscerotropic strains. Mem Inst Oswaldo Cruz. 2020;115: e200140. 32965329 10.1590/0074-02760200140 PMC7521155

[66] Oliveira A-C, Peixoto JR, de Arruda LB, Campos MA, Gazzinelli RT, Golenbock DT, et al. Expression of functional TLR4 confers proinflammatory responsiveness to *Trypanosoma cruzi* glycoinositolphospholipids and higher resistance to infection with T. cruzi. The Journal of Immunology. 2004;173: 5688–5696. 15494520 10.4049/jimmunol.173.9.5688

[67] Gazzinelli RT, Ropert C, Campos MA. Role of the Toll/interleukin-1 receptor signaling pathway in host resistance and pathogenesis during infection with protozoan parasites. Immunol Rev. 2004;201: 9–25. doi: 10.1111/j.0105-2896.2004.00174.x 15361229

[68] Cantanhêde LM, Fernandes FG, Ferreira GEM, Porrozzi R, Ferreira R de GM, Cupolillo E. New insights into the genetic diversity of Leishmania RNA Virus 1 and its species-specific relationship with Leishmania parasites. PLoS One. 2018;13: e0198727. doi: 10.1371/journal.pone.0198727 29912912 PMC6005476

[69] Soares RPP, Cardoso TL, Barron T, Araújo MSS, Pimenta PFP, Turco SJ. *Leishmania braziliensis*: a novel mechanism in the lipophosphoglycan regulation during metacyclogenesis. Int J Parasitol. 2005;35: 245–253. 15722076 10.1016/j.ijpara.2004.12.008

[70] Ilg T, Etges R, Overath P, McConville MJ, Thomas-Oates J, Thomas J, et al. Structure of *Leishmania mexicana* lipophosphoglycan. Journal of Biological Chemistry. 1992;267: 6834–6840. 1551890

[71] McConville MJ, Blackwell JM. Developmental changes in the glycosylated phosphatidylinositols of *Leishmania donovani*. Characterization of the promastigote and amastigote glycolipids. Journal of Biological Chemistry. 1991;266: 15170–15179. 1831200

[72] Lima JB, Araújo-Santos T, Lázaro-Souza M, Carneiro AB, Ibraim IC, Jesus-Santos FH, et al. *Leishmania infantum* lipophosphoglycan induced-Prostaglandin E2 production in association with PPAR-γ expression via activation of Toll like receptors-1 and 2. Sci Rep. 2017;7: 14321. 29084985 10.1038/s41598-017-14229-8 PMC5662570

[73] Eresh S, de Bruijn MHL, Mendoza-León JA, Barker DC. *Leishmania* (*Viannia*) *lainsoni* occupies a unique niche within the subgenus *Viannia*. Trans R Soc Trop Med Hyg. 1995;89: 231–236. 7778160 10.1016/0035-9203(95)90509-x

