# Supplementary figures and images for: Unveiling the Enigmatic nature of six neglected Amazonian Leishmania (Viannia) species using the hamster model: Virulence, Histopathology and prospection of LRV1
Source: PLoS Negl Trop Dis. 2024 Aug 9;18(8):e0012333. doi: 10.1371/journal.pntd.0012333 (PMC11315283; doi:10.1371/journal.pntd.0012333)

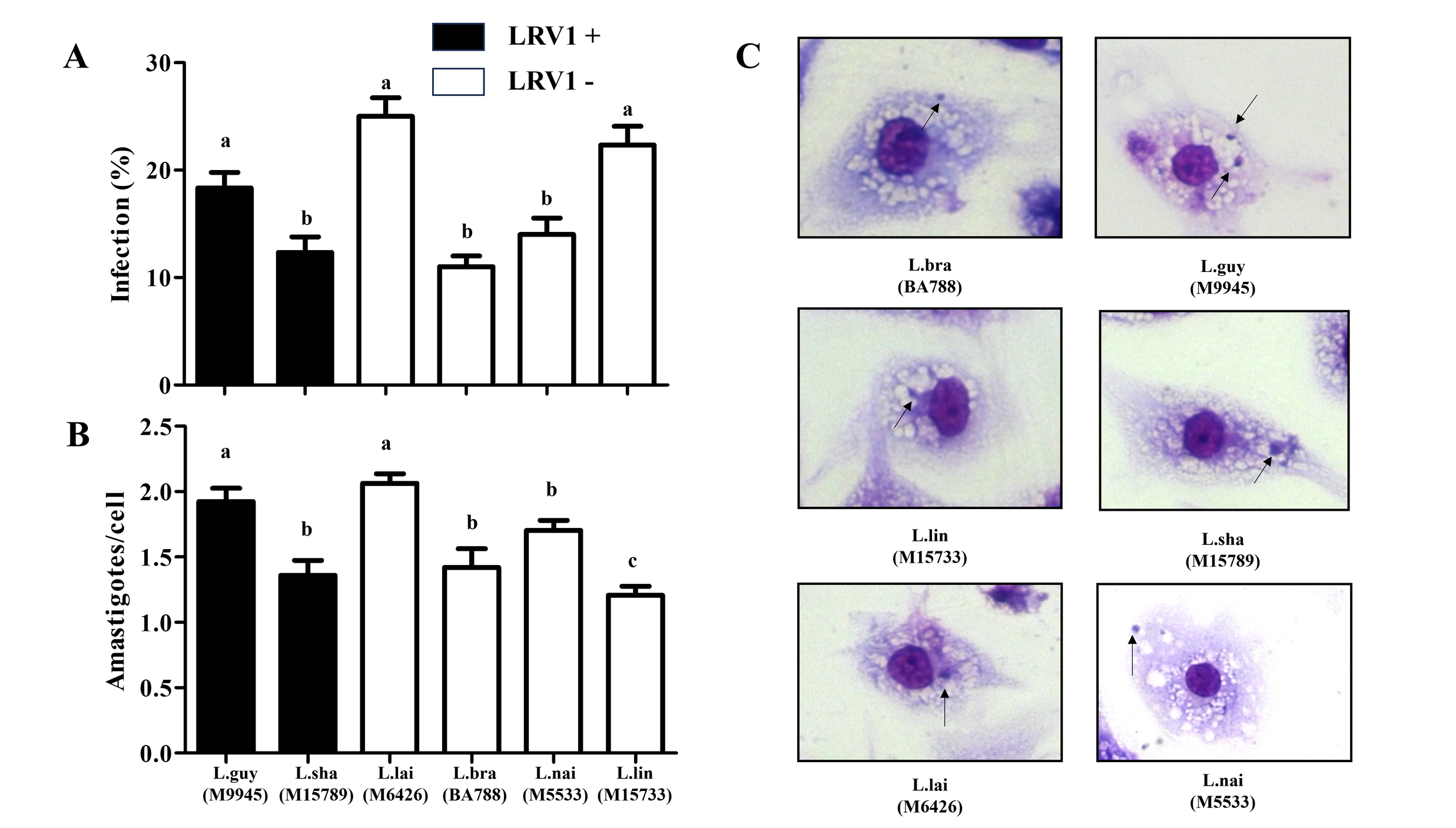

Supplement: S1 Fig — (A) Macrophage infection (%), (B) number of amastigotes per macrophage and (C) Intracellular amastigote forms in THP-1 macrophages. Legend: L.braz, L. braziliensis; L.guy, L. guyanensis; L.sha, L. shawi, L.lind, L. linderbergi, L.lai, L. lainsoni and L.nai, L. naiffi. Magnification of 1000x. Dark and white bars indicate LRV1 presence and absence, respectively. Letters above bars indicate statistical differences (P<0.05). (TIF) [file pntd.0012333.s001.tif]

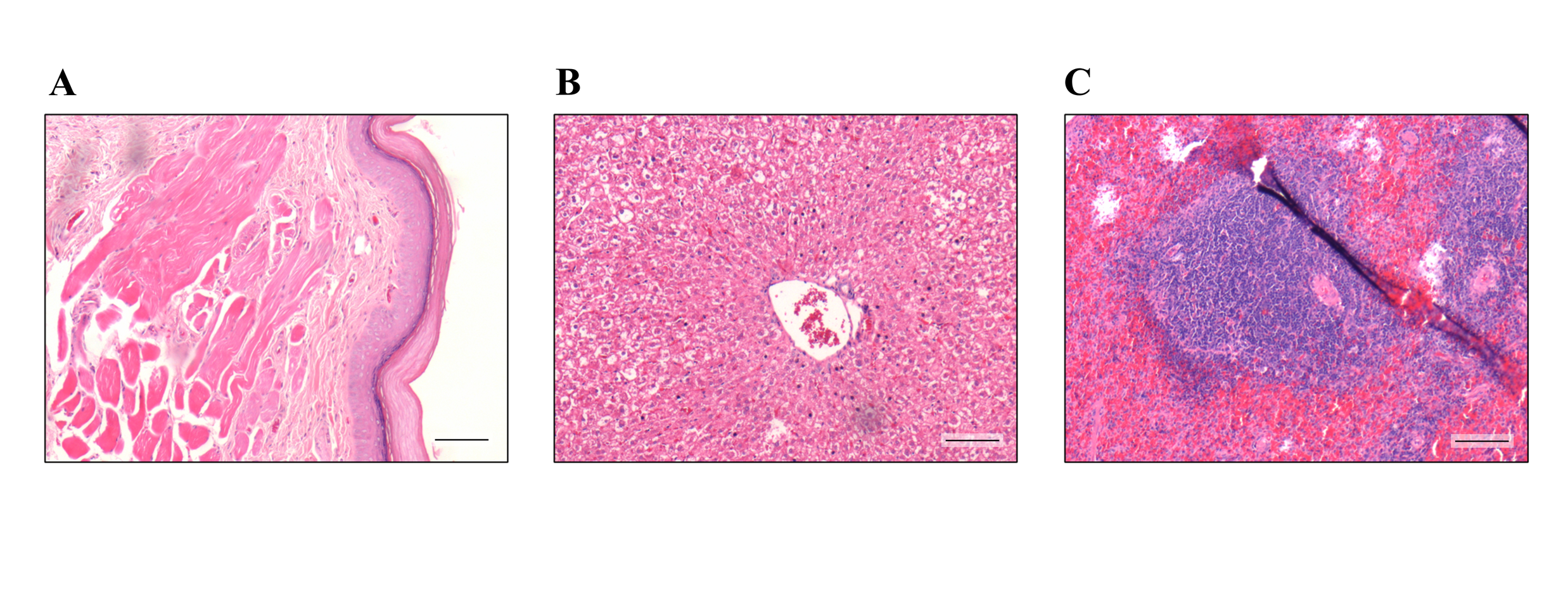

Supplement: S2 Fig — Representative negative controls of skin (A), liver (B) and spleen (C) of uninfected M. auratus stained with hematoxylin-eosin (HE). Scale bar = 20 μm. (TIF) [file pntd.0012333.s002.tif]
