# Supplementary material for: Unveiling the Enigmatic nature of six neglected Amazonian Leishmania (Viannia) species using the hamster model: Virulence, Histopathology and prospection of LRV1
Source: PLoS Negl Trop Dis. 2024 Aug 9;18(8):e0012333. doi: 10.1371/journal.pntd.0012333 (PMC11315283; doi:10.1371/journal.pntd.0012333)
Supplement: S1 File — (DOCX) [file pntd.0012333.s003.docx]

**Raw data and statistical analysis of Figure 2:**

**Number of amastigotes/cell**

| **L.guy (M9945)** | **L.sha (M15789)** | **L.lai (M6426)** | **L.braz (BA788)** | **L.nai (M5533)** | **L.lin (M15733)** |
| --- | --- | --- | --- | --- | --- |
| 9,98 | 5,65 | 5,49 | 3,45 | 1,8 | 1,5 |
| 9,58 | 5,73 | 5,23 | 3,28 | 1,75 | 1,75 |
| 10,72 | 5,46 | 5,16 | 3,45 | 1,2 | 1,5 |
| 9,72 | 2,4 | 5,28 | 3,54 | 2,4 | 1,8 |

**Statistical analysis**

| **Number of values** | **L.guy (M9945)** | **L.sha (M15789)** | **L.lai (M6426)** | **L.braz (BA788)** | **L.nai (M5533)** | **L.lin (M15733)** |
| --- | --- | --- | --- | --- | --- | --- |
|  |  |  |  |  |  |  |
| **Minimum** | 9,580 | 2,400 | 5,160 | 3,280 | 1,200 | 1,500 |
| **25% Percentile** | 9,615 | 3,165 | 5,178 | 3,323 | 1,338 | 1,500 |
| **Median** | 9,850 | 5,555 | 5,255 | 3,450 | 1,775 | 1,625 |
| **75% Percentile** | 10,54 | 5,710 | 5,438 | 3,518 | 2,250 | 1,788 |
| **Maximum** | 10,72 | 5,730 | 5,490 | 3,540 | 2,400 | 1,800 |
|  |  |  |  |  |  |  |
| **Mean** | 10,00 | 4,810 | 5,290 | 3,430 | 1,788 | 1,638 |
| **Std. Deviation** | 0,5078 | 1,611 | 0,1421 | 0,1086 | 0,4905 | 0,1601 |
| **Std. Error** | 0,2539 | 0,8053 | 0,07106 | 0,05431 | 0,2453 | 0,08004 |
|  |  |  |  |  |  |  |
| **Lower 95% CI of mean** | 9,192 | 2,247 | 5,064 | 3,257 | 1,007 | 1,383 |
| **Upper 95% CI of mean** | 10,81 | 7,373 | 5,516 | 3,603 | 2,568 | 1,892 |
|  |  |  |  |  |  |  |
| **Sum** | 40,00 | 19,24 | 21,16 | 13,72 | 7,150 | 6,550 |

**Percentage of infected macrophages (%)**

| **L.guy (M9945)** | **L.sha (M15789)** | **L.lai (M6426)** | **L.braz (BA788)** | **L.nai (M5533)** | **L.lin (M15733)** |
| --- | --- | --- | --- | --- | --- |
| 66 | 54 | 39 | 40 | 5 | 4 |
| 60 | 51 | 40 | 43 | 4 | 4 |
| 58 | 57 | 43 | 42 | 5 | 4 |
| 69 | 47 | 42 | 42 | 5 | 5 |

**Statistical analysis**

| **Number of values** | **L.guy (M9945)** | **L.sha (M15789)** | **L.lai (M6426)** | **L.braz (BA788)** | **L.nai (M5533)** | **L.lin (M15733)** |
| --- | --- | --- | --- | --- | --- | --- |
|  |  |  |  |  |  |  |
| **Minimum** | 58,00 | 47,00 | 39,00 | 40,00 | 4,000 | 4,000 |
| **25% Percentile** | 58,50 | 48,00 | 39,25 | 40,50 | 4,250 | 4,000 |
| **Median** | 63,00 | 52,50 | 41,00 | 42,00 | 5,000 | 4,000 |
| **75% Percentile** | 68,25 | 56,25 | 42,75 | 42,75 | 5,000 | 4,750 |
| **Maximum** | 69,00 | 57,00 | 43,00 | 43,00 | 5,000 | 5,000 |
|  |  |  |  |  |  |  |
| **Mean** | 63,25 | 52,25 | 41,00 | 41,75 | 4,750 | 4,250 |
| **Std. Deviation** | 5,123 | 4,272 | 1,826 | 1,258 | 0,5000 | 0,5000 |
| **Std. Error** | 2,562 | 2,136 | 0,9129 | 0,6292 | 0,2500 | 0,2500 |
|  |  |  |  |  |  |  |
| **Lower 95% CI of mean** | 55,10 | 45,45 | 38,09 | 39,75 | 3,954 | 3,454 |
| **Upper 95% CI of mean** | 71,40 | 59,05 | 43,91 | 43,75 | 5,546 | 5,046 |
|  |  |  |  |  |  |  |
| **Sum** | 253,0 | 209,0 | 164,0 | 167,0 | 19,00 | 17,00 |

**THP1- Percentage of infected macrophages**

| **L.guy (M9945)** | **L.sha (M15789)** | **L.lai (M6426)** | **L.braz (BA788)** | **L.nai (M5533)** | **L.lin (M15733)** |
| --- | --- | --- | --- | --- | --- |
| 16 | 12 | 25 | 9 | 15 | 19 |
| 21 | 10 | 28 | 12 | 11 | 23 |
| 18 | 15 | 22 | 12 | 16 | 25 |

**Statistical analysis**

| **Number of values** | **L.guy (M9945)** | **L.sha (M15789)** | **L.lai (M6426)** | **L.braz (BA788)** | **L.nai (M5533)** | **L.lin (M15733)** |
| --- | --- | --- | --- | --- | --- | --- |
|  |  |  |  |  |  |  |
| **Minimum** | 16,00 | 10,00 | 22,00 | 9,000 | 11,00 | 19,00 |
| **25% Percentile** | 16,00 | 10,00 | 22,00 | 9,000 | 11,00 | 19,00 |
| **Median** | 18,00 | 12,00 | 25,00 | 12,00 | 15,00 | 23,00 |
| **75% Percentile** | 21,00 | 15,00 | 28,00 | 12,00 | 16,00 | 25,00 |
| **Maximum** | 21,00 | 15,00 | 28,00 | 12,00 | 16,00 | 25,00 |
|  |  |  |  |  |  |  |
| **Mean** | 18,33 | 12,33 | 25,00 | 11,00 | 14,00 | 22,33 |
| **Std. Deviation** | 2,517 | 2,517 | 3,000 | 1,732 | 2,646 | 3,055 |
| **Std. Error** | 1,453 | 1,453 | 1,732 | 1,000 | 1,528 | 1,764 |
|  |  |  |  |  |  |  |
| **Lower 95% CI of mean** | 12,08 | 6,082 | 17,55 | 6,697 | 7,428 | 14,74 |
| **Upper 95% CI of mean** | 24,58 | 18,58 | 32,45 | 15,30 | 20,57 | 29,92 |
|  |  |  |  |  |  |  |
| **Sum** | 55,00 | 37,00 | 75,00 | 33,00 | 42,00 | 67,00 |

**THP-1 Number of amastigote/cell**

| **L.guy (M9945)** | **L.sha (M15789)** | **L.lai (M6426)** | **L.braz (BA788)** | **L.nai (M5533)** | **L.lin (M15733)** |
| --- | --- | --- | --- | --- | --- |
| 2,13 | 1,58 | 2,2 | 1,67 | 1,73 | 1,32 |
| 1,81 | 1,3 | 2,04 | 1,17 | 1,82 | 1,22 |
| 1,83 | 1,2 | 1,95 | 1,42 | 1,56 | 1,08 |

**Statistical analysis**

| **Number of values** | **L.guy (M9945)** | **L.sha (M15789)** | **L.lai (M6426)** | **L.braz (BA788)** | **L.nai (M5533)** | **L.lin (M15733)** |
| --- | --- | --- | --- | --- | --- | --- |
|  |  |  |  |  |  |  |
| **Minimum** | 1,810 | 1,200 | 1,950 | 1,170 | 1,560 | 1,080 |
| **25% Percentile** | 1,810 | 1,200 | 1,950 | 1,170 | 1,560 | 1,080 |
| **Median** | 1,830 | 1,300 | 2,040 | 1,420 | 1,730 | 1,220 |
| **75% Percentile** | 2,130 | 1,580 | 2,200 | 1,670 | 1,820 | 1,320 |
| **Maximum** | 2,130 | 1,580 | 2,200 | 1,670 | 1,820 | 1,320 |
|  |  |  |  |  |  |  |
| **Mean** | 1,923 | 1,360 | 2,063 | 1,420 | 1,703 | 1,207 |
| **Std. Deviation** | 0,1793 | 0,1970 | 0,1266 | 0,2500 | 0,1320 | 0,1206 |
| **Std. Error** | 0,1035 | 0,1137 | 0,07311 | 0,1443 | 0,07623 | 0,06960 |
|  |  |  |  |  |  |  |
| **Lower 95% CI of mean** | 1,478 | 0,8707 | 1,749 | 0,7990 | 1,375 | 0,9072 |
| **Upper 95% CI of mean** | 2,369 | 1,849 | 2,378 | 2,041 | 2,031 | 1,506 |
|  |  |  |  |  |  |  |
| **Sum** | 5,770 | 4,080 | 6,190 | 4,260 | 5,110 | 3,620 |

**Raw data and statistical analysis of Figure 3:**

**Lesion size**

| **10** | **20** | **30** | **40** |
| --- | --- | --- | --- |
| 0,48 | 0,08 | 0,54 | 0,92 |
| 0,44 | 0,05 | 0,72 | 0,39 |
| 0,28 | 0,11 | 0,09 | 0,73 |
| 0,01 | 0,14 | 0,85 | 1 |
| 0,11 | 0,11 | 0,55 | 0,95 |
| 0,2 | 0,17 | 0,73 | 0,4 |
| 0,11 | 0,06 | 0,11 | 0,74 |
| 0,59 | 0,04 | 0,86 | 1,01 |

**Statistical analysis**

| **Number of values** | **10** | **20** | **30** | **40** |
| --- | --- | --- | --- | --- |
|  |  |  |  |  |
| **Minimum** | -0,0100 | -0,0600 | 0,0900 | 0,3900 |
| **25% Percentile** | 0,1100 | -0,0275 | 0,2175 | 0,4825 |
| **Median** | 0,2400 | 0,0950 | 0,6350 | 0,8300 |
| **75% Percentile** | 0,4700 | 0,1325 | 0,8200 | 0,9875 |
| **Maximum** | 0,5900 | 0,1700 | 0,8600 | 1,010 |
|  |  |  |  |  |
| **Mean** | 0,2750 | 0,0675 | 0,5563 | 0,7675 |
| **Std. Deviation** | 0,2104 | 0,08481 | 0,3053 | 0,2533 |
| **Std. Error** | 0,07438 | 0,02999 | 0,1079 | 0,08956 |
|  |  |  |  |  |
| **Lower 95% CI of mean** | 0,09912 | -0,003404 | 0,3010 | 0,5557 |
| **Upper 95% CI of mean** | 0,4509 | 0,1384 | 0,8115 | 0,9793 |
|  |  |  |  |  |
| **Sum** | 2,200 | 0,5400 | 4,450 | 6,140 |

**Raw data and statistical analysis of Figure 7:**

**Clinical score (20 days)**

| **L.guy (M9945)** | | | | |
| --- | --- | --- | --- | --- |
| 3 | 0 | 3 | 2 | |
| **L.braz (BA788)** | | | |  |
| 4 | 0 | 0 | 0 |  |
| **L.sha (M15789)** | | | |  |
| 1 | 1 | 1 | 0 |  |
| **L.lai (M6426)** | | | |  |
| 5 | 5 | 4 | 7 |  |
| **L.nai (M5533)** | | | |  |
| 0 | 0 | 0 | 0 |  |
| **L.lind (M15733)** | | | |  |
| 1 | 1 | 1 | 0 |  |

**Statistical analysis**

| **Number of values** | **L.guy (M9945)** | **L.sha (M15789)** | **L.lai (M6426)** | **L.braz (BA788)** | **L.nai (M5533)** | **L.lin (M15733)** |
| --- | --- | --- | --- | --- | --- | --- |
|  |  |  |  |  |  |  |
| **Minimum** | 0,0 | 0,0 | 0,0 | 4,000 | 0,0 | 0,0 |
| **25% Percentile** | 0,5000 | 0,0 | 0,2500 | 4,250 | 0,0 | 0,2500 |
| **Median** | 2,500 | 0,0 | 1,000 | 5,000 | 0,0 | 1,000 |
| **75% Percentile** | 3,000 | 3,000 | 1,000 | 6,500 | 0,0 | 1,000 |
| **Maximum** | 3,000 | 4,000 | 1,000 | 7,000 | 0,0 | 1,000 |
|  |  |  |  |  |  |  |
| **Mean** | 2,000 | 1,000 | 0,7500 | 5,250 | 0,0 | 0,7500 |
| **Std. Deviation** | 1,414 | 2,000 | 0,5000 | 1,258 | 0,0 | 0,5000 |
| **Std. Error** | 0,7071 | 1,000 | 0,2500 | 0,6292 | 0,0 | 0,2500 |
|  |  |  |  |  |  |  |
| **Lower 95% CI of mean** | -0,2503 | -2,182 | -0,04562 | 3,248 | 0,0 | -0,04562 |
| **Upper 95% CI of mean** | 4,250 | 4,182 | 1,546 | 7,252 | 0,0 | 1,546 |
|  |  |  |  |  |  |  |
| **Sum** | 8,000 | 4,000 | 3,000 | 21,00 | 0,0 | 3,000 |

**Clinical score (40 days)**

| **L.guy (M9945)** | | | | |
| --- | --- | --- | --- | --- |
| 17 | 15 | 14 | 18 | |
| **L.bra (BA788)** | | | |  |
| 17 | 15 | 15 | 17 |  |
| **L.sha (M15789)** | | | |  |
| 15 | 9 | 10 | 8 |  |
| **L.lai (M6426)** | | | |  |
| 14 | 14 | 14 | 8 |  |
| **L.nai (M5533)** | | | |  |
| 0 | 0 | 0 | 0 |  |
| **L.lind (M15733)** | | | |  |
| 0 | 0 | 5 | 0 |  |

**Statistical analysis**

| **Number of values** | **L.guy (M9945)** | **L.sha (M15789)** | **L.lai (M6426)** | **L.braz (BA788)** | **L.nai (M5533)** | **L.lin (M15733)** |
| --- | --- | --- | --- | --- | --- | --- |
|  |  |  |  |  |  |  |
| **Minimum** | 14,00 | 15,00 | 8,000 | 8,000 | 0,0 | 0,0 |
| **25% Percentile** | 14,25 | 15,00 | 8,250 | 9,500 | 0,0 | 0,0 |
| **Median** | 16,00 | 16,00 | 9,500 | 14,00 | 0,0 | 0,0 |
| **75% Percentile** | 17,75 | 17,00 | 13,75 | 14,00 | 0,0 | 3,750 |
| **Maximum** | 18,00 | 17,00 | 15,00 | 14,00 | 0,0 | 5,000 |
|  |  |  |  |  |  |  |
| **Mean** | 16,00 | 16,00 | 10,50 | 12,50 | 0,0 | 1,250 |
| **Std. Deviation** | 1,826 | 1,155 | 3,109 | 3,000 | 0,0 | 2,500 |
| **Std. Error** | 0,9129 | 0,5774 | 1,555 | 1,500 | 0,0 | 1,250 |
|  |  |  |  |  |  |  |
| **Lower 95% CI of mean** | 13,09 | 14,16 | 5,553 | 7,726 | 0,0 | -2,728 |
| **Upper 95% CI of mean** | 18,91 | 17,84 | 15,45 | 17,27 | 0,0 | 5,228 |
|  |  |  |  |  |  |  |
| **Sum** | 64,00 | 64,00 | 42,00 | 50,00 | 0,0 | 5,000 |

**Raw data and statistical analysis of Figure 11:**

**Parasite load (dermis)**

**
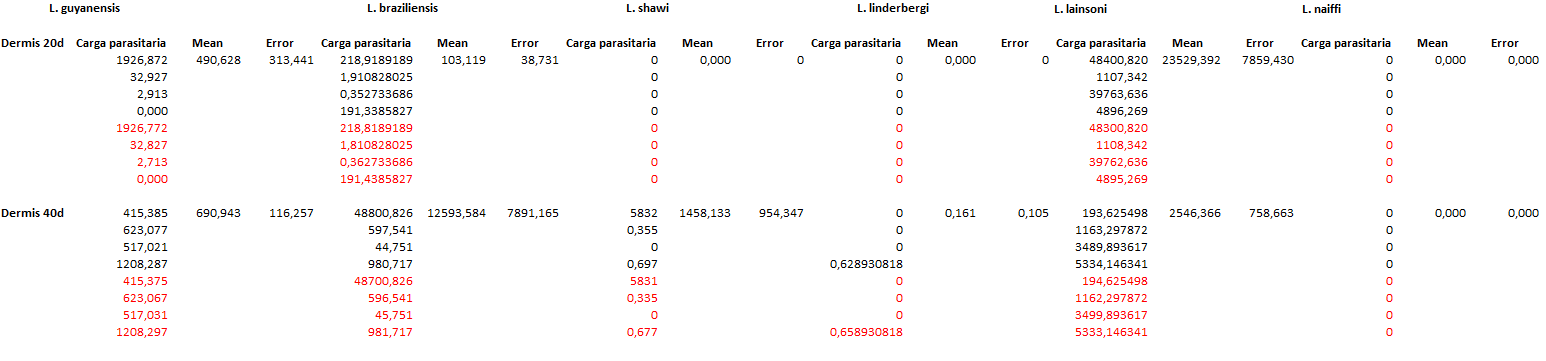
**

**Statistical analysis**

**
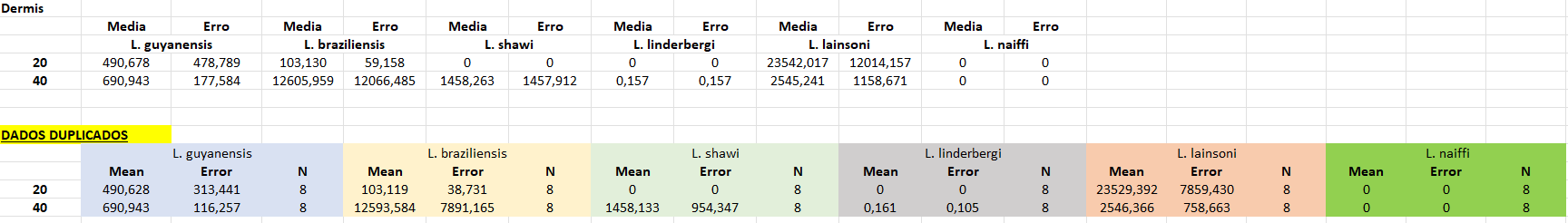
**

**Parasite load (lymph node)**

**
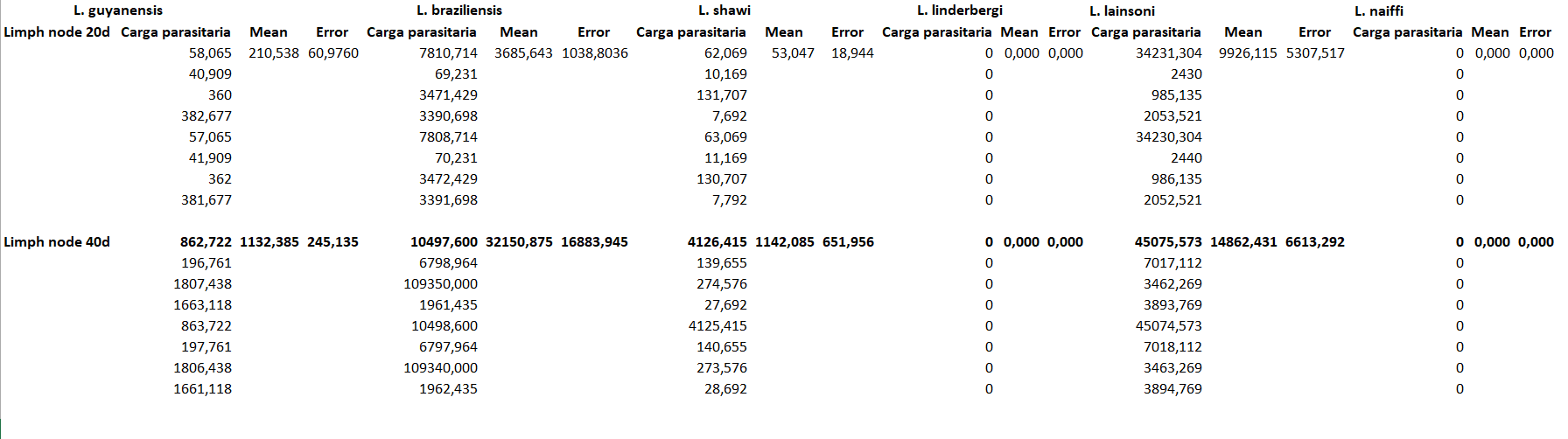
**

**Statistical analysis**

**
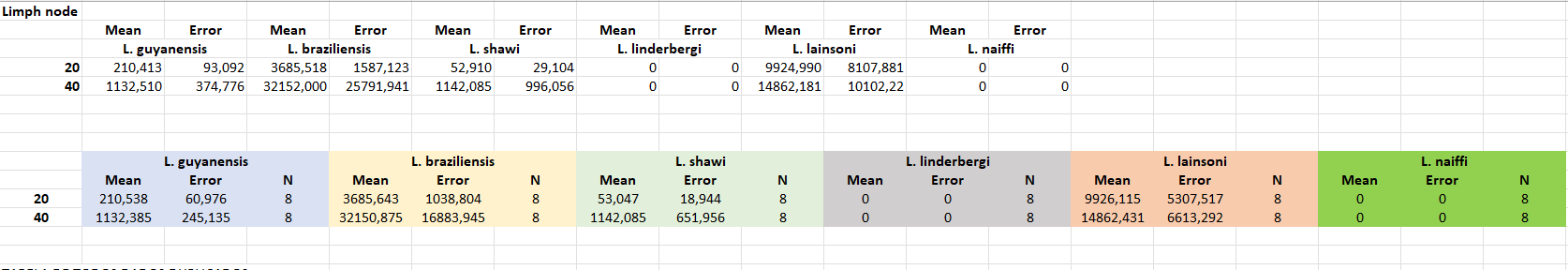
**

**Raw data and statistical analysis of Figure 12:**

**Nitric oxide (µM)**

**LPG (WT, TLR2KO and TLR4KO)**

| **Control** | **LPS** | **S.a** | **L.b** | **L.g** | **L.s** | **L.la** | **L.li** | **L.n** |
| --- | --- | --- | --- | --- | --- | --- | --- | --- |
| 1,996316 | 48,6982 | 46,92389 | 19,71787 | 23,0084 | 33,65423 | 32,52513 | 47,46156 | 28,09474 |
| 2,136553 | 48,26806 | 50,53702 | 19,9652 | 26,60002 | 48,05299 | 33,98759 | 37,11682 | 24,70743 |
| 7,534301 | 49,90257 | 53,38667 | 17,28761 | 17,88979 | 33,36389 | 32,12726 | 44,8915 | 19,70711 |
| 2,340423 | 56,59117 | 51,30051 | 17,44891 | 23,59983 | 34,8371 | 25,58921 | 33,43917 | 23,78264 |
|  |  |  | 15,86816 | 20,54588 | 36,94476 | 27,3205 | 34,92313 | 21,61046 |
| **Control** | **LPS** | **S.a** | **L.b** | **L.g** | **L.s** | **L.la** | **L.li** | **L.n** |
| 2,211826 | 58,35472 | 17,34264 | 36,7512 | 43,13871 | 40,45036 | 56,12877 | 34,04136 | 34,3532 |
| 1,125293 | 63,04319 | 14,15964 | 35,65436 | 36,18128 | 37,0523 | 57,13959 | 55,19323 | 42,98816 |
| 1,4694 | 61,87107 | 14,10588 | 28,14851 | 44,68719 | 30,1809 | 57,41918 | 32,77246 | 38,17065 |
| 2,222136 | 63,37655 | 13,2241 | 32,06274 | 38,08462 | 34,42848 | 50,04237 | 27,9657 | 27,99796 |
|  |  |  | 41,54721 | 39,00941 | 30,61103 | 50,7736 | 27,54632 | 27,90118 |
| **Control** | **LPS** | **S.a** | **L.b** | **L.g** | **L.s** | **L.la** | **L.li** | **L.n** |
| 0,856458 | 14,54613 | 48,22505 | 19,71787 | 23,0084 | 33,65423 | 32,52513 | 47,46156 | 28,09474 |
| 2,598504 | 19,67485 | 51,0962 | 19,9652 | 26,60002 | 48,05299 | 33,98759 | 37,11682 | 24,70743 |
| 1,501661 | 13,17033 | 44,16028 | 17,28761 | 17,88979 | 33,36389 | 32,12726 | 44,8915 | 19,70711 |
| 2,501724 | 16,6968 | 48,02073 | 17,44891 | 23,59983 | 34,8371 | 25,58921 | 33,43917 | 23,78264 |
|  |  |  | 15,86816 | 20,54588 | 36,94476 | 27,3205 | 34,92313 | 21,61046 |

**Statistical analysis**

| **Tukey's multiple comparisons test** | **Mean Diff,** | **95,00% CI of diff,** | **Significant?** | **Summary** | **Adjusted P Value** |  |
| --- | --- | --- | --- | --- | --- | --- |
| **Control vs. LPS** | -47,36 | -59,59 to -35,13 | Yes | **** | <0,0001 | A-B |
| **Control vs. S.a** | -47,04 | -59,27 to -34,80 | Yes | **** | <0,0001 | A-C |
| **Control vs. L.b** | -14,56 | -26,16 to -2,953 | Yes | ** | 0,0018 | A-D |
| **Control vs. L.g** | -18,83 | -30,43 to -7,224 | Yes | **** | <0,0001 | A-E |
| **Control vs. L.s** | -33,87 | -45,47 to -22,27 | Yes | **** | <0,0001 | A-F |
| **Control vs. L.la** | -26,81 | -38,41 to -15,21 | Yes | **** | <0,0001 | A-G |
| **Control vs. L.li** | -36,06 | -47,67 to -24,46 | Yes | **** | <0,0001 | A-H |
| **Control vs. L.n** | -20,08 | -31,68 to -8,476 | Yes | **** | <0,0001 | A-I |
| **Control vs. Control** | 1,745 | -10,49 to 13,97 | No | ns | >0,9999 | A-J |
| **Control vs. LPS** | -58,16 | -70,39 to -45,93 | Yes | **** | <0,0001 | A-K |
| **Control vs. S.a** | -11,21 | -23,44 to 1,024 | No | ns | 0,1215 | A-L |
| **Control vs. L.b** | -31,33 | -42,93 to -19,73 | Yes | **** | <0,0001 | A-M |
| **Control vs. L.g** | -36,72 | -48,32 to -25,12 | Yes | **** | <0,0001 | A-N |
| **Control vs. L.s** | -31,04 | -42,65 to -19,44 | Yes | **** | <0,0001 | A-O |
| **Control vs. L.la** | -50,80 | -62,40 to -39,20 | Yes | **** | <0,0001 | A-P |
| **Control vs. L.li** | -32,00 | -43,60 to -20,40 | Yes | **** | <0,0001 | A-Q |
| **Control vs. L.n** | -30,78 | -42,38 to -19,18 | Yes | **** | <0,0001 | A-R |
| **Control vs. Control** | 1,637 | -10,59 to 13,87 | No | ns | >0,9999 | A-S |
| **Control vs. LPS** | -12,52 | -24,75 to -0,2899 | Yes | * | 0,0380 | A-T |
| **Control vs. S.a** | -44,37 | -56,60 to -32,14 | Yes | **** | <0,0001 | A-U |
| **Control vs. L.b** | -14,56 | -26,16 to -2,953 | Yes | ** | 0,0018 | A-V |
| **Control vs. L.g** | -18,83 | -30,43 to -7,224 | Yes | **** | <0,0001 | A-W |
| **Control vs. L.s** | -33,87 | -45,47 to -22,27 | Yes | **** | <0,0001 | A-X |
| **Control vs. L.la** | -26,81 | -38,41 to -15,21 | Yes | **** | <0,0001 | A-Y |
| **Control vs. L.li** | -36,06 | -47,67 to -24,46 | Yes | **** | <0,0001 | A-Z |
| **Control vs. L.n** | -20,08 | -31,68 to -8,476 | Yes | **** | <0,0001 | A-AA |
| **LPS vs. S.a** | 0,3280 | -11,90 to 12,56 | No | ns | >0,9999 | B-C |
| **LPS vs. L.b** | 32,81 | 21,20 to 44,41 | Yes | **** | <0,0001 | B-D |
| **LPS vs. L.g** | 28,54 | 16,93 to 40,14 | Yes | **** | <0,0001 | B-E |
| **LPS vs. L.s** | 13,49 | 1,892 to 25,10 | Yes | ** | 0,0065 | B-F |
| **LPS vs. L.la** | 20,56 | 8,952 to 32,16 | Yes | **** | <0,0001 | B-G |
| **LPS vs. L.li** | 11,30 | -0,3040 to 22,90 | No | ns | 0,0669 | B-H |
| **LPS vs. L.n** | 27,28 | 15,68 to 38,89 | Yes | **** | <0,0001 | B-I |
| **LPS vs. Control** | 49,11 | 36,88 to 61,34 | Yes | **** | <0,0001 | B-J |
| **LPS vs. LPS** | -10,80 | -23,03 to 1,434 | No | ns | 0,1671 | B-K |
| **LPS vs. S.a** | 36,16 | 23,93 to 48,39 | Yes | **** | <0,0001 | B-L |
| **LPS vs. L.b** | 16,03 | 4,430 to 27,63 | Yes | *** | 0,0003 | B-M |
| **LPS vs. L.g** | 10,64 | -0,9578 to 22,25 | No | ns | 0,1201 | B-N |
| **LPS vs. L.s** | 16,32 | 4,718 to 27,92 | Yes | *** | 0,0002 | B-O |
| **LPS vs. L.la** | -3,436 | -15,04 to 8,167 | No | ns | >0,9999 | B-P |
| **LPS vs. L.li** | 15,36 | 3,759 to 26,96 | Yes | *** | 0,0006 | B-Q |
| **LPS vs. L.n** | 16,58 | 4,980 to 28,19 | Yes | *** | 0,0001 | B-R |
| **LPS vs. Control** | 49,00 | 36,77 to 61,23 | Yes | **** | <0,0001 | B-S |
| **LPS vs. LPS** | 34,84 | 22,61 to 47,07 | Yes | **** | <0,0001 | B-T |
| **LPS vs. S.a** | 2,989 | -9,241 to 15,22 | No | ns | >0,9999 | B-U |
| **LPS vs. L.b** | 32,81 | 21,20 to 44,41 | Yes | **** | <0,0001 | B-V |
| **LPS vs. L.g** | 28,54 | 16,93 to 40,14 | Yes | **** | <0,0001 | B-W |
| **LPS vs. L.s** | 13,49 | 1,892 to 25,10 | Yes | ** | 0,0065 | B-X |
| **LPS vs. L.la** | 20,56 | 8,952 to 32,16 | Yes | **** | <0,0001 | B-Y |
| **LPS vs. L.li** | 11,30 | -0,3040 to 22,90 | No | ns | 0,0669 | B-Z |
| **LPS vs. L.n** | 27,28 | 15,68 to 38,89 | Yes | **** | <0,0001 | B-AA |
| **S.a vs. L.b** | 32,48 | 20,88 to 44,08 | Yes | **** | <0,0001 | C-D |
| **S.a vs. L.g** | 28,21 | 16,61 to 39,81 | Yes | **** | <0,0001 | C-E |
| **S.a vs. L.s** | 13,17 | 1,564 to 24,77 | Yes | ** | 0,0095 | C-F |
| **S.a vs. L.la** | 20,23 | 8,624 to 31,83 | Yes | **** | <0,0001 | C-G |
| **S.a vs. L.li** | 10,97 | -0,6320 to 22,57 | No | ns | 0,0904 | C-H |
| **S.a vs. L.n** | 26,96 | 15,35 to 38,56 | Yes | **** | <0,0001 | C-I |
| **S.a vs. Control** | 48,78 | 36,55 to 61,01 | Yes | **** | <0,0001 | C-J |
| **S.a vs. LPS** | -11,12 | -23,35 to 1,106 | No | ns | 0,1298 | C-K |
| **S.a vs. S.a** | 35,83 | 23,60 to 48,06 | Yes | **** | <0,0001 | C-L |
| **S.a vs. L.b** | 15,70 | 4,102 to 27,31 | Yes | *** | 0,0004 | C-M |
| **S.a vs. L.g** | 10,32 | -1,286 to 21,92 | No | ns | 0,1575 | C-N |
| **S.a vs. L.s** | 15,99 | 4,390 to 27,60 | Yes | *** | 0,0003 | C-O |
| **S.a vs. L.la** | -3,764 | -15,37 to 7,839 | No | ns | >0,9999 | C-P |
| **S.a vs. L.li** | 15,03 | 3,431 to 26,64 | Yes | *** | 0,0010 | C-Q |
| **S.a vs. L.n** | 16,25 | 4,652 to 27,86 | Yes | *** | 0,0002 | C-R |
| **S.a vs. Control** | 48,67 | 36,44 to 60,90 | Yes | **** | <0,0001 | C-S |
| **S.a vs. LPS** | 34,51 | 22,28 to 46,75 | Yes | **** | <0,0001 | C-T |
| **S.a vs. S.a** | 2,661 | -9,569 to 14,89 | No | ns | >0,9999 | C-U |
| **S.a vs. L.b** | 32,48 | 20,88 to 44,08 | Yes | **** | <0,0001 | C-V |
| **S.a vs. L.g** | 28,21 | 16,61 to 39,81 | Yes | **** | <0,0001 | C-W |
| **S.a vs. L.s** | 13,17 | 1,564 to 24,77 | Yes | ** | 0,0095 | C-X |
| **S.a vs. L.la** | 20,23 | 8,624 to 31,83 | Yes | **** | <0,0001 | C-Y |
| **S.a vs. L.li** | 10,97 | -0,6320 to 22,57 | No | ns | 0,0904 | C-Z |
| **S.a vs. L.n** | 26,96 | 15,35 to 38,56 | Yes | **** | <0,0001 | C-AA |
| **L.b vs. L.g** | -4,271 | -15,21 to 6,668 | No | ns | 0,9992 | D-E |
| **L.b vs. L.s** | -19,31 | -30,25 to -8,374 | Yes | **** | <0,0001 | D-F |
| **L.b vs. L.la** | -12,25 | -23,19 to -1,313 | Yes | * | 0,0115 | D-G |
| **L.b vs. L.li** | -21,51 | -32,45 to -10,57 | Yes | **** | <0,0001 | D-H |
| **L.b vs. L.n** | -5,523 | -16,46 to 5,416 | No | ns | 0,9737 | D-I |
| **L.b vs. Control** | 16,30 | 4,698 to 27,90 | Yes | *** | 0,0002 | D-J |
| **L.b vs. LPS** | -43,60 | -55,21 to -32,00 | Yes | **** | <0,0001 | D-K |
| **L.b vs. S.a** | 3,349 | -8,253 to 14,95 | No | ns | >0,9999 | D-L |
| **L.b vs. L.b** | -16,78 | -27,71 to -5,836 | Yes | **** | <0,0001 | D-M |
| **L.b vs. L.g** | -22,16 | -33,10 to -11,22 | Yes | **** | <0,0001 | D-N |
| **L.b vs. L.s** | -16,49 | -27,43 to -5,548 | Yes | **** | <0,0001 | D-O |
| **L.b vs. L.la** | -36,24 | -47,18 to -25,30 | Yes | **** | <0,0001 | D-P |
| **L.b vs. L.li** | -17,45 | -28,39 to -6,507 | Yes | **** | <0,0001 | D-Q |
| **L.b vs. L.n** | -16,22 | -27,16 to -5,286 | Yes | **** | <0,0001 | D-R |
| **L.b vs. Control** | 16,19 | 4,590 to 27,80 | Yes | *** | 0,0002 | D-S |
| **L.b vs. LPS** | 2,036 | -9,567 to 13,64 | No | ns | >0,9999 | D-T |
| **L.b vs. S.a** | -29,82 | -41,42 to -18,22 | Yes | **** | <0,0001 | D-U |
| **L.b vs. L.b** | 0,000 | -10,94 to 10,94 | No | ns | >0,9999 | D-V |
| **L.b vs. L.g** | -4,271 | -15,21 to 6,668 | No | ns | 0,9992 | D-W |
| **L.b vs. L.s** | -19,31 | -30,25 to -8,374 | Yes | **** | <0,0001 | D-X |
| **L.b vs. L.la** | -12,25 | -23,19 to -1,313 | Yes | * | 0,0115 | D-Y |
| **L.b vs. L.li** | -21,51 | -32,45 to -10,57 | Yes | **** | <0,0001 | D-Z |
| **L.b vs. L.n** | -5,523 | -16,46 to 5,416 | No | ns | 0,9737 | D-AA |
| **L.g vs. L.s** | -15,04 | -25,98 to -4,103 | Yes | *** | 0,0003 | E-F |
| **L.g vs. L.la** | -7,981 | -18,92 to 2,958 | No | ns | 0,5207 | E-G |
| **L.g vs. L.li** | -17,24 | -28,18 to -6,299 | Yes | **** | <0,0001 | E-H |
| **L.g vs. L.n** | -1,252 | -12,19 to 9,687 | No | ns | >0,9999 | E-I |
| **L.g vs. Control** | 20,57 | 8,969 to 32,17 | Yes | **** | <0,0001 | E-J |
| **L.g vs. LPS** | -39,33 | -50,94 to -27,73 | Yes | **** | <0,0001 | E-K |
| **L.g vs. S.a** | 7,621 | -3,982 to 19,22 | No | ns | 0,7264 | E-L |
| **L.g vs. L.b** | -12,50 | -23,44 to -1,565 | Yes | ** | 0,0085 | E-M |
| **L.g vs. L.g** | -17,89 | -28,83 to -6,952 | Yes | **** | <0,0001 | E-N |
| **L.g vs. L.s** | -12,22 | -23,15 to -1,277 | Yes | * | 0,0120 | E-O |
| **L.g vs. L.la** | -31,97 | -42,91 to -21,03 | Yes | **** | <0,0001 | E-P |
| **L.g vs. L.li** | -13,18 | -24,11 to -2,236 | Yes | ** | 0,0037 | E-Q |
| **L.g vs. L.n** | -11,95 | -22,89 to -1,014 | Yes | * | 0,0164 | E-R |
| **L.g vs. Control** | 20,46 | 8,862 to 32,07 | Yes | **** | <0,0001 | E-S |
| **L.g vs. LPS** | 6,307 | -5,296 to 17,91 | No | ns | 0,9418 | E-T |
| **L.g vs. S.a** | -25,55 | -37,15 to -13,94 | Yes | **** | <0,0001 | E-U |
| **L.g vs. L.b** | 4,271 | -6,668 to 15,21 | No | ns | 0,9992 | E-V |
| **L.g vs. L.g** | 0,000 | -10,94 to 10,94 | No | ns | >0,9999 | E-W |
| **L.g vs. L.s** | -15,04 | -25,98 to -4,103 | Yes | *** | 0,0003 | E-X |
| **L.g vs. L.la** | -7,981 | -18,92 to 2,958 | No | ns | 0,5207 | E-Y |
| **L.g vs. L.li** | -17,24 | -28,18 to -6,299 | Yes | **** | <0,0001 | E-Z |
| **L.g vs. L.n** | -1,252 | -12,19 to 9,687 | No | ns | >0,9999 | E-AA |
| **L.s vs. L.la** | 7,061 | -3,878 to 18,00 | No | ns | 0,7557 | F-G |
| **L.s vs. L.li** | -2,196 | -13,13 to 8,743 | No | ns | >0,9999 | F-H |
| **L.s vs. L.n** | 13,79 | 2,851 to 24,73 | Yes | ** | 0,0016 | F-I |
| **L.s vs. Control** | 35,61 | 24,01 to 47,22 | Yes | **** | <0,0001 | F-J |
| **L.s vs. LPS** | -24,29 | -35,89 to -12,69 | Yes | **** | <0,0001 | F-K |
| **L.s vs. S.a** | 22,66 | 11,06 to 34,27 | Yes | **** | <0,0001 | F-L |
| **L.s vs. L.b** | 2,538 | -8,401 to 13,48 | No | ns | >0,9999 | F-M |
| **L.s vs. L.g** | -2,850 | -13,79 to 8,089 | No | ns | >0,9999 | F-N |
| **L.s vs. L.s** | 2,826 | -8,113 to 13,77 | No | ns | >0,9999 | F-O |
| **L.s vs. L.la** | -16,93 | -27,87 to -5,991 | Yes | **** | <0,0001 | F-P |
| **L.s vs. L.li** | 1,867 | -9,072 to 12,81 | No | ns | >0,9999 | F-Q |
| **L.s vs. L.n** | 3,088 | -7,851 to 14,03 | No | ns | >0,9999 | F-R |
| **L.s vs. Control** | 35,51 | 23,90 to 47,11 | Yes | **** | <0,0001 | F-S |
| **L.s vs. LPS** | 21,35 | 9,746 to 32,95 | Yes | **** | <0,0001 | F-T |
| **L.s vs. S.a** | -10,50 | -22,11 to 1,098 | No | ns | 0,1351 | F-U |
| **L.s vs. L.b** | 19,31 | 8,374 to 30,25 | Yes | **** | <0,0001 | F-V |
| **L.s vs. L.g** | 15,04 | 4,103 to 25,98 | Yes | *** | 0,0003 | F-W |
| **L.s vs. L.s** | 0,000 | -10,94 to 10,94 | No | ns | >0,9999 | F-X |
| **L.s vs. L.la** | 7,061 | -3,878 to 18,00 | No | ns | 0,7557 | F-Y |
| **L.s vs. L.li** | -2,196 | -13,13 to 8,743 | No | ns | >0,9999 | F-Z |
| **L.s vs. L.n** | 13,79 | 2,851 to 24,73 | Yes | ** | 0,0016 | F-AA |
| **L.la vs. L.li** | -9,256 | -20,20 to 1,683 | No | ns | 0,2304 | G-H |
| **L.la vs. L.n** | 6,729 | -4,210 to 17,67 | No | ns | 0,8265 | G-I |
| **L.la vs. Control** | 28,55 | 16,95 to 40,16 | Yes | **** | <0,0001 | G-J |
| **L.la vs. LPS** | -31,35 | -42,95 to -19,75 | Yes | **** | <0,0001 | G-K |
| **L.la vs. S.a** | 15,60 | 3,999 to 27,20 | Yes | *** | 0,0005 | G-L |
| **L.la vs. L.b** | -4,523 | -15,46 to 6,416 | No | ns | 0,9981 | G-M |
| **L.la vs. L.g** | -9,910 | -20,85 to 1,029 | No | ns | 0,1344 | G-N |
| **L.la vs. L.s** | -4,235 | -15,17 to 6,704 | No | ns | 0,9993 | G-O |
| **L.la vs. L.la** | -23,99 | -34,93 to -13,05 | Yes | **** | <0,0001 | G-P |
| **L.la vs. L.li** | -5,194 | -16,13 to 5,745 | No | ns | 0,9874 | G-Q |
| **L.la vs. L.n** | -3,972 | -14,91 to 6,967 | No | ns | 0,9998 | G-R |
| **L.la vs. Control** | 28,45 | 16,84 to 40,05 | Yes | **** | <0,0001 | G-S |
| **L.la vs. LPS** | 14,29 | 2,685 to 25,89 | Yes | ** | 0,0025 | G-T |
| **L.la vs. S.a** | -17,57 | -29,17 to -5,963 | Yes | **** | <0,0001 | G-U |
| **L.la vs. L.b** | 12,25 | 1,313 to 23,19 | Yes | * | 0,0115 | G-V |
| **L.la vs. L.g** | 7,981 | -2,958 to 18,92 | No | ns | 0,5207 | G-W |
| **L.la vs. L.s** | -7,061 | -18,00 to 3,878 | No | ns | 0,7557 | G-X |
| **L.la vs. L.la** | 0,000 | -10,94 to 10,94 | No | ns | >0,9999 | G-Y |
| **L.la vs. L.li** | -9,256 | -20,20 to 1,683 | No | ns | 0,2304 | G-Z |
| **L.la vs. L.n** | 6,729 | -4,210 to 17,67 | No | ns | 0,8265 | G-AA |
| **L.li vs. L.n** | 15,99 | 5,047 to 26,92 | Yes | **** | <0,0001 | H-I |
| **L.li vs. Control** | 37,81 | 26,21 to 49,41 | Yes | **** | <0,0001 | H-J |
| **L.li vs. LPS** | -22,09 | -33,70 to -10,49 | Yes | **** | <0,0001 | H-K |
| **L.li vs. S.a** | 24,86 | 13,26 to 36,46 | Yes | **** | <0,0001 | H-L |
| **L.li vs. L.b** | 4,734 | -6,205 to 15,67 | No | ns | 0,9964 | H-M |
| **L.li vs. L.g** | -0,6538 | -11,59 to 10,29 | No | ns | >0,9999 | H-N |
| **L.li vs. L.s** | 5,022 | -5,917 to 15,96 | No | ns | 0,9919 | H-O |
| **L.li vs. L.la** | -14,73 | -25,67 to -3,795 | Yes | *** | 0,0004 | H-P |
| **L.li vs. L.li** | 4,063 | -6,876 to 15,00 | No | ns | 0,9997 | H-Q |
| **L.li vs. L.n** | 5,284 | -5,655 to 16,22 | No | ns | 0,9844 | H-R |
| **L.li vs. Control** | 37,70 | 26,10 to 49,30 | Yes | **** | <0,0001 | H-S |
| **L.li vs. LPS** | 23,54 | 11,94 to 35,15 | Yes | **** | <0,0001 | H-T |
| **L.li vs. S.a** | -8,309 | -19,91 to 3,293 | No | ns | 0,5596 | H-U |
| **L.li vs. L.b** | 21,51 | 10,57 to 32,45 | Yes | **** | <0,0001 | H-V |
| **L.li vs. L.g** | 17,24 | 6,299 to 28,18 | Yes | **** | <0,0001 | H-W |
| **L.li vs. L.s** | 2,196 | -8,743 to 13,13 | No | ns | >0,9999 | H-X |
| **L.li vs. L.la** | 9,256 | -1,683 to 20,20 | No | ns | 0,2304 | H-Y |
| **L.li vs. L.li** | 0,000 | -10,94 to 10,94 | No | ns | >0,9999 | H-Z |
| **L.li vs. L.n** | 15,99 | 5,047 to 26,92 | Yes | **** | <0,0001 | H-AA |
| **L.n vs. Control** | 21,82 | 10,22 to 33,43 | Yes | **** | <0,0001 | I-J |
| **L.n vs. LPS** | -38,08 | -49,68 to -26,48 | Yes | **** | <0,0001 | I-K |
| **L.n vs. S.a** | 8,872 | -2,730 to 20,48 | No | ns | 0,4220 | I-L |
| **L.n vs. L.b** | -11,25 | -22,19 to -0,3133 | Yes | * | 0,0359 | I-M |
| **L.n vs. L.g** | -16,64 | -27,58 to -5,701 | Yes | **** | <0,0001 | I-N |
| **L.n vs. L.s** | -10,96 | -21,90 to -0,02510 | Yes | * | 0,0487 | I-O |
| **L.n vs. L.la** | -30,72 | -41,66 to -19,78 | Yes | **** | <0,0001 | I-P |
| **L.n vs. L.li** | -11,92 | -22,86 to -0,9843 | Yes | * | 0,0169 | I-Q |
| **L.n vs. L.n** | -10,70 | -21,64 to 0,2373 | No | ns | 0,0637 | I-R |
| **L.n vs. Control** | 21,72 | 10,11 to 33,32 | Yes | **** | <0,0001 | I-S |
| **L.n vs. LPS** | 7,558 | -4,044 to 19,16 | No | ns | 0,7404 | I-T |
| **L.n vs. S.a** | -24,30 | -35,90 to -12,69 | Yes | **** | <0,0001 | I-U |
| **L.n vs. L.b** | 5,523 | -5,416 to 16,46 | No | ns | 0,9737 | I-V |
| **L.n vs. L.g** | 1,252 | -9,687 to 12,19 | No | ns | >0,9999 | I-W |
| **L.n vs. L.s** | -13,79 | -24,73 to -2,851 | Yes | ** | 0,0016 | I-X |
| **L.n vs. L.la** | -6,729 | -17,67 to 4,210 | No | ns | 0,8265 | I-Y |
| **L.n vs. L.li** | -15,99 | -26,92 to -5,047 | Yes | **** | <0,0001 | I-Z |
| **L.n vs. L.n** | 0,000 | -10,94 to 10,94 | No | ns | >0,9999 | I-AA |
| **Control vs. LPS** | -59,90 | -72,13 to -47,67 | Yes | **** | <0,0001 | J-K |
| **Control vs. S.a** | -12,95 | -25,18 to -0,7207 | Yes | * | 0,0250 | J-L |
| **Control vs. L.b** | -33,08 | -44,68 to -21,47 | Yes | **** | <0,0001 | J-M |
| **Control vs. L.g** | -38,46 | -50,07 to -26,86 | Yes | **** | <0,0001 | J-N |
| **Control vs. L.s** | -32,79 | -44,39 to -21,18 | Yes | **** | <0,0001 | J-O |
| **Control vs. L.la** | -52,54 | -64,15 to -40,94 | Yes | **** | <0,0001 | J-P |
| **Control vs. L.li** | -33,75 | -45,35 to -22,14 | Yes | **** | <0,0001 | J-Q |
| **Control vs. L.n** | -32,53 | -44,13 to -20,92 | Yes | **** | <0,0001 | J-R |
| **Control vs. Control** | -0,1074 | -12,34 to 12,12 | No | ns | >0,9999 | J-S |
| **Control vs. LPS** | -14,26 | -26,50 to -2,035 | Yes | ** | 0,0062 | J-T |
| **Control vs. S.a** | -46,12 | -58,35 to -33,89 | Yes | **** | <0,0001 | J-U |
| **Control vs. L.b** | -16,30 | -27,90 to -4,698 | Yes | *** | 0,0002 | J-V |
| **Control vs. L.g** | -20,57 | -32,17 to -8,969 | Yes | **** | <0,0001 | J-W |
| **Control vs. L.s** | -35,61 | -47,22 to -24,01 | Yes | **** | <0,0001 | J-X |
| **Control vs. L.la** | -28,55 | -40,16 to -16,95 | Yes | **** | <0,0001 | J-Y |
| **Control vs. L.li** | -37,81 | -49,41 to -26,21 | Yes | **** | <0,0001 | J-Z |
| **Control vs. L.n** | -21,82 | -33,43 to -10,22 | Yes | **** | <0,0001 | J-AA |
| **LPS vs. S.a** | 46,95 | 34,72 to 59,18 | Yes | **** | <0,0001 | K-L |
| **LPS vs. L.b** | 26,83 | 15,23 to 38,43 | Yes | **** | <0,0001 | K-M |
| **LPS vs. L.g** | 21,44 | 9,839 to 33,04 | Yes | **** | <0,0001 | K-N |
| **LPS vs. L.s** | 27,12 | 15,51 to 38,72 | Yes | **** | <0,0001 | K-O |
| **LPS vs. L.la** | 7,361 | -4,242 to 18,96 | No | ns | 0,7829 | K-P |
| **LPS vs. L.li** | 26,16 | 14,55 to 37,76 | Yes | **** | <0,0001 | K-Q |
| **LPS vs. L.n** | 27,38 | 15,78 to 38,98 | Yes | **** | <0,0001 | K-R |
| **LPS vs. Control** | 59,80 | 47,57 to 72,03 | Yes | **** | <0,0001 | K-S |
| **LPS vs. LPS** | 45,64 | 33,41 to 57,87 | Yes | **** | <0,0001 | K-T |
| **LPS vs. S.a** | 13,79 | 1,556 to 26,02 | Yes | * | 0,0105 | K-U |
| **LPS vs. L.b** | 43,60 | 32,00 to 55,21 | Yes | **** | <0,0001 | K-V |
| **LPS vs. L.g** | 39,33 | 27,73 to 50,94 | Yes | **** | <0,0001 | K-W |
| **LPS vs. L.s** | 24,29 | 12,69 to 35,89 | Yes | **** | <0,0001 | K-X |
| **LPS vs. L.la** | 31,35 | 19,75 to 42,95 | Yes | **** | <0,0001 | K-Y |
| **LPS vs. L.li** | 22,09 | 10,49 to 33,70 | Yes | **** | <0,0001 | K-Z |
| **LPS vs. L.n** | 38,08 | 26,48 to 49,68 | Yes | **** | <0,0001 | K-AA |
| **S.a vs. L.b** | -20,12 | -31,73 to -8,522 | Yes | **** | <0,0001 | L-M |
| **S.a vs. L.g** | -25,51 | -37,11 to -13,91 | Yes | **** | <0,0001 | L-N |
| **S.a vs. L.s** | -19,84 | -31,44 to -8,234 | Yes | **** | <0,0001 | L-O |
| **S.a vs. L.la** | -39,59 | -51,20 to -27,99 | Yes | **** | <0,0001 | L-P |
| **S.a vs. L.li** | -20,80 | -32,40 to -9,193 | Yes | **** | <0,0001 | L-Q |
| **S.a vs. L.n** | -19,57 | -31,18 to -7,972 | Yes | **** | <0,0001 | L-R |
| **S.a vs. Control** | 12,84 | 0,6133 to 25,07 | Yes | * | 0,0278 | L-S |
| **S.a vs. LPS** | -1,314 | -13,54 to 10,92 | No | ns | >0,9999 | L-T |
| **S.a vs. S.a** | -33,17 | -45,40 to -20,94 | Yes | **** | <0,0001 | L-U |
| **S.a vs. L.b** | -3,349 | -14,95 to 8,253 | No | ns | >0,9999 | L-V |
| **S.a vs. L.g** | -7,621 | -19,22 to 3,982 | No | ns | 0,7264 | L-W |
| **S.a vs. L.s** | -22,66 | -34,27 to -11,06 | Yes | **** | <0,0001 | L-X |
| **S.a vs. L.la** | -15,60 | -27,20 to -3,999 | Yes | *** | 0,0005 | L-Y |
| **S.a vs. L.li** | -24,86 | -36,46 to -13,26 | Yes | **** | <0,0001 | L-Z |
| **S.a vs. L.n** | -8,872 | -20,48 to 2,730 | No | ns | 0,4220 | L-AA |
| **L.b vs. L.g** | -5,387 | -16,33 to 5,552 | No | ns | 0,9803 | M-N |
| **L.b vs. L.s** | 0,2882 | -10,65 to 11,23 | No | ns | >0,9999 | M-O |
| **L.b vs. L.la** | -19,47 | -30,41 to -8,529 | Yes | **** | <0,0001 | M-P |
| **L.b vs. L.li** | -0,6710 | -11,61 to 10,27 | No | ns | >0,9999 | M-Q |
| **L.b vs. L.n** | 0,5506 | -10,39 to 11,49 | No | ns | >0,9999 | M-R |
| **L.b vs. Control** | 32,97 | 21,37 to 44,57 | Yes | **** | <0,0001 | M-S |
| **L.b vs. LPS** | 18,81 | 7,208 to 30,41 | Yes | **** | <0,0001 | M-T |
| **L.b vs. S.a** | -13,04 | -24,65 to -1,440 | Yes | * | 0,0109 | M-U |
| **L.b vs. L.b** | 16,78 | 5,836 to 27,71 | Yes | **** | <0,0001 | M-V |
| **L.b vs. L.g** | 12,50 | 1,565 to 23,44 | Yes | ** | 0,0085 | M-W |
| **L.b vs. L.s** | -2,538 | -13,48 to 8,401 | No | ns | >0,9999 | M-X |
| **L.b vs. L.la** | 4,523 | -6,416 to 15,46 | No | ns | 0,9981 | M-Y |
| **L.b vs. L.li** | -4,734 | -15,67 to 6,205 | No | ns | 0,9964 | M-Z |
| **L.b vs. L.n** | 11,25 | 0,3133 to 22,19 | Yes | * | 0,0359 | M-AA |
| **L.g vs. L.s** | 5,676 | -5,263 to 16,61 | No | ns | 0,9643 | N-O |
| **L.g vs. L.la** | -14,08 | -25,02 to -3,141 | Yes | ** | 0,0011 | N-P |
| **L.g vs. L.li** | 4,716 | -6,223 to 15,66 | No | ns | 0,9966 | N-Q |
| **L.g vs. L.n** | 5,938 | -5,001 to 16,88 | No | ns | 0,9426 | N-R |
| **L.g vs. Control** | 38,36 | 26,75 to 49,96 | Yes | **** | <0,0001 | N-S |
| **L.g vs. LPS** | 24,20 | 12,60 to 35,80 | Yes | **** | <0,0001 | N-T |
| **L.g vs. S.a** | -7,655 | -19,26 to 3,947 | No | ns | 0,7185 | N-U |
| **L.g vs. L.b** | 22,16 | 11,22 to 33,10 | Yes | **** | <0,0001 | N-V |
| **L.g vs. L.g** | 17,89 | 6,952 to 28,83 | Yes | **** | <0,0001 | N-W |
| **L.g vs. L.s** | 2,850 | -8,089 to 13,79 | No | ns | >0,9999 | N-X |
| **L.g vs. L.la** | 9,910 | -1,029 to 20,85 | No | ns | 0,1344 | N-Y |
| **L.g vs. L.li** | 0,6538 | -10,29 to 11,59 | No | ns | >0,9999 | N-Z |
| **L.g vs. L.n** | 16,64 | 5,701 to 27,58 | Yes | **** | <0,0001 | N-AA |
| **L.s vs. L.la** | -19,76 | -30,70 to -8,817 | Yes | **** | <0,0001 | O-P |
| **L.s vs. L.li** | -0,9592 | -11,90 to 9,980 | No | ns | >0,9999 | O-Q |
| **L.s vs. L.n** | 0,2624 | -10,68 to 11,20 | No | ns | >0,9999 | O-R |
| **L.s vs. Control** | 32,68 | 21,08 to 44,28 | Yes | **** | <0,0001 | O-S |
| **L.s vs. LPS** | 18,52 | 6,920 to 30,13 | Yes | **** | <0,0001 | O-T |
| **L.s vs. S.a** | -13,33 | -24,93 to -1,728 | Yes | ** | 0,0078 | O-U |
| **L.s vs. L.b** | 16,49 | 5,548 to 27,43 | Yes | **** | <0,0001 | O-V |
| **L.s vs. L.g** | 12,22 | 1,277 to 23,15 | Yes | * | 0,0120 | O-W |
| **L.s vs. L.s** | -2,826 | -13,77 to 8,113 | No | ns | >0,9999 | O-X |
| **L.s vs. L.la** | 4,235 | -6,704 to 15,17 | No | ns | 0,9993 | O-Y |
| **L.s vs. L.li** | -5,022 | -15,96 to 5,917 | No | ns | 0,9919 | O-Z |
| **L.s vs. L.n** | 10,96 | 0,02510 to 21,90 | Yes | * | 0,0487 | O-AA |
| **L.la vs. L.li** | 18,80 | 7,858 to 29,74 | Yes | **** | <0,0001 | P-Q |
| **L.la vs. L.n** | 20,02 | 9,079 to 30,96 | Yes | **** | <0,0001 | P-R |
| **L.la vs. Control** | 52,44 | 40,83 to 64,04 | Yes | **** | <0,0001 | P-S |
| **L.la vs. LPS** | 38,28 | 26,68 to 49,88 | Yes | **** | <0,0001 | P-T |
| **L.la vs. S.a** | 6,425 | -5,177 to 18,03 | No | ns | 0,9300 | P-U |
| **L.la vs. L.b** | 36,24 | 25,30 to 47,18 | Yes | **** | <0,0001 | P-V |
| **L.la vs. L.g** | 31,97 | 21,03 to 42,91 | Yes | **** | <0,0001 | P-W |
| **L.la vs. L.s** | 16,93 | 5,991 to 27,87 | Yes | **** | <0,0001 | P-X |
| **L.la vs. L.la** | 23,99 | 13,05 to 34,93 | Yes | **** | <0,0001 | P-Y |
| **L.la vs. L.li** | 14,73 | 3,795 to 25,67 | Yes | *** | 0,0004 | P-Z |
| **L.la vs. L.n** | 30,72 | 19,78 to 41,66 | Yes | **** | <0,0001 | P-AA |
| **L.li vs. L.n** | 1,222 | -9,717 to 12,16 | No | ns | >0,9999 | Q-R |
| **L.li vs. Control** | 33,64 | 22,04 to 45,24 | Yes | **** | <0,0001 | Q-S |
| **L.li vs. LPS** | 19,48 | 7,879 to 31,08 | Yes | **** | <0,0001 | Q-T |
| **L.li vs. S.a** | -12,37 | -23,97 to -0,7692 | Yes | * | 0,0228 | Q-U |
| **L.li vs. L.b** | 17,45 | 6,507 to 28,39 | Yes | **** | <0,0001 | Q-V |
| **L.li vs. L.g** | 13,18 | 2,236 to 24,11 | Yes | ** | 0,0037 | Q-W |
| **L.li vs. L.s** | -1,867 | -12,81 to 9,072 | No | ns | >0,9999 | Q-X |
| **L.li vs. L.la** | 5,194 | -5,745 to 16,13 | No | ns | 0,9874 | Q-Y |
| **L.li vs. L.li** | -4,063 | -15,00 to 6,876 | No | ns | 0,9997 | Q-Z |
| **L.li vs. L.n** | 11,92 | 0,9843 to 22,86 | Yes | * | 0,0169 | Q-AA |
| **L.n vs. Control** | 32,42 | 20,82 to 44,02 | Yes | **** | <0,0001 | R-S |
| **L.n vs. LPS** | 18,26 | 6,658 to 29,86 | Yes | **** | <0,0001 | R-T |
| **L.n vs. S.a** | -13,59 | -25,20 to -1,991 | Yes | ** | 0,0058 | R-U |
| **L.n vs. L.b** | 16,22 | 5,286 to 27,16 | Yes | **** | <0,0001 | R-V |
| **L.n vs. L.g** | 11,95 | 1,014 to 22,89 | Yes | * | 0,0164 | R-W |
| **L.n vs. L.s** | -3,088 | -14,03 to 7,851 | No | ns | >0,9999 | R-X |
| **L.n vs. L.la** | 3,972 | -6,967 to 14,91 | No | ns | 0,9998 | R-Y |
| **L.n vs. L.li** | -5,284 | -16,22 to 5,655 | No | ns | 0,9844 | R-Z |
| **L.n vs. L.n** | 10,70 | -0,2373 to 21,64 | No | ns | 0,0637 | R-AA |
| **Control vs. LPS** | -14,16 | -26,39 to -1,927 | Yes | ** | 0,0070 | S-T |
| **Control vs. S.a** | -46,01 | -58,24 to -33,78 | Yes | **** | <0,0001 | S-U |
| **Control vs. L.b** | -16,19 | -27,80 to -4,590 | Yes | *** | 0,0002 | S-V |
| **Control vs. L.g** | -20,46 | -32,07 to -8,862 | Yes | **** | <0,0001 | S-W |
| **Control vs. L.s** | -35,51 | -47,11 to -23,90 | Yes | **** | <0,0001 | S-X |
| **Control vs. L.la** | -28,45 | -40,05 to -16,84 | Yes | **** | <0,0001 | S-Y |
| **Control vs. L.li** | -37,70 | -49,30 to -26,10 | Yes | **** | <0,0001 | S-Z |
| **Control vs. L.n** | -21,72 | -33,32 to -10,11 | Yes | **** | <0,0001 | S-AA |
| **LPS vs. S.a** | -31,85 | -44,08 to -19,62 | Yes | **** | <0,0001 | T-U |
| **LPS vs. L.b** | -2,036 | -13,64 to 9,567 | No | ns | >0,9999 | T-V |
| **LPS vs. L.g** | -6,307 | -17,91 to 5,296 | No | ns | 0,9418 | T-W |
| **LPS vs. L.s** | -21,35 | -32,95 to -9,746 | Yes | **** | <0,0001 | T-X |
| **LPS vs. L.la** | -14,29 | -25,89 to -2,685 | Yes | ** | 0,0025 | T-Y |
| **LPS vs. L.li** | -23,54 | -35,15 to -11,94 | Yes | **** | <0,0001 | T-Z |
| **LPS vs. L.n** | -7,558 | -19,16 to 4,044 | No | ns | 0,7404 | T-AA |
| **S.a vs. L.b** | 29,82 | 18,22 to 41,42 | Yes | **** | <0,0001 | U-V |
| **S.a vs. L.g** | 25,55 | 13,94 to 37,15 | Yes | **** | <0,0001 | U-W |
| **S.a vs. L.s** | 10,50 | -1,098 to 22,11 | No | ns | 0,1351 | U-X |
| **S.a vs. L.la** | 17,57 | 5,963 to 29,17 | Yes | **** | <0,0001 | U-Y |
| **S.a vs. L.li** | 8,309 | -3,293 to 19,91 | No | ns | 0,5596 | U-Z |
| **S.a vs. L.n** | 24,30 | 12,69 to 35,90 | Yes | **** | <0,0001 | U-AA |
| **L.b vs. L.g** | -4,271 | -15,21 to 6,668 | No | ns | 0,9992 | V-W |
| **L.b vs. L.s** | -19,31 | -30,25 to -8,374 | Yes | **** | <0,0001 | V-X |
| **L.b vs. L.la** | -12,25 | -23,19 to -1,313 | Yes | * | 0,0115 | V-Y |
| **L.b vs. L.li** | -21,51 | -32,45 to -10,57 | Yes | **** | <0,0001 | V-Z |
| **L.b vs. L.n** | -5,523 | -16,46 to 5,416 | No | ns | 0,9737 | V-AA |
| **L.g vs. L.s** | -15,04 | -25,98 to -4,103 | Yes | *** | 0,0003 | W-X |
| **L.g vs. L.la** | -7,981 | -18,92 to 2,958 | No | ns | 0,5207 | W-Y |
| **L.g vs. L.li** | -17,24 | -28,18 to -6,299 | Yes | **** | <0,0001 | W-Z |
| **L.g vs. L.n** | -1,252 | -12,19 to 9,687 | No | ns | >0,9999 | W-AA |
| **L.s vs. L.la** | 7,061 | -3,878 to 18,00 | No | ns | 0,7557 | X-Y |
| **L.s vs. L.li** | -2,196 | -13,13 to 8,743 | No | ns | >0,9999 | X-Z |
| **L.s vs. L.n** | 13,79 | 2,851 to 24,73 | Yes | ** | 0,0016 | X-AA |
| **L.la vs. L.li** | -9,256 | -20,20 to 1,683 | No | ns | 0,2304 | Y-Z |
| **L.la vs. L.n** | 6,729 | -4,210 to 17,67 | No | ns | 0,8265 | Y-AA |
| **L.li vs. L.n** | 15,99 | 5,047 to 26,92 | Yes | **** | <0,0001 | Z-AA |

**GIPL (WT, TLR2KO and TLR4KO)**

| **Control** | **LPS** | **S.a** | **L.b** | **L.g** | **L.s** | **L.la** | **L.li** | **L.n** |
| --- | --- | --- | --- | --- | --- | --- | --- | --- |
| 1,996316 | 48,6982 | 46,92389 | 20,10499 | 17,12631 | 22,43847 | 17,78226 | 18,30918 | 21,48142 |
| 2,136553 | 48,26806 | 50,53702 | 19,37376 | 18,81458 | 21,11581 | 15,56707 | 20,07273 | 19,90067 |
| 7,534301 | 49,90257 | 53,38667 | 19,71787 | 17,29836 | 23,52456 | 19,37376 | 22,99765 | 19,88992 |
| 2,340423 | 56,59117 | 51,30051 | 18,19089 | 20,3093 | 26,43872 | 22,5245 | 18,38445 | 17,73925 |
|  |  |  | 19,42753 | 16,59939 | 21,93306 | 23,77189 | 15,78213 | 16,92199 |
| **Control** | **LPS** | **S.a** | **L.g** | **L.s** | **L.la** | **L.b** | **L.n** | **L.li** |
| 2,211826 | 58,35472 | 17,34264 | 37,23511 | 37,46093 | 50,37572 | 45,34315 | 32,23479 | 36,19203 |
| 1,125293 | 63,04319 | 14,15964 | 26,62153 | 35,84792 | 52,24681 | 29,52494 | 29,34213 | 34,07362 |
| 1,4694 | 61,87107 | 14,10588 | 25,27736 | 27,47105 | 51,88119 | 24,34182 | 27,97645 | 34,60053 |
| 2,222136 | 63,37655 | 13,2241 | 32,17027 | 36,98778 | 52,28982 | 25,93331 | 29,18083 | 28,46036 |
|  |  |  | 27,67536 | 33,03054 | 55,53734 | 27,00865 | 33,2241 | 34,66505 |
| **Control** | **LPS** | **S.a** | **L.g** | **L.s** | **L.la** | **L.b** | **L.n** | **L.li** |
| 0,856458 | 14,54613 | 48,22505 | 17,12631 | 22,43847 | 17,78226 | 20,10499 | 21,48142 | 18,30918 |
| 2,598504 | 19,67485 | 51,0962 | 18,81458 | 21,11581 | 15,56707 | 19,37376 | 19,90067 | 20,07273 |
| 1,501661 | 13,17033 | 44,16028 | 17,29836 | 23,52456 | 19,37376 | 19,71787 | 19,88992 | 22,99765 |
| 2,501724 | 16,6968 | 48,02073 | 20,3093 | 26,43872 | 22,5245 | 18,19089 | 17,73925 | 18,38445 |
|  |  |  | 16,59939 | 21,93306 | 23,77189 | 19,42753 | 16,92199 | 15,78213 |

**Statistical analysis**

| **Tukey's multiple comparisons test** | **Mean Diff,** | **95,00% CI of diff,** | **Significant?** | **Summary** | **Adjusted P Value** |  |
| --- | --- | --- | --- | --- | --- | --- |
| **Control vs. LPS** | -47,36 | -55,58 to -39,15 | Yes | **** | <0,0001 | A-B |
| **Control vs. S.a** | -47,04 | -55,25 to -38,82 | Yes | **** | <0,0001 | A-C |
| **Control vs. L.b** | -15,86 | -23,65 to -8,069 | Yes | **** | <0,0001 | A-D |
| **Control vs. L.g** | -14,53 | -22,32 to -6,736 | Yes | **** | <0,0001 | A-E |
| **Control vs. L.s** | -19,59 | -27,38 to -11,80 | Yes | **** | <0,0001 | A-F |
| **Control vs. L.la** | -16,30 | -24,09 to -8,510 | Yes | **** | <0,0001 | A-G |
| **Control vs. L.li** | -15,61 | -23,40 to -7,816 | Yes | **** | <0,0001 | A-H |
| **Control vs. L.n** | -15,68 | -23,48 to -7,893 | Yes | **** | <0,0001 | A-I |
| **Control vs. Control** | 1,745 | -6,468 to 9,958 | No | ns | >0,9999 | A-J |
| **Control vs. LPS** | -58,16 | -66,37 to -49,95 | Yes | **** | <0,0001 | A-K |
| **Control vs. S.a** | -11,21 | -19,42 to -2,993 | Yes | *** | 0,0003 | A-L |
| **Control vs. L.g** | -26,29 | -34,09 to -18,50 | Yes | **** | <0,0001 | A-M |
| **Control vs. L.s** | -30,66 | -38,45 to -22,87 | Yes | **** | <0,0001 | A-N |
| **Control vs. L.la** | -48,96 | -56,76 to -41,17 | Yes | **** | <0,0001 | A-O |
| **Control vs. L.b** | -26,93 | -34,72 to -19,14 | Yes | **** | <0,0001 | A-P |
| **Control vs. L.n** | -26,89 | -34,68 to -19,10 | Yes | **** | <0,0001 | A-Q |
| **Control vs. L.li** | -30,10 | -37,89 to -22,30 | Yes | **** | <0,0001 | A-R |
| **Control vs. Control** | 1,637 | -6,576 to 9,850 | No | ns | >0,9999 | A-S |
| **Control vs. LPS** | -12,52 | -20,73 to -4,307 | Yes | **** | <0,0001 | A-T |
| **Control vs. S.a** | -44,37 | -52,59 to -36,16 | Yes | **** | <0,0001 | A-U |
| **Control vs. L.g** | -14,53 | -22,32 to -6,736 | Yes | **** | <0,0001 | A-V |
| **Control vs. L.s** | -19,59 | -27,38 to -11,80 | Yes | **** | <0,0001 | A-W |
| **Control vs. L.la** | -16,30 | -24,09 to -8,510 | Yes | **** | <0,0001 | A-X |
| **Control vs. L.b** | -15,86 | -23,65 to -8,069 | Yes | **** | <0,0001 | A-Y |
| **Control vs. L.n** | -15,68 | -23,48 to -7,893 | Yes | **** | <0,0001 | A-Z |
| **Control vs. L.li** | -15,61 | -23,40 to -7,816 | Yes | **** | <0,0001 | A-AA |
| **LPS vs. S.a** | 0,3280 | -7,885 to 8,541 | No | ns | >0,9999 | B-C |
| **LPS vs. L.b** | 31,50 | 23,71 to 39,29 | Yes | **** | <0,0001 | B-D |
| **LPS vs. L.g** | 32,84 | 25,04 to 40,63 | Yes | **** | <0,0001 | B-E |
| **LPS vs. L.s** | 27,77 | 19,98 to 35,57 | Yes | **** | <0,0001 | B-F |
| **LPS vs. L.la** | 31,06 | 23,27 to 38,85 | Yes | **** | <0,0001 | B-G |
| **LPS vs. L.li** | 31,76 | 23,96 to 39,55 | Yes | **** | <0,0001 | B-H |
| **LPS vs. L.n** | 31,68 | 23,89 to 39,47 | Yes | **** | <0,0001 | B-I |
| **LPS vs. Control** | 49,11 | 40,89 to 57,32 | Yes | **** | <0,0001 | B-J |
| **LPS vs. LPS** | -10,80 | -19,01 to -2,583 | Yes | *** | 0,0007 | B-K |
| **LPS vs. S.a** | 36,16 | 27,94 to 44,37 | Yes | **** | <0,0001 | B-L |
| **LPS vs. L.g** | 21,07 | 13,28 to 28,86 | Yes | **** | <0,0001 | B-M |
| **LPS vs. L.s** | 16,71 | 8,914 to 24,50 | Yes | **** | <0,0001 | B-N |
| **LPS vs. L.la** | -1,601 | -9,393 to 6,191 | No | ns | >0,9999 | B-O |
| **LPS vs. L.b** | 20,43 | 12,64 to 28,23 | Yes | **** | <0,0001 | B-P |
| **LPS vs. L.n** | 20,47 | 12,68 to 28,27 | Yes | **** | <0,0001 | B-Q |
| **LPS vs. L.li** | 17,27 | 9,475 to 25,06 | Yes | **** | <0,0001 | B-R |
| **LPS vs. Control** | 49,00 | 40,79 to 57,21 | Yes | **** | <0,0001 | B-S |
| **LPS vs. LPS** | 34,84 | 26,63 to 43,06 | Yes | **** | <0,0001 | B-T |
| **LPS vs. S.a** | 2,989 | -5,224 to 11,20 | No | ns | 0,9998 | B-U |
| **LPS vs. L.g** | 32,84 | 25,04 to 40,63 | Yes | **** | <0,0001 | B-V |
| **LPS vs. L.s** | 27,77 | 19,98 to 35,57 | Yes | **** | <0,0001 | B-W |
| **LPS vs. L.la** | 31,06 | 23,27 to 38,85 | Yes | **** | <0,0001 | B-X |
| **LPS vs. L.b** | 31,50 | 23,71 to 39,29 | Yes | **** | <0,0001 | B-Y |
| **LPS vs. L.n** | 31,68 | 23,89 to 39,47 | Yes | **** | <0,0001 | B-Z |
| **LPS vs. L.li** | 31,76 | 23,96 to 39,55 | Yes | **** | <0,0001 | B-AA |
| **S.a vs. L.b** | 31,17 | 23,38 to 38,97 | Yes | **** | <0,0001 | C-D |
| **S.a vs. L.g** | 32,51 | 24,72 to 40,30 | Yes | **** | <0,0001 | C-E |
| **S.a vs. L.s** | 27,45 | 19,66 to 35,24 | Yes | **** | <0,0001 | C-F |
| **S.a vs. L.la** | 30,73 | 22,94 to 38,52 | Yes | **** | <0,0001 | C-G |
| **S.a vs. L.li** | 31,43 | 23,64 to 39,22 | Yes | **** | <0,0001 | C-H |
| **S.a vs. L.n** | 31,35 | 23,56 to 39,14 | Yes | **** | <0,0001 | C-I |
| **S.a vs. Control** | 48,78 | 40,57 to 56,99 | Yes | **** | <0,0001 | C-J |
| **S.a vs. LPS** | -11,12 | -19,34 to -2,911 | Yes | *** | 0,0004 | C-K |
| **S.a vs. S.a** | 35,83 | 27,62 to 44,04 | Yes | **** | <0,0001 | C-L |
| **S.a vs. L.g** | 20,74 | 12,95 to 28,53 | Yes | **** | <0,0001 | C-M |
| **S.a vs. L.s** | 16,38 | 8,586 to 24,17 | Yes | **** | <0,0001 | C-N |
| **S.a vs. L.la** | -1,929 | -9,721 to 5,863 | No | ns | >0,9999 | C-O |
| **S.a vs. L.b** | 20,11 | 12,31 to 27,90 | Yes | **** | <0,0001 | C-P |
| **S.a vs. L.n** | 20,15 | 12,35 to 27,94 | Yes | **** | <0,0001 | C-Q |
| **S.a vs. L.li** | 16,94 | 9,147 to 24,73 | Yes | **** | <0,0001 | C-R |
| **S.a vs. Control** | 48,67 | 40,46 to 56,89 | Yes | **** | <0,0001 | C-S |
| **S.a vs. LPS** | 34,51 | 26,30 to 42,73 | Yes | **** | <0,0001 | C-T |
| **S.a vs. S.a** | 2,661 | -5,552 to 10,87 | No | ns | >0,9999 | C-U |
| **S.a vs. L.g** | 32,51 | 24,72 to 40,30 | Yes | **** | <0,0001 | C-V |
| **S.a vs. L.s** | 27,45 | 19,66 to 35,24 | Yes | **** | <0,0001 | C-W |
| **S.a vs. L.la** | 30,73 | 22,94 to 38,52 | Yes | **** | <0,0001 | C-X |
| **S.a vs. L.b** | 31,17 | 23,38 to 38,97 | Yes | **** | <0,0001 | C-Y |
| **S.a vs. L.n** | 31,35 | 23,56 to 39,14 | Yes | **** | <0,0001 | C-Z |
| **S.a vs. L.li** | 31,43 | 23,64 to 39,22 | Yes | **** | <0,0001 | C-AA |
| **L.b vs. L.g** | 1,333 | -6,013 to 8,679 | No | ns | >0,9999 | D-E |
| **L.b vs. L.s** | -3,727 | -11,07 to 3,619 | No | ns | 0,9722 | D-F |
| **L.b vs. L.la** | -0,4409 | -7,787 to 6,905 | No | ns | >0,9999 | D-G |
| **L.b vs. L.li** | 0,2538 | -7,092 to 7,600 | No | ns | >0,9999 | D-H |
| **L.b vs. L.n** | 0,1764 | -7,170 to 7,522 | No | ns | >0,9999 | D-I |
| **L.b vs. Control** | 17,61 | 9,814 to 25,40 | Yes | **** | <0,0001 | D-J |
| **L.b vs. LPS** | -42,30 | -50,09 to -34,51 | Yes | **** | <0,0001 | D-K |
| **L.b vs. S.a** | 4,655 | -3,137 to 12,45 | No | ns | 0,8623 | D-L |
| **L.b vs. L.g** | -10,43 | -17,78 to -3,087 | Yes | *** | 0,0001 | D-M |
| **L.b vs. L.s** | -14,80 | -22,14 to -7,451 | Yes | **** | <0,0001 | D-N |
| **L.b vs. L.la** | -33,10 | -40,45 to -25,76 | Yes | **** | <0,0001 | D-O |
| **L.b vs. L.b** | -11,07 | -18,41 to -3,721 | Yes | **** | <0,0001 | D-P |
| **L.b vs. L.n** | -11,03 | -18,37 to -3,683 | Yes | **** | <0,0001 | D-Q |
| **L.b vs. L.li** | -14,24 | -21,58 to -6,889 | Yes | **** | <0,0001 | D-R |
| **L.b vs. Control** | 17,50 | 9,707 to 25,29 | Yes | **** | <0,0001 | D-S |
| **L.b vs. LPS** | 3,341 | -4,451 to 11,13 | No | ns | 0,9968 | D-T |
| **L.b vs. S.a** | -28,51 | -36,30 to -20,72 | Yes | **** | <0,0001 | D-U |
| **L.b vs. L.g** | 1,333 | -6,013 to 8,679 | No | ns | >0,9999 | D-V |
| **L.b vs. L.s** | -3,727 | -11,07 to 3,619 | No | ns | 0,9722 | D-W |
| **L.b vs. L.la** | -0,4409 | -7,787 to 6,905 | No | ns | >0,9999 | D-X |
| **L.b vs. L.b** | 0,000 | -7,346 to 7,346 | No | ns | >0,9999 | D-Y |
| **L.b vs. L.n** | 0,1764 | -7,170 to 7,522 | No | ns | >0,9999 | D-Z |
| **L.b vs. L.li** | 0,2538 | -7,092 to 7,600 | No | ns | >0,9999 | D-AA |
| **L.g vs. L.s** | -5,061 | -12,41 to 2,286 | No | ns | 0,6381 | E-F |
| **L.g vs. L.la** | -1,774 | -9,120 to 5,572 | No | ns | >0,9999 | E-G |
| **L.g vs. L.li** | -1,080 | -8,426 to 6,266 | No | ns | >0,9999 | E-H |
| **L.g vs. L.n** | -1,157 | -8,503 to 6,189 | No | ns | >0,9999 | E-I |
| **L.g vs. Control** | 16,27 | 8,481 to 24,06 | Yes | **** | <0,0001 | E-J |
| **L.g vs. LPS** | -43,63 | -51,42 to -35,84 | Yes | **** | <0,0001 | E-K |
| **L.g vs. S.a** | 3,322 | -4,470 to 11,11 | No | ns | 0,9971 | E-L |
| **L.g vs. L.g** | -11,77 | -19,11 to -4,420 | Yes | **** | <0,0001 | E-M |
| **L.g vs. L.s** | -16,13 | -23,48 to -8,784 | Yes | **** | <0,0001 | E-N |
| **L.g vs. L.la** | -34,44 | -41,78 to -27,09 | Yes | **** | <0,0001 | E-O |
| **L.g vs. L.b** | -12,40 | -19,75 to -5,055 | Yes | **** | <0,0001 | E-P |
| **L.g vs. L.n** | -12,36 | -19,71 to -5,016 | Yes | **** | <0,0001 | E-Q |
| **L.g vs. L.li** | -15,57 | -22,91 to -8,223 | Yes | **** | <0,0001 | E-R |
| **L.g vs. Control** | 16,17 | 8,373 to 23,96 | Yes | **** | <0,0001 | E-S |
| **L.g vs. LPS** | 2,008 | -5,784 to 9,799 | No | ns | >0,9999 | E-T |
| **L.g vs. S.a** | -29,85 | -37,64 to -22,05 | Yes | **** | <0,0001 | E-U |
| **L.g vs. L.g** | 0,000 | -7,346 to 7,346 | No | ns | >0,9999 | E-V |
| **L.g vs. L.s** | -5,061 | -12,41 to 2,286 | No | ns | 0,6381 | E-W |
| **L.g vs. L.la** | -1,774 | -9,120 to 5,572 | No | ns | >0,9999 | E-X |
| **L.g vs. L.b** | -1,333 | -8,679 to 6,013 | No | ns | >0,9999 | E-Y |
| **L.g vs. L.n** | -1,157 | -8,503 to 6,189 | No | ns | >0,9999 | E-Z |
| **L.g vs. L.li** | -1,080 | -8,426 to 6,266 | No | ns | >0,9999 | E-AA |
| **L.s vs. L.la** | 3,286 | -4,060 to 10,63 | No | ns | 0,9943 | F-G |
| **L.s vs. L.li** | 3,981 | -3,365 to 11,33 | No | ns | 0,9435 | F-H |
| **L.s vs. L.n** | 3,903 | -3,443 to 11,25 | No | ns | 0,9539 | F-I |
| **L.s vs. Control** | 21,33 | 13,54 to 29,12 | Yes | **** | <0,0001 | F-J |
| **L.s vs. LPS** | -38,57 | -46,36 to -30,78 | Yes | **** | <0,0001 | F-K |
| **L.s vs. S.a** | 8,382 | 0,5904 to 16,17 | Yes | * | 0,0203 | F-L |
| **L.s vs. L.g** | -6,706 | -14,05 to 0,6403 | No | ns | 0,1257 | F-M |
| **L.s vs. L.s** | -11,07 | -18,42 to -3,723 | Yes | **** | <0,0001 | F-N |
| **L.s vs. L.la** | -29,38 | -36,72 to -22,03 | Yes | **** | <0,0001 | F-O |
| **L.s vs. L.b** | -7,340 | -14,69 to 0,005822 | No | ns | 0,0505 | F-P |
| **L.s vs. L.n** | -7,302 | -14,65 to 0,04454 | No | ns | 0,0535 | F-Q |
| **L.s vs. L.li** | -10,51 | -17,85 to -3,162 | Yes | *** | 0,0001 | F-R |
| **L.s vs. Control** | 21,23 | 13,43 to 29,02 | Yes | **** | <0,0001 | F-S |
| **L.s vs. LPS** | 7,068 | -0,7236 to 14,86 | No | ns | 0,1329 | F-T |
| **L.s vs. S.a** | -24,79 | -32,58 to -16,99 | Yes | **** | <0,0001 | F-U |
| **L.s vs. L.g** | 5,061 | -2,286 to 12,41 | No | ns | 0,6381 | F-V |
| **L.s vs. L.s** | 0,000 | -7,346 to 7,346 | No | ns | >0,9999 | F-W |
| **L.s vs. L.la** | 3,286 | -4,060 to 10,63 | No | ns | 0,9943 | F-X |
| **L.s vs. L.b** | 3,727 | -3,619 to 11,07 | No | ns | 0,9722 | F-Y |
| **L.s vs. L.n** | 3,903 | -3,443 to 11,25 | No | ns | 0,9539 | F-Z |
| **L.s vs. L.li** | 3,981 | -3,365 to 11,33 | No | ns | 0,9435 | F-AA |
| **L.la vs. L.li** | 0,6947 | -6,651 to 8,041 | No | ns | >0,9999 | G-H |
| **L.la vs. L.n** | 0,6172 | -6,729 to 7,963 | No | ns | >0,9999 | G-I |
| **L.la vs. Control** | 18,05 | 10,26 to 25,84 | Yes | **** | <0,0001 | G-J |
| **L.la vs. LPS** | -41,86 | -49,65 to -34,07 | Yes | **** | <0,0001 | G-K |
| **L.la vs. S.a** | 5,096 | -2,696 to 12,89 | No | ns | 0,7338 | G-L |
| **L.la vs. L.g** | -9,992 | -17,34 to -2,646 | Yes | *** | 0,0004 | G-M |
| **L.la vs. L.s** | -14,36 | -21,70 to -7,010 | Yes | **** | <0,0001 | G-N |
| **L.la vs. L.la** | -32,66 | -40,01 to -25,32 | Yes | **** | <0,0001 | G-O |
| **L.la vs. L.b** | -10,63 | -17,97 to -3,280 | Yes | **** | <0,0001 | G-P |
| **L.la vs. L.n** | -10,59 | -17,93 to -3,242 | Yes | *** | 0,0001 | G-Q |
| **L.la vs. L.li** | -13,79 | -21,14 to -6,448 | Yes | **** | <0,0001 | G-R |
| **L.la vs. Control** | 17,94 | 10,15 to 25,73 | Yes | **** | <0,0001 | G-S |
| **L.la vs. LPS** | 3,782 | -4,010 to 11,57 | No | ns | 0,9835 | G-T |
| **L.la vs. S.a** | -28,07 | -35,86 to -20,28 | Yes | **** | <0,0001 | G-U |
| **L.la vs. L.g** | 1,774 | -5,572 to 9,120 | No | ns | >0,9999 | G-V |
| **L.la vs. L.s** | -3,286 | -10,63 to 4,060 | No | ns | 0,9943 | G-W |
| **L.la vs. L.la** | 0,000 | -7,346 to 7,346 | No | ns | >0,9999 | G-X |
| **L.la vs. L.b** | 0,4409 | -6,905 to 7,787 | No | ns | >0,9999 | G-Y |
| **L.la vs. L.n** | 0,6172 | -6,729 to 7,963 | No | ns | >0,9999 | G-Z |
| **L.la vs. L.li** | 0,6947 | -6,651 to 8,041 | No | ns | >0,9999 | G-AA |
| **L.li vs. L.n** | -0,07742 | -7,423 to 7,269 | No | ns | >0,9999 | H-I |
| **L.li vs. Control** | 17,35 | 9,560 to 25,14 | Yes | **** | <0,0001 | H-J |
| **L.li vs. LPS** | -42,55 | -50,34 to -34,76 | Yes | **** | <0,0001 | H-K |
| **L.li vs. S.a** | 4,401 | -3,391 to 12,19 | No | ns | 0,9156 | H-L |
| **L.li vs. L.g** | -10,69 | -18,03 to -3,341 | Yes | **** | <0,0001 | H-M |
| **L.li vs. L.s** | -15,05 | -22,40 to -7,704 | Yes | **** | <0,0001 | H-N |
| **L.li vs. L.la** | -33,36 | -40,70 to -26,01 | Yes | **** | <0,0001 | H-O |
| **L.li vs. L.b** | -11,32 | -18,67 to -3,975 | Yes | **** | <0,0001 | H-P |
| **L.li vs. L.n** | -11,28 | -18,63 to -3,936 | Yes | **** | <0,0001 | H-Q |
| **L.li vs. L.li** | -14,49 | -21,84 to -7,143 | Yes | **** | <0,0001 | H-R |
| **L.li vs. Control** | 17,24 | 9,453 to 25,04 | Yes | **** | <0,0001 | H-S |
| **L.li vs. LPS** | 3,087 | -4,704 to 10,88 | No | ns | 0,9990 | H-T |
| **L.li vs. S.a** | -28,77 | -36,56 to -20,97 | Yes | **** | <0,0001 | H-U |
| **L.li vs. L.g** | 1,080 | -6,266 to 8,426 | No | ns | >0,9999 | H-V |
| **L.li vs. L.s** | -3,981 | -11,33 to 3,365 | No | ns | 0,9435 | H-W |
| **L.li vs. L.la** | -0,6947 | -8,041 to 6,651 | No | ns | >0,9999 | H-X |
| **L.li vs. L.b** | -0,2538 | -7,600 to 7,092 | No | ns | >0,9999 | H-Y |
| **L.li vs. L.n** | -0,07742 | -7,423 to 7,269 | No | ns | >0,9999 | H-Z |
| **L.li vs. L.li** | 0,000 | -7,346 to 7,346 | No | ns | >0,9999 | H-AA |
| **L.n vs. Control** | 17,43 | 9,638 to 25,22 | Yes | **** | <0,0001 | I-J |
| **L.n vs. LPS** | -42,47 | -50,27 to -34,68 | Yes | **** | <0,0001 | I-K |
| **L.n vs. S.a** | 4,479 | -3,313 to 12,27 | No | ns | 0,9010 | I-L |
| **L.n vs. L.g** | -10,61 | -17,96 to -3,263 | Yes | **** | <0,0001 | I-M |
| **L.n vs. L.s** | -14,97 | -22,32 to -7,627 | Yes | **** | <0,0001 | I-N |
| **L.n vs. L.la** | -33,28 | -40,63 to -25,93 | Yes | **** | <0,0001 | I-O |
| **L.n vs. L.b** | -11,24 | -18,59 to -3,898 | Yes | **** | <0,0001 | I-P |
| **L.n vs. L.n** | -11,21 | -18,55 to -3,859 | Yes | **** | <0,0001 | I-Q |
| **L.n vs. L.li** | -14,41 | -21,76 to -7,066 | Yes | **** | <0,0001 | I-R |
| **L.n vs. Control** | 17,32 | 9,530 to 25,11 | Yes | **** | <0,0001 | I-S |
| **L.n vs. LPS** | 3,165 | -4,627 to 10,96 | No | ns | 0,9986 | I-T |
| **L.n vs. S.a** | -28,69 | -36,48 to -20,90 | Yes | **** | <0,0001 | I-U |
| **L.n vs. L.g** | 1,157 | -6,189 to 8,503 | No | ns | >0,9999 | I-V |
| **L.n vs. L.s** | -3,903 | -11,25 to 3,443 | No | ns | 0,9539 | I-W |
| **L.n vs. L.la** | -0,6172 | -7,963 to 6,729 | No | ns | >0,9999 | I-X |
| **L.n vs. L.b** | -0,1764 | -7,522 to 7,170 | No | ns | >0,9999 | I-Y |
| **L.n vs. L.n** | 0,000 | -7,346 to 7,346 | No | ns | >0,9999 | I-Z |
| **L.n vs. L.li** | 0,07742 | -7,269 to 7,423 | No | ns | >0,9999 | I-AA |
| **Control vs. LPS** | -59,90 | -68,12 to -51,69 | Yes | **** | <0,0001 | J-K |
| **Control vs. S.a** | -12,95 | -21,16 to -4,738 | Yes | **** | <0,0001 | J-L |
| **Control vs. L.g** | -28,04 | -35,83 to -20,25 | Yes | **** | <0,0001 | J-M |
| **Control vs. L.s** | -32,40 | -40,19 to -24,61 | Yes | **** | <0,0001 | J-N |
| **Control vs. L.la** | -50,71 | -58,50 to -42,92 | Yes | **** | <0,0001 | J-O |
| **Control vs. L.b** | -28,67 | -36,46 to -20,88 | Yes | **** | <0,0001 | J-P |
| **Control vs. L.n** | -28,63 | -36,43 to -20,84 | Yes | **** | <0,0001 | J-Q |
| **Control vs. L.li** | -31,84 | -39,63 to -24,05 | Yes | **** | <0,0001 | J-R |
| **Control vs. Control** | -0,1074 | -8,321 to 8,106 | No | ns | >0,9999 | J-S |
| **Control vs. LPS** | -14,26 | -22,48 to -6,052 | Yes | **** | <0,0001 | J-T |
| **Control vs. S.a** | -46,12 | -54,33 to -37,91 | Yes | **** | <0,0001 | J-U |
| **Control vs. L.g** | -16,27 | -24,06 to -8,481 | Yes | **** | <0,0001 | J-V |
| **Control vs. L.s** | -21,33 | -29,12 to -13,54 | Yes | **** | <0,0001 | J-W |
| **Control vs. L.la** | -18,05 | -25,84 to -10,26 | Yes | **** | <0,0001 | J-X |
| **Control vs. L.b** | -17,61 | -25,40 to -9,814 | Yes | **** | <0,0001 | J-Y |
| **Control vs. L.n** | -17,43 | -25,22 to -9,638 | Yes | **** | <0,0001 | J-Z |
| **Control vs. L.li** | -17,35 | -25,14 to -9,560 | Yes | **** | <0,0001 | J-AA |
| **LPS vs. S.a** | 46,95 | 38,74 to 55,17 | Yes | **** | <0,0001 | K-L |
| **LPS vs. L.g** | 31,87 | 24,07 to 39,66 | Yes | **** | <0,0001 | K-M |
| **LPS vs. L.s** | 27,50 | 19,71 to 35,29 | Yes | **** | <0,0001 | K-N |
| **LPS vs. L.la** | 9,195 | 1,404 to 16,99 | Yes | ** | 0,0051 | K-O |
| **LPS vs. L.b** | 31,23 | 23,44 to 39,02 | Yes | **** | <0,0001 | K-P |
| **LPS vs. L.n** | 31,27 | 23,48 to 39,06 | Yes | **** | <0,0001 | K-Q |
| **LPS vs. L.li** | 28,06 | 20,27 to 35,85 | Yes | **** | <0,0001 | K-R |
| **LPS vs. Control** | 59,80 | 51,58 to 68,01 | Yes | **** | <0,0001 | K-S |
| **LPS vs. LPS** | 45,64 | 37,43 to 53,85 | Yes | **** | <0,0001 | K-T |
| **LPS vs. S.a** | 13,79 | 5,573 to 22,00 | Yes | **** | <0,0001 | K-U |
| **LPS vs. L.g** | 43,63 | 35,84 to 51,42 | Yes | **** | <0,0001 | K-V |
| **LPS vs. L.s** | 38,57 | 30,78 to 46,36 | Yes | **** | <0,0001 | K-W |
| **LPS vs. L.la** | 41,86 | 34,07 to 49,65 | Yes | **** | <0,0001 | K-X |
| **LPS vs. L.b** | 42,30 | 34,51 to 50,09 | Yes | **** | <0,0001 | K-Y |
| **LPS vs. L.n** | 42,47 | 34,68 to 50,27 | Yes | **** | <0,0001 | K-Z |
| **LPS vs. L.li** | 42,55 | 34,76 to 50,34 | Yes | **** | <0,0001 | K-AA |
| **S.a vs. L.g** | -15,09 | -22,88 to -7,296 | Yes | **** | <0,0001 | L-M |
| **S.a vs. L.s** | -19,45 | -27,24 to -11,66 | Yes | **** | <0,0001 | L-N |
| **S.a vs. L.la** | -37,76 | -45,55 to -29,97 | Yes | **** | <0,0001 | L-O |
| **S.a vs. L.b** | -15,72 | -23,51 to -7,931 | Yes | **** | <0,0001 | L-P |
| **S.a vs. L.n** | -15,68 | -23,48 to -7,892 | Yes | **** | <0,0001 | L-Q |
| **S.a vs. L.li** | -18,89 | -26,68 to -11,10 | Yes | **** | <0,0001 | L-R |
| **S.a vs. Control** | 12,84 | 4,630 to 21,06 | Yes | **** | <0,0001 | L-S |
| **S.a vs. LPS** | -1,314 | -9,527 to 6,899 | No | ns | >0,9999 | L-T |
| **S.a vs. S.a** | -33,17 | -41,38 to -24,95 | Yes | **** | <0,0001 | L-U |
| **S.a vs. L.g** | -3,322 | -11,11 to 4,470 | No | ns | 0,9971 | L-V |
| **S.a vs. L.s** | -8,382 | -16,17 to -0,5904 | Yes | * | 0,0203 | L-W |
| **S.a vs. L.la** | -5,096 | -12,89 to 2,696 | No | ns | 0,7338 | L-X |
| **S.a vs. L.b** | -4,655 | -12,45 to 3,137 | No | ns | 0,8623 | L-Y |
| **S.a vs. L.n** | -4,479 | -12,27 to 3,313 | No | ns | 0,9010 | L-Z |
| **S.a vs. L.li** | -4,401 | -12,19 to 3,391 | No | ns | 0,9156 | L-AA |
| **L.g vs. L.s** | -4,364 | -11,71 to 2,982 | No | ns | 0,8686 | M-N |
| **L.g vs. L.la** | -22,67 | -30,02 to -15,32 | Yes | **** | <0,0001 | M-O |
| **L.g vs. L.b** | -0,6344 | -7,981 to 6,712 | No | ns | >0,9999 | M-P |
| **L.g vs. L.n** | -0,5957 | -7,942 to 6,750 | No | ns | >0,9999 | M-Q |
| **L.g vs. L.li** | -3,802 | -11,15 to 3,544 | No | ns | 0,9652 | M-R |
| **L.g vs. Control** | 27,93 | 20,14 to 35,72 | Yes | **** | <0,0001 | M-S |
| **L.g vs. LPS** | 13,77 | 5,982 to 21,57 | Yes | **** | <0,0001 | M-T |
| **L.g vs. S.a** | -18,08 | -25,87 to -10,29 | Yes | **** | <0,0001 | M-U |
| **L.g vs. L.g** | 11,77 | 4,420 to 19,11 | Yes | **** | <0,0001 | M-V |
| **L.g vs. L.s** | 6,706 | -0,6403 to 14,05 | No | ns | 0,1257 | M-W |
| **L.g vs. L.la** | 9,992 | 2,646 to 17,34 | Yes | *** | 0,0004 | M-X |
| **L.g vs. L.b** | 10,43 | 3,087 to 17,78 | Yes | *** | 0,0001 | M-Y |
| **L.g vs. L.n** | 10,61 | 3,263 to 17,96 | Yes | **** | <0,0001 | M-Z |
| **L.g vs. L.li** | 10,69 | 3,341 to 18,03 | Yes | **** | <0,0001 | M-AA |
| **L.s vs. L.la** | -18,31 | -25,65 to -10,96 | Yes | **** | <0,0001 | N-O |
| **L.s vs. L.b** | 3,729 | -3,617 to 11,08 | No | ns | 0,9720 | N-P |
| **L.s vs. L.n** | 3,768 | -3,578 to 11,11 | No | ns | 0,9685 | N-Q |
| **L.s vs. L.li** | 0,5613 | -6,785 to 7,907 | No | ns | >0,9999 | N-R |
| **L.s vs. Control** | 32,30 | 24,50 to 40,09 | Yes | **** | <0,0001 | N-S |
| **L.s vs. LPS** | 18,14 | 10,35 to 25,93 | Yes | **** | <0,0001 | N-T |
| **L.s vs. S.a** | -13,72 | -21,51 to -5,924 | Yes | **** | <0,0001 | N-U |
| **L.s vs. L.g** | 16,13 | 8,784 to 23,48 | Yes | **** | <0,0001 | N-V |
| **L.s vs. L.s** | 11,07 | 3,723 to 18,42 | Yes | **** | <0,0001 | N-W |
| **L.s vs. L.la** | 14,36 | 7,010 to 21,70 | Yes | **** | <0,0001 | N-X |
| **L.s vs. L.b** | 14,80 | 7,451 to 22,14 | Yes | **** | <0,0001 | N-Y |
| **L.s vs. L.n** | 14,97 | 7,627 to 22,32 | Yes | **** | <0,0001 | N-Z |
| **L.s vs. L.li** | 15,05 | 7,704 to 22,40 | Yes | **** | <0,0001 | N-AA |
| **L.la vs. L.b** | 22,04 | 14,69 to 29,38 | Yes | **** | <0,0001 | O-P |
| **L.la vs. L.n** | 22,07 | 14,73 to 29,42 | Yes | **** | <0,0001 | O-Q |
| **L.la vs. L.li** | 18,87 | 11,52 to 26,21 | Yes | **** | <0,0001 | O-R |
| **L.la vs. Control** | 50,60 | 42,81 to 58,39 | Yes | **** | <0,0001 | O-S |
| **L.la vs. LPS** | 36,44 | 28,65 to 44,24 | Yes | **** | <0,0001 | O-T |
| **L.la vs. S.a** | 4,591 | -3,201 to 12,38 | No | ns | 0,8773 | O-U |
| **L.la vs. L.g** | 34,44 | 27,09 to 41,78 | Yes | **** | <0,0001 | O-V |
| **L.la vs. L.s** | 29,38 | 22,03 to 36,72 | Yes | **** | <0,0001 | O-W |
| **L.la vs. L.la** | 32,66 | 25,32 to 40,01 | Yes | **** | <0,0001 | O-X |
| **L.la vs. L.b** | 33,10 | 25,76 to 40,45 | Yes | **** | <0,0001 | O-Y |
| **L.la vs. L.n** | 33,28 | 25,93 to 40,63 | Yes | **** | <0,0001 | O-Z |
| **L.la vs. L.li** | 33,36 | 26,01 to 40,70 | Yes | **** | <0,0001 | O-AA |
| **L.b vs. L.n** | 0,03871 | -7,307 to 7,385 | No | ns | >0,9999 | P-Q |
| **L.b vs. L.li** | -3,168 | -10,51 to 4,178 | No | ns | 0,9966 | P-R |
| **L.b vs. Control** | 28,57 | 20,77 to 36,36 | Yes | **** | <0,0001 | P-S |
| **L.b vs. LPS** | 14,41 | 6,617 to 22,20 | Yes | **** | <0,0001 | P-T |
| **L.b vs. S.a** | -17,45 | -25,24 to -9,654 | Yes | **** | <0,0001 | P-U |
| **L.b vs. L.g** | 12,40 | 5,055 to 19,75 | Yes | **** | <0,0001 | P-V |
| **L.b vs. L.s** | 7,340 | -0,005822 to 14,69 | No | ns | 0,0505 | P-W |
| **L.b vs. L.la** | 10,63 | 3,280 to 17,97 | Yes | **** | <0,0001 | P-X |
| **L.b vs. L.b** | 11,07 | 3,721 to 18,41 | Yes | **** | <0,0001 | P-Y |
| **L.b vs. L.n** | 11,24 | 3,898 to 18,59 | Yes | **** | <0,0001 | P-Z |
| **L.b vs. L.li** | 11,32 | 3,975 to 18,67 | Yes | **** | <0,0001 | P-AA |
| **L.n vs. L.li** | -3,207 | -10,55 to 4,139 | No | ns | 0,9959 | Q-R |
| **L.n vs. Control** | 28,53 | 20,74 to 36,32 | Yes | **** | <0,0001 | Q-S |
| **L.n vs. LPS** | 14,37 | 6,578 to 22,16 | Yes | **** | <0,0001 | Q-T |
| **L.n vs. S.a** | -17,48 | -25,28 to -9,692 | Yes | **** | <0,0001 | Q-U |
| **L.n vs. L.g** | 12,36 | 5,016 to 19,71 | Yes | **** | <0,0001 | Q-V |
| **L.n vs. L.s** | 7,302 | -0,04454 to 14,65 | No | ns | 0,0535 | Q-W |
| **L.n vs. L.la** | 10,59 | 3,242 to 17,93 | Yes | *** | 0,0001 | Q-X |
| **L.n vs. L.b** | 11,03 | 3,683 to 18,37 | Yes | **** | <0,0001 | Q-Y |
| **L.n vs. L.n** | 11,21 | 3,859 to 18,55 | Yes | **** | <0,0001 | Q-Z |
| **L.n vs. L.li** | 11,28 | 3,936 to 18,63 | Yes | **** | <0,0001 | Q-AA |
| **L.li vs. Control** | 31,73 | 23,94 to 39,53 | Yes | **** | <0,0001 | R-S |
| **L.li vs. LPS** | 17,58 | 9,785 to 25,37 | Yes | **** | <0,0001 | R-T |
| **L.li vs. S.a** | -14,28 | -22,07 to -6,486 | Yes | **** | <0,0001 | R-U |
| **L.li vs. L.g** | 15,57 | 8,223 to 22,91 | Yes | **** | <0,0001 | R-V |
| **L.li vs. L.s** | 10,51 | 3,162 to 17,85 | Yes | *** | 0,0001 | R-W |
| **L.li vs. L.la** | 13,79 | 6,448 to 21,14 | Yes | **** | <0,0001 | R-X |
| **L.li vs. L.b** | 14,24 | 6,889 to 21,58 | Yes | **** | <0,0001 | R-Y |
| **L.li vs. L.n** | 14,41 | 7,066 to 21,76 | Yes | **** | <0,0001 | R-Z |
| **L.li vs. L.li** | 14,49 | 7,143 to 21,84 | Yes | **** | <0,0001 | R-AA |
| **Control vs. LPS** | -14,16 | -22,37 to -5,944 | Yes | **** | <0,0001 | S-T |
| **Control vs. S.a** | -46,01 | -54,22 to -37,80 | Yes | **** | <0,0001 | S-U |
| **Control vs. L.g** | -16,17 | -23,96 to -8,373 | Yes | **** | <0,0001 | S-V |
| **Control vs. L.s** | -21,23 | -29,02 to -13,43 | Yes | **** | <0,0001 | S-W |
| **Control vs. L.la** | -17,94 | -25,73 to -10,15 | Yes | **** | <0,0001 | S-X |
| **Control vs. L.b** | -17,50 | -25,29 to -9,707 | Yes | **** | <0,0001 | S-Y |
| **Control vs. L.n** | -17,32 | -25,11 to -9,530 | Yes | **** | <0,0001 | S-Z |
| **Control vs. L.li** | -17,24 | -25,04 to -9,453 | Yes | **** | <0,0001 | S-AA |
| **LPS vs. S.a** | -31,85 | -40,07 to -23,64 | Yes | **** | <0,0001 | T-U |
| **LPS vs. L.g** | -2,008 | -9,799 to 5,784 | No | ns | >0,9999 | T-V |
| **LPS vs. L.s** | -7,068 | -14,86 to 0,7236 | No | ns | 0,1329 | T-W |
| **LPS vs. L.la** | -3,782 | -11,57 to 4,010 | No | ns | 0,9835 | T-X |
| **LPS vs. L.b** | -3,341 | -11,13 to 4,451 | No | ns | 0,9968 | T-Y |
| **LPS vs. L.n** | -3,165 | -10,96 to 4,627 | No | ns | 0,9986 | T-Z |
| **LPS vs. L.li** | -3,087 | -10,88 to 4,704 | No | ns | 0,9990 | T-AA |
| **S.a vs. L.g** | 29,85 | 22,05 to 37,64 | Yes | **** | <0,0001 | U-V |
| **S.a vs. L.s** | 24,79 | 16,99 to 32,58 | Yes | **** | <0,0001 | U-W |
| **S.a vs. L.la** | 28,07 | 20,28 to 35,86 | Yes | **** | <0,0001 | U-X |
| **S.a vs. L.b** | 28,51 | 20,72 to 36,30 | Yes | **** | <0,0001 | U-Y |
| **S.a vs. L.n** | 28,69 | 20,90 to 36,48 | Yes | **** | <0,0001 | U-Z |
| **S.a vs. L.li** | 28,77 | 20,97 to 36,56 | Yes | **** | <0,0001 | U-AA |
| **L.g vs. L.s** | -5,061 | -12,41 to 2,286 | No | ns | 0,6381 | V-W |
| **L.g vs. L.la** | -1,774 | -9,120 to 5,572 | No | ns | >0,9999 | V-X |
| **L.g vs. L.b** | -1,333 | -8,679 to 6,013 | No | ns | >0,9999 | V-Y |
| **L.g vs. L.n** | -1,157 | -8,503 to 6,189 | No | ns | >0,9999 | V-Z |
| **L.g vs. L.li** | -1,080 | -8,426 to 6,266 | No | ns | >0,9999 | V-AA |
| **L.s vs. L.la** | 3,286 | -4,060 to 10,63 | No | ns | 0,9943 | W-X |
| **L.s vs. L.b** | 3,727 | -3,619 to 11,07 | No | ns | 0,9722 | W-Y |
| **L.s vs. L.n** | 3,903 | -3,443 to 11,25 | No | ns | 0,9539 | W-Z |
| **L.s vs. L.li** | 3,981 | -3,365 to 11,33 | No | ns | 0,9435 | W-AA |
| **L.la vs. L.b** | 0,4409 | -6,905 to 7,787 | No | ns | >0,9999 | X-Y |
| **L.la vs. L.n** | 0,6172 | -6,729 to 7,963 | No | ns | >0,9999 | X-Z |
| **L.la vs. L.li** | 0,6947 | -6,651 to 8,041 | No | ns | >0,9999 | X-AA |
| **L.b vs. L.n** | 0,1764 | -7,170 to 7,522 | No | ns | >0,9999 | Y-Z |
| **L.b vs. L.li** | 0,2538 | -7,092 to 7,600 | No | ns | >0,9999 | Y-AA |
| **L.n vs. L.li** | 0,07742 | -7,269 to 7,423 | No | ns | >0,9999 | Z-AA |

**Raw data and statistical analysis of Figure 13:**

**TNF-α (pg/mL)**

**LPG (WT, TLR2KO and TLR4KO)**

| Control | LPS | S.a | L.g | L.s | L.la | L.b | L.n | L.li |
| --- | --- | --- | --- | --- | --- | --- | --- | --- |
| 13,6 | 7053,4 | 5693,1 | 110,9 | 554,4 | 4898 | 72,2 | 12367 | 69858 |
| 10,8 | 8277,4 | 5234,6 | 108,1 | 550,9 | 6052 | 73,4 | 10285 | 87513 |
| 10,2 | 5685,6 | 5015,9 | 100 | 668,7 | 6427 | 54,9 | 11615 | 1017 |
| 9,6 | 6958,1 | 6143,6 | 176,4 | 709,9 | 6709 | 81,5 | 10805 | 79748 |
|  |  |  | 164,8 | 736,9 | 4297 | 100,5 | 9822 | 68722 |
| Control | LPS | S.a | L.g | L.s | L.la | L.b | L.n | L.li |
| 7 | 3187 | 267 | 216,77 | 244,11 | 8051 | 317,77 | 128,21 | 78,78 |
| 4 | 3522 | 100 | 244,11 | 866,47 | 9303 | 141,13 | 299,29 | 98,86 |
| 6 | 4272 | 239 | 331,65 | 331,65 | 5503 | 111,28 | 216,77 | 171,85 |
| 4 | 4263 | 119 | 221,31 | 185,22 | 6410 | 145,47 | 493,47 | 59,71 |
|  |  |  | 185,22 | 145,47 | 5670 | 90,72 | 194,19 | 189,7 |
| Control | LPS | S.a | L.g | L.s | L.la | L.b | L.n | L.li |
| 7,39 | 200 | 7903,47 | 31,91 | 551,46 | 391,99 | 48,02 | 51,47 | 1,736 |
| 8,51 | 170 | 5464,4 | 46,87 | 434,38 | 274,22 | 47,44 | 45,71 | 1,519 |
| 4,05 | 108 | 6518,25 | 60,7 | 284,15 | 224,67 | 50,9 | 44,56 | 1,79 |
| 7,39 | 190 | 8002,28 | 95,33 | 353,82 | 253,8 | 56,08 | 53,2 | 1,317 |
|  |  |  | 35,93 | 282,98 | 271,89 | 61,85 | 53,2 | 1,283 |

**Statistical analysis**

| **Tukey's multiple comparisons test** | **Mean Diff,** | **95,00% CI of diff,** | **Significant?** | **Summary** | **Adjusted P Value** |  |
| --- | --- | --- | --- | --- | --- | --- |
| **Control vs. LPS** | -6983 | -25758 to 11793 | No | ns | 0,9997 | A-B |
| **Control vs. S.a** | -5511 | -24286 to 13265 | No | ns | >0,9999 | A-C |
| **Control vs. L.g** | -121,0 | -17933 to 17691 | No | ns | >0,9999 | A-D |
| **Control vs. L.s** | -633,1 | -18445 to 17179 | No | ns | >0,9999 | A-E |
| **Control vs. L.la** | -5666 | -23478 to 12146 | No | ns | >0,9999 | A-F |
| **Control vs. L.b** | -65,45 | -17877 to 17747 | No | ns | >0,9999 | A-G |
| **Control vs. L.n** | -10968 | -28780 to 6844 | No | ns | 0,8253 | A-H |
| **Control vs. L.li** | -61361 | -79173 to -43549 | Yes | **** | <0,0001 | A-I |
| **Control vs. Control** | 5,800 | -18770 to 18781 | No | ns | >0,9999 | A-J |
| **Control vs. LPS** | -3800 | -22575 to 14976 | No | ns | >0,9999 | A-K |
| **Control vs. S.a** | -170,2 | -18946 to 18605 | No | ns | >0,9999 | A-L |
| **Control vs. L.g** | -228,8 | -18041 to 17583 | No | ns | >0,9999 | A-M |
| **Control vs. L.s** | -343,5 | -18156 to 17468 | No | ns | >0,9999 | A-N |
| **Control vs. L.la** | -6976 | -24788 to 10836 | No | ns | 0,9992 | A-O |
| **Control vs. L.b** | -150,2 | -17962 to 17662 | No | ns | >0,9999 | A-P |
| **Control vs. L.n** | -255,3 | -18067 to 17557 | No | ns | >0,9999 | A-Q |
| **Control vs. L.li** | -108,7 | -17921 to 17703 | No | ns | >0,9999 | A-R |
| **Control vs. Control** | 4,215 | -18771 to 18780 | No | ns | >0,9999 | A-S |
| **Control vs. LPS** | -156,0 | -18931 to 18620 | No | ns | >0,9999 | A-T |
| **Control vs. S.a** | -6961 | -25737 to 11814 | No | ns | 0,9997 | A-U |
| **Control vs. L.g** | -43,10 | -17855 to 17769 | No | ns | >0,9999 | A-V |
| **Control vs. L.s** | -370,3 | -18182 to 17442 | No | ns | >0,9999 | A-W |
| **Control vs. L.la** | -272,3 | -18084 to 17540 | No | ns | >0,9999 | A-X |
| **Control vs. L.b** | -41,81 | -17854 to 17770 | No | ns | >0,9999 | A-Y |
| **Control vs. L.n** | -38,58 | -17851 to 17773 | No | ns | >0,9999 | A-Z |
| **Control vs. L.li** | 9,521 | -17803 to 17822 | No | ns | >0,9999 | A-AA |
| **LPS vs. S.a** | 1472 | -17304 to 20247 | No | ns | >0,9999 | B-C |
| **LPS vs. L.g** | 6862 | -10950 to 24674 | No | ns | 0,9994 | B-D |
| **LPS vs. L.s** | 6349 | -11463 to 24161 | No | ns | 0,9998 | B-E |
| **LPS vs. L.la** | 1317 | -16495 to 19129 | No | ns | >0,9999 | B-F |
| **LPS vs. L.b** | 6917 | -10895 to 24729 | No | ns | 0,9993 | B-G |
| **LPS vs. L.n** | -3985 | -21797 to 13827 | No | ns | >0,9999 | B-H |
| **LPS vs. L.li** | -54378 | -72190 to -36566 | Yes | **** | <0,0001 | B-I |
| **LPS vs. Control** | 6988 | -11787 to 25764 | No | ns | 0,9997 | B-J |
| **LPS vs. LPS** | 3183 | -15593 to 21958 | No | ns | >0,9999 | B-K |
| **LPS vs. S.a** | 6812 | -11963 to 25588 | No | ns | 0,9998 | B-L |
| **LPS vs. L.g** | 6754 | -11058 to 24566 | No | ns | 0,9995 | B-M |
| **LPS vs. L.s** | 6639 | -11173 to 24451 | No | ns | 0,9996 | B-N |
| **LPS vs. L.la** | 6,225 | -17806 to 17818 | No | ns | >0,9999 | B-O |
| **LPS vs. L.b** | 6832 | -10980 to 24644 | No | ns | 0,9994 | B-P |
| **LPS vs. L.n** | 6727 | -11085 to 24539 | No | ns | 0,9996 | B-Q |
| **LPS vs. L.li** | 6874 | -10938 to 24686 | No | ns | 0,9994 | B-R |
| **LPS vs. Control** | 6987 | -11789 to 25762 | No | ns | 0,9997 | B-S |
| **LPS vs. LPS** | 6827 | -11949 to 25602 | No | ns | 0,9998 | B-T |
| **LPS vs. S.a** | 21,52 | -18754 to 18797 | No | ns | >0,9999 | B-U |
| **LPS vs. L.g** | 6939 | -10873 to 24751 | No | ns | 0,9993 | B-V |
| **LPS vs. L.s** | 6612 | -11200 to 24424 | No | ns | 0,9997 | B-W |
| **LPS vs. L.la** | 6710 | -11102 to 24522 | No | ns | 0,9996 | B-X |
| **LPS vs. L.b** | 6941 | -10871 to 24753 | No | ns | 0,9993 | B-Y |
| **LPS vs. L.n** | 6944 | -10868 to 24756 | No | ns | 0,9993 | B-Z |
| **LPS vs. L.li** | 6992 | -10820 to 24804 | No | ns | 0,9992 | B-AA |
| **S.a vs. L.g** | 5390 | -12422 to 23202 | No | ns | >0,9999 | C-D |
| **S.a vs. L.s** | 4878 | -12934 to 22690 | No | ns | >0,9999 | C-E |
| **S.a vs. L.la** | -154,8 | -17967 to 17657 | No | ns | >0,9999 | C-F |
| **S.a vs. L.b** | 5445 | -12367 to 23257 | No | ns | >0,9999 | C-G |
| **S.a vs. L.n** | -5457 | -23269 to 12355 | No | ns | >0,9999 | C-H |
| **S.a vs. L.li** | -55850 | -73662 to -38038 | Yes | **** | <0,0001 | C-I |
| **S.a vs. Control** | 5517 | -13259 to 24292 | No | ns | >0,9999 | C-J |
| **S.a vs. LPS** | 1711 | -17065 to 20486 | No | ns | >0,9999 | C-K |
| **S.a vs. S.a** | 5341 | -13435 to 24116 | No | ns | >0,9999 | C-L |
| **S.a vs. L.g** | 5282 | -12530 to 23094 | No | ns | >0,9999 | C-M |
| **S.a vs. L.s** | 5167 | -12645 to 22979 | No | ns | >0,9999 | C-N |
| **S.a vs. L.la** | -1466 | -19278 to 16346 | No | ns | >0,9999 | C-O |
| **S.a vs. L.b** | 5361 | -12451 to 23173 | No | ns | >0,9999 | C-P |
| **S.a vs. L.n** | 5255 | -12557 to 23067 | No | ns | >0,9999 | C-Q |
| **S.a vs. L.li** | 5402 | -12410 to 23214 | No | ns | >0,9999 | C-R |
| **S.a vs. Control** | 5515 | -13261 to 24290 | No | ns | >0,9999 | C-S |
| **S.a vs. LPS** | 5355 | -13421 to 24130 | No | ns | >0,9999 | C-T |
| **S.a vs. S.a** | -1450 | -20226 to 17325 | No | ns | >0,9999 | C-U |
| **S.a vs. L.g** | 5468 | -12344 to 23280 | No | ns | >0,9999 | C-V |
| **S.a vs. L.s** | 5140 | -12672 to 22952 | No | ns | >0,9999 | C-W |
| **S.a vs. L.la** | 5238 | -12574 to 23051 | No | ns | >0,9999 | C-X |
| **S.a vs. L.b** | 5469 | -12343 to 23281 | No | ns | >0,9999 | C-Y |
| **S.a vs. L.n** | 5472 | -12340 to 23284 | No | ns | >0,9999 | C-Z |
| **S.a vs. L.li** | 5520 | -12292 to 23332 | No | ns | >0,9999 | C-AA |
| **L.g vs. L.s** | -512,1 | -17305 to 16281 | No | ns | >0,9999 | D-E |
| **L.g vs. L.la** | -5545 | -22338 to 11249 | No | ns | >0,9999 | D-F |
| **L.g vs. L.b** | 55,54 | -16738 to 16849 | No | ns | >0,9999 | D-G |
| **L.g vs. L.n** | -10847 | -27640 to 5947 | No | ns | 0,7546 | D-H |
| **L.g vs. L.li** | -61240 | -78033 to -44446 | Yes | **** | <0,0001 | D-I |
| **L.g vs. Control** | 126,8 | -17685 to 17939 | No | ns | >0,9999 | D-J |
| **L.g vs. LPS** | -3679 | -21491 to 14133 | No | ns | >0,9999 | D-K |
| **L.g vs. S.a** | -49,21 | -17861 to 17763 | No | ns | >0,9999 | D-L |
| **L.g vs. L.g** | -107,8 | -16901 to 16686 | No | ns | >0,9999 | D-M |
| **L.g vs. L.s** | -222,5 | -17016 to 16571 | No | ns | >0,9999 | D-N |
| **L.g vs. L.la** | -6855 | -23649 to 9938 | No | ns | 0,9985 | D-O |
| **L.g vs. L.b** | -29,23 | -16823 to 16764 | No | ns | >0,9999 | D-P |
| **L.g vs. L.n** | -134,3 | -16928 to 16659 | No | ns | >0,9999 | D-Q |
| **L.g vs. L.li** | 12,26 | -16781 to 16806 | No | ns | >0,9999 | D-R |
| **L.g vs. Control** | 125,2 | -17687 to 17937 | No | ns | >0,9999 | D-S |
| **L.g vs. LPS** | -34,96 | -17847 to 17777 | No | ns | >0,9999 | D-T |
| **L.g vs. S.a** | -6840 | -24652 to 10972 | No | ns | 0,9994 | D-U |
| **L.g vs. L.g** | 77,89 | -16715 to 16871 | No | ns | >0,9999 | D-V |
| **L.g vs. L.s** | -249,3 | -17043 to 16544 | No | ns | >0,9999 | D-W |
| **L.g vs. L.la** | -151,3 | -16945 to 16642 | No | ns | >0,9999 | D-X |
| **L.g vs. L.b** | 79,18 | -16714 to 16873 | No | ns | >0,9999 | D-Y |
| **L.g vs. L.n** | 82,41 | -16711 to 16876 | No | ns | >0,9999 | D-Z |
| **L.g vs. L.li** | 130,5 | -16663 to 16924 | No | ns | >0,9999 | D-AA |
| **L.s vs. L.la** | -5032 | -21826 to 11761 | No | ns | >0,9999 | E-F |
| **L.s vs. L.b** | 567,7 | -16226 to 17361 | No | ns | >0,9999 | E-G |
| **L.s vs. L.n** | -10335 | -27128 to 6459 | No | ns | 0,8260 | E-H |
| **L.s vs. L.li** | -60727 | -77521 to -43934 | Yes | **** | <0,0001 | E-I |
| **L.s vs. Control** | 638,9 | -17173 to 18451 | No | ns | >0,9999 | E-J |
| **L.s vs. LPS** | -3167 | -20979 to 14645 | No | ns | >0,9999 | E-K |
| **L.s vs. S.a** | 462,9 | -17349 to 18275 | No | ns | >0,9999 | E-L |
| **L.s vs. L.g** | 404,3 | -16389 to 17198 | No | ns | >0,9999 | E-M |
| **L.s vs. L.s** | 289,6 | -16504 to 17083 | No | ns | >0,9999 | E-N |
| **L.s vs. L.la** | -6343 | -23137 to 10450 | No | ns | 0,9996 | E-O |
| **L.s vs. L.b** | 482,9 | -16310 to 17276 | No | ns | >0,9999 | E-P |
| **L.s vs. L.n** | 377,8 | -16416 to 17171 | No | ns | >0,9999 | E-Q |
| **L.s vs. L.li** | 524,4 | -16269 to 17318 | No | ns | >0,9999 | E-R |
| **L.s vs. Control** | 637,3 | -17175 to 18449 | No | ns | >0,9999 | E-S |
| **L.s vs. LPS** | 477,2 | -17335 to 18289 | No | ns | >0,9999 | E-T |
| **L.s vs. S.a** | -6328 | -24140 to 11484 | No | ns | 0,9998 | E-U |
| **L.s vs. L.g** | 590,0 | -16203 to 17383 | No | ns | >0,9999 | E-V |
| **L.s vs. L.s** | 262,8 | -16531 to 17056 | No | ns | >0,9999 | E-W |
| **L.s vs. L.la** | 360,8 | -16432 to 17154 | No | ns | >0,9999 | E-X |
| **L.s vs. L.b** | 591,3 | -16202 to 17385 | No | ns | >0,9999 | E-Y |
| **L.s vs. L.n** | 594,5 | -16199 to 17388 | No | ns | >0,9999 | E-Z |
| **L.s vs. L.li** | 642,6 | -16151 to 17436 | No | ns | >0,9999 | E-AA |
| **L.la vs. L.b** | 5600 | -11193 to 22393 | No | ns | >0,9999 | F-G |
| **L.la vs. L.n** | -5302 | -22096 to 11491 | No | ns | >0,9999 | F-H |
| **L.la vs. L.li** | -55695 | -72488 to -38902 | Yes | **** | <0,0001 | F-I |
| **L.la vs. Control** | 5671 | -12141 to 23483 | No | ns | >0,9999 | F-J |
| **L.la vs. LPS** | 1866 | -15946 to 19678 | No | ns | >0,9999 | F-K |
| **L.la vs. S.a** | 5495 | -12317 to 23307 | No | ns | >0,9999 | F-L |
| **L.la vs. L.g** | 5437 | -11357 to 22230 | No | ns | >0,9999 | F-M |
| **L.la vs. L.s** | 5322 | -11471 to 22115 | No | ns | >0,9999 | F-N |
| **L.la vs. L.la** | -1311 | -18104 to 15483 | No | ns | >0,9999 | F-O |
| **L.la vs. L.b** | 5515 | -11278 to 22309 | No | ns | >0,9999 | F-P |
| **L.la vs. L.n** | 5410 | -11383 to 22204 | No | ns | >0,9999 | F-Q |
| **L.la vs. L.li** | 5557 | -11237 to 22350 | No | ns | >0,9999 | F-R |
| **L.la vs. Control** | 5670 | -12142 to 23482 | No | ns | >0,9999 | F-S |
| **L.la vs. LPS** | 5510 | -12302 to 23322 | No | ns | >0,9999 | F-T |
| **L.la vs. S.a** | -1296 | -19108 to 16517 | No | ns | >0,9999 | F-U |
| **L.la vs. L.g** | 5622 | -11171 to 22416 | No | ns | >0,9999 | F-V |
| **L.la vs. L.s** | 5295 | -11498 to 22089 | No | ns | >0,9999 | F-W |
| **L.la vs. L.la** | 5393 | -11400 to 22187 | No | ns | >0,9999 | F-X |
| **L.la vs. L.b** | 5624 | -11170 to 22417 | No | ns | >0,9999 | F-Y |
| **L.la vs. L.n** | 5627 | -11166 to 22420 | No | ns | >0,9999 | F-Z |
| **L.la vs. L.li** | 5675 | -11118 to 22468 | No | ns | >0,9999 | F-AA |
| **L.b vs. L.n** | -10902 | -27696 to 5891 | No | ns | 0,7462 | G-H |
| **L.b vs. L.li** | -61295 | -78088 to -44502 | Yes | **** | <0,0001 | G-I |
| **L.b vs. Control** | 71,25 | -17741 to 17883 | No | ns | >0,9999 | G-J |
| **L.b vs. LPS** | -3735 | -21547 to 14078 | No | ns | >0,9999 | G-K |
| **L.b vs. S.a** | -104,8 | -17917 to 17707 | No | ns | >0,9999 | G-L |
| **L.b vs. L.g** | -163,3 | -16957 to 16630 | No | ns | >0,9999 | G-M |
| **L.b vs. L.s** | -278,1 | -17071 to 16515 | No | ns | >0,9999 | G-N |
| **L.b vs. L.la** | -6911 | -23704 to 9882 | No | ns | 0,9983 | G-O |
| **L.b vs. L.b** | -84,77 | -16878 to 16709 | No | ns | >0,9999 | G-P |
| **L.b vs. L.n** | -189,9 | -16983 to 16603 | No | ns | >0,9999 | G-Q |
| **L.b vs. L.li** | -43,28 | -16837 to 16750 | No | ns | >0,9999 | G-R |
| **L.b vs. Control** | 69,67 | -17742 to 17882 | No | ns | >0,9999 | G-S |
| **L.b vs. LPS** | -90,50 | -17903 to 17722 | No | ns | >0,9999 | G-T |
| **L.b vs. S.a** | -6896 | -24708 to 10916 | No | ns | 0,9993 | G-U |
| **L.b vs. L.g** | 22,35 | -16771 to 16816 | No | ns | >0,9999 | G-V |
| **L.b vs. L.s** | -304,9 | -17098 to 16488 | No | ns | >0,9999 | G-W |
| **L.b vs. L.la** | -206,8 | -17000 to 16587 | No | ns | >0,9999 | G-X |
| **L.b vs. L.b** | 23,64 | -16770 to 16817 | No | ns | >0,9999 | G-Y |
| **L.b vs. L.n** | 26,87 | -16766 to 16820 | No | ns | >0,9999 | G-Z |
| **L.b vs. L.li** | 74,97 | -16718 to 16868 | No | ns | >0,9999 | G-AA |
| **L.n vs. L.li** | -50393 | -67186 to -33599 | Yes | **** | <0,0001 | H-I |
| **L.n vs. Control** | 10974 | -6838 to 28786 | No | ns | 0,8246 | H-J |
| **L.n vs. LPS** | 7168 | -10644 to 24980 | No | ns | 0,9988 | H-K |
| **L.n vs. S.a** | 10798 | -7014 to 28610 | No | ns | 0,8452 | H-L |
| **L.n vs. L.g** | 10739 | -6054 to 27532 | No | ns | 0,7706 | H-M |
| **L.n vs. L.s** | 10624 | -6169 to 27418 | No | ns | 0,7870 | H-N |
| **L.n vs. L.la** | 3991 | -12802 to 20785 | No | ns | >0,9999 | H-O |
| **L.n vs. L.b** | 10818 | -5976 to 27611 | No | ns | 0,7590 | H-P |
| **L.n vs. L.n** | 10712 | -6081 to 27506 | No | ns | 0,7744 | H-Q |
| **L.n vs. L.li** | 10859 | -5934 to 27652 | No | ns | 0,7527 | H-R |
| **L.n vs. Control** | 10972 | -6840 to 28784 | No | ns | 0,8248 | H-S |
| **L.n vs. LPS** | 10812 | -7000 to 28624 | No | ns | 0,8436 | H-T |
| **L.n vs. S.a** | 4007 | -13805 to 21819 | No | ns | >0,9999 | H-U |
| **L.n vs. L.g** | 10925 | -5869 to 27718 | No | ns | 0,7428 | H-V |
| **L.n vs. L.s** | 10597 | -6196 to 27391 | No | ns | 0,7908 | H-W |
| **L.n vs. L.la** | 10695 | -6098 to 27489 | No | ns | 0,7769 | H-X |
| **L.n vs. L.b** | 10926 | -5867 to 27719 | No | ns | 0,7426 | H-Y |
| **L.n vs. L.n** | 10929 | -5864 to 27723 | No | ns | 0,7421 | H-Z |
| **L.n vs. L.li** | 10977 | -5816 to 27771 | No | ns | 0,7347 | H-AA |
| **L.li vs. Control** | 61366 | 43554 to 79178 | Yes | **** | <0,0001 | I-J |
| **L.li vs. LPS** | 57561 | 39749 to 75373 | Yes | **** | <0,0001 | I-K |
| **L.li vs. S.a** | 61190 | 43378 to 79002 | Yes | **** | <0,0001 | I-L |
| **L.li vs. L.g** | 61132 | 44338 to 77925 | Yes | **** | <0,0001 | I-M |
| **L.li vs. L.s** | 61017 | 44224 to 77810 | Yes | **** | <0,0001 | I-N |
| **L.li vs. L.la** | 54384 | 37591 to 71178 | Yes | **** | <0,0001 | I-O |
| **L.li vs. L.b** | 61210 | 44417 to 78004 | Yes | **** | <0,0001 | I-P |
| **L.li vs. L.n** | 61105 | 44312 to 77899 | Yes | **** | <0,0001 | I-Q |
| **L.li vs. L.li** | 61252 | 44458 to 78045 | Yes | **** | <0,0001 | I-R |
| **L.li vs. Control** | 61365 | 43553 to 79177 | Yes | **** | <0,0001 | I-S |
| **L.li vs. LPS** | 61205 | 43393 to 79017 | Yes | **** | <0,0001 | I-T |
| **L.li vs. S.a** | 54400 | 36587 to 72212 | Yes | **** | <0,0001 | I-U |
| **L.li vs. L.g** | 61317 | 44524 to 78111 | Yes | **** | <0,0001 | I-V |
| **L.li vs. L.s** | 60990 | 44197 to 77784 | Yes | **** | <0,0001 | I-W |
| **L.li vs. L.la** | 61088 | 44295 to 77882 | Yes | **** | <0,0001 | I-X |
| **L.li vs. L.b** | 61319 | 44525 to 78112 | Yes | **** | <0,0001 | I-Y |
| **L.li vs. L.n** | 61322 | 44529 to 78115 | Yes | **** | <0,0001 | I-Z |
| **L.li vs. L.li** | 61370 | 44577 to 78163 | Yes | **** | <0,0001 | I-AA |
| **Control vs. LPS** | -3806 | -22581 to 14970 | No | ns | >0,9999 | J-K |
| **Control vs. S.a** | -176,0 | -18952 to 18600 | No | ns | >0,9999 | J-L |
| **Control vs. L.g** | -234,6 | -18047 to 17577 | No | ns | >0,9999 | J-M |
| **Control vs. L.s** | -349,3 | -18161 to 17463 | No | ns | >0,9999 | J-N |
| **Control vs. L.la** | -6982 | -24794 to 10830 | No | ns | 0,9992 | J-O |
| **Control vs. L.b** | -156,0 | -17968 to 17656 | No | ns | >0,9999 | J-P |
| **Control vs. L.n** | -261,1 | -18073 to 17551 | No | ns | >0,9999 | J-Q |
| **Control vs. L.li** | -114,5 | -17927 to 17697 | No | ns | >0,9999 | J-R |
| **Control vs. Control** | -1,585 | -18777 to 18774 | No | ns | >0,9999 | J-S |
| **Control vs. LPS** | -161,8 | -18937 to 18614 | No | ns | >0,9999 | J-T |
| **Control vs. S.a** | -6967 | -25742 to 11809 | No | ns | 0,9997 | J-U |
| **Control vs. L.g** | -48,90 | -17861 to 17763 | No | ns | >0,9999 | J-V |
| **Control vs. L.s** | -376,1 | -18188 to 17436 | No | ns | >0,9999 | J-W |
| **Control vs. L.la** | -278,1 | -18090 to 17534 | No | ns | >0,9999 | J-X |
| **Control vs. L.b** | -47,61 | -17860 to 17764 | No | ns | >0,9999 | J-Y |
| **Control vs. L.n** | -44,38 | -17856 to 17768 | No | ns | >0,9999 | J-Z |
| **Control vs. L.li** | 3,721 | -17808 to 17816 | No | ns | >0,9999 | J-AA |
| **LPS vs. S.a** | 3630 | -15146 to 22405 | No | ns | >0,9999 | K-L |
| **LPS vs. L.g** | 3571 | -14241 to 21383 | No | ns | >0,9999 | K-M |
| **LPS vs. L.s** | 3456 | -14356 to 21268 | No | ns | >0,9999 | K-N |
| **LPS vs. L.la** | -3176 | -20988 to 14636 | No | ns | >0,9999 | K-O |
| **LPS vs. L.b** | 3650 | -14162 to 21462 | No | ns | >0,9999 | K-P |
| **LPS vs. L.n** | 3545 | -14267 to 21357 | No | ns | >0,9999 | K-Q |
| **LPS vs. L.li** | 3691 | -14121 to 21503 | No | ns | >0,9999 | K-R |
| **LPS vs. Control** | 3804 | -14971 to 22580 | No | ns | >0,9999 | K-S |
| **LPS vs. LPS** | 3644 | -15132 to 22420 | No | ns | >0,9999 | K-T |
| **LPS vs. S.a** | -3161 | -21937 to 15614 | No | ns | >0,9999 | K-U |
| **LPS vs. L.g** | 3757 | -14055 to 21569 | No | ns | >0,9999 | K-V |
| **LPS vs. L.s** | 3430 | -14382 to 21242 | No | ns | >0,9999 | K-W |
| **LPS vs. L.la** | 3528 | -14284 to 21340 | No | ns | >0,9999 | K-X |
| **LPS vs. L.b** | 3758 | -14054 to 21570 | No | ns | >0,9999 | K-Y |
| **LPS vs. L.n** | 3761 | -14051 to 21573 | No | ns | >0,9999 | K-Z |
| **LPS vs. L.li** | 3809 | -14003 to 21621 | No | ns | >0,9999 | K-AA |
| **S.a vs. L.g** | -58,56 | -17871 to 17753 | No | ns | >0,9999 | L-M |
| **S.a vs. L.s** | -173,3 | -17985 to 17639 | No | ns | >0,9999 | L-N |
| **S.a vs. L.la** | -6806 | -24618 to 11006 | No | ns | 0,9995 | L-O |
| **S.a vs. L.b** | 19,98 | -17792 to 17832 | No | ns | >0,9999 | L-P |
| **S.a vs. L.n** | -85,14 | -17897 to 17727 | No | ns | >0,9999 | L-Q |
| **S.a vs. L.li** | 61,47 | -17751 to 17873 | No | ns | >0,9999 | L-R |
| **S.a vs. Control** | 174,4 | -18601 to 18950 | No | ns | >0,9999 | L-S |
| **S.a vs. LPS** | 14,25 | -18761 to 18790 | No | ns | >0,9999 | L-T |
| **S.a vs. S.a** | -6791 | -25566 to 11985 | No | ns | 0,9998 | L-U |
| **S.a vs. L.g** | 127,1 | -17685 to 17939 | No | ns | >0,9999 | L-V |
| **S.a vs. L.s** | -200,1 | -18012 to 17612 | No | ns | >0,9999 | L-W |
| **S.a vs. L.la** | -102,1 | -17914 to 17710 | No | ns | >0,9999 | L-X |
| **S.a vs. L.b** | 128,4 | -17684 to 17940 | No | ns | >0,9999 | L-Y |
| **S.a vs. L.n** | 131,6 | -17680 to 17944 | No | ns | >0,9999 | L-Z |
| **S.a vs. L.li** | 179,7 | -17632 to 17992 | No | ns | >0,9999 | L-AA |
| **L.g vs. L.s** | -114,8 | -16908 to 16679 | No | ns | >0,9999 | M-N |
| **L.g vs. L.la** | -6748 | -23541 to 10046 | No | ns | 0,9988 | M-O |
| **L.g vs. L.b** | 78,54 | -16715 to 16872 | No | ns | >0,9999 | M-P |
| **L.g vs. L.n** | -26,57 | -16820 to 16767 | No | ns | >0,9999 | M-Q |
| **L.g vs. L.li** | 120,0 | -16673 to 16913 | No | ns | >0,9999 | M-R |
| **L.g vs. Control** | 233,0 | -17579 to 18045 | No | ns | >0,9999 | M-S |
| **L.g vs. LPS** | 72,81 | -17739 to 17885 | No | ns | >0,9999 | M-T |
| **L.g vs. S.a** | -6732 | -24544 to 11080 | No | ns | 0,9995 | M-U |
| **L.g vs. L.g** | 185,7 | -16608 to 16979 | No | ns | >0,9999 | M-V |
| **L.g vs. L.s** | -141,5 | -16935 to 16652 | No | ns | >0,9999 | M-W |
| **L.g vs. L.la** | -43,50 | -16837 to 16750 | No | ns | >0,9999 | M-X |
| **L.g vs. L.b** | 187,0 | -16606 to 16980 | No | ns | >0,9999 | M-Y |
| **L.g vs. L.n** | 190,2 | -16603 to 16984 | No | ns | >0,9999 | M-Z |
| **L.g vs. L.li** | 238,3 | -16555 to 17032 | No | ns | >0,9999 | M-AA |
| **L.s vs. L.la** | -6633 | -23426 to 10161 | No | ns | 0,9991 | N-O |
| **L.s vs. L.b** | 193,3 | -16600 to 16987 | No | ns | >0,9999 | N-P |
| **L.s vs. L.n** | 88,20 | -16705 to 16882 | No | ns | >0,9999 | N-Q |
| **L.s vs. L.li** | 234,8 | -16559 to 17028 | No | ns | >0,9999 | N-R |
| **L.s vs. Control** | 347,7 | -17464 to 18160 | No | ns | >0,9999 | N-S |
| **L.s vs. LPS** | 187,6 | -17624 to 18000 | No | ns | >0,9999 | N-T |
| **L.s vs. S.a** | -6618 | -24430 to 11195 | No | ns | 0,9997 | N-U |
| **L.s vs. L.g** | 300,4 | -16493 to 17094 | No | ns | >0,9999 | N-V |
| **L.s vs. L.s** | -26,77 | -16820 to 16767 | No | ns | >0,9999 | N-W |
| **L.s vs. L.la** | 71,27 | -16722 to 16865 | No | ns | >0,9999 | N-X |
| **L.s vs. L.b** | 301,7 | -16492 to 17095 | No | ns | >0,9999 | N-Y |
| **L.s vs. L.n** | 305,0 | -16488 to 17098 | No | ns | >0,9999 | N-Z |
| **L.s vs. L.li** | 353,1 | -16440 to 17146 | No | ns | >0,9999 | N-AA |
| **L.la vs. L.b** | 6826 | -9967 to 23619 | No | ns | 0,9986 | O-P |
| **L.la vs. L.n** | 6721 | -10072 to 23514 | No | ns | 0,9989 | O-Q |
| **L.la vs. L.li** | 6868 | -9926 to 23661 | No | ns | 0,9984 | O-R |
| **L.la vs. Control** | 6981 | -10831 to 24793 | No | ns | 0,9992 | O-S |
| **L.la vs. LPS** | 6820 | -10992 to 24632 | No | ns | 0,9994 | O-T |
| **L.la vs. S.a** | 15,30 | -17797 to 17827 | No | ns | >0,9999 | O-U |
| **L.la vs. L.g** | 6933 | -9860 to 23727 | No | ns | 0,9982 | O-V |
| **L.la vs. L.s** | 6606 | -10187 to 23399 | No | ns | 0,9991 | O-W |
| **L.la vs. L.la** | 6704 | -10089 to 23497 | No | ns | 0,9989 | O-X |
| **L.la vs. L.b** | 6935 | -9859 to 23728 | No | ns | 0,9982 | O-Y |
| **L.la vs. L.n** | 6938 | -9856 to 23731 | No | ns | 0,9982 | O-Z |
| **L.la vs. L.li** | 6986 | -9807 to 23779 | No | ns | 0,9980 | O-AA |
| **L.b vs. L.n** | -105,1 | -16898 to 16688 | No | ns | >0,9999 | P-Q |
| **L.b vs. L.li** | 41,49 | -16752 to 16835 | No | ns | >0,9999 | P-R |
| **L.b vs. Control** | 154,4 | -17658 to 17966 | No | ns | >0,9999 | P-S |
| **L.b vs. LPS** | -5,726 | -17818 to 17806 | No | ns | >0,9999 | P-T |
| **L.b vs. S.a** | -6811 | -24623 to 11001 | No | ns | 0,9995 | P-U |
| **L.b vs. L.g** | 107,1 | -16686 to 16900 | No | ns | >0,9999 | P-V |
| **L.b vs. L.s** | -220,1 | -17013 to 16573 | No | ns | >0,9999 | P-W |
| **L.b vs. L.la** | -122,0 | -16915 to 16671 | No | ns | >0,9999 | P-X |
| **L.b vs. L.b** | 108,4 | -16685 to 16902 | No | ns | >0,9999 | P-Y |
| **L.b vs. L.n** | 111,6 | -16682 to 16905 | No | ns | >0,9999 | P-Z |
| **L.b vs. L.li** | 159,7 | -16634 to 16953 | No | ns | >0,9999 | P-AA |
| **L.n vs. L.li** | 146,6 | -16647 to 16940 | No | ns | >0,9999 | Q-R |
| **L.n vs. Control** | 259,6 | -17552 to 18072 | No | ns | >0,9999 | Q-S |
| **L.n vs. LPS** | 99,39 | -17713 to 17911 | No | ns | >0,9999 | Q-T |
| **L.n vs. S.a** | -6706 | -24518 to 11106 | No | ns | 0,9996 | Q-U |
| **L.n vs. L.g** | 212,2 | -16581 to 17006 | No | ns | >0,9999 | Q-V |
| **L.n vs. L.s** | -115,0 | -16908 to 16678 | No | ns | >0,9999 | Q-W |
| **L.n vs. L.la** | -16,93 | -16810 to 16776 | No | ns | >0,9999 | Q-X |
| **L.n vs. L.b** | 213,5 | -16580 to 17007 | No | ns | >0,9999 | Q-Y |
| **L.n vs. L.n** | 216,8 | -16577 to 17010 | No | ns | >0,9999 | Q-Z |
| **L.n vs. L.li** | 264,9 | -16528 to 17058 | No | ns | >0,9999 | Q-AA |
| **L.li vs. Control** | 112,9 | -17699 to 17925 | No | ns | >0,9999 | R-S |
| **L.li vs. LPS** | -47,22 | -17859 to 17765 | No | ns | >0,9999 | R-T |
| **L.li vs. S.a** | -6852 | -24664 to 10960 | No | ns | 0,9994 | R-U |
| **L.li vs. L.g** | 65,63 | -16728 to 16859 | No | ns | >0,9999 | R-V |
| **L.li vs. L.s** | -261,6 | -17055 to 16532 | No | ns | >0,9999 | R-W |
| **L.li vs. L.la** | -163,5 | -16957 to 16630 | No | ns | >0,9999 | R-X |
| **L.li vs. L.b** | 66,92 | -16726 to 16860 | No | ns | >0,9999 | R-Y |
| **L.li vs. L.n** | 70,15 | -16723 to 16863 | No | ns | >0,9999 | R-Z |
| **L.li vs. L.li** | 118,3 | -16675 to 16912 | No | ns | >0,9999 | R-AA |
| **Control vs. LPS** | -160,2 | -18936 to 18615 | No | ns | >0,9999 | S-T |
| **Control vs. S.a** | -6965 | -25741 to 11810 | No | ns | 0,9997 | S-U |
| **Control vs. L.g** | -47,31 | -17859 to 17765 | No | ns | >0,9999 | S-V |
| **Control vs. L.s** | -374,5 | -18187 to 17437 | No | ns | >0,9999 | S-W |
| **Control vs. L.la** | -276,5 | -18089 to 17536 | No | ns | >0,9999 | S-X |
| **Control vs. L.b** | -46,02 | -17858 to 17766 | No | ns | >0,9999 | S-Y |
| **Control vs. L.n** | -42,79 | -17855 to 17769 | No | ns | >0,9999 | S-Z |
| **Control vs. L.li** | 5,306 | -17807 to 17817 | No | ns | >0,9999 | S-AA |
| **LPS vs. S.a** | -6805 | -25581 to 11970 | No | ns | 0,9998 | T-U |
| **LPS vs. L.g** | 112,9 | -17699 to 17925 | No | ns | >0,9999 | T-V |
| **LPS vs. L.s** | -214,4 | -18026 to 17598 | No | ns | >0,9999 | T-W |
| **LPS vs. L.la** | -116,3 | -17928 to 17696 | No | ns | >0,9999 | T-X |
| **LPS vs. L.b** | 114,1 | -17698 to 17926 | No | ns | >0,9999 | T-Y |
| **LPS vs. L.n** | 117,4 | -17695 to 17929 | No | ns | >0,9999 | T-Z |
| **LPS vs. L.li** | 165,5 | -17647 to 17977 | No | ns | >0,9999 | T-AA |
| **S.a vs. L.g** | 6918 | -10894 to 24730 | No | ns | 0,9993 | U-V |
| **S.a vs. L.s** | 6591 | -11221 to 24403 | No | ns | 0,9997 | U-W |
| **S.a vs. L.la** | 6689 | -11123 to 24501 | No | ns | 0,9996 | U-X |
| **S.a vs. L.b** | 6919 | -10893 to 24731 | No | ns | 0,9993 | U-Y |
| **S.a vs. L.n** | 6922 | -10890 to 24734 | No | ns | 0,9993 | U-Z |
| **S.a vs. L.li** | 6971 | -10841 to 24783 | No | ns | 0,9992 | U-AA |
| **L.g vs. L.s** | -327,2 | -17121 to 16466 | No | ns | >0,9999 | V-W |
| **L.g vs. L.la** | -229,2 | -17023 to 16564 | No | ns | >0,9999 | V-X |
| **L.g vs. L.b** | 1,290 | -16792 to 16795 | No | ns | >0,9999 | V-Y |
| **L.g vs. L.n** | 4,520 | -16789 to 16798 | No | ns | >0,9999 | V-Z |
| **L.g vs. L.li** | 52,62 | -16741 to 16846 | No | ns | >0,9999 | V-AA |
| **L.s vs. L.la** | 98,04 | -16695 to 16891 | No | ns | >0,9999 | W-X |
| **L.s vs. L.b** | 328,5 | -16465 to 17122 | No | ns | >0,9999 | W-Y |
| **L.s vs. L.n** | 331,7 | -16462 to 17125 | No | ns | >0,9999 | W-Z |
| **L.s vs. L.li** | 379,8 | -16414 to 17173 | No | ns | >0,9999 | W-AA |
| **L.la vs. L.b** | 230,5 | -16563 to 17024 | No | ns | >0,9999 | X-Y |
| **L.la vs. L.n** | 233,7 | -16560 to 17027 | No | ns | >0,9999 | X-Z |
| **L.la vs. L.li** | 281,8 | -16512 to 17075 | No | ns | >0,9999 | X-AA |
| **L.b vs. L.n** | 3,230 | -16790 to 16797 | No | ns | >0,9999 | Y-Z |
| **L.b vs. L.li** | 51,33 | -16742 to 16845 | No | ns | >0,9999 | Y-AA |
| **L.n vs. L.li** | 48,10 | -16745 to 16841 | No | ns | >0,9999 | Z-AA |

**TNF-α (pg/mL)**

**GIPL (WT, TLR2KO and TLR4KO)**

| Control | LPS | S.a | L.g | L.s | L.la | L.b | L.n | L.li |
| --- | --- | --- | --- | --- | --- | --- | --- | --- |
| 13,6 | 7053,4 | 5693,1 | 59 | 783 | 5994 | 42,3 | 91,9 | 106,9 |
| 10,8 | 8277,4 | 5234,6 | 60,1 | 457,4 | 5227 | 61,9 | 79,2 | 126 |
| 10,2 | 5685,6 | 5015,9 | 59 | 976,1 | 4957 | 38,8 | 120,2 | 136,4 |
| 9,6 | 6958,1 | 6143,6 | 47,4 | 686 | 2252 | 105,7 | 93 | 132,4 |
|  |  |  | 54,9 | 624 | 6681 | 78,6 | 94,2 | 140,5 |
| Control | LPS | S.a | L.g | L.s | L.la | L.b | L.n | L.li |
| 7 | 3187 | 267 | 128,21 | 543,97 | 5311 | 185,22 | 290,06 | 470,44 |
| 4 | 3522 | 100 | 123,94 | 653,12 | 3816 | 207,72 | 470,44 | 294,67 |
| 6 | 4272 | 239 | 41,98 | 575,96 | 3784 | 78,78 | 163 | 401,11 |
| 4 | 4263 | 119 | 67,2 | 154,2 | 9667 | 136,8 | 203,2 | 442,74 |
|  |  |  | 35,37 | 299,29 | 8664 | 171,85 | 276,23 | 639,56 |
| Control | LPS | S.a | L.g | L.s | L.la | L.b | L.n | L.li |
| 7,39 | 200 | 7903,47 | 59 | 783 | 5994 | 42,3 | 91,9 | 106,9 |
| 8,51 | 170 | 5464,4 | 60,1 | 457,4 | 5227 | 61,9 | 79,2 | 126 |
| 4,05 | 108 | 6518,25 | 59 | 976,1 | 4957 | 38,8 | 120,2 | 136,4 |
| 7,39 | 190 | 8002,28 | 47,4 | 686 | 2252 | 105,7 | 93 | 132,4 |
|  |  |  | 54,9 | 624 | 6681 | 78,6 | 94,2 | 140,5 |

**Statistical analysis**

| **Tukey's multiple comparisons test** | **Mean Diff,** | **95,00% CI of diff,** | **Significant?** | **Summary** | **Adjusted P Value** |  |
| --- | --- | --- | --- | --- | --- | --- |
| **Control vs. LPS** | -6983 | -9134 to -4831 | Yes | **** | <0,0001 | A-B |
| **Control vs. S.a** | -5511 | -7662 to -3359 | Yes | **** | <0,0001 | A-C |
| **Control vs. L.g** | -45,03 | -2086 to 1996 | No | ns | >0,9999 | A-D |
| **Control vs. L.s** | -694,3 | -2735 to 1347 | No | ns | >0,9999 | A-E |
| **Control vs. L.la** | -5011 | -7052 to -2970 | Yes | **** | <0,0001 | A-F |
| **Control vs. L.b** | -54,41 | -2096 to 1987 | No | ns | >0,9999 | A-G |
| **Control vs. L.n** | -84,65 | -2126 to 1957 | No | ns | >0,9999 | A-H |
| **Control vs. L.li** | -117,4 | -2159 to 1924 | No | ns | >0,9999 | A-I |
| **Control vs. Control** | 5,800 | -2146 to 2157 | No | ns | >0,9999 | A-J |
| **Control vs. LPS** | -3800 | -5952 to -1648 | Yes | **** | <0,0001 | A-K |
| **Control vs. S.a** | -170,2 | -2322 to 1981 | No | ns | >0,9999 | A-L |
| **Control vs. L.g** | -68,29 | -2109 to 1973 | No | ns | >0,9999 | A-M |
| **Control vs. L.s** | -434,3 | -2475 to 1607 | No | ns | >0,9999 | A-N |
| **Control vs. L.la** | -6237 | -8279 to -4196 | Yes | **** | <0,0001 | A-O |
| **Control vs. L.b** | -145,0 | -2186 to 1896 | No | ns | >0,9999 | A-P |
| **Control vs. L.n** | -269,5 | -2311 to 1772 | No | ns | >0,9999 | A-Q |
| **Control vs. L.li** | -438,7 | -2480 to 1603 | No | ns | >0,9999 | A-R |
| **Control vs. Control** | 4,215 | -2147 to 2156 | No | ns | >0,9999 | A-S |
| **Control vs. LPS** | -156,0 | -2308 to 1996 | No | ns | >0,9999 | A-T |
| **Control vs. S.a** | -6961 | -9113 to -4809 | Yes | **** | <0,0001 | A-U |
| **Control vs. L.g** | -45,03 | -2086 to 1996 | No | ns | >0,9999 | A-V |
| **Control vs. L.s** | -694,3 | -2735 to 1347 | No | ns | >0,9999 | A-W |
| **Control vs. L.la** | -5011 | -7052 to -2970 | Yes | **** | <0,0001 | A-X |
| **Control vs. L.b** | -54,41 | -2096 to 1987 | No | ns | >0,9999 | A-Y |
| **Control vs. L.n** | -84,65 | -2126 to 1957 | No | ns | >0,9999 | A-Z |
| **Control vs. L.li** | -117,4 | -2159 to 1924 | No | ns | >0,9999 | A-AA |
| **LPS vs. S.a** | 1472 | -679,7 to 3623 | No | ns | 0,6518 | B-C |
| **LPS vs. L.g** | 6938 | 4896 to 8979 | Yes | **** | <0,0001 | B-D |
| **LPS vs. L.s** | 6288 | 4247 to 8329 | Yes | **** | <0,0001 | B-E |
| **LPS vs. L.la** | 1971 | -69,73 to 4013 | No | ns | 0,0729 | B-F |
| **LPS vs. L.b** | 6928 | 4887 to 8969 | Yes | **** | <0,0001 | B-G |
| **LPS vs. L.n** | 6898 | 4857 to 8939 | Yes | **** | <0,0001 | B-H |
| **LPS vs. L.li** | 6865 | 4824 to 8906 | Yes | **** | <0,0001 | B-I |
| **LPS vs. Control** | 6988 | 4837 to 9140 | Yes | **** | <0,0001 | B-J |
| **LPS vs. LPS** | 3183 | 1031 to 5334 | Yes | **** | <0,0001 | B-K |
| **LPS vs. S.a** | 6812 | 4661 to 8964 | Yes | **** | <0,0001 | B-L |
| **LPS vs. L.g** | 6914 | 4873 to 8955 | Yes | **** | <0,0001 | B-M |
| **LPS vs. L.s** | 6548 | 4507 to 8589 | Yes | **** | <0,0001 | B-N |
| **LPS vs. L.la** | 745,2 | -1296 to 2786 | No | ns | 0,9997 | B-O |
| **LPS vs. L.b** | 6838 | 4796 to 8879 | Yes | **** | <0,0001 | B-P |
| **LPS vs. L.n** | 6713 | 4672 to 8754 | Yes | **** | <0,0001 | B-Q |
| **LPS vs. L.li** | 6544 | 4503 to 8585 | Yes | **** | <0,0001 | B-R |
| **LPS vs. Control** | 6987 | 4835 to 9138 | Yes | **** | <0,0001 | B-S |
| **LPS vs. LPS** | 6827 | 4675 to 8978 | Yes | **** | <0,0001 | B-T |
| **LPS vs. S.a** | 21,52 | -2130 to 2173 | No | ns | >0,9999 | B-U |
| **LPS vs. L.g** | 6938 | 4896 to 8979 | Yes | **** | <0,0001 | B-V |
| **LPS vs. L.s** | 6288 | 4247 to 8329 | Yes | **** | <0,0001 | B-W |
| **LPS vs. L.la** | 1971 | -69,73 to 4013 | No | ns | 0,0729 | B-X |
| **LPS vs. L.b** | 6928 | 4887 to 8969 | Yes | **** | <0,0001 | B-Y |
| **LPS vs. L.n** | 6898 | 4857 to 8939 | Yes | **** | <0,0001 | B-Z |
| **LPS vs. L.li** | 6865 | 4824 to 8906 | Yes | **** | <0,0001 | B-AA |
| **S.a vs. L.g** | 5466 | 3425 to 7507 | Yes | **** | <0,0001 | C-D |
| **S.a vs. L.s** | 4817 | 2775 to 6858 | Yes | **** | <0,0001 | C-E |
| **S.a vs. L.la** | 499,6 | -1542 to 2541 | No | ns | >0,9999 | C-F |
| **S.a vs. L.b** | 5456 | 3415 to 7497 | Yes | **** | <0,0001 | C-G |
| **S.a vs. L.n** | 5426 | 3385 to 7467 | Yes | **** | <0,0001 | C-H |
| **S.a vs. L.li** | 5393 | 3352 to 7435 | Yes | **** | <0,0001 | C-I |
| **S.a vs. Control** | 5517 | 3365 to 7668 | Yes | **** | <0,0001 | C-J |
| **S.a vs. LPS** | 1711 | -440,8 to 3862 | No | ns | 0,3430 | C-K |
| **S.a vs. S.a** | 5341 | 3189 to 7492 | Yes | **** | <0,0001 | C-L |
| **S.a vs. L.g** | 5442 | 3401 to 7484 | Yes | **** | <0,0001 | C-M |
| **S.a vs. L.s** | 5076 | 3035 to 7118 | Yes | **** | <0,0001 | C-N |
| **S.a vs. L.la** | -726,6 | -2768 to 1315 | No | ns | 0,9998 | C-O |
| **S.a vs. L.b** | 5366 | 3325 to 7407 | Yes | **** | <0,0001 | C-P |
| **S.a vs. L.n** | 5241 | 3200 to 7282 | Yes | **** | <0,0001 | C-Q |
| **S.a vs. L.li** | 5072 | 3031 to 7113 | Yes | **** | <0,0001 | C-R |
| **S.a vs. Control** | 5515 | 3363 to 7667 | Yes | **** | <0,0001 | C-S |
| **S.a vs. LPS** | 5355 | 3203 to 7506 | Yes | **** | <0,0001 | C-T |
| **S.a vs. S.a** | -1450 | -3602 to 701,3 | No | ns | 0,6798 | C-U |
| **S.a vs. L.g** | 5466 | 3425 to 7507 | Yes | **** | <0,0001 | C-V |
| **S.a vs. L.s** | 4817 | 2775 to 6858 | Yes | **** | <0,0001 | C-W |
| **S.a vs. L.la** | 499,6 | -1542 to 2541 | No | ns | >0,9999 | C-X |
| **S.a vs. L.b** | 5456 | 3415 to 7497 | Yes | **** | <0,0001 | C-Y |
| **S.a vs. L.n** | 5426 | 3385 to 7467 | Yes | **** | <0,0001 | C-Z |
| **S.a vs. L.li** | 5393 | 3352 to 7435 | Yes | **** | <0,0001 | C-AA |
| **L.g vs. L.s** | -649,2 | -2574 to 1275 | No | ns | >0,9999 | D-E |
| **L.g vs. L.la** | -4966 | -6891 to -3042 | Yes | **** | <0,0001 | D-F |
| **L.g vs. L.b** | -9,380 | -1934 to 1915 | No | ns | >0,9999 | D-G |
| **L.g vs. L.n** | -39,62 | -1964 to 1885 | No | ns | >0,9999 | D-H |
| **L.g vs. L.li** | -72,36 | -1997 to 1852 | No | ns | >0,9999 | D-I |
| **L.g vs. Control** | 50,83 | -1990 to 2092 | No | ns | >0,9999 | D-J |
| **L.g vs. LPS** | -3755 | -5796 to -1714 | Yes | **** | <0,0001 | D-K |
| **L.g vs. S.a** | -125,2 | -2166 to 1916 | No | ns | >0,9999 | D-L |
| **L.g vs. L.g** | -23,26 | -1948 to 1901 | No | ns | >0,9999 | D-M |
| **L.g vs. L.s** | -389,2 | -2314 to 1535 | No | ns | >0,9999 | D-N |
| **L.g vs. L.la** | -6192 | -8117 to -4268 | Yes | **** | <0,0001 | D-O |
| **L.g vs. L.b** | -99,99 | -2024 to 1824 | No | ns | >0,9999 | D-P |
| **L.g vs. L.n** | -224,5 | -2149 to 1700 | No | ns | >0,9999 | D-Q |
| **L.g vs. L.li** | -393,6 | -2318 to 1531 | No | ns | >0,9999 | D-R |
| **L.g vs. Control** | 49,25 | -1992 to 2090 | No | ns | >0,9999 | D-S |
| **L.g vs. LPS** | -110,9 | -2152 to 1930 | No | ns | >0,9999 | D-T |
| **L.g vs. S.a** | -6916 | -8957 to -4875 | Yes | **** | <0,0001 | D-U |
| **L.g vs. L.g** | 0,000 | -1924 to 1924 | No | ns | >0,9999 | D-V |
| **L.g vs. L.s** | -649,2 | -2574 to 1275 | No | ns | >0,9999 | D-W |
| **L.g vs. L.la** | -4966 | -6891 to -3042 | Yes | **** | <0,0001 | D-X |
| **L.g vs. L.b** | -9,380 | -1934 to 1915 | No | ns | >0,9999 | D-Y |
| **L.g vs. L.n** | -39,62 | -1964 to 1885 | No | ns | >0,9999 | D-Z |
| **L.g vs. L.li** | -72,36 | -1997 to 1852 | No | ns | >0,9999 | D-AA |
| **L.s vs. L.la** | -4317 | -6241 to -2392 | Yes | **** | <0,0001 | E-F |
| **L.s vs. L.b** | 639,8 | -1285 to 2564 | No | ns | >0,9999 | E-G |
| **L.s vs. L.n** | 609,6 | -1315 to 2534 | No | ns | >0,9999 | E-H |
| **L.s vs. L.li** | 576,9 | -1348 to 2501 | No | ns | >0,9999 | E-I |
| **L.s vs. Control** | 700,1 | -1341 to 2741 | No | ns | >0,9999 | E-J |
| **L.s vs. LPS** | -3106 | -5147 to -1065 | Yes | **** | <0,0001 | E-K |
| **L.s vs. S.a** | 524,1 | -1517 to 2565 | No | ns | >0,9999 | E-L |
| **L.s vs. L.g** | 626,0 | -1298 to 2550 | No | ns | >0,9999 | E-M |
| **L.s vs. L.s** | 260,0 | -1664 to 2184 | No | ns | >0,9999 | E-N |
| **L.s vs. L.la** | -5543 | -7468 to -3619 | Yes | **** | <0,0001 | E-O |
| **L.s vs. L.b** | 549,2 | -1375 to 2474 | No | ns | >0,9999 | E-P |
| **L.s vs. L.n** | 424,7 | -1500 to 2349 | No | ns | >0,9999 | E-Q |
| **L.s vs. L.li** | 255,6 | -1669 to 2180 | No | ns | >0,9999 | E-R |
| **L.s vs. Control** | 698,5 | -1343 to 2740 | No | ns | >0,9999 | E-S |
| **L.s vs. LPS** | 538,3 | -1503 to 2579 | No | ns | >0,9999 | E-T |
| **L.s vs. S.a** | -6267 | -8308 to -4226 | Yes | **** | <0,0001 | E-U |
| **L.s vs. L.g** | 649,2 | -1275 to 2574 | No | ns | >0,9999 | E-V |
| **L.s vs. L.s** | 0,000 | -1924 to 1924 | No | ns | >0,9999 | E-W |
| **L.s vs. L.la** | -4317 | -6241 to -2392 | Yes | **** | <0,0001 | E-X |
| **L.s vs. L.b** | 639,8 | -1285 to 2564 | No | ns | >0,9999 | E-Y |
| **L.s vs. L.n** | 609,6 | -1315 to 2534 | No | ns | >0,9999 | E-Z |
| **L.s vs. L.li** | 576,9 | -1348 to 2501 | No | ns | >0,9999 | E-AA |
| **L.la vs. L.b** | 4957 | 3032 to 6881 | Yes | **** | <0,0001 | F-G |
| **L.la vs. L.n** | 4927 | 3002 to 6851 | Yes | **** | <0,0001 | F-H |
| **L.la vs. L.li** | 4894 | 2969 to 6818 | Yes | **** | <0,0001 | F-I |
| **L.la vs. Control** | 5017 | 2976 to 7058 | Yes | **** | <0,0001 | F-J |
| **L.la vs. LPS** | 1211 | -830,0 to 3252 | No | ns | 0,8698 | F-K |
| **L.la vs. S.a** | 4841 | 2800 to 6882 | Yes | **** | <0,0001 | F-L |
| **L.la vs. L.g** | 4943 | 3018 to 6867 | Yes | **** | <0,0001 | F-M |
| **L.la vs. L.s** | 4577 | 2652 to 6501 | Yes | **** | <0,0001 | F-N |
| **L.la vs. L.la** | -1226 | -3151 to 698,2 | No | ns | 0,7762 | F-O |
| **L.la vs. L.b** | 4866 | 2942 to 6791 | Yes | **** | <0,0001 | F-P |
| **L.la vs. L.n** | 4742 | 2817 to 6666 | Yes | **** | <0,0001 | F-Q |
| **L.la vs. L.li** | 4572 | 2648 to 6497 | Yes | **** | <0,0001 | F-R |
| **L.la vs. Control** | 5015 | 2974 to 7057 | Yes | **** | <0,0001 | F-S |
| **L.la vs. LPS** | 4855 | 2814 to 6896 | Yes | **** | <0,0001 | F-T |
| **L.la vs. S.a** | -1950 | -3991 to 91,26 | No | ns | 0,0816 | F-U |
| **L.la vs. L.g** | 4966 | 3042 to 6891 | Yes | **** | <0,0001 | F-V |
| **L.la vs. L.s** | 4317 | 2392 to 6241 | Yes | **** | <0,0001 | F-W |
| **L.la vs. L.la** | 0,000 | -1924 to 1924 | No | ns | >0,9999 | F-X |
| **L.la vs. L.b** | 4957 | 3032 to 6881 | Yes | **** | <0,0001 | F-Y |
| **L.la vs. L.n** | 4927 | 3002 to 6851 | Yes | **** | <0,0001 | F-Z |
| **L.la vs. L.li** | 4894 | 2969 to 6818 | Yes | **** | <0,0001 | F-AA |
| **L.b vs. L.n** | -30,24 | -1955 to 1894 | No | ns | >0,9999 | G-H |
| **L.b vs. L.li** | -62,98 | -1987 to 1861 | No | ns | >0,9999 | G-I |
| **L.b vs. Control** | 60,21 | -1981 to 2101 | No | ns | >0,9999 | G-J |
| **L.b vs. LPS** | -3746 | -5787 to -1704 | Yes | **** | <0,0001 | G-K |
| **L.b vs. S.a** | -115,8 | -2157 to 1925 | No | ns | >0,9999 | G-L |
| **L.b vs. L.g** | -13,88 | -1938 to 1911 | No | ns | >0,9999 | G-M |
| **L.b vs. L.s** | -379,8 | -2304 to 1545 | No | ns | >0,9999 | G-N |
| **L.b vs. L.la** | -6183 | -8107 to -4259 | Yes | **** | <0,0001 | G-O |
| **L.b vs. L.b** | -90,61 | -2015 to 1834 | No | ns | >0,9999 | G-P |
| **L.b vs. L.n** | -215,1 | -2140 to 1709 | No | ns | >0,9999 | G-Q |
| **L.b vs. L.li** | -384,2 | -2309 to 1540 | No | ns | >0,9999 | G-R |
| **L.b vs. Control** | 58,63 | -1983 to 2100 | No | ns | >0,9999 | G-S |
| **L.b vs. LPS** | -101,5 | -2143 to 1940 | No | ns | >0,9999 | G-T |
| **L.b vs. S.a** | -6907 | -8948 to -4865 | Yes | **** | <0,0001 | G-U |
| **L.b vs. L.g** | 9,380 | -1915 to 1934 | No | ns | >0,9999 | G-V |
| **L.b vs. L.s** | -639,8 | -2564 to 1285 | No | ns | >0,9999 | G-W |
| **L.b vs. L.la** | -4957 | -6881 to -3032 | Yes | **** | <0,0001 | G-X |
| **L.b vs. L.b** | 0,000 | -1924 to 1924 | No | ns | >0,9999 | G-Y |
| **L.b vs. L.n** | -30,24 | -1955 to 1894 | No | ns | >0,9999 | G-Z |
| **L.b vs. L.li** | -62,98 | -1987 to 1861 | No | ns | >0,9999 | G-AA |
| **L.n vs. L.li** | -32,74 | -1957 to 1892 | No | ns | >0,9999 | H-I |
| **L.n vs. Control** | 90,45 | -1951 to 2132 | No | ns | >0,9999 | H-J |
| **L.n vs. LPS** | -3715 | -5756 to -1674 | Yes | **** | <0,0001 | H-K |
| **L.n vs. S.a** | -85,55 | -2127 to 1956 | No | ns | >0,9999 | H-L |
| **L.n vs. L.g** | 16,36 | -1908 to 1941 | No | ns | >0,9999 | H-M |
| **L.n vs. L.s** | -349,6 | -2274 to 1575 | No | ns | >0,9999 | H-N |
| **L.n vs. L.la** | -6153 | -8077 to -4228 | Yes | **** | <0,0001 | H-O |
| **L.n vs. L.b** | -60,37 | -1985 to 1864 | No | ns | >0,9999 | H-P |
| **L.n vs. L.n** | -184,9 | -2109 to 1740 | No | ns | >0,9999 | H-Q |
| **L.n vs. L.li** | -354,0 | -2278 to 1570 | No | ns | >0,9999 | H-R |
| **L.n vs. Control** | 88,87 | -1952 to 2130 | No | ns | >0,9999 | H-S |
| **L.n vs. LPS** | -71,30 | -2112 to 1970 | No | ns | >0,9999 | H-T |
| **L.n vs. S.a** | -6876 | -8918 to -4835 | Yes | **** | <0,0001 | H-U |
| **L.n vs. L.g** | 39,62 | -1885 to 1964 | No | ns | >0,9999 | H-V |
| **L.n vs. L.s** | -609,6 | -2534 to 1315 | No | ns | >0,9999 | H-W |
| **L.n vs. L.la** | -4927 | -6851 to -3002 | Yes | **** | <0,0001 | H-X |
| **L.n vs. L.b** | 30,24 | -1894 to 1955 | No | ns | >0,9999 | H-Y |
| **L.n vs. L.n** | 0,000 | -1924 to 1924 | No | ns | >0,9999 | H-Z |
| **L.n vs. L.li** | -32,74 | -1957 to 1892 | No | ns | >0,9999 | H-AA |
| **L.li vs. Control** | 123,2 | -1918 to 2164 | No | ns | >0,9999 | I-J |
| **L.li vs. LPS** | -3683 | -5724 to -1641 | Yes | **** | <0,0001 | I-K |
| **L.li vs. S.a** | -52,81 | -2094 to 1988 | No | ns | >0,9999 | I-L |
| **L.li vs. L.g** | 49,10 | -1875 to 1974 | No | ns | >0,9999 | I-M |
| **L.li vs. L.s** | -316,9 | -2241 to 1608 | No | ns | >0,9999 | I-N |
| **L.li vs. L.la** | -6120 | -8044 to -4196 | Yes | **** | <0,0001 | I-O |
| **L.li vs. L.b** | -27,63 | -1952 to 1897 | No | ns | >0,9999 | I-P |
| **L.li vs. L.n** | -152,1 | -2077 to 1772 | No | ns | >0,9999 | I-Q |
| **L.li vs. L.li** | -321,3 | -2246 to 1603 | No | ns | >0,9999 | I-R |
| **L.li vs. Control** | 121,6 | -1920 to 2163 | No | ns | >0,9999 | I-S |
| **L.li vs. LPS** | -38,56 | -2080 to 2003 | No | ns | >0,9999 | I-T |
| **L.li vs. S.a** | -6844 | -8885 to -4803 | Yes | **** | <0,0001 | I-U |
| **L.li vs. L.g** | 72,36 | -1852 to 1997 | No | ns | >0,9999 | I-V |
| **L.li vs. L.s** | -576,9 | -2501 to 1348 | No | ns | >0,9999 | I-W |
| **L.li vs. L.la** | -4894 | -6818 to -2969 | Yes | **** | <0,0001 | I-X |
| **L.li vs. L.b** | 62,98 | -1861 to 1987 | No | ns | >0,9999 | I-Y |
| **L.li vs. L.n** | 32,74 | -1892 to 1957 | No | ns | >0,9999 | I-Z |
| **L.li vs. L.li** | 0,000 | -1924 to 1924 | No | ns | >0,9999 | I-AA |
| **Control vs. LPS** | -3806 | -5957 to -1654 | Yes | **** | <0,0001 | J-K |
| **Control vs. S.a** | -176,0 | -2328 to 1976 | No | ns | >0,9999 | J-L |
| **Control vs. L.g** | -74,09 | -2115 to 1967 | No | ns | >0,9999 | J-M |
| **Control vs. L.s** | -440,1 | -2481 to 1601 | No | ns | >0,9999 | J-N |
| **Control vs. L.la** | -6243 | -8284 to -4202 | Yes | **** | <0,0001 | J-O |
| **Control vs. L.b** | -150,8 | -2192 to 1890 | No | ns | >0,9999 | J-P |
| **Control vs. L.n** | -275,3 | -2316 to 1766 | No | ns | >0,9999 | J-Q |
| **Control vs. L.li** | -444,5 | -2486 to 1597 | No | ns | >0,9999 | J-R |
| **Control vs. Control** | -1,585 | -2153 to 2150 | No | ns | >0,9999 | J-S |
| **Control vs. LPS** | -161,8 | -2313 to 1990 | No | ns | >0,9999 | J-T |
| **Control vs. S.a** | -6967 | -9118 to -4815 | Yes | **** | <0,0001 | J-U |
| **Control vs. L.g** | -50,83 | -2092 to 1990 | No | ns | >0,9999 | J-V |
| **Control vs. L.s** | -700,1 | -2741 to 1341 | No | ns | >0,9999 | J-W |
| **Control vs. L.la** | -5017 | -7058 to -2976 | Yes | **** | <0,0001 | J-X |
| **Control vs. L.b** | -60,21 | -2101 to 1981 | No | ns | >0,9999 | J-Y |
| **Control vs. L.n** | -90,45 | -2132 to 1951 | No | ns | >0,9999 | J-Z |
| **Control vs. L.li** | -123,2 | -2164 to 1918 | No | ns | >0,9999 | J-AA |
| **LPS vs. S.a** | 3630 | 1478 to 5781 | Yes | **** | <0,0001 | K-L |
| **LPS vs. L.g** | 3732 | 1691 to 5773 | Yes | **** | <0,0001 | K-M |
| **LPS vs. L.s** | 3366 | 1325 to 5407 | Yes | **** | <0,0001 | K-N |
| **LPS vs. L.la** | -2437 | -4479 to -396,2 | Yes | ** | 0,0042 | K-O |
| **LPS vs. L.b** | 3655 | 1614 to 5696 | Yes | **** | <0,0001 | K-P |
| **LPS vs. L.n** | 3530 | 1489 to 5572 | Yes | **** | <0,0001 | K-Q |
| **LPS vs. L.li** | 3361 | 1320 to 5402 | Yes | **** | <0,0001 | K-R |
| **LPS vs. Control** | 3804 | 1653 to 5956 | Yes | **** | <0,0001 | K-S |
| **LPS vs. LPS** | 3644 | 1492 to 5796 | Yes | **** | <0,0001 | K-T |
| **LPS vs. S.a** | -3161 | -5313 to -1010 | Yes | **** | <0,0001 | K-U |
| **LPS vs. L.g** | 3755 | 1714 to 5796 | Yes | **** | <0,0001 | K-V |
| **LPS vs. L.s** | 3106 | 1065 to 5147 | Yes | **** | <0,0001 | K-W |
| **LPS vs. L.la** | -1211 | -3252 to 830,0 | No | ns | 0,8698 | K-X |
| **LPS vs. L.b** | 3746 | 1704 to 5787 | Yes | **** | <0,0001 | K-Y |
| **LPS vs. L.n** | 3715 | 1674 to 5756 | Yes | **** | <0,0001 | K-Z |
| **LPS vs. L.li** | 3683 | 1641 to 5724 | Yes | **** | <0,0001 | K-AA |
| **S.a vs. L.g** | 101,9 | -1939 to 2143 | No | ns | >0,9999 | L-M |
| **S.a vs. L.s** | -264,1 | -2305 to 1777 | No | ns | >0,9999 | L-N |
| **S.a vs. L.la** | -6067 | -8108 to -4026 | Yes | **** | <0,0001 | L-O |
| **S.a vs. L.b** | 25,18 | -2016 to 2066 | No | ns | >0,9999 | L-P |
| **S.a vs. L.n** | -99,34 | -2140 to 1942 | No | ns | >0,9999 | L-Q |
| **S.a vs. L.li** | -268,5 | -2310 to 1773 | No | ns | >0,9999 | L-R |
| **S.a vs. Control** | 174,4 | -1977 to 2326 | No | ns | >0,9999 | L-S |
| **S.a vs. LPS** | 14,25 | -2137 to 2166 | No | ns | >0,9999 | L-T |
| **S.a vs. S.a** | -6791 | -8942 to -4639 | Yes | **** | <0,0001 | L-U |
| **S.a vs. L.g** | 125,2 | -1916 to 2166 | No | ns | >0,9999 | L-V |
| **S.a vs. L.s** | -524,1 | -2565 to 1517 | No | ns | >0,9999 | L-W |
| **S.a vs. L.la** | -4841 | -6882 to -2800 | Yes | **** | <0,0001 | L-X |
| **S.a vs. L.b** | 115,8 | -1925 to 2157 | No | ns | >0,9999 | L-Y |
| **S.a vs. L.n** | 85,55 | -1956 to 2127 | No | ns | >0,9999 | L-Z |
| **S.a vs. L.li** | 52,81 | -1988 to 2094 | No | ns | >0,9999 | L-AA |
| **L.g vs. L.s** | -366,0 | -2290 to 1558 | No | ns | >0,9999 | M-N |
| **L.g vs. L.la** | -6169 | -8093 to -4245 | Yes | **** | <0,0001 | M-O |
| **L.g vs. L.b** | -76,73 | -2001 to 1848 | No | ns | >0,9999 | M-P |
| **L.g vs. L.n** | -201,2 | -2126 to 1723 | No | ns | >0,9999 | M-Q |
| **L.g vs. L.li** | -370,4 | -2295 to 1554 | No | ns | >0,9999 | M-R |
| **L.g vs. Control** | 72,51 | -1969 to 2114 | No | ns | >0,9999 | M-S |
| **L.g vs. LPS** | -87,66 | -2129 to 1953 | No | ns | >0,9999 | M-T |
| **L.g vs. S.a** | -6893 | -8934 to -4852 | Yes | **** | <0,0001 | M-U |
| **L.g vs. L.g** | 23,26 | -1901 to 1948 | No | ns | >0,9999 | M-V |
| **L.g vs. L.s** | -626,0 | -2550 to 1298 | No | ns | >0,9999 | M-W |
| **L.g vs. L.la** | -4943 | -6867 to -3018 | Yes | **** | <0,0001 | M-X |
| **L.g vs. L.b** | 13,88 | -1911 to 1938 | No | ns | >0,9999 | M-Y |
| **L.g vs. L.n** | -16,36 | -1941 to 1908 | No | ns | >0,9999 | M-Z |
| **L.g vs. L.li** | -49,10 | -1974 to 1875 | No | ns | >0,9999 | M-AA |
| **L.s vs. L.la** | -5803 | -7728 to -3879 | Yes | **** | <0,0001 | N-O |
| **L.s vs. L.b** | 289,2 | -1635 to 2214 | No | ns | >0,9999 | N-P |
| **L.s vs. L.n** | 164,7 | -1760 to 2089 | No | ns | >0,9999 | N-Q |
| **L.s vs. L.li** | -4,396 | -1929 to 1920 | No | ns | >0,9999 | N-R |
| **L.s vs. Control** | 438,5 | -1603 to 2480 | No | ns | >0,9999 | N-S |
| **L.s vs. LPS** | 278,3 | -1763 to 2319 | No | ns | >0,9999 | N-T |
| **L.s vs. S.a** | -6527 | -8568 to -4486 | Yes | **** | <0,0001 | N-U |
| **L.s vs. L.g** | 389,2 | -1535 to 2314 | No | ns | >0,9999 | N-V |
| **L.s vs. L.s** | -260,0 | -2184 to 1664 | No | ns | >0,9999 | N-W |
| **L.s vs. L.la** | -4577 | -6501 to -2652 | Yes | **** | <0,0001 | N-X |
| **L.s vs. L.b** | 379,8 | -1545 to 2304 | No | ns | >0,9999 | N-Y |
| **L.s vs. L.n** | 349,6 | -1575 to 2274 | No | ns | >0,9999 | N-Z |
| **L.s vs. L.li** | 316,9 | -1608 to 2241 | No | ns | >0,9999 | N-AA |
| **L.la vs. L.b** | 6092 | 4168 to 8017 | Yes | **** | <0,0001 | O-P |
| **L.la vs. L.n** | 5968 | 4043 to 7892 | Yes | **** | <0,0001 | O-Q |
| **L.la vs. L.li** | 5799 | 3874 to 7723 | Yes | **** | <0,0001 | O-R |
| **L.la vs. Control** | 6242 | 4200 to 8283 | Yes | **** | <0,0001 | O-S |
| **L.la vs. LPS** | 6081 | 4040 to 8123 | Yes | **** | <0,0001 | O-T |
| **L.la vs. S.a** | -723,7 | -2765 to 1317 | No | ns | 0,9998 | O-U |
| **L.la vs. L.g** | 6192 | 4268 to 8117 | Yes | **** | <0,0001 | O-V |
| **L.la vs. L.s** | 5543 | 3619 to 7468 | Yes | **** | <0,0001 | O-W |
| **L.la vs. L.la** | 1226 | -698,2 to 3151 | No | ns | 0,7762 | O-X |
| **L.la vs. L.b** | 6183 | 4259 to 8107 | Yes | **** | <0,0001 | O-Y |
| **L.la vs. L.n** | 6153 | 4228 to 8077 | Yes | **** | <0,0001 | O-Z |
| **L.la vs. L.li** | 6120 | 4196 to 8044 | Yes | **** | <0,0001 | O-AA |
| **L.b vs. L.n** | -124,5 | -2049 to 1800 | No | ns | >0,9999 | P-Q |
| **L.b vs. L.li** | -293,6 | -2218 to 1631 | No | ns | >0,9999 | P-R |
| **L.b vs. Control** | 149,2 | -1892 to 2190 | No | ns | >0,9999 | P-S |
| **L.b vs. LPS** | -10,93 | -2052 to 2030 | No | ns | >0,9999 | P-T |
| **L.b vs. S.a** | -6816 | -8857 to -4775 | Yes | **** | <0,0001 | P-U |
| **L.b vs. L.g** | 99,99 | -1824 to 2024 | No | ns | >0,9999 | P-V |
| **L.b vs. L.s** | -549,2 | -2474 to 1375 | No | ns | >0,9999 | P-W |
| **L.b vs. L.la** | -4866 | -6791 to -2942 | Yes | **** | <0,0001 | P-X |
| **L.b vs. L.b** | 90,61 | -1834 to 2015 | No | ns | >0,9999 | P-Y |
| **L.b vs. L.n** | 60,37 | -1864 to 1985 | No | ns | >0,9999 | P-Z |
| **L.b vs. L.li** | 27,63 | -1897 to 1952 | No | ns | >0,9999 | P-AA |
| **L.n vs. L.li** | -169,1 | -2094 to 1755 | No | ns | >0,9999 | Q-R |
| **L.n vs. Control** | 273,8 | -1767 to 2315 | No | ns | >0,9999 | Q-S |
| **L.n vs. LPS** | 113,6 | -1928 to 2155 | No | ns | >0,9999 | Q-T |
| **L.n vs. S.a** | -6692 | -8733 to -4650 | Yes | **** | <0,0001 | Q-U |
| **L.n vs. L.g** | 224,5 | -1700 to 2149 | No | ns | >0,9999 | Q-V |
| **L.n vs. L.s** | -424,7 | -2349 to 1500 | No | ns | >0,9999 | Q-W |
| **L.n vs. L.la** | -4742 | -6666 to -2817 | Yes | **** | <0,0001 | Q-X |
| **L.n vs. L.b** | 215,1 | -1709 to 2140 | No | ns | >0,9999 | Q-Y |
| **L.n vs. L.n** | 184,9 | -1740 to 2109 | No | ns | >0,9999 | Q-Z |
| **L.n vs. L.li** | 152,1 | -1772 to 2077 | No | ns | >0,9999 | Q-AA |
| **L.li vs. Control** | 442,9 | -1598 to 2484 | No | ns | >0,9999 | R-S |
| **L.li vs. LPS** | 282,7 | -1758 to 2324 | No | ns | >0,9999 | R-T |
| **L.li vs. S.a** | -6522 | -8564 to -4481 | Yes | **** | <0,0001 | R-U |
| **L.li vs. L.g** | 393,6 | -1531 to 2318 | No | ns | >0,9999 | R-V |
| **L.li vs. L.s** | -255,6 | -2180 to 1669 | No | ns | >0,9999 | R-W |
| **L.li vs. L.la** | -4572 | -6497 to -2648 | Yes | **** | <0,0001 | R-X |
| **L.li vs. L.b** | 384,2 | -1540 to 2309 | No | ns | >0,9999 | R-Y |
| **L.li vs. L.n** | 354,0 | -1570 to 2278 | No | ns | >0,9999 | R-Z |
| **L.li vs. L.li** | 321,3 | -1603 to 2246 | No | ns | >0,9999 | R-AA |
| **Control vs. LPS** | -160,2 | -2312 to 1991 | No | ns | >0,9999 | S-T |
| **Control vs. S.a** | -6965 | -9117 to -4814 | Yes | **** | <0,0001 | S-U |
| **Control vs. L.g** | -49,25 | -2090 to 1992 | No | ns | >0,9999 | S-V |
| **Control vs. L.s** | -698,5 | -2740 to 1343 | No | ns | >0,9999 | S-W |
| **Control vs. L.la** | -5015 | -7057 to -2974 | Yes | **** | <0,0001 | S-X |
| **Control vs. L.b** | -58,63 | -2100 to 1983 | No | ns | >0,9999 | S-Y |
| **Control vs. L.n** | -88,87 | -2130 to 1952 | No | ns | >0,9999 | S-Z |
| **Control vs. L.li** | -121,6 | -2163 to 1920 | No | ns | >0,9999 | S-AA |
| **LPS vs. S.a** | -6805 | -8957 to -4654 | Yes | **** | <0,0001 | T-U |
| **LPS vs. L.g** | 110,9 | -1930 to 2152 | No | ns | >0,9999 | T-V |
| **LPS vs. L.s** | -538,3 | -2579 to 1503 | No | ns | >0,9999 | T-W |
| **LPS vs. L.la** | -4855 | -6896 to -2814 | Yes | **** | <0,0001 | T-X |
| **LPS vs. L.b** | 101,5 | -1940 to 2143 | No | ns | >0,9999 | T-Y |
| **LPS vs. L.n** | 71,30 | -1970 to 2112 | No | ns | >0,9999 | T-Z |
| **LPS vs. L.li** | 38,56 | -2003 to 2080 | No | ns | >0,9999 | T-AA |
| **S.a vs. L.g** | 6916 | 4875 to 8957 | Yes | **** | <0,0001 | U-V |
| **S.a vs. L.s** | 6267 | 4226 to 8308 | Yes | **** | <0,0001 | U-W |
| **S.a vs. L.la** | 1950 | -91,26 to 3991 | No | ns | 0,0816 | U-X |
| **S.a vs. L.b** | 6907 | 4865 to 8948 | Yes | **** | <0,0001 | U-Y |
| **S.a vs. L.n** | 6876 | 4835 to 8918 | Yes | **** | <0,0001 | U-Z |
| **S.a vs. L.li** | 6844 | 4803 to 8885 | Yes | **** | <0,0001 | U-AA |
| **L.g vs. L.s** | -649,2 | -2574 to 1275 | No | ns | >0,9999 | V-W |
| **L.g vs. L.la** | -4966 | -6891 to -3042 | Yes | **** | <0,0001 | V-X |
| **L.g vs. L.b** | -9,380 | -1934 to 1915 | No | ns | >0,9999 | V-Y |
| **L.g vs. L.n** | -39,62 | -1964 to 1885 | No | ns | >0,9999 | V-Z |
| **L.g vs. L.li** | -72,36 | -1997 to 1852 | No | ns | >0,9999 | V-AA |
| **L.s vs. L.la** | -4317 | -6241 to -2392 | Yes | **** | <0,0001 | W-X |
| **L.s vs. L.b** | 639,8 | -1285 to 2564 | No | ns | >0,9999 | W-Y |
| **L.s vs. L.n** | 609,6 | -1315 to 2534 | No | ns | >0,9999 | W-Z |
| **L.s vs. L.li** | 576,9 | -1348 to 2501 | No | ns | >0,9999 | W-AA |
| **L.la vs. L.b** | 4957 | 3032 to 6881 | Yes | **** | <0,0001 | X-Y |
| **L.la vs. L.n** | 4927 | 3002 to 6851 | Yes | **** | <0,0001 | X-Z |
| **L.la vs. L.li** | 4894 | 2969 to 6818 | Yes | **** | <0,0001 | X-AA |
| **L.b vs. L.n** | -30,24 | -1955 to 1894 | No | ns | >0,9999 | Y-Z |
| **L.b vs. L.li** | -62,98 | -1987 to 1861 | No | ns | >0,9999 | Y-AA |
| **L.n vs. L.li** | -32,74 | -1957 to 1892 | No | ns | >0,9999 | Z-AA |

**Raw data and statistical analysis of Figure 14:**

**IL-6 (pg/mL)**

**LPG (WT, TLR2KO and TLR4KO)**

| **Control** | **LPS** | **S.a** | **L.g** | **L.s** | **L.la** | **L.b** | **L.n** | **L.li** |
| --- | --- | --- | --- | --- | --- | --- | --- | --- |
| 4,9 | 1000 | 946,1 | 18 | 41,4 | 57,4 | 742,2 | 55 | 18,4 |
| 4,5 | 1584 | 955 | 14,8 | 29 | 49,4 | 623,2 | 58,1 | 36,6 |
| 5,9 | 1690 | 940,6 | 14,2 | 28,6 | 33,1 | 818,8 | 49,4 | 26,7 |
| 3 | 1992 | 786,2 | 23 | 27,5 | 62,1 | 1044 | 28,6 | 10,3 |
|  |  |  | 24,1 | 29,7 | 90,5 | 722,5 | 57 | 20,1 |
| **Control** | **LPS** | **S.a** | **L.g** | **L.s** | **L.la** | **L.b** | **L.n** | **L.li** |
| 3,16 | 2917 | 31,15 | 21,19 | 46,76 | 5940 | 45,64 | 36,71 | 34,49 |
| 5,66 | 2139 | 26,71 | 20,09 | 68 | 6670 | 40,06 | 49 | 28,93 |
| 5,66 | 4830 | 23,39 | 31,15 | 42,29 | 4885 | 30,04 | 76,92 | 35,6 |
| 5,66 | 3758 | 37,83 | 17,89 | 42,29 | 5812 | 7,95 | 50,11 | 13,49 |
|  |  |  | 17,89 | 13,49 | 5455 | 20,09 | 44,53 | 40,06 |
| **Control** | **LPS** | **S.a** | **L.g** | **L.s** | **L.la** | **L.b** | **L.n** | **L.li** |
| 1,08 | 13,23 | 2,076 | 1,08 | 6,84 | 6,65 | 3,74 | 4,9 | 60,48 |
| 0,89 | 10,53 | 1944 | 3,35 | 8,98 | 6,06 | 4,32 | 3,35 | 34,96 |
| 0 | 17,65 | 1767 | 3,74 | 7,04 | 4,13 | 0,89 | 5,29 | 23,16 |
| 0,34 | 11,88 | 1246 | 2,21 | 5,29 | 2,4 | 4,13 | 1,26 | 15,92 |
|  |  |  | 1,64 | 6,06 | 2,78 | 2,21 | 7,23 | 29,37 |

**Statistical analysis**

| **Tukey's multiple comparisons test** | **Mean Diff,** | **95,00% CI of diff,** | **Significant?** | **Summary** | **Adjusted P Value** |  |
| --- | --- | --- | --- | --- | --- | --- |
| **Control vs. LPS** | -1562 | -2358 to -765,6 | Yes | **** | <0,0001 | A-B |
| **Control vs. S.a** | -902,4 | -1699 to -106,1 | Yes | ** | 0,0097 | A-C |
| **Control vs. L.g** | -14,25 | -769,7 to 741,2 | No | ns | >0,9999 | A-D |
| **Control vs. L.s** | -26,67 | -782,1 to 728,8 | No | ns | >0,9999 | A-E |
| **Control vs. L.la** | -53,93 | -809,4 to 701,5 | No | ns | >0,9999 | A-F |
| **Control vs. L.b** | -785,6 | -1541 to -30,11 | Yes | * | 0,0314 | A-G |
| **Control vs. L.n** | -45,05 | -800,5 to 710,4 | No | ns | >0,9999 | A-H |
| **Control vs. L.li** | -17,85 | -773,3 to 737,6 | No | ns | >0,9999 | A-I |
| **Control vs. Control** | -0,4600 | -796,8 to 795,9 | No | ns | >0,9999 | A-J |
| **Control vs. LPS** | -3406 | -4203 to -2610 | Yes | **** | <0,0001 | A-K |
| **Control vs. S.a** | -25,20 | -821,5 to 771,1 | No | ns | >0,9999 | A-L |
| **Control vs. L.g** | -17,07 | -772,5 to 738,4 | No | ns | >0,9999 | A-M |
| **Control vs. L.s** | -37,99 | -793,4 to 717,5 | No | ns | >0,9999 | A-N |
| **Control vs. L.la** | -5748 | -6503 to -4992 | Yes | **** | <0,0001 | A-O |
| **Control vs. L.b** | -24,18 | -779,6 to 731,3 | No | ns | >0,9999 | A-P |
| **Control vs. L.n** | -46,88 | -802,3 to 708,6 | No | ns | >0,9999 | A-Q |
| **Control vs. L.li** | -25,94 | -781,4 to 729,5 | No | ns | >0,9999 | A-R |
| **Control vs. Control** | 3,998 | -792,3 to 800,3 | No | ns | >0,9999 | A-S |
| **Control vs. LPS** | -8,748 | -805,1 to 787,6 | No | ns | >0,9999 | A-T |
| **Control vs. S.a** | -1235 | -2032 to -438,9 | Yes | **** | <0,0001 | A-U |
| **Control vs. L.g** | 2,171 | -753,3 to 757,6 | No | ns | >0,9999 | A-V |
| **Control vs. L.s** | -2,267 | -757,7 to 753,2 | No | ns | >0,9999 | A-W |
| **Control vs. L.la** | 0,1710 | -755,3 to 755,6 | No | ns | >0,9999 | A-X |
| **Control vs. L.b** | 1,517 | -753,9 to 757,0 | No | ns | >0,9999 | A-Y |
| **Control vs. L.n** | 0,1690 | -755,3 to 755,6 | No | ns | >0,9999 | A-Z |
| **Control vs. L.li** | -28,20 | -783,7 to 727,3 | No | ns | >0,9999 | A-AA |
| **LPS vs. S.a** | 659,5 | -136,8 to 1456 | No | ns | 0,2669 | B-C |
| **LPS vs. L.g** | 1548 | 792,2 to 2303 | Yes | **** | <0,0001 | B-D |
| **LPS vs. L.s** | 1535 | 779,8 to 2291 | Yes | **** | <0,0001 | B-E |
| **LPS vs. L.la** | 1508 | 752,5 to 2263 | Yes | **** | <0,0001 | B-F |
| **LPS vs. L.b** | 776,4 | 20,90 to 1532 | Yes | * | 0,0363 | B-G |
| **LPS vs. L.n** | 1517 | 761,4 to 2272 | Yes | **** | <0,0001 | B-H |
| **LPS vs. L.li** | 1544 | 788,6 to 2300 | Yes | **** | <0,0001 | B-I |
| **LPS vs. Control** | 1561 | 765,1 to 2358 | Yes | **** | <0,0001 | B-J |
| **LPS vs. LPS** | -1845 | -2641 to -1048 | Yes | **** | <0,0001 | B-K |
| **LPS vs. S.a** | 1537 | 740,4 to 2333 | Yes | **** | <0,0001 | B-L |
| **LPS vs. L.g** | 1545 | 789,4 to 2300 | Yes | **** | <0,0001 | B-M |
| **LPS vs. L.s** | 1524 | 768,5 to 2279 | Yes | **** | <0,0001 | B-N |
| **LPS vs. L.la** | -4186 | -4941 to -3430 | Yes | **** | <0,0001 | B-O |
| **LPS vs. L.b** | 1538 | 782,3 to 2293 | Yes | **** | <0,0001 | B-P |
| **LPS vs. L.n** | 1515 | 759,6 to 2271 | Yes | **** | <0,0001 | B-Q |
| **LPS vs. L.li** | 1536 | 780,5 to 2291 | Yes | **** | <0,0001 | B-R |
| **LPS vs. Control** | 1566 | 769,6 to 2362 | Yes | **** | <0,0001 | B-S |
| **LPS vs. LPS** | 1553 | 756,9 to 2349 | Yes | **** | <0,0001 | B-T |
| **LPS vs. S.a** | 326,7 | -469,6 to 1123 | No | ns | 0,9984 | B-U |
| **LPS vs. L.g** | 1564 | 808,6 to 2320 | Yes | **** | <0,0001 | B-V |
| **LPS vs. L.s** | 1560 | 804,2 to 2315 | Yes | **** | <0,0001 | B-W |
| **LPS vs. L.la** | 1562 | 806,6 to 2318 | Yes | **** | <0,0001 | B-X |
| **LPS vs. L.b** | 1563 | 808,0 to 2319 | Yes | **** | <0,0001 | B-Y |
| **LPS vs. L.n** | 1562 | 806,6 to 2318 | Yes | **** | <0,0001 | B-Z |
| **LPS vs. L.li** | 1534 | 778,3 to 2289 | Yes | **** | <0,0001 | B-AA |
| **S.a vs. L.g** | 888,2 | 132,7 to 1644 | Yes | ** | 0,0055 | C-D |
| **S.a vs. L.s** | 875,7 | 120,3 to 1631 | Yes | ** | 0,0068 | C-E |
| **S.a vs. L.la** | 848,5 | 93,02 to 1604 | Yes | * | 0,0111 | C-F |
| **S.a vs. L.b** | 116,8 | -638,6 to 872,3 | No | ns | >0,9999 | C-G |
| **S.a vs. L.n** | 857,4 | 101,9 to 1613 | Yes | ** | 0,0095 | C-H |
| **S.a vs. L.li** | 884,6 | 129,1 to 1640 | Yes | ** | 0,0058 | C-I |
| **S.a vs. Control** | 901,9 | 105,6 to 1698 | Yes | ** | 0,0098 | C-J |
| **S.a vs. LPS** | -2504 | -3300 to -1708 | Yes | **** | <0,0001 | C-K |
| **S.a vs. S.a** | 877,2 | 80,88 to 1674 | Yes | * | 0,0146 | C-L |
| **S.a vs. L.g** | 885,3 | 129,9 to 1641 | Yes | ** | 0,0057 | C-M |
| **S.a vs. L.s** | 864,4 | 109,0 to 1620 | Yes | ** | 0,0084 | C-N |
| **S.a vs. L.la** | -4845 | -5601 to -4090 | Yes | **** | <0,0001 | C-O |
| **S.a vs. L.b** | 878,2 | 122,8 to 1634 | Yes | ** | 0,0065 | C-P |
| **S.a vs. L.n** | 855,5 | 100,1 to 1611 | Yes | ** | 0,0098 | C-Q |
| **S.a vs. L.li** | 876,5 | 121,0 to 1632 | Yes | ** | 0,0067 | C-R |
| **S.a vs. Control** | 906,4 | 110,1 to 1703 | Yes | ** | 0,0091 | C-S |
| **S.a vs. LPS** | 893,7 | 97,33 to 1690 | Yes | * | 0,0112 | C-T |
| **S.a vs. S.a** | -332,8 | -1129 to 463,5 | No | ns | 0,9978 | C-U |
| **S.a vs. L.g** | 904,6 | 149,1 to 1660 | Yes | ** | 0,0040 | C-V |
| **S.a vs. L.s** | 900,1 | 144,7 to 1656 | Yes | ** | 0,0044 | C-W |
| **S.a vs. L.la** | 902,6 | 147,1 to 1658 | Yes | ** | 0,0042 | C-X |
| **S.a vs. L.b** | 903,9 | 148,5 to 1659 | Yes | ** | 0,0041 | C-Y |
| **S.a vs. L.n** | 902,6 | 147,1 to 1658 | Yes | ** | 0,0042 | C-Z |
| **S.a vs. L.li** | 874,2 | 118,7 to 1630 | Yes | ** | 0,0070 | C-AA |
| **L.g vs. L.s** | -12,42 | -724,7 to 699,8 | No | ns | >0,9999 | D-E |
| **L.g vs. L.la** | -39,68 | -751,9 to 672,6 | No | ns | >0,9999 | D-F |
| **L.g vs. L.b** | -771,3 | -1484 to -59,07 | Yes | * | 0,0185 | D-G |
| **L.g vs. L.n** | -30,80 | -743,1 to 681,5 | No | ns | >0,9999 | D-H |
| **L.g vs. L.li** | -3,600 | -715,9 to 708,7 | No | ns | >0,9999 | D-I |
| **L.g vs. Control** | 13,79 | -741,7 to 769,2 | No | ns | >0,9999 | D-J |
| **L.g vs. LPS** | -3392 | -4148 to -2637 | Yes | **** | <0,0001 | D-K |
| **L.g vs. S.a** | -10,95 | -766,4 to 744,5 | No | ns | >0,9999 | D-L |
| **L.g vs. L.g** | -2,822 | -715,1 to 709,4 | No | ns | >0,9999 | D-M |
| **L.g vs. L.s** | -23,75 | -736,0 to 688,5 | No | ns | >0,9999 | D-N |
| **L.g vs. L.la** | -5734 | -6446 to -5021 | Yes | **** | <0,0001 | D-O |
| **L.g vs. L.b** | -9,936 | -722,2 to 702,3 | No | ns | >0,9999 | D-P |
| **L.g vs. L.n** | -32,63 | -744,9 to 679,6 | No | ns | >0,9999 | D-Q |
| **L.g vs. L.li** | -11,69 | -723,9 to 700,6 | No | ns | >0,9999 | D-R |
| **L.g vs. Control** | 18,24 | -737,2 to 773,7 | No | ns | >0,9999 | D-S |
| **L.g vs. LPS** | 5,498 | -750,0 to 761,0 | No | ns | >0,9999 | D-T |
| **L.g vs. S.a** | -1221 | -1976 to -465,5 | Yes | **** | <0,0001 | D-U |
| **L.g vs. L.g** | 16,42 | -695,8 to 728,7 | No | ns | >0,9999 | D-V |
| **L.g vs. L.s** | 11,98 | -700,3 to 724,2 | No | ns | >0,9999 | D-W |
| **L.g vs. L.la** | 14,42 | -697,8 to 726,7 | No | ns | >0,9999 | D-X |
| **L.g vs. L.b** | 15,76 | -696,5 to 728,0 | No | ns | >0,9999 | D-Y |
| **L.g vs. L.n** | 14,41 | -697,8 to 726,7 | No | ns | >0,9999 | D-Z |
| **L.g vs. L.li** | -13,96 | -726,2 to 698,3 | No | ns | >0,9999 | D-AA |
| **L.s vs. L.la** | -27,26 | -739,5 to 685,0 | No | ns | >0,9999 | E-F |
| **L.s vs. L.b** | -758,9 | -1471 to -46,65 | Yes | * | 0,0230 | E-G |
| **L.s vs. L.n** | -18,38 | -730,6 to 693,9 | No | ns | >0,9999 | E-H |
| **L.s vs. L.li** | 8,820 | -703,4 to 721,1 | No | ns | >0,9999 | E-I |
| **L.s vs. Control** | 26,21 | -729,3 to 781,7 | No | ns | >0,9999 | E-J |
| **L.s vs. LPS** | -3380 | -4135 to -2624 | Yes | **** | <0,0001 | E-K |
| **L.s vs. S.a** | 1,470 | -754,0 to 756,9 | No | ns | >0,9999 | E-L |
| **L.s vs. L.g** | 9,598 | -702,7 to 721,9 | No | ns | >0,9999 | E-M |
| **L.s vs. L.s** | -11,33 | -723,6 to 700,9 | No | ns | >0,9999 | E-N |
| **L.s vs. L.la** | -5721 | -6433 to -5009 | Yes | **** | <0,0001 | E-O |
| **L.s vs. L.b** | 2,484 | -709,8 to 714,7 | No | ns | >0,9999 | E-P |
| **L.s vs. L.n** | -20,21 | -732,5 to 692,0 | No | ns | >0,9999 | E-Q |
| **L.s vs. L.li** | 0,7260 | -711,5 to 713,0 | No | ns | >0,9999 | E-R |
| **L.s vs. Control** | 30,66 | -724,8 to 786,1 | No | ns | >0,9999 | E-S |
| **L.s vs. LPS** | 17,92 | -737,5 to 773,4 | No | ns | >0,9999 | E-T |
| **L.s vs. S.a** | -1209 | -1964 to -453,1 | Yes | **** | <0,0001 | E-U |
| **L.s vs. L.g** | 28,84 | -683,4 to 741,1 | No | ns | >0,9999 | E-V |
| **L.s vs. L.s** | 24,40 | -687,9 to 736,7 | No | ns | >0,9999 | E-W |
| **L.s vs. L.la** | 26,84 | -685,4 to 739,1 | No | ns | >0,9999 | E-X |
| **L.s vs. L.b** | 28,18 | -684,1 to 740,4 | No | ns | >0,9999 | E-Y |
| **L.s vs. L.n** | 26,83 | -685,4 to 739,1 | No | ns | >0,9999 | E-Z |
| **L.s vs. L.li** | -1,538 | -713,8 to 710,7 | No | ns | >0,9999 | E-AA |
| **L.la vs. L.b** | -731,6 | -1444 to -19,39 | Yes | * | 0,0365 | F-G |
| **L.la vs. L.n** | 8,880 | -703,4 to 721,1 | No | ns | >0,9999 | F-H |
| **L.la vs. L.li** | 36,08 | -676,2 to 748,3 | No | ns | >0,9999 | F-I |
| **L.la vs. Control** | 53,47 | -702,0 to 808,9 | No | ns | >0,9999 | F-J |
| **L.la vs. LPS** | -3353 | -4108 to -2597 | Yes | **** | <0,0001 | F-K |
| **L.la vs. S.a** | 28,73 | -726,7 to 784,2 | No | ns | >0,9999 | F-L |
| **L.la vs. L.g** | 36,86 | -675,4 to 749,1 | No | ns | >0,9999 | F-M |
| **L.la vs. L.s** | 15,93 | -696,3 to 728,2 | No | ns | >0,9999 | F-N |
| **L.la vs. L.la** | -5694 | -6406 to -4982 | Yes | **** | <0,0001 | F-O |
| **L.la vs. L.b** | 29,74 | -682,5 to 742,0 | No | ns | >0,9999 | F-P |
| **L.la vs. L.n** | 7,046 | -705,2 to 719,3 | No | ns | >0,9999 | F-Q |
| **L.la vs. L.li** | 27,99 | -684,3 to 740,2 | No | ns | >0,9999 | F-R |
| **L.la vs. Control** | 57,92 | -697,5 to 813,4 | No | ns | >0,9999 | F-S |
| **L.la vs. LPS** | 45,18 | -710,3 to 800,6 | No | ns | >0,9999 | F-T |
| **L.la vs. S.a** | -1181 | -1937 to -425,8 | Yes | **** | <0,0001 | F-U |
| **L.la vs. L.g** | 56,10 | -656,2 to 768,3 | No | ns | >0,9999 | F-V |
| **L.la vs. L.s** | 51,66 | -660,6 to 763,9 | No | ns | >0,9999 | F-W |
| **L.la vs. L.la** | 54,10 | -658,2 to 766,3 | No | ns | >0,9999 | F-X |
| **L.la vs. L.b** | 55,44 | -656,8 to 767,7 | No | ns | >0,9999 | F-Y |
| **L.la vs. L.n** | 54,09 | -658,2 to 766,3 | No | ns | >0,9999 | F-Z |
| **L.la vs. L.li** | 25,72 | -686,5 to 738,0 | No | ns | >0,9999 | F-AA |
| **L.b vs. L.n** | 740,5 | 28,27 to 1453 | Yes | * | 0,0315 | G-H |
| **L.b vs. L.li** | 767,7 | 55,47 to 1480 | Yes | * | 0,0197 | G-I |
| **L.b vs. Control** | 785,1 | 29,65 to 1541 | Yes | * | 0,0317 | G-J |
| **L.b vs. LPS** | -2621 | -3376 to -1865 | Yes | **** | <0,0001 | G-K |
| **L.b vs. S.a** | 760,4 | 4,912 to 1516 | Yes | * | 0,0464 | G-L |
| **L.b vs. L.g** | 768,5 | 56,25 to 1481 | Yes | * | 0,0195 | G-M |
| **L.b vs. L.s** | 747,6 | 35,32 to 1460 | Yes | * | 0,0280 | G-N |
| **L.b vs. L.la** | -4962 | -5675 to -4250 | Yes | **** | <0,0001 | G-O |
| **L.b vs. L.b** | 761,4 | 49,13 to 1474 | Yes | * | 0,0220 | G-P |
| **L.b vs. L.n** | 738,7 | 26,43 to 1451 | Yes | * | 0,0325 | G-Q |
| **L.b vs. L.li** | 759,6 | 47,37 to 1472 | Yes | * | 0,0227 | G-R |
| **L.b vs. Control** | 789,6 | 34,10 to 1545 | Yes | * | 0,0295 | G-S |
| **L.b vs. LPS** | 776,8 | 21,36 to 1532 | Yes | * | 0,0361 | G-T |
| **L.b vs. S.a** | -449,6 | -1205 to 305,8 | No | ns | 0,8665 | G-U |
| **L.b vs. L.g** | 787,7 | 75,48 to 1500 | Yes | * | 0,0138 | G-V |
| **L.b vs. L.s** | 783,3 | 71,05 to 1496 | Yes | * | 0,0150 | G-W |
| **L.b vs. L.la** | 785,7 | 73,48 to 1498 | Yes | * | 0,0143 | G-X |
| **L.b vs. L.b** | 787,1 | 74,83 to 1499 | Yes | * | 0,0140 | G-Y |
| **L.b vs. L.n** | 785,7 | 73,48 to 1498 | Yes | * | 0,0143 | G-Z |
| **L.b vs. L.li** | 757,4 | 45,11 to 1470 | Yes | * | 0,0236 | G-AA |
| **L.n vs. L.li** | 27,20 | -685,1 to 739,5 | No | ns | >0,9999 | H-I |
| **L.n vs. Control** | 44,59 | -710,9 to 800,0 | No | ns | >0,9999 | H-J |
| **L.n vs. LPS** | -3361 | -4117 to -2606 | Yes | **** | <0,0001 | H-K |
| **L.n vs. S.a** | 19,85 | -735,6 to 775,3 | No | ns | >0,9999 | H-L |
| **L.n vs. L.g** | 27,98 | -684,3 to 740,2 | No | ns | >0,9999 | H-M |
| **L.n vs. L.s** | 7,054 | -705,2 to 719,3 | No | ns | >0,9999 | H-N |
| **L.n vs. L.la** | -5703 | -6415 to -4991 | Yes | **** | <0,0001 | H-O |
| **L.n vs. L.b** | 20,86 | -691,4 to 733,1 | No | ns | >0,9999 | H-P |
| **L.n vs. L.n** | -1,834 | -714,1 to 710,4 | No | ns | >0,9999 | H-Q |
| **L.n vs. L.li** | 19,11 | -693,1 to 731,4 | No | ns | >0,9999 | H-R |
| **L.n vs. Control** | 49,04 | -706,4 to 804,5 | No | ns | >0,9999 | H-S |
| **L.n vs. LPS** | 36,30 | -719,2 to 791,8 | No | ns | >0,9999 | H-T |
| **L.n vs. S.a** | -1190 | -1946 to -434,7 | Yes | **** | <0,0001 | H-U |
| **L.n vs. L.g** | 47,22 | -665,0 to 759,5 | No | ns | >0,9999 | H-V |
| **L.n vs. L.s** | 42,78 | -669,5 to 755,0 | No | ns | >0,9999 | H-W |
| **L.n vs. L.la** | 45,22 | -667,0 to 757,5 | No | ns | >0,9999 | H-X |
| **L.n vs. L.b** | 46,56 | -665,7 to 758,8 | No | ns | >0,9999 | H-Y |
| **L.n vs. L.n** | 45,21 | -667,0 to 757,5 | No | ns | >0,9999 | H-Z |
| **L.n vs. L.li** | 16,84 | -695,4 to 729,1 | No | ns | >0,9999 | H-AA |
| **L.li vs. Control** | 17,39 | -738,1 to 772,8 | No | ns | >0,9999 | I-J |
| **L.li vs. LPS** | -3389 | -4144 to -2633 | Yes | **** | <0,0001 | I-K |
| **L.li vs. S.a** | -7,350 | -762,8 to 748,1 | No | ns | >0,9999 | I-L |
| **L.li vs. L.g** | 0,7780 | -711,5 to 713,0 | No | ns | >0,9999 | I-M |
| **L.li vs. L.s** | -20,15 | -732,4 to 692,1 | No | ns | >0,9999 | I-N |
| **L.li vs. L.la** | -5730 | -6442 to -5018 | Yes | **** | <0,0001 | I-O |
| **L.li vs. L.b** | -6,336 | -718,6 to 705,9 | No | ns | >0,9999 | I-P |
| **L.li vs. L.n** | -29,03 | -741,3 to 683,2 | No | ns | >0,9999 | I-Q |
| **L.li vs. L.li** | -8,094 | -720,3 to 704,2 | No | ns | >0,9999 | I-R |
| **L.li vs. Control** | 21,84 | -733,6 to 777,3 | No | ns | >0,9999 | I-S |
| **L.li vs. LPS** | 9,098 | -746,4 to 764,6 | No | ns | >0,9999 | I-T |
| **L.li vs. S.a** | -1217 | -1973 to -461,9 | Yes | **** | <0,0001 | I-U |
| **L.li vs. L.g** | 20,02 | -692,2 to 732,3 | No | ns | >0,9999 | I-V |
| **L.li vs. L.s** | 15,58 | -696,7 to 727,8 | No | ns | >0,9999 | I-W |
| **L.li vs. L.la** | 18,02 | -694,2 to 730,3 | No | ns | >0,9999 | I-X |
| **L.li vs. L.b** | 19,36 | -692,9 to 731,6 | No | ns | >0,9999 | I-Y |
| **L.li vs. L.n** | 18,01 | -694,2 to 730,3 | No | ns | >0,9999 | I-Z |
| **L.li vs. L.li** | -10,36 | -722,6 to 701,9 | No | ns | >0,9999 | I-AA |
| **Control vs. LPS** | -3406 | -4202 to -2610 | Yes | **** | <0,0001 | J-K |
| **Control vs. S.a** | -24,74 | -821,1 to 771,6 | No | ns | >0,9999 | J-L |
| **Control vs. L.g** | -16,61 | -772,1 to 738,9 | No | ns | >0,9999 | J-M |
| **Control vs. L.s** | -37,53 | -793,0 to 717,9 | No | ns | >0,9999 | J-N |
| **Control vs. L.la** | -5747 | -6503 to -4992 | Yes | **** | <0,0001 | J-O |
| **Control vs. L.b** | -23,72 | -779,2 to 731,7 | No | ns | >0,9999 | J-P |
| **Control vs. L.n** | -46,42 | -801,9 to 709,0 | No | ns | >0,9999 | J-Q |
| **Control vs. L.li** | -25,48 | -780,9 to 730,0 | No | ns | >0,9999 | J-R |
| **Control vs. Control** | 4,458 | -791,9 to 800,8 | No | ns | >0,9999 | J-S |
| **Control vs. LPS** | -8,288 | -804,6 to 788,0 | No | ns | >0,9999 | J-T |
| **Control vs. S.a** | -1235 | -2031 to -438,4 | Yes | **** | <0,0001 | J-U |
| **Control vs. L.g** | 2,631 | -752,8 to 758,1 | No | ns | >0,9999 | J-V |
| **Control vs. L.s** | -1,807 | -757,3 to 753,7 | No | ns | >0,9999 | J-W |
| **Control vs. L.la** | 0,6310 | -754,8 to 756,1 | No | ns | >0,9999 | J-X |
| **Control vs. L.b** | 1,977 | -753,5 to 757,4 | No | ns | >0,9999 | J-Y |
| **Control vs. L.n** | 0,6290 | -754,8 to 756,1 | No | ns | >0,9999 | J-Z |
| **Control vs. L.li** | -27,74 | -783,2 to 727,7 | No | ns | >0,9999 | J-AA |
| **LPS vs. S.a** | 3381 | 2585 to 4178 | Yes | **** | <0,0001 | K-L |
| **LPS vs. L.g** | 3389 | 2634 to 4145 | Yes | **** | <0,0001 | K-M |
| **LPS vs. L.s** | 3368 | 2613 to 4124 | Yes | **** | <0,0001 | K-N |
| **LPS vs. L.la** | -2341 | -3097 to -1586 | Yes | **** | <0,0001 | K-O |
| **LPS vs. L.b** | 3382 | 2627 to 4138 | Yes | **** | <0,0001 | K-P |
| **LPS vs. L.n** | 3360 | 2604 to 4115 | Yes | **** | <0,0001 | K-Q |
| **LPS vs. L.li** | 3380 | 2625 to 4136 | Yes | **** | <0,0001 | K-R |
| **LPS vs. Control** | 3410 | 2614 to 4207 | Yes | **** | <0,0001 | K-S |
| **LPS vs. LPS** | 3398 | 2601 to 4194 | Yes | **** | <0,0001 | K-T |
| **LPS vs. S.a** | 2171 | 1375 to 2968 | Yes | **** | <0,0001 | K-U |
| **LPS vs. L.g** | 3409 | 2653 to 4164 | Yes | **** | <0,0001 | K-V |
| **LPS vs. L.s** | 3404 | 2649 to 4160 | Yes | **** | <0,0001 | K-W |
| **LPS vs. L.la** | 3407 | 2651 to 4162 | Yes | **** | <0,0001 | K-X |
| **LPS vs. L.b** | 3408 | 2652 to 4163 | Yes | **** | <0,0001 | K-Y |
| **LPS vs. L.n** | 3407 | 2651 to 4162 | Yes | **** | <0,0001 | K-Z |
| **LPS vs. L.li** | 3378 | 2623 to 4134 | Yes | **** | <0,0001 | K-AA |
| **S.a vs. L.g** | 8,128 | -747,3 to 763,6 | No | ns | >0,9999 | L-M |
| **S.a vs. L.s** | -12,80 | -768,3 to 742,7 | No | ns | >0,9999 | L-N |
| **S.a vs. L.la** | -5723 | -6478 to -4967 | Yes | **** | <0,0001 | L-O |
| **S.a vs. L.b** | 1,014 | -754,4 to 756,5 | No | ns | >0,9999 | L-P |
| **S.a vs. L.n** | -21,68 | -777,1 to 733,8 | No | ns | >0,9999 | L-Q |
| **S.a vs. L.li** | -0,7440 | -756,2 to 754,7 | No | ns | >0,9999 | L-R |
| **S.a vs. Control** | 29,19 | -767,1 to 825,5 | No | ns | >0,9999 | L-S |
| **S.a vs. LPS** | 16,45 | -779,9 to 812,8 | No | ns | >0,9999 | L-T |
| **S.a vs. S.a** | -1210 | -2006 to -413,7 | Yes | **** | <0,0001 | L-U |
| **S.a vs. L.g** | 27,37 | -728,1 to 782,8 | No | ns | >0,9999 | L-V |
| **S.a vs. L.s** | 22,93 | -732,5 to 778,4 | No | ns | >0,9999 | L-W |
| **S.a vs. L.la** | 25,37 | -730,1 to 780,8 | No | ns | >0,9999 | L-X |
| **S.a vs. L.b** | 26,71 | -728,7 to 782,2 | No | ns | >0,9999 | L-Y |
| **S.a vs. L.n** | 25,36 | -730,1 to 780,8 | No | ns | >0,9999 | L-Z |
| **S.a vs. L.li** | -3,008 | -758,5 to 752,4 | No | ns | >0,9999 | L-AA |
| **L.g vs. L.s** | -20,92 | -733,2 to 691,3 | No | ns | >0,9999 | M-N |
| **L.g vs. L.la** | -5731 | -6443 to -5019 | Yes | **** | <0,0001 | M-O |
| **L.g vs. L.b** | -7,114 | -719,4 to 705,1 | No | ns | >0,9999 | M-P |
| **L.g vs. L.n** | -29,81 | -742,1 to 682,4 | No | ns | >0,9999 | M-Q |
| **L.g vs. L.li** | -8,872 | -721,1 to 703,4 | No | ns | >0,9999 | M-R |
| **L.g vs. Control** | 21,06 | -734,4 to 776,5 | No | ns | >0,9999 | M-S |
| **L.g vs. LPS** | 8,320 | -747,1 to 763,8 | No | ns | >0,9999 | M-T |
| **L.g vs. S.a** | -1218 | -1974 to -462,7 | Yes | **** | <0,0001 | M-U |
| **L.g vs. L.g** | 19,24 | -693,0 to 731,5 | No | ns | >0,9999 | M-V |
| **L.g vs. L.s** | 14,80 | -697,5 to 727,1 | No | ns | >0,9999 | M-W |
| **L.g vs. L.la** | 17,24 | -695,0 to 729,5 | No | ns | >0,9999 | M-X |
| **L.g vs. L.b** | 18,58 | -693,7 to 730,8 | No | ns | >0,9999 | M-Y |
| **L.g vs. L.n** | 17,24 | -695,0 to 729,5 | No | ns | >0,9999 | M-Z |
| **L.g vs. L.li** | -11,14 | -723,4 to 701,1 | No | ns | >0,9999 | M-AA |
| **L.s vs. L.la** | -5710 | -6422 to -4998 | Yes | **** | <0,0001 | N-O |
| **L.s vs. L.b** | 13,81 | -698,4 to 726,1 | No | ns | >0,9999 | N-P |
| **L.s vs. L.n** | -8,888 | -721,1 to 703,4 | No | ns | >0,9999 | N-Q |
| **L.s vs. L.li** | 12,05 | -700,2 to 724,3 | No | ns | >0,9999 | N-R |
| **L.s vs. Control** | 41,99 | -713,5 to 797,4 | No | ns | >0,9999 | N-S |
| **L.s vs. LPS** | 29,24 | -726,2 to 784,7 | No | ns | >0,9999 | N-T |
| **L.s vs. S.a** | -1197 | -1953 to -441,7 | Yes | **** | <0,0001 | N-U |
| **L.s vs. L.g** | 40,16 | -672,1 to 752,4 | No | ns | >0,9999 | N-V |
| **L.s vs. L.s** | 35,72 | -676,5 to 748,0 | No | ns | >0,9999 | N-W |
| **L.s vs. L.la** | 38,16 | -674,1 to 750,4 | No | ns | >0,9999 | N-X |
| **L.s vs. L.b** | 39,51 | -672,7 to 751,8 | No | ns | >0,9999 | N-Y |
| **L.s vs. L.n** | 38,16 | -674,1 to 750,4 | No | ns | >0,9999 | N-Z |
| **L.s vs. L.li** | 9,788 | -702,5 to 722,0 | No | ns | >0,9999 | N-AA |
| **L.la vs. L.b** | 5724 | 5011 to 6436 | Yes | **** | <0,0001 | O-P |
| **L.la vs. L.n** | 5701 | 4989 to 6413 | Yes | **** | <0,0001 | O-Q |
| **L.la vs. L.li** | 5722 | 5010 to 6434 | Yes | **** | <0,0001 | O-R |
| **L.la vs. Control** | 5752 | 4996 to 6507 | Yes | **** | <0,0001 | O-S |
| **L.la vs. LPS** | 5739 | 4984 to 6495 | Yes | **** | <0,0001 | O-T |
| **L.la vs. S.a** | 4513 | 3757 to 5268 | Yes | **** | <0,0001 | O-U |
| **L.la vs. L.g** | 5750 | 5038 to 6462 | Yes | **** | <0,0001 | O-V |
| **L.la vs. L.s** | 5746 | 5033 to 6458 | Yes | **** | <0,0001 | O-W |
| **L.la vs. L.la** | 5748 | 5036 to 6460 | Yes | **** | <0,0001 | O-X |
| **L.la vs. L.b** | 5749 | 5037 to 6462 | Yes | **** | <0,0001 | O-Y |
| **L.la vs. L.n** | 5748 | 5036 to 6460 | Yes | **** | <0,0001 | O-Z |
| **L.la vs. L.li** | 5720 | 5007 to 6432 | Yes | **** | <0,0001 | O-AA |
| **L.b vs. L.n** | -22,70 | -735,0 to 689,6 | No | ns | >0,9999 | P-Q |
| **L.b vs. L.li** | -1,758 | -714,0 to 710,5 | No | ns | >0,9999 | P-R |
| **L.b vs. Control** | 28,18 | -727,3 to 783,6 | No | ns | >0,9999 | P-S |
| **L.b vs. LPS** | 15,43 | -740,0 to 770,9 | No | ns | >0,9999 | P-T |
| **L.b vs. S.a** | -1211 | -1966 to -455,6 | Yes | **** | <0,0001 | P-U |
| **L.b vs. L.g** | 26,35 | -685,9 to 738,6 | No | ns | >0,9999 | P-V |
| **L.b vs. L.s** | 21,91 | -690,3 to 734,2 | No | ns | >0,9999 | P-W |
| **L.b vs. L.la** | 24,35 | -687,9 to 736,6 | No | ns | >0,9999 | P-X |
| **L.b vs. L.b** | 25,70 | -686,6 to 738,0 | No | ns | >0,9999 | P-Y |
| **L.b vs. L.n** | 24,35 | -687,9 to 736,6 | No | ns | >0,9999 | P-Z |
| **L.b vs. L.li** | -4,022 | -716,3 to 708,2 | No | ns | >0,9999 | P-AA |
| **L.n vs. L.li** | 20,94 | -691,3 to 733,2 | No | ns | >0,9999 | Q-R |
| **L.n vs. Control** | 50,88 | -704,6 to 806,3 | No | ns | >0,9999 | Q-S |
| **L.n vs. LPS** | 38,13 | -717,3 to 793,6 | No | ns | >0,9999 | Q-T |
| **L.n vs. S.a** | -1188 | -1944 to -432,9 | Yes | **** | <0,0001 | Q-U |
| **L.n vs. L.g** | 49,05 | -663,2 to 761,3 | No | ns | >0,9999 | Q-V |
| **L.n vs. L.s** | 44,61 | -667,6 to 756,9 | No | ns | >0,9999 | Q-W |
| **L.n vs. L.la** | 47,05 | -665,2 to 759,3 | No | ns | >0,9999 | Q-X |
| **L.n vs. L.b** | 48,40 | -663,9 to 760,6 | No | ns | >0,9999 | Q-Y |
| **L.n vs. L.n** | 47,05 | -665,2 to 759,3 | No | ns | >0,9999 | Q-Z |
| **L.n vs. L.li** | 18,68 | -693,6 to 730,9 | No | ns | >0,9999 | Q-AA |
| **L.li vs. Control** | 29,94 | -725,5 to 785,4 | No | ns | >0,9999 | R-S |
| **L.li vs. LPS** | 17,19 | -738,3 to 772,6 | No | ns | >0,9999 | R-T |
| **L.li vs. S.a** | -1209 | -1965 to -453,8 | Yes | **** | <0,0001 | R-U |
| **L.li vs. L.g** | 28,11 | -684,1 to 740,4 | No | ns | >0,9999 | R-V |
| **L.li vs. L.s** | 23,67 | -688,6 to 735,9 | No | ns | >0,9999 | R-W |
| **L.li vs. L.la** | 26,11 | -686,1 to 738,4 | No | ns | >0,9999 | R-X |
| **L.li vs. L.b** | 27,46 | -684,8 to 739,7 | No | ns | >0,9999 | R-Y |
| **L.li vs. L.n** | 26,11 | -686,1 to 738,4 | No | ns | >0,9999 | R-Z |
| **L.li vs. L.li** | -2,264 | -714,5 to 710,0 | No | ns | >0,9999 | R-AA |
| **Control vs. LPS** | -12,75 | -809,1 to 783,6 | No | ns | >0,9999 | S-T |
| **Control vs. S.a** | -1239 | -2036 to -442,9 | Yes | **** | <0,0001 | S-U |
| **Control vs. L.g** | -1,827 | -757,3 to 753,6 | No | ns | >0,9999 | S-V |
| **Control vs. L.s** | -6,265 | -761,7 to 749,2 | No | ns | >0,9999 | S-W |
| **Control vs. L.la** | -3,827 | -759,3 to 751,6 | No | ns | >0,9999 | S-X |
| **Control vs. L.b** | -2,481 | -757,9 to 753,0 | No | ns | >0,9999 | S-Y |
| **Control vs. L.n** | -3,829 | -759,3 to 751,6 | No | ns | >0,9999 | S-Z |
| **Control vs. L.li** | -32,20 | -787,7 to 723,3 | No | ns | >0,9999 | S-AA |
| **LPS vs. S.a** | -1226 | -2023 to -430,1 | Yes | **** | <0,0001 | T-U |
| **LPS vs. L.g** | 10,92 | -744,5 to 766,4 | No | ns | >0,9999 | T-V |
| **LPS vs. L.s** | 6,481 | -749,0 to 761,9 | No | ns | >0,9999 | T-W |
| **LPS vs. L.la** | 8,919 | -746,5 to 764,4 | No | ns | >0,9999 | T-X |
| **LPS vs. L.b** | 10,26 | -745,2 to 765,7 | No | ns | >0,9999 | T-Y |
| **LPS vs. L.n** | 8,917 | -746,5 to 764,4 | No | ns | >0,9999 | T-Z |
| **LPS vs. L.li** | -19,46 | -774,9 to 736,0 | No | ns | >0,9999 | T-AA |
| **S.a vs. L.g** | 1237 | 481,9 to 1993 | Yes | **** | <0,0001 | U-V |
| **S.a vs. L.s** | 1233 | 477,5 to 1988 | Yes | **** | <0,0001 | U-W |
| **S.a vs. L.la** | 1235 | 479,9 to 1991 | Yes | **** | <0,0001 | U-X |
| **S.a vs. L.b** | 1237 | 481,3 to 1992 | Yes | **** | <0,0001 | U-Y |
| **S.a vs. L.n** | 1235 | 479,9 to 1991 | Yes | **** | <0,0001 | U-Z |
| **S.a vs. L.li** | 1207 | 451,5 to 1962 | Yes | **** | <0,0001 | U-AA |
| **L.g vs. L.s** | -4,438 | -716,7 to 707,8 | No | ns | >0,9999 | V-W |
| **L.g vs. L.la** | -2,000 | -714,3 to 710,3 | No | ns | >0,9999 | V-X |
| **L.g vs. L.b** | -0,6540 | -712,9 to 711,6 | No | ns | >0,9999 | V-Y |
| **L.g vs. L.n** | -2,002 | -714,3 to 710,3 | No | ns | >0,9999 | V-Z |
| **L.g vs. L.li** | -30,37 | -742,6 to 681,9 | No | ns | >0,9999 | V-AA |
| **L.s vs. L.la** | 2,438 | -709,8 to 714,7 | No | ns | >0,9999 | W-X |
| **L.s vs. L.b** | 3,784 | -708,5 to 716,0 | No | ns | >0,9999 | W-Y |
| **L.s vs. L.n** | 2,436 | -709,8 to 714,7 | No | ns | >0,9999 | W-Z |
| **L.s vs. L.li** | -25,94 | -738,2 to 686,3 | No | ns | >0,9999 | W-AA |
| **L.la vs. L.b** | 1,346 | -710,9 to 713,6 | No | ns | >0,9999 | X-Y |
| **L.la vs. L.n** | -0,002000 | -712,3 to 712,3 | No | ns | >0,9999 | X-Z |
| **L.la vs. L.li** | -28,37 | -740,6 to 683,9 | No | ns | >0,9999 | X-AA |
| **L.b vs. L.n** | -1,348 | -713,6 to 710,9 | No | ns | >0,9999 | Y-Z |
| **L.b vs. L.li** | -29,72 | -742,0 to 682,5 | No | ns | >0,9999 | Y-AA |
| **L.n vs. L.li** | -28,37 | -740,6 to 683,9 | No | ns | >0,9999 | Z-AA |

**Raw data and statistical analysis of Figure 15:**

**IL-12 (pg/mL)**

**LPG (WT, TLR2KO and TLR4KO)**

| **Control** | **LPS** | **S.a** | **L.g** | **L.s** | **L.la** | **L.b** | **L.n** | **L.li** |
| --- | --- | --- | --- | --- | --- | --- | --- | --- |
| 0 | 32 | 86,3 | 32 | 12,5 | 16,4 | 5,32 | 0 | 12,6 |
| 6,4 | 65,7 | 79,4 | 28 | 14,5 | 23 | 7,6 | 0 | 0 |
| 0 | 65,7 | 65,7 | 19,7 | 18,7 | 16,4 | 5 | 3,4 | 0 |
| 6,4 | 76 | 48,7 | 22,3 | 9 | 16,4 | 6,1 | 9,5 | 0 |
|  |  |  | 27 | 7,9 | 12,6 | 4,1 | 0 | 3,4 |
| **Control** | **LPS** | **S.a** | **L.g** | **L.s** | **L.la** | **L.b** | **L.n** | **L.li** |
| 0 | 65 | 6,28 | 21,13 | 0 | 21,28 | 4 | 1,13 | 9,66 |
| 0 | 56 | 7,32 | 45 | 0 | 9,66 | 6 | 9,66 | 0 |
| 0 | 45 | 5,26 | 45 | 21,28 | 9,66 | 21,28 | 1,13 | 1,13 |
| 9,66 | 55 | 6,13 | 48 | 1,13 | 21,28 | 9,66 | 0 | 1,13 |
|  |  |  | 35 | 21,28 | 35,26 | 12 | 0 | 0 |
| **Control** | **LPS** | **S.a** | **L.g** | **L.s** | **L.la** | **L.b** | **L.n** | **L.li** |
| 0 | 0 | 55,1 | 15,71 | 0 | 0 | 0 | 0 | 0 |
| 0 | 6,43 | 62 | 0 | 3,43 | 0 | 0 | 0 | 0 |
| 0 | 7 | 63 | 6,43 | 0 | 0 | 3,43 | 6,43 | 0 |
| 0 | 3,43 | 55 | 0 | 0 | 0 | 0 | 9,47 | 6,43 |
|  |  |  | 9,47 | 0 | 0 | 3,43 | 3,43 | 0 |

**Statistical analysis**

| **Tukey's multiple comparisons test** | **Mean Diff,** | **95,00% CI of diff,** | **Significant?** | **Summary** | **Adjusted P Value** |  |
| --- | --- | --- | --- | --- | --- | --- |
| **Control vs. LPS** | -56,65 | -75,45 to -37,85 | Yes | **** | <0,0001 | A-B |
| **Control vs. S.a** | -66,83 | -85,63 to -48,02 | Yes | **** | <0,0001 | A-C |
| **Control vs. L.g** | -22,60 | -40,44 to -4,760 | Yes | ** | 0,0015 | A-D |
| **Control vs. L.s** | -9,320 | -27,16 to 8,520 | No | ns | 0,9615 | A-E |
| **Control vs. L.la** | -13,76 | -31,60 to 4,080 | No | ns | 0,4042 | A-F |
| **Control vs. L.b** | -2,424 | -20,26 to 15,42 | No | ns | >0,9999 | A-G |
| **Control vs. L.n** | 0,6200 | -17,22 to 18,46 | No | ns | >0,9999 | A-H |
| **Control vs. L.li** | 0,000 | -17,84 to 17,84 | No | ns | >0,9999 | A-I |
| **Control vs. Control** | 0,7850 | -18,02 to 19,59 | No | ns | >0,9999 | A-J |
| **Control vs. LPS** | -52,05 | -70,85 to -33,25 | Yes | **** | <0,0001 | A-K |
| **Control vs. S.a** | -3,048 | -21,85 to 15,76 | No | ns | >0,9999 | A-L |
| **Control vs. L.g** | -35,63 | -53,47 to -17,79 | Yes | **** | <0,0001 | A-M |
| **Control vs. L.s** | -5,538 | -23,38 to 12,30 | No | ns | >0,9999 | A-N |
| **Control vs. L.la** | -16,23 | -34,07 to 1,612 | No | ns | 0,1297 | A-O |
| **Control vs. L.b** | -7,388 | -25,23 to 10,45 | No | ns | 0,9981 | A-P |
| **Control vs. L.n** | 0,8160 | -17,02 to 18,66 | No | ns | >0,9999 | A-Q |
| **Control vs. L.li** | 0,8160 | -17,02 to 18,66 | No | ns | >0,9999 | A-R |
| **Control vs. Control** | 3,200 | -15,60 to 22,00 | No | ns | >0,9999 | A-S |
| **Control vs. LPS** | -1,015 | -19,82 to 17,79 | No | ns | >0,9999 | A-T |
| **Control vs. S.a** | -55,58 | -74,38 to -36,77 | Yes | **** | <0,0001 | A-U |
| **Control vs. L.g** | -3,122 | -20,96 to 14,72 | No | ns | >0,9999 | A-V |
| **Control vs. L.s** | 2,514 | -15,33 to 20,35 | No | ns | >0,9999 | A-W |
| **Control vs. L.la** | 3,200 | -14,64 to 21,04 | No | ns | >0,9999 | A-X |
| **Control vs. L.b** | 1,828 | -16,01 to 19,67 | No | ns | >0,9999 | A-Y |
| **Control vs. L.n** | -0,6660 | -18,51 to 17,17 | No | ns | >0,9999 | A-Z |
| **Control vs. L.li** | 1,914 | -15,93 to 19,75 | No | ns | >0,9999 | A-AA |
| **LPS vs. S.a** | -10,18 | -28,98 to 8,630 | No | ns | 0,9444 | B-C |
| **LPS vs. L.g** | 34,05 | 16,21 to 51,89 | Yes | **** | <0,0001 | B-D |
| **LPS vs. L.s** | 47,33 | 29,49 to 65,17 | Yes | **** | <0,0001 | B-E |
| **LPS vs. L.la** | 42,89 | 25,05 to 60,73 | Yes | **** | <0,0001 | B-F |
| **LPS vs. L.b** | 54,23 | 36,39 to 72,07 | Yes | **** | <0,0001 | B-G |
| **LPS vs. L.n** | 57,27 | 39,43 to 75,11 | Yes | **** | <0,0001 | B-H |
| **LPS vs. L.li** | 56,65 | 38,81 to 74,49 | Yes | **** | <0,0001 | B-I |
| **LPS vs. Control** | 57,44 | 38,63 to 76,24 | Yes | **** | <0,0001 | B-J |
| **LPS vs. LPS** | 4,600 | -14,20 to 23,40 | No | ns | >0,9999 | B-K |
| **LPS vs. S.a** | 53,60 | 34,80 to 72,41 | Yes | **** | <0,0001 | B-L |
| **LPS vs. L.g** | 21,02 | 3,184 to 38,86 | Yes | ** | 0,0052 | B-M |
| **LPS vs. L.s** | 51,11 | 33,27 to 68,95 | Yes | **** | <0,0001 | B-N |
| **LPS vs. L.la** | 40,42 | 22,58 to 58,26 | Yes | **** | <0,0001 | B-O |
| **LPS vs. L.b** | 49,26 | 31,42 to 67,10 | Yes | **** | <0,0001 | B-P |
| **LPS vs. L.n** | 57,47 | 39,63 to 75,31 | Yes | **** | <0,0001 | B-Q |
| **LPS vs. L.li** | 57,47 | 39,63 to 75,31 | Yes | **** | <0,0001 | B-R |
| **LPS vs. Control** | 59,85 | 41,05 to 78,65 | Yes | **** | <0,0001 | B-S |
| **LPS vs. LPS** | 55,64 | 36,83 to 74,44 | Yes | **** | <0,0001 | B-T |
| **LPS vs. S.a** | 1,075 | -17,73 to 19,88 | No | ns | >0,9999 | B-U |
| **LPS vs. L.g** | 53,53 | 35,69 to 71,37 | Yes | **** | <0,0001 | B-V |
| **LPS vs. L.s** | 59,16 | 41,32 to 77,00 | Yes | **** | <0,0001 | B-W |
| **LPS vs. L.la** | 59,85 | 42,01 to 77,69 | Yes | **** | <0,0001 | B-X |
| **LPS vs. L.b** | 58,48 | 40,64 to 76,32 | Yes | **** | <0,0001 | B-Y |
| **LPS vs. L.n** | 55,98 | 38,14 to 73,82 | Yes | **** | <0,0001 | B-Z |
| **LPS vs. L.li** | 58,56 | 40,72 to 76,40 | Yes | **** | <0,0001 | B-AA |
| **S.a vs. L.g** | 44,23 | 26,39 to 62,06 | Yes | **** | <0,0001 | C-D |
| **S.a vs. L.s** | 57,51 | 39,67 to 75,34 | Yes | **** | <0,0001 | C-E |
| **S.a vs. L.la** | 53,07 | 35,23 to 70,90 | Yes | **** | <0,0001 | C-F |
| **S.a vs. L.b** | 64,40 | 46,56 to 82,24 | Yes | **** | <0,0001 | C-G |
| **S.a vs. L.n** | 67,45 | 49,61 to 85,28 | Yes | **** | <0,0001 | C-H |
| **S.a vs. L.li** | 66,83 | 48,99 to 84,66 | Yes | **** | <0,0001 | C-I |
| **S.a vs. Control** | 67,61 | 48,81 to 86,41 | Yes | **** | <0,0001 | C-J |
| **S.a vs. LPS** | 14,78 | -4,030 to 33,58 | No | ns | 0,3666 | C-K |
| **S.a vs. S.a** | 63,78 | 44,97 to 82,58 | Yes | **** | <0,0001 | C-L |
| **S.a vs. L.g** | 31,20 | 13,36 to 49,04 | Yes | **** | <0,0001 | C-M |
| **S.a vs. L.s** | 61,29 | 43,45 to 79,13 | Yes | **** | <0,0001 | C-N |
| **S.a vs. L.la** | 50,60 | 32,76 to 68,44 | Yes | **** | <0,0001 | C-O |
| **S.a vs. L.b** | 59,44 | 41,60 to 77,28 | Yes | **** | <0,0001 | C-P |
| **S.a vs. L.n** | 67,64 | 49,80 to 85,48 | Yes | **** | <0,0001 | C-Q |
| **S.a vs. L.li** | 67,64 | 49,80 to 85,48 | Yes | **** | <0,0001 | C-R |
| **S.a vs. Control** | 70,03 | 51,22 to 88,83 | Yes | **** | <0,0001 | C-S |
| **S.a vs. LPS** | 65,81 | 47,01 to 84,61 | Yes | **** | <0,0001 | C-T |
| **S.a vs. S.a** | 11,25 | -7,555 to 30,05 | No | ns | 0,8607 | C-U |
| **S.a vs. L.g** | 63,70 | 45,86 to 81,54 | Yes | **** | <0,0001 | C-V |
| **S.a vs. L.s** | 69,34 | 51,50 to 87,18 | Yes | **** | <0,0001 | C-W |
| **S.a vs. L.la** | 70,03 | 52,19 to 87,86 | Yes | **** | <0,0001 | C-X |
| **S.a vs. L.b** | 68,65 | 50,81 to 86,49 | Yes | **** | <0,0001 | C-Y |
| **S.a vs. L.n** | 66,16 | 48,32 to 84,00 | Yes | **** | <0,0001 | C-Z |
| **S.a vs. L.li** | 68,74 | 50,90 to 86,58 | Yes | **** | <0,0001 | C-AA |
| **L.g vs. L.s** | 13,28 | -3,539 to 30,10 | No | ns | 0,3568 | D-E |
| **L.g vs. L.la** | 8,840 | -7,979 to 25,66 | No | ns | 0,9589 | D-F |
| **L.g vs. L.b** | 20,18 | 3,357 to 37,00 | Yes | ** | 0,0039 | D-G |
| **L.g vs. L.n** | 23,22 | 6,401 to 40,04 | Yes | *** | 0,0003 | D-H |
| **L.g vs. L.li** | 22,60 | 5,781 to 39,42 | Yes | *** | 0,0005 | D-I |
| **L.g vs. Control** | 23,39 | 5,545 to 41,22 | Yes | *** | 0,0008 | D-J |
| **L.g vs. LPS** | -29,45 | -47,29 to -11,61 | Yes | **** | <0,0001 | D-K |
| **L.g vs. S.a** | 19,55 | 1,713 to 37,39 | Yes | * | 0,0157 | D-L |
| **L.g vs. L.g** | -13,03 | -29,85 to 3,793 | No | ns | 0,3958 | D-M |
| **L.g vs. L.s** | 17,06 | 0,2427 to 33,88 | Yes | * | 0,0424 | D-N |
| **L.g vs. L.la** | 6,372 | -10,45 to 23,19 | No | ns | 0,9995 | D-O |
| **L.g vs. L.b** | 15,21 | -1,607 to 32,03 | No | ns | 0,1364 | D-P |
| **L.g vs. L.n** | 23,42 | 6,597 to 40,24 | Yes | *** | 0,0002 | D-Q |
| **L.g vs. L.li** | 23,42 | 6,597 to 40,24 | Yes | *** | 0,0002 | D-R |
| **L.g vs. Control** | 25,80 | 7,960 to 43,64 | Yes | **** | <0,0001 | D-S |
| **L.g vs. LPS** | 21,59 | 3,745 to 39,42 | Yes | ** | 0,0034 | D-T |
| **L.g vs. S.a** | -32,98 | -50,81 to -15,14 | Yes | **** | <0,0001 | D-U |
| **L.g vs. L.g** | 19,48 | 2,659 to 36,30 | Yes | ** | 0,0069 | D-V |
| **L.g vs. L.s** | 25,11 | 8,295 to 41,93 | Yes | **** | <0,0001 | D-W |
| **L.g vs. L.la** | 25,80 | 8,981 to 42,62 | Yes | **** | <0,0001 | D-X |
| **L.g vs. L.b** | 24,43 | 7,609 to 41,25 | Yes | **** | <0,0001 | D-Y |
| **L.g vs. L.n** | 21,93 | 5,115 to 38,75 | Yes | *** | 0,0009 | D-Z |
| **L.g vs. L.li** | 24,51 | 7,695 to 41,33 | Yes | **** | <0,0001 | D-AA |
| **L.s vs. L.la** | -4,440 | -21,26 to 12,38 | No | ns | >0,9999 | E-F |
| **L.s vs. L.b** | 6,896 | -9,923 to 23,72 | No | ns | 0,9984 | E-G |
| **L.s vs. L.n** | 9,940 | -6,879 to 26,76 | No | ns | 0,8741 | E-H |
| **L.s vs. L.li** | 9,320 | -7,499 to 26,14 | No | ns | 0,9296 | E-I |
| **L.s vs. Control** | 10,11 | -7,735 to 27,94 | No | ns | 0,9134 | E-J |
| **L.s vs. LPS** | -42,73 | -60,57 to -24,89 | Yes | **** | <0,0001 | E-K |
| **L.s vs. S.a** | 6,273 | -11,57 to 24,11 | No | ns | 0,9999 | E-L |
| **L.s vs. L.g** | -26,31 | -43,13 to -9,487 | Yes | **** | <0,0001 | E-M |
| **L.s vs. L.s** | 3,782 | -13,04 to 20,60 | No | ns | >0,9999 | E-N |
| **L.s vs. L.la** | -6,908 | -23,73 to 9,911 | No | ns | 0,9983 | E-O |
| **L.s vs. L.b** | 1,932 | -14,89 to 18,75 | No | ns | >0,9999 | E-P |
| **L.s vs. L.n** | 10,14 | -6,683 to 26,96 | No | ns | 0,8523 | E-Q |
| **L.s vs. L.li** | 10,14 | -6,683 to 26,96 | No | ns | 0,8523 | E-R |
| **L.s vs. Control** | 12,52 | -5,320 to 30,36 | No | ns | 0,6010 | E-S |
| **L.s vs. LPS** | 8,305 | -9,535 to 26,14 | No | ns | 0,9902 | E-T |
| **L.s vs. S.a** | -46,26 | -64,09 to -28,42 | Yes | **** | <0,0001 | E-U |
| **L.s vs. L.g** | 6,198 | -10,62 to 23,02 | No | ns | 0,9997 | E-V |
| **L.s vs. L.s** | 11,83 | -4,985 to 28,65 | No | ns | 0,5959 | E-W |
| **L.s vs. L.la** | 12,52 | -4,299 to 29,34 | No | ns | 0,4784 | E-X |
| **L.s vs. L.b** | 11,15 | -5,671 to 27,97 | No | ns | 0,7105 | E-Y |
| **L.s vs. L.n** | 8,654 | -8,165 to 25,47 | No | ns | 0,9674 | E-Z |
| **L.s vs. L.li** | 11,23 | -5,585 to 28,05 | No | ns | 0,6966 | E-AA |
| **L.la vs. L.b** | 11,34 | -5,483 to 28,16 | No | ns | 0,6800 | F-G |
| **L.la vs. L.n** | 14,38 | -2,439 to 31,20 | No | ns | 0,2139 | F-H |
| **L.la vs. L.li** | 13,76 | -3,059 to 30,58 | No | ns | 0,2889 | F-I |
| **L.la vs. Control** | 14,55 | -3,295 to 32,38 | No | ns | 0,2952 | F-J |
| **L.la vs. LPS** | -38,29 | -56,13 to -20,45 | Yes | **** | <0,0001 | F-K |
| **L.la vs. S.a** | 10,71 | -7,127 to 28,55 | No | ns | 0,8564 | F-L |
| **L.la vs. L.g** | -21,87 | -38,69 to -5,047 | Yes | *** | 0,0009 | F-M |
| **L.la vs. L.s** | 8,222 | -8,597 to 25,04 | No | ns | 0,9820 | F-N |
| **L.la vs. L.la** | -2,468 | -19,29 to 14,35 | No | ns | >0,9999 | F-O |
| **L.la vs. L.b** | 6,372 | -10,45 to 23,19 | No | ns | 0,9995 | F-P |
| **L.la vs. L.n** | 14,58 | -2,243 to 31,40 | No | ns | 0,1933 | F-Q |
| **L.la vs. L.li** | 14,58 | -2,243 to 31,40 | No | ns | 0,1933 | F-R |
| **L.la vs. Control** | 16,96 | -0,8795 to 34,80 | No | ns | 0,0857 | F-S |
| **L.la vs. LPS** | 12,75 | -5,095 to 30,58 | No | ns | 0,5645 | F-T |
| **L.la vs. S.a** | -41,82 | -59,65 to -23,98 | Yes | **** | <0,0001 | F-U |
| **L.la vs. L.g** | 10,64 | -6,181 to 27,46 | No | ns | 0,7874 | F-V |
| **L.la vs. L.s** | 16,27 | -0,5453 to 33,09 | No | ns | 0,0716 | F-W |
| **L.la vs. L.la** | 16,96 | 0,1407 to 33,78 | Yes | * | 0,0455 | F-X |
| **L.la vs. L.b** | 15,59 | -1,231 to 32,41 | No | ns | 0,1095 | F-Y |
| **L.la vs. L.n** | 13,09 | -3,725 to 29,91 | No | ns | 0,3852 | F-Z |
| **L.la vs. L.li** | 15,67 | -1,145 to 32,49 | No | ns | 0,1040 | F-AA |
| **L.b vs. L.n** | 3,044 | -13,78 to 19,86 | No | ns | >0,9999 | G-H |
| **L.b vs. L.li** | 2,424 | -14,40 to 19,24 | No | ns | >0,9999 | G-I |
| **L.b vs. Control** | 3,209 | -14,63 to 21,05 | No | ns | >0,9999 | G-J |
| **L.b vs. LPS** | -49,63 | -67,47 to -31,79 | Yes | **** | <0,0001 | G-K |
| **L.b vs. S.a** | -0,6235 | -18,46 to 17,22 | No | ns | >0,9999 | G-L |
| **L.b vs. L.g** | -33,20 | -50,02 to -16,38 | Yes | **** | <0,0001 | G-M |
| **L.b vs. L.s** | -3,114 | -19,93 to 13,71 | No | ns | >0,9999 | G-N |
| **L.b vs. L.la** | -13,80 | -30,62 to 3,015 | No | ns | 0,2831 | G-O |
| **L.b vs. L.b** | -4,964 | -21,78 to 11,86 | No | ns | >0,9999 | G-P |
| **L.b vs. L.n** | 3,240 | -13,58 to 20,06 | No | ns | >0,9999 | G-Q |
| **L.b vs. L.li** | 3,240 | -13,58 to 20,06 | No | ns | >0,9999 | G-R |
| **L.b vs. Control** | 5,624 | -12,22 to 23,46 | No | ns | >0,9999 | G-S |
| **L.b vs. LPS** | 1,409 | -16,43 to 19,25 | No | ns | >0,9999 | G-T |
| **L.b vs. S.a** | -53,15 | -70,99 to -35,31 | Yes | **** | <0,0001 | G-U |
| **L.b vs. L.g** | -0,6980 | -17,52 to 16,12 | No | ns | >0,9999 | G-V |
| **L.b vs. L.s** | 4,938 | -11,88 to 21,76 | No | ns | >0,9999 | G-W |
| **L.b vs. L.la** | 5,624 | -11,20 to 22,44 | No | ns | >0,9999 | G-X |
| **L.b vs. L.b** | 4,252 | -12,57 to 21,07 | No | ns | >0,9999 | G-Y |
| **L.b vs. L.n** | 1,758 | -15,06 to 18,58 | No | ns | >0,9999 | G-Z |
| **L.b vs. L.li** | 4,338 | -12,48 to 21,16 | No | ns | >0,9999 | G-AA |
| **L.n vs. L.li** | -0,6200 | -17,44 to 16,20 | No | ns | >0,9999 | H-I |
| **L.n vs. Control** | 0,1650 | -17,67 to 18,00 | No | ns | >0,9999 | H-J |
| **L.n vs. LPS** | -52,67 | -70,51 to -34,83 | Yes | **** | <0,0001 | H-K |
| **L.n vs. S.a** | -3,668 | -21,51 to 14,17 | No | ns | >0,9999 | H-L |
| **L.n vs. L.g** | -36,25 | -53,07 to -19,43 | Yes | **** | <0,0001 | H-M |
| **L.n vs. L.s** | -6,158 | -22,98 to 10,66 | No | ns | 0,9997 | H-N |
| **L.n vs. L.la** | -16,85 | -33,67 to -0,02872 | Yes | * | 0,0490 | H-O |
| **L.n vs. L.b** | -8,008 | -24,83 to 8,811 | No | ns | 0,9870 | H-P |
| **L.n vs. L.n** | 0,1960 | -16,62 to 17,02 | No | ns | >0,9999 | H-Q |
| **L.n vs. L.li** | 0,1960 | -16,62 to 17,02 | No | ns | >0,9999 | H-R |
| **L.n vs. Control** | 2,580 | -15,26 to 20,42 | No | ns | >0,9999 | H-S |
| **L.n vs. LPS** | -1,635 | -19,47 to 16,20 | No | ns | >0,9999 | H-T |
| **L.n vs. S.a** | -56,20 | -74,03 to -38,36 | Yes | **** | <0,0001 | H-U |
| **L.n vs. L.g** | -3,742 | -20,56 to 13,08 | No | ns | >0,9999 | H-V |
| **L.n vs. L.s** | 1,894 | -14,93 to 18,71 | No | ns | >0,9999 | H-W |
| **L.n vs. L.la** | 2,580 | -14,24 to 19,40 | No | ns | >0,9999 | H-X |
| **L.n vs. L.b** | 1,208 | -15,61 to 18,03 | No | ns | >0,9999 | H-Y |
| **L.n vs. L.n** | -1,286 | -18,11 to 15,53 | No | ns | >0,9999 | H-Z |
| **L.n vs. L.li** | 1,294 | -15,53 to 18,11 | No | ns | >0,9999 | H-AA |
| **L.li vs. Control** | 0,7850 | -17,05 to 18,62 | No | ns | >0,9999 | I-J |
| **L.li vs. LPS** | -52,05 | -69,89 to -34,21 | Yes | **** | <0,0001 | I-K |
| **L.li vs. S.a** | -3,048 | -20,89 to 14,79 | No | ns | >0,9999 | I-L |
| **L.li vs. L.g** | -35,63 | -52,45 to -18,81 | Yes | **** | <0,0001 | I-M |
| **L.li vs. L.s** | -5,538 | -22,36 to 11,28 | No | ns | >0,9999 | I-N |
| **L.li vs. L.la** | -16,23 | -33,05 to 0,5913 | No | ns | 0,0737 | I-O |
| **L.li vs. L.b** | -7,388 | -24,21 to 9,431 | No | ns | 0,9955 | I-P |
| **L.li vs. L.n** | 0,8160 | -16,00 to 17,64 | No | ns | >0,9999 | I-Q |
| **L.li vs. L.li** | 0,8160 | -16,00 to 17,64 | No | ns | >0,9999 | I-R |
| **L.li vs. Control** | 3,200 | -14,64 to 21,04 | No | ns | >0,9999 | I-S |
| **L.li vs. LPS** | -1,015 | -18,85 to 16,82 | No | ns | >0,9999 | I-T |
| **L.li vs. S.a** | -55,58 | -73,41 to -37,74 | Yes | **** | <0,0001 | I-U |
| **L.li vs. L.g** | -3,122 | -19,94 to 13,70 | No | ns | >0,9999 | I-V |
| **L.li vs. L.s** | 2,514 | -14,31 to 19,33 | No | ns | >0,9999 | I-W |
| **L.li vs. L.la** | 3,200 | -13,62 to 20,02 | No | ns | >0,9999 | I-X |
| **L.li vs. L.b** | 1,828 | -14,99 to 18,65 | No | ns | >0,9999 | I-Y |
| **L.li vs. L.n** | -0,6660 | -17,49 to 16,15 | No | ns | >0,9999 | I-Z |
| **L.li vs. L.li** | 1,914 | -14,91 to 18,73 | No | ns | >0,9999 | I-AA |
| **Control vs. LPS** | -52,84 | -71,64 to -34,03 | Yes | **** | <0,0001 | J-K |
| **Control vs. S.a** | -3,833 | -22,64 to 14,97 | No | ns | >0,9999 | J-L |
| **Control vs. L.g** | -36,41 | -54,25 to -18,57 | Yes | **** | <0,0001 | J-M |
| **Control vs. L.s** | -6,323 | -24,16 to 11,52 | No | ns | 0,9998 | J-N |
| **Control vs. L.la** | -17,01 | -34,85 to 0,8265 | No | ns | 0,0830 | J-O |
| **Control vs. L.b** | -8,173 | -26,01 to 9,667 | No | ns | 0,9921 | J-P |
| **Control vs. L.n** | 0,03100 | -17,81 to 17,87 | No | ns | >0,9999 | J-Q |
| **Control vs. L.li** | 0,03100 | -17,81 to 17,87 | No | ns | >0,9999 | J-R |
| **Control vs. Control** | 2,415 | -16,39 to 21,22 | No | ns | >0,9999 | J-S |
| **Control vs. LPS** | -1,800 | -20,60 to 17,00 | No | ns | >0,9999 | J-T |
| **Control vs. S.a** | -56,36 | -75,16 to -37,56 | Yes | **** | <0,0001 | J-U |
| **Control vs. L.g** | -3,907 | -21,75 to 13,93 | No | ns | >0,9999 | J-V |
| **Control vs. L.s** | 1,729 | -16,11 to 19,57 | No | ns | >0,9999 | J-W |
| **Control vs. L.la** | 2,415 | -15,42 to 20,25 | No | ns | >0,9999 | J-X |
| **Control vs. L.b** | 1,043 | -16,80 to 18,88 | No | ns | >0,9999 | J-Y |
| **Control vs. L.n** | -1,451 | -19,29 to 16,39 | No | ns | >0,9999 | J-Z |
| **Control vs. L.li** | 1,129 | -16,71 to 18,97 | No | ns | >0,9999 | J-AA |
| **LPS vs. S.a** | 49,00 | 30,20 to 67,81 | Yes | **** | <0,0001 | K-L |
| **LPS vs. L.g** | 16,42 | -1,416 to 34,26 | No | ns | 0,1164 | K-M |
| **LPS vs. L.s** | 46,51 | 28,67 to 64,35 | Yes | **** | <0,0001 | K-N |
| **LPS vs. L.la** | 35,82 | 17,98 to 53,66 | Yes | **** | <0,0001 | K-O |
| **LPS vs. L.b** | 44,66 | 26,82 to 62,50 | Yes | **** | <0,0001 | K-P |
| **LPS vs. L.n** | 52,87 | 35,03 to 70,71 | Yes | **** | <0,0001 | K-Q |
| **LPS vs. L.li** | 52,87 | 35,03 to 70,71 | Yes | **** | <0,0001 | K-R |
| **LPS vs. Control** | 55,25 | 36,45 to 74,05 | Yes | **** | <0,0001 | K-S |
| **LPS vs. LPS** | 51,04 | 32,23 to 69,84 | Yes | **** | <0,0001 | K-T |
| **LPS vs. S.a** | -3,525 | -22,33 to 15,28 | No | ns | >0,9999 | K-U |
| **LPS vs. L.g** | 48,93 | 31,09 to 66,77 | Yes | **** | <0,0001 | K-V |
| **LPS vs. L.s** | 54,56 | 36,72 to 72,40 | Yes | **** | <0,0001 | K-W |
| **LPS vs. L.la** | 55,25 | 37,41 to 73,09 | Yes | **** | <0,0001 | K-X |
| **LPS vs. L.b** | 53,88 | 36,04 to 71,72 | Yes | **** | <0,0001 | K-Y |
| **LPS vs. L.n** | 51,38 | 33,54 to 69,22 | Yes | **** | <0,0001 | K-Z |
| **LPS vs. L.li** | 53,96 | 36,12 to 71,80 | Yes | **** | <0,0001 | K-AA |
| **S.a vs. L.g** | -32,58 | -50,42 to -14,74 | Yes | **** | <0,0001 | L-M |
| **S.a vs. L.s** | -2,491 | -20,33 to 15,35 | No | ns | >0,9999 | L-N |
| **S.a vs. L.la** | -13,18 | -31,02 to 4,659 | No | ns | 0,4942 | L-O |
| **S.a vs. L.b** | -4,341 | -22,18 to 13,50 | No | ns | >0,9999 | L-P |
| **S.a vs. L.n** | 3,864 | -13,98 to 21,70 | No | ns | >0,9999 | L-Q |
| **S.a vs. L.li** | 3,864 | -13,98 to 21,70 | No | ns | >0,9999 | L-R |
| **S.a vs. Control** | 6,248 | -12,56 to 25,05 | No | ns | >0,9999 | L-S |
| **S.a vs. LPS** | 2,033 | -16,77 to 20,84 | No | ns | >0,9999 | L-T |
| **S.a vs. S.a** | -52,53 | -71,33 to -33,72 | Yes | **** | <0,0001 | L-U |
| **S.a vs. L.g** | -0,07450 | -17,91 to 17,77 | No | ns | >0,9999 | L-V |
| **S.a vs. L.s** | 5,562 | -12,28 to 23,40 | No | ns | >0,9999 | L-W |
| **S.a vs. L.la** | 6,248 | -11,59 to 24,09 | No | ns | 0,9999 | L-X |
| **S.a vs. L.b** | 4,876 | -12,96 to 22,72 | No | ns | >0,9999 | L-Y |
| **S.a vs. L.n** | 2,382 | -15,46 to 20,22 | No | ns | >0,9999 | L-Z |
| **S.a vs. L.li** | 4,962 | -12,88 to 22,80 | No | ns | >0,9999 | L-AA |
| **L.g vs. L.s** | 30,09 | 13,27 to 46,91 | Yes | **** | <0,0001 | M-N |
| **L.g vs. L.la** | 19,40 | 2,579 to 36,22 | Yes | ** | 0,0074 | M-O |
| **L.g vs. L.b** | 28,24 | 11,42 to 45,06 | Yes | **** | <0,0001 | M-P |
| **L.g vs. L.n** | 36,44 | 19,62 to 53,26 | Yes | **** | <0,0001 | M-Q |
| **L.g vs. L.li** | 36,44 | 19,62 to 53,26 | Yes | **** | <0,0001 | M-R |
| **L.g vs. Control** | 38,83 | 20,99 to 56,67 | Yes | **** | <0,0001 | M-S |
| **L.g vs. LPS** | 34,61 | 16,77 to 52,45 | Yes | **** | <0,0001 | M-T |
| **L.g vs. S.a** | -19,95 | -37,79 to -2,109 | Yes | * | 0,0118 | M-U |
| **L.g vs. L.g** | 32,50 | 15,68 to 49,32 | Yes | **** | <0,0001 | M-V |
| **L.g vs. L.s** | 38,14 | 21,32 to 54,96 | Yes | **** | <0,0001 | M-W |
| **L.g vs. L.la** | 38,83 | 22,01 to 55,65 | Yes | **** | <0,0001 | M-X |
| **L.g vs. L.b** | 37,45 | 20,63 to 54,27 | Yes | **** | <0,0001 | M-Y |
| **L.g vs. L.n** | 34,96 | 18,14 to 51,78 | Yes | **** | <0,0001 | M-Z |
| **L.g vs. L.li** | 37,54 | 20,72 to 54,36 | Yes | **** | <0,0001 | M-AA |
| **L.s vs. L.la** | -10,69 | -27,51 to 6,129 | No | ns | 0,7800 | N-O |
| **L.s vs. L.b** | -1,850 | -18,67 to 14,97 | No | ns | >0,9999 | N-P |
| **L.s vs. L.n** | 6,354 | -10,47 to 23,17 | No | ns | 0,9996 | N-Q |
| **L.s vs. L.li** | 6,354 | -10,47 to 23,17 | No | ns | 0,9996 | N-R |
| **L.s vs. Control** | 8,738 | -9,102 to 26,58 | No | ns | 0,9816 | N-S |
| **L.s vs. LPS** | 4,523 | -13,32 to 22,36 | No | ns | >0,9999 | N-T |
| **L.s vs. S.a** | -50,04 | -67,88 to -32,20 | Yes | **** | <0,0001 | N-U |
| **L.s vs. L.g** | 2,416 | -14,40 to 19,24 | No | ns | >0,9999 | N-V |
| **L.s vs. L.s** | 8,052 | -8,767 to 24,87 | No | ns | 0,9861 | N-W |
| **L.s vs. L.la** | 8,738 | -8,081 to 25,56 | No | ns | 0,9638 | N-X |
| **L.s vs. L.b** | 7,366 | -9,453 to 24,19 | No | ns | 0,9957 | N-Y |
| **L.s vs. L.n** | 4,872 | -11,95 to 21,69 | No | ns | >0,9999 | N-Z |
| **L.s vs. L.li** | 7,452 | -9,367 to 24,27 | No | ns | 0,9950 | N-AA |
| **L.la vs. L.b** | 8,840 | -7,979 to 25,66 | No | ns | 0,9589 | O-P |
| **L.la vs. L.n** | 17,04 | 0,2247 to 33,86 | Yes | * | 0,0429 | O-Q |
| **L.la vs. L.li** | 17,04 | 0,2247 to 33,86 | Yes | * | 0,0429 | O-R |
| **L.la vs. Control** | 19,43 | 1,588 to 37,27 | Yes | * | 0,0171 | O-S |
| **L.la vs. LPS** | 15,21 | -2,627 to 33,05 | No | ns | 0,2179 | O-T |
| **L.la vs. S.a** | -39,35 | -57,19 to -21,51 | Yes | **** | <0,0001 | O-U |
| **L.la vs. L.g** | 13,11 | -3,713 to 29,93 | No | ns | 0,3833 | O-V |
| **L.la vs. L.s** | 18,74 | 1,923 to 35,56 | Yes | * | 0,0124 | O-W |
| **L.la vs. L.la** | 19,43 | 2,609 to 36,25 | Yes | ** | 0,0072 | O-X |
| **L.la vs. L.b** | 18,06 | 1,237 to 34,88 | Yes | * | 0,0208 | O-Y |
| **L.la vs. L.n** | 15,56 | -1,257 to 32,38 | No | ns | 0,1112 | O-Z |
| **L.la vs. L.li** | 18,14 | 1,323 to 34,96 | Yes | * | 0,0195 | O-AA |
| **L.b vs. L.n** | 8,204 | -8,615 to 25,02 | No | ns | 0,9825 | P-Q |
| **L.b vs. L.li** | 8,204 | -8,615 to 25,02 | No | ns | 0,9825 | P-R |
| **L.b vs. Control** | 10,59 | -7,252 to 28,43 | No | ns | 0,8695 | P-S |
| **L.b vs. LPS** | 6,373 | -11,47 to 24,21 | No | ns | 0,9998 | P-T |
| **L.b vs. S.a** | -48,19 | -66,03 to -30,35 | Yes | **** | <0,0001 | P-U |
| **L.b vs. L.g** | 4,266 | -12,55 to 21,09 | No | ns | >0,9999 | P-V |
| **L.b vs. L.s** | 9,902 | -6,917 to 26,72 | No | ns | 0,8781 | P-W |
| **L.b vs. L.la** | 10,59 | -6,231 to 27,41 | No | ns | 0,7944 | P-X |
| **L.b vs. L.b** | 9,216 | -7,603 to 26,04 | No | ns | 0,9369 | P-Y |
| **L.b vs. L.n** | 6,722 | -10,10 to 23,54 | No | ns | 0,9989 | P-Z |
| **L.b vs. L.li** | 9,302 | -7,517 to 26,12 | No | ns | 0,9309 | P-AA |
| **L.n vs. L.li** | 0,000 | -16,82 to 16,82 | No | ns | >0,9999 | Q-R |
| **L.n vs. Control** | 2,384 | -15,46 to 20,22 | No | ns | >0,9999 | Q-S |
| **L.n vs. LPS** | -1,831 | -19,67 to 16,01 | No | ns | >0,9999 | Q-T |
| **L.n vs. S.a** | -56,39 | -74,23 to -38,55 | Yes | **** | <0,0001 | Q-U |
| **L.n vs. L.g** | -3,938 | -20,76 to 12,88 | No | ns | >0,9999 | Q-V |
| **L.n vs. L.s** | 1,698 | -15,12 to 18,52 | No | ns | >0,9999 | Q-W |
| **L.n vs. L.la** | 2,384 | -14,44 to 19,20 | No | ns | >0,9999 | Q-X |
| **L.n vs. L.b** | 1,012 | -15,81 to 17,83 | No | ns | >0,9999 | Q-Y |
| **L.n vs. L.n** | -1,482 | -18,30 to 15,34 | No | ns | >0,9999 | Q-Z |
| **L.n vs. L.li** | 1,098 | -15,72 to 17,92 | No | ns | >0,9999 | Q-AA |
| **L.li vs. Control** | 2,384 | -15,46 to 20,22 | No | ns | >0,9999 | R-S |
| **L.li vs. LPS** | -1,831 | -19,67 to 16,01 | No | ns | >0,9999 | R-T |
| **L.li vs. S.a** | -56,39 | -74,23 to -38,55 | Yes | **** | <0,0001 | R-U |
| **L.li vs. L.g** | -3,938 | -20,76 to 12,88 | No | ns | >0,9999 | R-V |
| **L.li vs. L.s** | 1,698 | -15,12 to 18,52 | No | ns | >0,9999 | R-W |
| **L.li vs. L.la** | 2,384 | -14,44 to 19,20 | No | ns | >0,9999 | R-X |
| **L.li vs. L.b** | 1,012 | -15,81 to 17,83 | No | ns | >0,9999 | R-Y |
| **L.li vs. L.n** | -1,482 | -18,30 to 15,34 | No | ns | >0,9999 | R-Z |
| **L.li vs. L.li** | 1,098 | -15,72 to 17,92 | No | ns | >0,9999 | R-AA |
| **Control vs. LPS** | -4,215 | -23,02 to 14,59 | No | ns | >0,9999 | S-T |
| **Control vs. S.a** | -58,78 | -77,58 to -39,97 | Yes | **** | <0,0001 | S-U |
| **Control vs. L.g** | -6,322 | -24,16 to 11,52 | No | ns | 0,9998 | S-V |
| **Control vs. L.s** | -0,6860 | -18,53 to 17,15 | No | ns | >0,9999 | S-W |
| **Control vs. L.la** | 0,000 | -17,84 to 17,84 | No | ns | >0,9999 | S-X |
| **Control vs. L.b** | -1,372 | -19,21 to 16,47 | No | ns | >0,9999 | S-Y |
| **Control vs. L.n** | -3,866 | -21,71 to 13,97 | No | ns | >0,9999 | S-Z |
| **Control vs. L.li** | -1,286 | -19,13 to 16,55 | No | ns | >0,9999 | S-AA |
| **LPS vs. S.a** | -54,56 | -73,36 to -35,76 | Yes | **** | <0,0001 | T-U |
| **LPS vs. L.g** | -2,107 | -19,95 to 15,73 | No | ns | >0,9999 | T-V |
| **LPS vs. L.s** | 3,529 | -14,31 to 21,37 | No | ns | >0,9999 | T-W |
| **LPS vs. L.la** | 4,215 | -13,62 to 22,05 | No | ns | >0,9999 | T-X |
| **LPS vs. L.b** | 2,843 | -15,00 to 20,68 | No | ns | >0,9999 | T-Y |
| **LPS vs. L.n** | 0,3490 | -17,49 to 18,19 | No | ns | >0,9999 | T-Z |
| **LPS vs. L.li** | 2,929 | -14,91 to 20,77 | No | ns | >0,9999 | T-AA |
| **S.a vs. L.g** | 52,45 | 34,61 to 70,29 | Yes | **** | <0,0001 | U-V |
| **S.a vs. L.s** | 58,09 | 40,25 to 75,93 | Yes | **** | <0,0001 | U-W |
| **S.a vs. L.la** | 58,78 | 40,94 to 76,61 | Yes | **** | <0,0001 | U-X |
| **S.a vs. L.b** | 57,40 | 39,56 to 75,24 | Yes | **** | <0,0001 | U-Y |
| **S.a vs. L.n** | 54,91 | 37,07 to 72,75 | Yes | **** | <0,0001 | U-Z |
| **S.a vs. L.li** | 57,49 | 39,65 to 75,33 | Yes | **** | <0,0001 | U-AA |
| **L.g vs. L.s** | 5,636 | -11,18 to 22,46 | No | ns | >0,9999 | V-W |
| **L.g vs. L.la** | 6,322 | -10,50 to 23,14 | No | ns | 0,9996 | V-X |
| **L.g vs. L.b** | 4,950 | -11,87 to 21,77 | No | ns | >0,9999 | V-Y |
| **L.g vs. L.n** | 2,456 | -14,36 to 19,28 | No | ns | >0,9999 | V-Z |
| **L.g vs. L.li** | 5,036 | -11,78 to 21,86 | No | ns | >0,9999 | V-AA |
| **L.s vs. L.la** | 0,6860 | -16,13 to 17,51 | No | ns | >0,9999 | W-X |
| **L.s vs. L.b** | -0,6860 | -17,51 to 16,13 | No | ns | >0,9999 | W-Y |
| **L.s vs. L.n** | -3,180 | -20,00 to 13,64 | No | ns | >0,9999 | W-Z |
| **L.s vs. L.li** | -0,6000 | -17,42 to 16,22 | No | ns | >0,9999 | W-AA |
| **L.la vs. L.b** | -1,372 | -18,19 to 15,45 | No | ns | >0,9999 | X-Y |
| **L.la vs. L.n** | -3,866 | -20,69 to 12,95 | No | ns | >0,9999 | X-Z |
| **L.la vs. L.li** | -1,286 | -18,11 to 15,53 | No | ns | >0,9999 | X-AA |
| **L.b vs. L.n** | -2,494 | -19,31 to 14,33 | No | ns | >0,9999 | Y-Z |
| **L.b vs. L.li** | 0,08600 | -16,73 to 16,91 | No | ns | >0,9999 | Y-AA |
| **L.n vs. L.li** | 2,580 | -14,24 to 19,40 | No | ns | >0,9999 | Z-AA |

**Raw data and statistical analysis of Figure 16:**

**MCP-1 (µg/mL)**

**LPG (WT, TLR2KO and TLR4KO)**

| **Control** | **LPS** | **S.a** | **L.g** | **L.s** | **L.la** | **L.b** | **L.n** | **L.li** |
| --- | --- | --- | --- | --- | --- | --- | --- | --- |
| 6,8 | 1447 | 640 | 788,5 | 826,9 | 603,1 | 958,5 | 804,5 | 831,6 |
| 5,5 | 2161 | 536,9 | 895,8 | 839,9 | 659,4 | 811,5 | 644,3 | 1065 |
| 4,8 | 1489 | 532,6 | 751,6 | 938,2 | 643,2 | 638,6 | 929,9 | 981,4 |
| 5,8 | 1544 | 517,4 | 861,5 | 885,7 | 675 | 731 | 673,3 | 746,8 |
|  |  |  | 736,6 | 628,3 | 553,1 | 1020 | 640,8 | 688,3 |
| **Control** | **LPS** | **S.a** | **L.g** | **L.s** | **L.la** | **L.b** | **L.n** | **L.li** |
| 1,9 | 1837 | 500,46 | 1103 | 558,55 | 3137 | 2487 | 707,81 | 958,38 |
| 1,3 | 1837 | 336,55 | 1001 | 2180 | 2941 | 1342 | 1427 | 1088 |
| 1,7 | 2548 | 368,43 | 1028 | 1044 | 1699 | 1229 | 1188 | 2028 |
| 1,7 | 2053 | 485,65 | 612,14 | 683,34 | 1932 | 1412 | 1787 | 622,57 |
|  |  |  | 580,28 | 334,62 | 1661 |  | 787,62 | 1160 |
| **Control** | **LPS** | **S.a** | **L.g** | **L.s** | **L.la** | **L.b** | **L.n** | **L.li** |
| 8,1 | 509 | 2168 | 1274 | 1313 | 1501 | 1425 | 1452 | 962,11 |
| 8 | 668 | 1610 | 1230 | 941,83 | 927,54 | 986,83 | 1209 | 835,51 |
| 6,3 | 424 | 1714,35 | 1095 | 756,56 | 790,11 | 1045 | 1355 | 859,97 |
| 6,9 | 524 | 1827,95 | 963,67 | 746,3 | 943,91 | 1357 | 1271 | 628,56 |
|  |  |  | 951,19 | 1088 | 1033 | 1144 | 1601 | 812,05 |

**Statistical analysis**

| **Tukey's multiple comparisons test** | **Mean Diff,** | **95,00% CI of diff,** | **Significant?** | **Summary** | **Adjusted P Value** |  |
| --- | --- | --- | --- | --- | --- | --- |
| **Control vs. LPS** | -1655 | -2492 to -817,4 | Yes | **** | <0,0001 | A-B |
| **Control vs. S.a** | -551,0 | -1388 to 286,1 | No | ns | 0,7222 | A-C |
| **Control vs. L.g** | -801,1 | -1595 to -6,892 | Yes | * | 0,0453 | A-D |
| **Control vs. L.s** | -818,1 | -1612 to -23,89 | Yes | * | 0,0353 | A-E |
| **Control vs. L.la** | -621,0 | -1415 to 173,1 | No | ns | 0,3758 | A-F |
| **Control vs. L.b** | -826,2 | -1620 to -32,01 | Yes | * | 0,0313 | A-G |
| **Control vs. L.n** | -732,8 | -1527 to 61,35 | No | ns | 0,1139 | A-H |
| **Control vs. L.li** | -856,9 | -1651 to -62,71 | Yes | * | 0,0195 | A-I |
| **Control vs. Control** | 4,075 | -833,1 to 841,2 | No | ns | >0,9999 | A-J |
| **Control vs. LPS** | -2063 | -2900 to -1226 | Yes | **** | <0,0001 | A-K |
| **Control vs. S.a** | -417,0 | -1254 to 420,1 | No | ns | 0,9773 | A-L |
| **Control vs. L.g** | -859,2 | -1653 to -64,98 | Yes | * | 0,0188 | A-M |
| **Control vs. L.s** | -954,4 | -1749 to -160,2 | Yes | ** | 0,0038 | A-N |
| **Control vs. L.la** | -2268 | -3062 to -1474 | Yes | **** | <0,0001 | A-O |
| **Control vs. L.b** | -1612 | -2449 to -774,6 | Yes | **** | <0,0001 | A-P |
| **Control vs. L.n** | -1174 | -1968 to -379,6 | Yes | **** | <0,0001 | A-Q |
| **Control vs. L.li** | -1166 | -1960 to -371,5 | Yes | **** | <0,0001 | A-R |
| **Control vs. Control** | -1,600 | -838,7 to 835,5 | No | ns | >0,9999 | A-S |
| **Control vs. LPS** | -525,5 | -1363 to 311,6 | No | ns | 0,7980 | A-T |
| **Control vs. S.a** | -1824 | -2661 to -987,2 | Yes | **** | <0,0001 | A-U |
| **Control vs. L.g** | -1097 | -1891 to -302,9 | Yes | *** | 0,0003 | A-V |
| **Control vs. L.s** | -963,4 | -1758 to -169,2 | Yes | ** | 0,0032 | A-W |
| **Control vs. L.la** | -1033 | -1828 to -239,2 | Yes | *** | 0,0009 | A-X |
| **Control vs. L.b** | -1186 | -1980 to -391,7 | Yes | **** | <0,0001 | A-Y |
| **Control vs. L.n** | -1372 | -2166 to -577,7 | Yes | **** | <0,0001 | A-Z |
| **Control vs. L.li** | -813,9 | -1608 to -19,73 | Yes | * | 0,0376 | A-AA |
| **LPS vs. S.a** | 1104 | 266,4 to 1941 | Yes | *** | 0,0007 | B-C |
| **LPS vs. L.g** | 853,5 | 59,27 to 1648 | Yes | * | 0,0206 | B-D |
| **LPS vs. L.s** | 836,5 | 42,27 to 1631 | Yes | * | 0,0268 | B-E |
| **LPS vs. L.la** | 1033 | 239,3 to 1828 | Yes | *** | 0,0009 | B-F |
| **LPS vs. L.b** | 828,3 | 34,15 to 1623 | Yes | * | 0,0303 | B-G |
| **LPS vs. L.n** | 921,7 | 127,5 to 1716 | Yes | ** | 0,0067 | B-H |
| **LPS vs. L.li** | 797,6 | 3,447 to 1592 | Yes | * | 0,0476 | B-I |
| **LPS vs. Control** | 1659 | 821,5 to 2496 | Yes | **** | <0,0001 | B-J |
| **LPS vs. LPS** | -408,5 | -1246 to 428,6 | No | ns | 0,9823 | B-K |
| **LPS vs. S.a** | 1237 | 400,3 to 2075 | Yes | **** | <0,0001 | B-L |
| **LPS vs. L.g** | 795,4 | 1,183 to 1590 | Yes | * | 0,0492 | B-M |
| **LPS vs. L.s** | 700,1 | -94,03 to 1494 | No | ns | 0,1688 | B-N |
| **LPS vs. L.la** | -613,8 | -1408 to 180,4 | No | ns | 0,3998 | B-O |
| **LPS vs. L.b** | 42,75 | -794,4 to 879,9 | No | ns | >0,9999 | B-P |
| **LPS vs. L.n** | 480,8 | -313,4 to 1275 | No | ns | 0,8464 | B-Q |
| **LPS vs. L.li** | 488,9 | -305,3 to 1283 | No | ns | 0,8252 | B-R |
| **LPS vs. Control** | 1653 | 815,8 to 2490 | Yes | **** | <0,0001 | B-S |
| **LPS vs. LPS** | 1129 | 291,9 to 1966 | Yes | *** | 0,0004 | B-T |
| **LPS vs. S.a** | -169,8 | -1007 to 667,3 | No | ns | >0,9999 | B-U |
| **LPS vs. L.g** | 557,5 | -236,7 to 1352 | No | ns | 0,6001 | B-V |
| **LPS vs. L.s** | 691,1 | -103,1 to 1485 | No | ns | 0,1870 | B-W |
| **LPS vs. L.la** | 621,1 | -173,0 to 1415 | No | ns | 0,3754 | B-X |
| **LPS vs. L.b** | 468,7 | -325,5 to 1263 | No | ns | 0,8752 | B-Y |
| **LPS vs. L.n** | 282,7 | -511,5 to 1077 | No | ns | 0,9998 | B-Z |
| **LPS vs. L.li** | 840,6 | 46,43 to 1635 | Yes | * | 0,0251 | B-AA |
| **S.a vs. L.g** | -250,1 | -1044 to 544,1 | No | ns | >0,9999 | C-D |
| **S.a vs. L.s** | -267,1 | -1061 to 527,1 | No | ns | >0,9999 | C-E |
| **S.a vs. L.la** | -70,04 | -864,2 to 724,1 | No | ns | >0,9999 | C-F |
| **S.a vs. L.b** | -275,2 | -1069 to 519,0 | No | ns | 0,9999 | C-G |
| **S.a vs. L.n** | -181,8 | -976,0 to 612,3 | No | ns | >0,9999 | C-H |
| **S.a vs. L.li** | -305,9 | -1100 to 488,3 | No | ns | 0,9994 | C-I |
| **S.a vs. Control** | 555,1 | -282,1 to 1392 | No | ns | 0,7092 | C-J |
| **S.a vs. LPS** | -1512 | -2349 to -674,9 | Yes | **** | <0,0001 | C-K |
| **S.a vs. S.a** | 134,0 | -703,2 to 971,1 | No | ns | >0,9999 | C-L |
| **S.a vs. L.g** | -308,2 | -1102 to 486,0 | No | ns | 0,9993 | C-M |
| **S.a vs. L.s** | -403,4 | -1198 to 390,8 | No | ns | 0,9717 | C-N |
| **S.a vs. L.la** | -1717 | -2511 to -923,1 | Yes | **** | <0,0001 | C-O |
| **S.a vs. L.b** | -1061 | -1898 to -223,6 | Yes | ** | 0,0015 | C-P |
| **S.a vs. L.n** | -622,8 | -1417 to 171,4 | No | ns | 0,3702 | C-Q |
| **S.a vs. L.li** | -614,7 | -1409 to 179,5 | No | ns | 0,3967 | C-R |
| **S.a vs. Control** | 549,4 | -287,7 to 1387 | No | ns | 0,7272 | C-S |
| **S.a vs. LPS** | 25,48 | -811,7 to 862,6 | No | ns | >0,9999 | C-T |
| **S.a vs. S.a** | -1273 | -2110 to -436,2 | Yes | **** | <0,0001 | C-U |
| **S.a vs. L.g** | -546,0 | -1340 to 248,1 | No | ns | 0,6413 | C-V |
| **S.a vs. L.s** | -412,4 | -1207 to 381,8 | No | ns | 0,9637 | C-W |
| **S.a vs. L.la** | -482,4 | -1277 to 311,8 | No | ns | 0,8423 | C-X |
| **S.a vs. L.b** | -634,8 | -1429 to 159,3 | No | ns | 0,3323 | C-Y |
| **S.a vs. L.n** | -820,9 | -1615 to -26,69 | Yes | * | 0,0339 | C-Z |
| **S.a vs. L.li** | -262,9 | -1057 to 531,3 | No | ns | >0,9999 | C-AA |
| **L.g vs. L.s** | -17,00 | -765,8 to 731,8 | No | ns | >0,9999 | D-E |
| **L.g vs. L.la** | 180,0 | -568,7 to 928,8 | No | ns | >0,9999 | D-F |
| **L.g vs. L.b** | -25,12 | -773,9 to 723,6 | No | ns | >0,9999 | D-G |
| **L.g vs. L.n** | 68,24 | -680,5 to 817,0 | No | ns | >0,9999 | D-H |
| **L.g vs. L.li** | -55,82 | -804,6 to 692,9 | No | ns | >0,9999 | D-I |
| **L.g vs. Control** | 805,2 | 10,97 to 1599 | Yes | * | 0,0427 | D-J |
| **L.g vs. LPS** | -1262 | -2056 to -467,8 | Yes | **** | <0,0001 | D-K |
| **L.g vs. S.a** | 384,0 | -410,2 to 1178 | No | ns | 0,9841 | D-L |
| **L.g vs. L.g** | -58,08 | -806,8 to 690,7 | No | ns | >0,9999 | D-M |
| **L.g vs. L.s** | -153,3 | -902,1 to 595,5 | No | ns | >0,9999 | D-N |
| **L.g vs. L.la** | -1467 | -2216 to -718,4 | Yes | **** | <0,0001 | D-O |
| **L.g vs. L.b** | -810,7 | -1605 to -16,52 | Yes | * | 0,0394 | D-P |
| **L.g vs. L.n** | -372,7 | -1121 to 376,1 | No | ns | 0,9776 | D-Q |
| **L.g vs. L.li** | -364,6 | -1113 to 384,2 | No | ns | 0,9827 | D-R |
| **L.g vs. Control** | 799,5 | 5,292 to 1594 | Yes | * | 0,0463 | D-S |
| **L.g vs. LPS** | 275,6 | -518,6 to 1070 | No | ns | 0,9999 | D-T |
| **L.g vs. S.a** | -1023 | -1817 to -229,1 | Yes | ** | 0,0011 | D-U |
| **L.g vs. L.g** | -296,0 | -1045 to 452,8 | No | ns | 0,9991 | D-V |
| **L.g vs. L.s** | -162,3 | -911,1 to 586,4 | No | ns | >0,9999 | D-W |
| **L.g vs. L.la** | -232,3 | -981,1 to 516,5 | No | ns | >0,9999 | D-X |
| **L.g vs. L.b** | -384,8 | -1134 to 364,0 | No | ns | 0,9677 | D-Y |
| **L.g vs. L.n** | -570,8 | -1320 to 178,0 | No | ns | 0,4280 | D-Z |
| **L.g vs. L.li** | -12,84 | -761,6 to 735,9 | No | ns | >0,9999 | D-AA |
| **L.s vs. L.la** | 197,0 | -551,7 to 945,8 | No | ns | >0,9999 | E-F |
| **L.s vs. L.b** | -8,120 | -756,9 to 740,6 | No | ns | >0,9999 | E-G |
| **L.s vs. L.n** | 85,24 | -663,5 to 834,0 | No | ns | >0,9999 | E-H |
| **L.s vs. L.li** | -38,82 | -787,6 to 709,9 | No | ns | >0,9999 | E-I |
| **L.s vs. Control** | 822,2 | 27,97 to 1616 | Yes | * | 0,0332 | E-J |
| **L.s vs. LPS** | -1245 | -2039 to -450,8 | Yes | **** | <0,0001 | E-K |
| **L.s vs. S.a** | 401,0 | -393,2 to 1195 | No | ns | 0,9735 | E-L |
| **L.s vs. L.g** | -41,08 | -789,8 to 707,7 | No | ns | >0,9999 | E-M |
| **L.s vs. L.s** | -136,3 | -885,1 to 612,5 | No | ns | >0,9999 | E-N |
| **L.s vs. L.la** | -1450 | -2199 to -701,4 | Yes | **** | <0,0001 | E-O |
| **L.s vs. L.b** | -793,7 | -1588 to 0,4828 | No | ns | 0,0503 | E-P |
| **L.s vs. L.n** | -355,7 | -1104 to 393,1 | No | ns | 0,9873 | E-Q |
| **L.s vs. L.li** | -347,6 | -1096 to 401,2 | No | ns | 0,9905 | E-R |
| **L.s vs. Control** | 816,5 | 22,29 to 1611 | Yes | * | 0,0362 | E-S |
| **L.s vs. LPS** | 292,6 | -501,6 to 1087 | No | ns | 0,9997 | E-T |
| **L.s vs. S.a** | -1006 | -1800 to -212,1 | Yes | ** | 0,0015 | E-U |
| **L.s vs. L.g** | -279,0 | -1028 to 469,8 | No | ns | 0,9996 | E-V |
| **L.s vs. L.s** | -145,3 | -894,1 to 603,4 | No | ns | >0,9999 | E-W |
| **L.s vs. L.la** | -215,3 | -964,1 to 533,5 | No | ns | >0,9999 | E-X |
| **L.s vs. L.b** | -367,8 | -1117 to 381,0 | No | ns | 0,9808 | E-Y |
| **L.s vs. L.n** | -553,8 | -1303 to 195,0 | No | ns | 0,4914 | E-Z |
| **L.s vs. L.li** | 4,160 | -744,6 to 752,9 | No | ns | >0,9999 | E-AA |
| **L.la vs. L.b** | -205,2 | -953,9 to 543,6 | No | ns | >0,9999 | F-G |
| **L.la vs. L.n** | -111,8 | -860,6 to 637,0 | No | ns | >0,9999 | F-H |
| **L.la vs. L.li** | -235,9 | -984,6 to 512,9 | No | ns | >0,9999 | F-I |
| **L.la vs. Control** | 625,1 | -169,1 to 1419 | No | ns | 0,3627 | F-J |
| **L.la vs. LPS** | -1442 | -2236 to -647,8 | Yes | **** | <0,0001 | F-K |
| **L.la vs. S.a** | 204,0 | -590,2 to 998,2 | No | ns | >0,9999 | F-L |
| **L.la vs. L.g** | -238,1 | -986,9 to 510,6 | No | ns | >0,9999 | F-M |
| **L.la vs. L.s** | -333,3 | -1082 to 415,4 | No | ns | 0,9946 | F-N |
| **L.la vs. L.la** | -1647 | -2396 to -898,5 | Yes | **** | <0,0001 | F-O |
| **L.la vs. L.b** | -990,7 | -1785 to -196,6 | Yes | ** | 0,0020 | F-P |
| **L.la vs. L.n** | -552,7 | -1301 to 196,0 | No | ns | 0,4955 | F-Q |
| **L.la vs. L.li** | -544,6 | -1293 to 204,1 | No | ns | 0,5265 | F-R |
| **L.la vs. Control** | 619,4 | -174,7 to 1414 | No | ns | 0,3810 | F-S |
| **L.la vs. LPS** | 95,51 | -698,7 to 889,7 | No | ns | >0,9999 | F-T |
| **L.la vs. S.a** | -1203 | -1997 to -409,1 | Yes | **** | <0,0001 | F-U |
| **L.la vs. L.g** | -476,0 | -1225 to 272,8 | No | ns | 0,7791 | F-V |
| **L.la vs. L.s** | -342,4 | -1091 to 406,4 | No | ns | 0,9922 | F-W |
| **L.la vs. L.la** | -412,4 | -1161 to 336,4 | No | ns | 0,9334 | F-X |
| **L.la vs. L.b** | -564,8 | -1314 to 184,0 | No | ns | 0,4501 | F-Y |
| **L.la vs. L.n** | -750,8 | -1500 to -2,077 | Yes | * | 0,0485 | F-Z |
| **L.la vs. L.li** | -192,9 | -941,6 to 555,9 | No | ns | >0,9999 | F-AA |
| **L.b vs. L.n** | 93,36 | -655,4 to 842,1 | No | ns | >0,9999 | G-H |
| **L.b vs. L.li** | -30,70 | -779,5 to 718,1 | No | ns | >0,9999 | G-I |
| **L.b vs. Control** | 830,3 | 36,09 to 1624 | Yes | * | 0,0294 | G-J |
| **L.b vs. LPS** | -1237 | -2031 to -442,6 | Yes | **** | <0,0001 | G-K |
| **L.b vs. S.a** | 409,1 | -385,0 to 1203 | No | ns | 0,9668 | G-L |
| **L.b vs. L.g** | -32,96 | -781,7 to 715,8 | No | ns | >0,9999 | G-M |
| **L.b vs. L.s** | -128,2 | -876,9 to 620,6 | No | ns | >0,9999 | G-N |
| **L.b vs. L.la** | -1442 | -2191 to -693,3 | Yes | **** | <0,0001 | G-O |
| **L.b vs. L.b** | -785,6 | -1580 to 8,603 | No | ns | 0,0565 | G-P |
| **L.b vs. L.n** | -347,6 | -1096 to 401,2 | No | ns | 0,9905 | G-Q |
| **L.b vs. L.li** | -339,5 | -1088 to 409,3 | No | ns | 0,9931 | G-R |
| **L.b vs. Control** | 824,6 | 30,41 to 1619 | Yes | * | 0,0320 | G-S |
| **L.b vs. LPS** | 300,7 | -493,5 to 1095 | No | ns | 0,9995 | G-T |
| **L.b vs. S.a** | -998,2 | -1792 to -204,0 | Yes | ** | 0,0017 | G-U |
| **L.b vs. L.g** | -270,9 | -1020 to 477,9 | No | ns | 0,9998 | G-V |
| **L.b vs. L.s** | -137,2 | -886,0 to 611,5 | No | ns | >0,9999 | G-W |
| **L.b vs. L.la** | -207,2 | -956,0 to 541,6 | No | ns | >0,9999 | G-X |
| **L.b vs. L.b** | -359,6 | -1108 to 389,1 | No | ns | 0,9854 | G-Y |
| **L.b vs. L.n** | -545,7 | -1294 to 203,1 | No | ns | 0,5225 | G-Z |
| **L.b vs. L.li** | 12,28 | -736,5 to 761,0 | No | ns | >0,9999 | G-AA |
| **L.n vs. L.li** | -124,1 | -872,8 to 624,7 | No | ns | >0,9999 | H-I |
| **L.n vs. Control** | 736,9 | -57,27 to 1531 | No | ns | 0,1082 | H-J |
| **L.n vs. LPS** | -1330 | -2124 to -536,0 | Yes | **** | <0,0001 | H-K |
| **L.n vs. S.a** | 315,8 | -478,4 to 1110 | No | ns | 0,9990 | H-L |
| **L.n vs. L.g** | -126,3 | -875,1 to 622,4 | No | ns | >0,9999 | H-M |
| **L.n vs. L.s** | -221,5 | -970,3 to 527,2 | No | ns | >0,9999 | H-N |
| **L.n vs. L.la** | -1535 | -2284 to -786,7 | Yes | **** | <0,0001 | H-O |
| **L.n vs. L.b** | -878,9 | -1673 to -84,76 | Yes | * | 0,0137 | H-P |
| **L.n vs. L.n** | -440,9 | -1190 to 307,8 | No | ns | 0,8774 | H-Q |
| **L.n vs. L.li** | -432,8 | -1182 to 315,9 | No | ns | 0,8956 | H-R |
| **L.n vs. Control** | 731,2 | -62,95 to 1525 | No | ns | 0,1162 | H-S |
| **L.n vs. LPS** | 207,3 | -586,9 to 1001 | No | ns | >0,9999 | H-T |
| **L.n vs. S.a** | -1092 | -1886 to -297,3 | Yes | *** | 0,0003 | H-U |
| **L.n vs. L.g** | -364,2 | -1113 to 384,6 | No | ns | 0,9829 | H-V |
| **L.n vs. L.s** | -230,6 | -979,3 to 518,2 | No | ns | >0,9999 | H-W |
| **L.n vs. L.la** | -300,6 | -1049 to 448,2 | No | ns | 0,9988 | H-X |
| **L.n vs. L.b** | -453,0 | -1202 to 295,8 | No | ns | 0,8471 | H-Y |
| **L.n vs. L.n** | -639,0 | -1388 to 109,7 | No | ns | 0,2164 | H-Z |
| **L.n vs. L.li** | -81,08 | -829,8 to 667,7 | No | ns | >0,9999 | H-AA |
| **L.li vs. Control** | 861,0 | 66,79 to 1655 | Yes | * | 0,0183 | I-J |
| **L.li vs. LPS** | -1206 | -2000 to -411,9 | Yes | **** | <0,0001 | I-K |
| **L.li vs. S.a** | 439,8 | -354,3 to 1234 | No | ns | 0,9297 | I-L |
| **L.li vs. L.g** | -2,264 | -751,0 to 746,5 | No | ns | >0,9999 | I-M |
| **L.li vs. L.s** | -97,48 | -846,2 to 651,3 | No | ns | >0,9999 | I-N |
| **L.li vs. L.la** | -1411 | -2160 to -662,6 | Yes | **** | <0,0001 | I-O |
| **L.li vs. L.b** | -754,9 | -1549 to 39,30 | No | ns | 0,0858 | I-P |
| **L.li vs. L.n** | -316,9 | -1066 to 431,9 | No | ns | 0,9974 | I-Q |
| **L.li vs. L.li** | -308,8 | -1058 to 440,0 | No | ns | 0,9982 | I-R |
| **L.li vs. Control** | 855,3 | 61,11 to 1649 | Yes | * | 0,0200 | I-S |
| **L.li vs. LPS** | 331,4 | -462,8 to 1126 | No | ns | 0,9979 | I-T |
| **L.li vs. S.a** | -967,5 | -1762 to -173,3 | Yes | ** | 0,0030 | I-U |
| **L.li vs. L.g** | -240,2 | -988,9 to 508,6 | No | ns | >0,9999 | I-V |
| **L.li vs. L.s** | -106,5 | -855,3 to 642,2 | No | ns | >0,9999 | I-W |
| **L.li vs. L.la** | -176,5 | -925,3 to 572,3 | No | ns | >0,9999 | I-X |
| **L.li vs. L.b** | -328,9 | -1078 to 419,8 | No | ns | 0,9955 | I-Y |
| **L.li vs. L.n** | -515,0 | -1264 to 233,8 | No | ns | 0,6407 | I-Z |
| **L.li vs. L.li** | 42,98 | -705,8 to 791,7 | No | ns | >0,9999 | I-AA |
| **Control vs. LPS** | -2067 | -2904 to -1230 | Yes | **** | <0,0001 | J-K |
| **Control vs. S.a** | -421,1 | -1258 to 416,0 | No | ns | 0,9746 | J-L |
| **Control vs. L.g** | -863,2 | -1657 to -69,05 | Yes | * | 0,0176 | J-M |
| **Control vs. L.s** | -958,5 | -1753 to -164,3 | Yes | ** | 0,0035 | J-N |
| **Control vs. L.la** | -2272 | -3067 to -1478 | Yes | **** | <0,0001 | J-O |
| **Control vs. L.b** | -1616 | -2453 to -778,7 | Yes | **** | <0,0001 | J-P |
| **Control vs. L.n** | -1178 | -1972 to -383,7 | Yes | **** | <0,0001 | J-Q |
| **Control vs. L.li** | -1170 | -1964 to -375,6 | Yes | **** | <0,0001 | J-R |
| **Control vs. Control** | -5,675 | -842,8 to 831,5 | No | ns | >0,9999 | J-S |
| **Control vs. LPS** | -529,6 | -1367 to 307,5 | No | ns | 0,7866 | J-T |
| **Control vs. S.a** | -1828 | -2666 to -991,3 | Yes | **** | <0,0001 | J-U |
| **Control vs. L.g** | -1101 | -1895 to -306,9 | Yes | *** | 0,0002 | J-V |
| **Control vs. L.s** | -967,5 | -1762 to -173,3 | Yes | ** | 0,0030 | J-W |
| **Control vs. L.la** | -1037 | -1832 to -243,3 | Yes | *** | 0,0008 | J-X |
| **Control vs. L.b** | -1190 | -1984 to -395,7 | Yes | **** | <0,0001 | J-Y |
| **Control vs. L.n** | -1376 | -2170 to -581,8 | Yes | **** | <0,0001 | J-Z |
| **Control vs. L.li** | -818,0 | -1612 to -23,81 | Yes | * | 0,0354 | J-AA |
| **LPS vs. S.a** | 1646 | 808,8 to 2483 | Yes | **** | <0,0001 | K-L |
| **LPS vs. L.g** | 1204 | 409,7 to 1998 | Yes | **** | <0,0001 | K-M |
| **LPS vs. L.s** | 1109 | 314,5 to 1903 | Yes | *** | 0,0002 | K-N |
| **LPS vs. L.la** | -205,3 | -999,4 to 588,9 | No | ns | >0,9999 | K-O |
| **LPS vs. L.b** | 451,3 | -385,9 to 1288 | No | ns | 0,9462 | K-P |
| **LPS vs. L.n** | 889,3 | 95,08 to 1683 | Yes | * | 0,0116 | K-Q |
| **LPS vs. L.li** | 897,4 | 103,2 to 1692 | Yes | * | 0,0101 | K-R |
| **LPS vs. Control** | 2061 | 1224 to 2899 | Yes | **** | <0,0001 | K-S |
| **LPS vs. LPS** | 1538 | 700,4 to 2375 | Yes | **** | <0,0001 | K-T |
| **LPS vs. S.a** | 238,7 | -598,5 to 1076 | No | ns | >0,9999 | K-U |
| **LPS vs. L.g** | 966,0 | 171,8 to 1760 | Yes | ** | 0,0031 | K-V |
| **LPS vs. L.s** | 1100 | 305,4 to 1894 | Yes | *** | 0,0003 | K-W |
| **LPS vs. L.la** | 1030 | 235,5 to 1824 | Yes | *** | 0,0010 | K-X |
| **LPS vs. L.b** | 877,2 | 83,00 to 1671 | Yes | * | 0,0141 | K-Y |
| **LPS vs. L.n** | 691,2 | -103,0 to 1485 | No | ns | 0,1869 | K-Z |
| **LPS vs. L.li** | 1249 | 454,9 to 2043 | Yes | **** | <0,0001 | K-AA |
| **S.a vs. L.g** | -442,1 | -1236 to 352,1 | No | ns | 0,9261 | L-M |
| **S.a vs. L.s** | -537,3 | -1332 to 256,9 | No | ns | 0,6722 | L-N |
| **S.a vs. L.la** | -1851 | -2645 to -1057 | Yes | **** | <0,0001 | L-O |
| **S.a vs. L.b** | -1195 | -2032 to -357,6 | Yes | *** | 0,0001 | L-P |
| **S.a vs. L.n** | -756,7 | -1551 to 37,47 | No | ns | 0,0837 | L-Q |
| **S.a vs. L.li** | -748,6 | -1543 to 45,57 | No | ns | 0,0931 | L-R |
| **S.a vs. Control** | 415,4 | -421,7 to 1253 | No | ns | 0,9783 | L-S |
| **S.a vs. LPS** | -108,5 | -945,6 to 728,7 | No | ns | >0,9999 | L-T |
| **S.a vs. S.a** | -1407 | -2244 to -570,2 | Yes | **** | <0,0001 | L-U |
| **S.a vs. L.g** | -680,0 | -1474 to 114,2 | No | ns | 0,2114 | L-V |
| **S.a vs. L.s** | -546,4 | -1341 to 247,8 | No | ns | 0,6402 | L-W |
| **S.a vs. L.la** | -616,3 | -1411 to 177,8 | No | ns | 0,3912 | L-X |
| **S.a vs. L.b** | -768,8 | -1563 to 25,39 | No | ns | 0,0712 | L-Y |
| **S.a vs. L.n** | -954,8 | -1749 to -160,6 | Yes | ** | 0,0038 | L-Z |
| **S.a vs. L.li** | -396,9 | -1191 to 397,3 | No | ns | 0,9765 | L-AA |
| **L.g vs. L.s** | -95,22 | -844,0 to 653,5 | No | ns | >0,9999 | M-N |
| **L.g vs. L.la** | -1409 | -2158 to -660,4 | Yes | **** | <0,0001 | M-O |
| **L.g vs. L.b** | -752,6 | -1547 to 41,57 | No | ns | 0,0884 | M-P |
| **L.g vs. L.n** | -314,6 | -1063 to 434,2 | No | ns | 0,9976 | M-Q |
| **L.g vs. L.li** | -306,5 | -1055 to 442,3 | No | ns | 0,9984 | M-R |
| **L.g vs. Control** | 857,6 | 63,38 to 1652 | Yes | * | 0,0193 | M-S |
| **L.g vs. LPS** | 333,6 | -460,5 to 1128 | No | ns | 0,9976 | M-T |
| **L.g vs. S.a** | -965,2 | -1759 to -171,0 | Yes | ** | 0,0031 | M-U |
| **L.g vs. L.g** | -237,9 | -986,7 to 510,9 | No | ns | >0,9999 | M-V |
| **L.g vs. L.s** | -104,3 | -853,0 to 644,5 | No | ns | >0,9999 | M-W |
| **L.g vs. L.la** | -174,2 | -923,0 to 574,5 | No | ns | >0,9999 | M-X |
| **L.g vs. L.b** | -326,7 | -1075 to 422,1 | No | ns | 0,9959 | M-Y |
| **L.g vs. L.n** | -512,7 | -1261 to 236,0 | No | ns | 0,6493 | M-Z |
| **L.g vs. L.li** | 45,24 | -703,5 to 794,0 | No | ns | >0,9999 | M-AA |
| **L.s vs. L.la** | -1314 | -2063 to -565,1 | Yes | **** | <0,0001 | N-O |
| **L.s vs. L.b** | -657,4 | -1452 to 136,8 | No | ns | 0,2676 | N-P |
| **L.s vs. L.n** | -219,4 | -968,1 to 529,4 | No | ns | >0,9999 | N-Q |
| **L.s vs. L.li** | -211,3 | -960,1 to 537,5 | No | ns | >0,9999 | N-R |
| **L.s vs. Control** | 952,8 | 158,6 to 1747 | Yes | ** | 0,0039 | N-S |
| **L.s vs. LPS** | 428,9 | -365,3 to 1223 | No | ns | 0,9453 | N-T |
| **L.s vs. S.a** | -870,0 | -1664 to -75,79 | Yes | * | 0,0158 | N-U |
| **L.s vs. L.g** | -142,7 | -891,4 to 606,1 | No | ns | >0,9999 | N-V |
| **L.s vs. L.s** | -9,036 | -757,8 to 739,7 | No | ns | >0,9999 | N-W |
| **L.s vs. L.la** | -79,01 | -827,8 to 669,8 | No | ns | >0,9999 | N-X |
| **L.s vs. L.b** | -231,5 | -980,2 to 517,3 | No | ns | >0,9999 | N-Y |
| **L.s vs. L.n** | -417,5 | -1166 to 331,3 | No | ns | 0,9250 | N-Z |
| **L.s vs. L.li** | 140,5 | -608,3 to 889,2 | No | ns | >0,9999 | N-AA |
| **L.la vs. L.b** | 656,5 | -137,7 to 1451 | No | ns | 0,2700 | O-P |
| **L.la vs. L.n** | 1095 | 345,8 to 1843 | Yes | **** | <0,0001 | O-Q |
| **L.la vs. L.li** | 1103 | 353,8 to 1851 | Yes | **** | <0,0001 | O-R |
| **L.la vs. Control** | 2267 | 1472 to 3061 | Yes | **** | <0,0001 | O-S |
| **L.la vs. LPS** | 1743 | 948,6 to 2537 | Yes | **** | <0,0001 | O-T |
| **L.la vs. S.a** | 443,9 | -350,3 to 1238 | No | ns | 0,9232 | O-U |
| **L.la vs. L.g** | 1171 | 422,5 to 1920 | Yes | **** | <0,0001 | O-V |
| **L.la vs. L.s** | 1305 | 556,1 to 2054 | Yes | **** | <0,0001 | O-W |
| **L.la vs. L.la** | 1235 | 486,1 to 1984 | Yes | **** | <0,0001 | O-X |
| **L.la vs. L.b** | 1082 | 333,7 to 1831 | Yes | **** | <0,0001 | O-Y |
| **L.la vs. L.n** | 896,4 | 147,6 to 1645 | Yes | ** | 0,0041 | O-Z |
| **L.la vs. L.li** | 1454 | 705,6 to 2203 | Yes | **** | <0,0001 | O-AA |
| **L.b vs. L.n** | 438,0 | -356,2 to 1232 | No | ns | 0,9325 | P-Q |
| **L.b vs. L.li** | 446,1 | -348,1 to 1240 | No | ns | 0,9195 | P-R |
| **L.b vs. Control** | 1610 | 773,0 to 2447 | Yes | **** | <0,0001 | P-S |
| **L.b vs. LPS** | 1086 | 249,1 to 1923 | Yes | *** | 0,0010 | P-T |
| **L.b vs. S.a** | -212,6 | -1050 to 624,6 | No | ns | >0,9999 | P-U |
| **L.b vs. L.g** | 514,7 | -279,5 to 1309 | No | ns | 0,7484 | P-V |
| **L.b vs. L.s** | 648,4 | -145,8 to 1443 | No | ns | 0,2925 | P-W |
| **L.b vs. L.la** | 578,4 | -215,8 to 1373 | No | ns | 0,5239 | P-X |
| **L.b vs. L.b** | 425,9 | -368,2 to 1220 | No | ns | 0,9490 | P-Y |
| **L.b vs. L.n** | 239,9 | -554,3 to 1034 | No | ns | >0,9999 | P-Z |
| **L.b vs. L.li** | 797,9 | 3,677 to 1592 | Yes | * | 0,0474 | P-AA |
| **L.n vs. L.li** | 8,096 | -740,7 to 756,9 | No | ns | >0,9999 | Q-R |
| **L.n vs. Control** | 1172 | 378,0 to 1966 | Yes | **** | <0,0001 | Q-S |
| **L.n vs. LPS** | 648,2 | -145,9 to 1442 | No | ns | 0,2928 | Q-T |
| **L.n vs. S.a** | -650,6 | -1445 to 143,6 | No | ns | 0,2862 | Q-U |
| **L.n vs. L.g** | 76,71 | -672,0 to 825,5 | No | ns | >0,9999 | Q-V |
| **L.n vs. L.s** | 210,3 | -538,4 to 959,1 | No | ns | >0,9999 | Q-W |
| **L.n vs. L.la** | 140,4 | -608,4 to 889,1 | No | ns | >0,9999 | Q-X |
| **L.n vs. L.b** | -12,08 | -760,8 to 736,7 | No | ns | >0,9999 | Q-Y |
| **L.n vs. L.n** | -198,1 | -946,9 to 550,6 | No | ns | >0,9999 | Q-Z |
| **L.n vs. L.li** | 359,8 | -388,9 to 1109 | No | ns | 0,9853 | Q-AA |
| **L.li vs. Control** | 1164 | 369,9 to 1958 | Yes | **** | <0,0001 | R-S |
| **L.li vs. LPS** | 640,1 | -154,0 to 1434 | No | ns | 0,3163 | R-T |
| **L.li vs. S.a** | -658,7 | -1453 to 135,5 | No | ns | 0,2641 | R-U |
| **L.li vs. L.g** | 68,62 | -680,1 to 817,4 | No | ns | >0,9999 | R-V |
| **L.li vs. L.s** | 202,3 | -546,5 to 951,0 | No | ns | >0,9999 | R-W |
| **L.li vs. L.la** | 132,3 | -616,5 to 881,0 | No | ns | >0,9999 | R-X |
| **L.li vs. L.b** | -20,18 | -768,9 to 728,6 | No | ns | >0,9999 | R-Y |
| **L.li vs. L.n** | -206,2 | -955,0 to 542,6 | No | ns | >0,9999 | R-Z |
| **L.li vs. L.li** | 351,8 | -397,0 to 1101 | No | ns | 0,9890 | R-AA |
| **Control vs. LPS** | -523,9 | -1361 to 313,2 | No | ns | 0,8024 | S-T |
| **Control vs. S.a** | -1823 | -2660 to -985,6 | Yes | **** | <0,0001 | S-U |
| **Control vs. L.g** | -1095 | -1890 to -301,3 | Yes | *** | 0,0003 | S-V |
| **Control vs. L.s** | -961,8 | -1756 to -167,6 | Yes | ** | 0,0033 | S-W |
| **Control vs. L.la** | -1032 | -1826 to -237,6 | Yes | *** | 0,0009 | S-X |
| **Control vs. L.b** | -1184 | -1978 to -390,1 | Yes | **** | <0,0001 | S-Y |
| **Control vs. L.n** | -1370 | -2164 to -576,1 | Yes | **** | <0,0001 | S-Z |
| **Control vs. L.li** | -812,3 | -1606 to -18,13 | Yes | * | 0,0384 | S-AA |
| **LPS vs. S.a** | -1299 | -2136 to -461,7 | Yes | **** | <0,0001 | T-U |
| **LPS vs. L.g** | -571,5 | -1366 to 222,7 | No | ns | 0,5489 | T-V |
| **LPS vs. L.s** | -437,9 | -1232 to 356,3 | No | ns | 0,9326 | T-W |
| **LPS vs. L.la** | -507,9 | -1302 to 286,3 | No | ns | 0,7700 | T-X |
| **LPS vs. L.b** | -660,3 | -1454 to 133,9 | No | ns | 0,2598 | T-Y |
| **LPS vs. L.n** | -846,4 | -1641 to -52,17 | Yes | * | 0,0230 | T-Z |
| **LPS vs. L.li** | -288,4 | -1083 to 505,8 | No | ns | 0,9998 | T-AA |
| **S.a vs. L.g** | 727,3 | -66,88 to 1521 | No | ns | 0,1220 | U-V |
| **S.a vs. L.s** | 860,9 | 66,75 to 1655 | Yes | * | 0,0183 | U-W |
| **S.a vs. L.la** | 791,0 | -3,220 to 1585 | No | ns | 0,0523 | U-X |
| **S.a vs. L.b** | 638,5 | -155,7 to 1433 | No | ns | 0,3212 | U-Y |
| **S.a vs. L.n** | 452,5 | -341,7 to 1247 | No | ns | 0,9083 | U-Z |
| **S.a vs. L.li** | 1010 | 216,3 to 1805 | Yes | ** | 0,0014 | U-AA |
| **L.g vs. L.s** | 133,6 | -615,1 to 882,4 | No | ns | >0,9999 | V-W |
| **L.g vs. L.la** | 63,66 | -685,1 to 812,4 | No | ns | >0,9999 | V-X |
| **L.g vs. L.b** | -88,79 | -837,6 to 660,0 | No | ns | >0,9999 | V-Y |
| **L.g vs. L.n** | -274,8 | -1024 to 473,9 | No | ns | 0,9997 | V-Z |
| **L.g vs. L.li** | 283,1 | -465,6 to 1032 | No | ns | 0,9995 | V-AA |
| **L.s vs. L.la** | -69,97 | -818,7 to 678,8 | No | ns | >0,9999 | W-X |
| **L.s vs. L.b** | -222,4 | -971,2 to 526,3 | No | ns | >0,9999 | W-Y |
| **L.s vs. L.n** | -408,5 | -1157 to 340,3 | No | ns | 0,9394 | W-Z |
| **L.s vs. L.li** | 149,5 | -599,3 to 898,3 | No | ns | >0,9999 | W-AA |
| **L.la vs. L.b** | -152,5 | -901,2 to 596,3 | No | ns | >0,9999 | X-Y |
| **L.la vs. L.n** | -338,5 | -1087 to 410,3 | No | ns | 0,9933 | X-Z |
| **L.la vs. L.li** | 219,5 | -529,3 to 968,2 | No | ns | >0,9999 | X-AA |
| **L.b vs. L.n** | -186,0 | -934,8 to 562,7 | No | ns | >0,9999 | Y-Z |
| **L.b vs. L.li** | 371,9 | -376,8 to 1121 | No | ns | 0,9781 | Y-AA |
| **L.n vs. L.li** | 558,0 | -190,8 to 1307 | No | ns | 0,4757 | Z-AA |
