# Supplementary material for: Unveiling the Enigmatic nature of six neglected Amazonian Leishmania (Viannia) species using the hamster model: Virulence, Histopathology and prospection of LRV1
Source: PLoS Negl Trop Dis. 2024 Aug 9;18(8):e0012333. doi: 10.1371/journal.pntd.0012333 (PMC11315283; doi:10.1371/journal.pntd.0012333)
Supplement: Attachment — Submitted filename: Soares review.docx [file pntd.0012333.s004.docx]

The paper “Unveiling the Enigmatic nature of Amazonian Leishmania (Viannia) 3 species: their Leishmania RNA viruses, Virulence, and Histopathology” by Soares et al. covers various aspects of least studied Leishmania species from the subgenus L. (Viannia) relative to in vivo and in vitro infections and immune responses. Although this is a quite descriptive study it reveals some important aspects that might be useful for future studies, as the use of the hamster model for better undestanding these poorly studied parasites. The specificities of L. lainsoni are interesting and studies should be pursued.

Major points:

- The title could be improved, if possible, add the name of the species studied, and diminish the visibility of the LRV1, since I do not think this is a crucial aspect of the paper.
- In relation to the LRV1 infections, I do not think you can attribute your results to the presence of the virus, since you do not have a control of the same Leishmania species without the virus. The differences could be due solely to the parasite specificities.

Minor points, mostly related to language and clarity:

L92: in the number cases recorded by this parasite: improve language

L94: depending on the region [3]. Depending on the region: repetitive.

L126: been occurred: please correct

L150 and 152: was kept: were kept

L174: Resident hamster: Hamster resident

L182: from the peritoneal cavity mice: from mice peritoneal cavity

L192: vector of transmitting L. (V.) braziliensis: vector of L. (V.) braziliensis

L299: Regarding, A few amastigotes: please correct

L345: L,: L.

L377: on the species: on the *Leishmania* species

L385: The low infectivity for L. (V.) naiffi in hamster cells may be due the fact that originally this species was isolated from armadillos [17]: well, none of the species were isolated from hamster, so I am not sure this is a good argument.

L435:, L. (V.) lainsoni 20 days PI: L. (V.) lainsoni infected animals 20 days PI

L535: corroborates with the: corroborates the
