# Supplementary material for: Unveiling the Enigmatic nature of six neglected Amazonian Leishmania (Viannia) species using the hamster model: Virulence, Histopathology and prospection of LRV1
Source: PLoS Negl Trop Dis. 2024 Aug 9;18(8):e0012333. doi: 10.1371/journal.pntd.0012333 (PMC11315283; doi:10.1371/journal.pntd.0012333)
Supplement: Attachment — Submitted filename: Rebutal letter.docx [file pntd.0012333.s005.docx]

**Responses to reviewers.**

**First, we would like to thank all the reviewers for their comments. We endeavored to incorporate their input fully and constructively. All the detailed modifications are highlighted in yellow.**

Reviewer #1: The methods are well defined with few issues. Please verify general comments.

Major points:

The title could be improved, if possible, add the name of the species studied, and diminish the visibility of the LRV1, since I do not think this is a crucial aspect of the paper.

**Response: We agree with the reviewer concerning LRV1 and we put it at the end of the title focusing on its prospection/detection. However, adding the six names of the species in the title increased it above 250 characters. For this reason, we included the term “six neglected Amazonian”. Also, reviewer #3 suggested including the animal model in the title. Now the title reads as follows (Lines 3-5):**

**Unveiling the Enigmatic nature of six neglected Amazonian Leishmania (Viannia) species using the hamster model: Virulence, Histopathology and prospection of LRV1**

In relation to the LRV1 infections, I do not think you can attribute your results to the presence of the virus, since you do not have a control of the same Leishmania species without the virus. The differences could be due solely to the parasite specificities.

**Response: We agree with the reviewer, and we changed the text accordingly in the abstract (lines 61-63), results (lines 228-229) and discussion (lines 374-378 and 383-386).**

**Abstract: LRV1 presence was found in *L. (V.) guyanensis* and *L. (V.) shawi* with no apparent correlation with virulence *in vitro* and *in vivo*.**

**Results: LRV1 presence did not seem to affect infection in hamster resident peritoneal macrophages and human THP-1 cells**

**Discussion: Although it is tempting to suggest that LRV1 presence may increase infectivity/intracellular parasitism, at least for *L. (V.) guyanensis*, the present study did not find a coherent correlation between LRV1 presence and invasion/intracellular replication in our in vitro models.**

**Discussion: Like *in vitro* experiments, in our experimental in vivo conditions, LRV1 could not solely explain variations in pathology among species/strains, since LRV1-positive *L. (V.) shawi* did not cause external lesion either.**

Minor points, mostly related to language and clarity:

L92: in the number cases recorded by this parasite: improve language. **OK.**

L94: depending on the region [3]. Depending on the region: repetitive. **Not applicable after shortening Introduction.**

L126: been occurred: please correct. **OK.**

L150 and 152: was kept: were kept. **OK.**

L174: Resident hamster: Hamster resident. **OK.**

L182: from the peritoneal cavity mice: from mice peritoneal cavity. **OK.**

L192: vector of transmitting L. (V.) braziliensis: vector of L. (V.) braziliensis. **OK.**

L299: Regarding, A few amastigotes: please correct. **OK.**

L345: L,: L. **OK.**

L377: on the species: on the Leishmania species. **OK.**

L385: The low infectivity for L. (V.) naiffi in hamster cells may be due the fact that originally this species was isolated from armadillos [17]: well, none of the species were isolated from hamster, so I am not sure this is a good argument.

**Response: We agree with the reviewer and removed this.**

L435:, L. (V.) lainsoni 20 days PI: L. (V.) lainsoni infected animals 20 days PI. **OK.**

L535: corroborates with the: corroborates the. **OK.**

Reviewer #1: Dear Authors,

The rationale of your manuscript is well-presented, but the manuscript requires several improvements, particularly concerning the lengthy format which needs to be updated to be more concise. I recommend rewriting the manuscript with a clearer, more focused aim. It would be beneficial to first present the analysis of parasites characteristics followed by the infection patterns. Below are some specific considerations:

Abstract: The abstract contains double the words recommended by PNTD. Please reduce it to the essential content.

**Response: According to the submission form the abstract limit is 500 words, currently it has 477 words.**

Introduction: While the Introduction is clear, it is overly extensive. Please include only the essential background to support the manuscript’s rationale.

**Response: We agree with the reviewer, and we endeavored to decrease the length of the Introduction. We removed redundant parts and the final sentence as requested. However, since those species are not well known and the taxonomic names of the sandflies and reservoirs were updated during the last three decades, we felt the need to rewrite them to ensure that their naming was correct.**

Methods

1, Consider making Table 2 supplementary information.

**Response: Since Table 2 (and also Table 3) refer to the parameters used for PCA analysis, and summarize all data from the article, we would prefer to keep them in the text because they complement each other.**

2, For the in vitro experiments where cytokines/chemokines such as TNF-α, IL-6, IL-12, and MCP-1 were assayed, please clarify if kits were used.

3, Specify how many in vitro experiments were performed and whether they were conducted in triplicate.

**Response: This information was included in M&M (lines 177-182) as follows:**

**Supernatants were assayed for nitric oxide and cytokines using Griess method and CBA flex Inflammation kit (Beckton and Dickinson) according to manufacturer’s instructions. The following cytokines/chemokine were assayed TNF-α, IL-6, IL-12, and MCP-1. Two parallel experiments were performed in triplicate totalizing 6 samples for each glycoconjugate exposure. These experiments were performed in the Flow Cytometry platform in the Rene Rachou Institute, Oswaldo Cruz Foundation.**

4, The classification of infection as Higher, Moderate, and Lower needs clarification. Please explain the method used and define each level.

**Response:** **To evaluate the inflammation in the skin, a semi‐quantitative comparative analysis was performed according to the modified method of Ridley and Ridley (1983). The results were scored based on the intensity of cellularity observed using a 40× objective as follows: (−) negative, 1‐50 cells (+) discrete, 50‐100 cells (++) moderate and greater than 100 cells (+++) intense. This information was included in the M & M (lines 201-204) and reference was included (now reference 44).**

Results and Discussion

5, Combine Figures 1 and 2 into a single figure, as Figure 1 by itself is not informative.

**Response: We apologize for this, but we tried to merge them, however it was too big and above the limit of PNTD. We had to leave as it is. Although it is just a qualitative result, it shows the ability of our strains to differentially infect hamsters´ macrophages.**

6, Adjust the order of Figure 2 to correctly present the classification of parasites according to LRV detection.

**Response: It is an interesting suggestion but since we observed a cline among the infection rates, aesthetically putting *L. braziliensis* in the first place resulted in making the statistical analysis confusing. For this reason, we had to leave as it is to make it easier for the readers. However, we changed the text according to the reviewer’s suggestion regarding the role of LRV1, that was a very important point.**

7, In Graph 3A, use different colors for each circle representing different parasites to show how each infects the hamsters.

**Response: These two animals represent only lesions caused by *L. guyanensis*, so that there is only one species. No need to change the colors of the circles.**

8, Specify whether the L. (V.) guyanensis used was isolated from humans or insects.

**Response: This information is provided in Table 1 (host´s column).**

9, Figures 3 through 7, which observe in vivo infection on different days, could be combined into one figure. Also, Figures 9 through 11 should be merged into one figure.

10, Figures 12 through 16 should be combined; also, clarify the letters used to symbolize statistical differences.

**Response: We respectfully disagree with the reviewer on this point. In our experience publishing immunopathology and innate immunity data in previous papers. Those are the most comprehensive sizes. Merging them, in our humble opinion, would decrease their sizes, making them very difficult for readers to even see the letters above the columns. Regarding the meaning of the letters, we corrected the legends as also requested by reviewer #3.**

11, Address whether IHC was tested for granuloma regions, especially since granulomas were detected without the presence of parasites.

**Response: We also performed IHC in the viscera, however, it was negative even in granuloma areas. The literature [refs 35,60,61] and our experience working with viscerotropic parasites like *L. infantum*, we observed that parasites or antigenic material (particulated or homogenous) can be seen in granulomatous reaction. However, for dermotropic parasites, parasites circulate and pass through the viscera, causing an inflammatory response but not establishing itself precisely due to its tropism for the cutaneous tissue and its sensitivity to internal body temperature, higher than that of the tegument. In this way, we focused on describing the cellular components of the granulomatous areas and shortened the visceralization part that was too extensive as well as other parts of the Discussion as requested.**

13, The aim presented in the Discussion seems to diverge from the rest of the manuscript. If the focus is on infection models, include more discussion on infection in THP-1 cells and consider the relevance of measured cytokines in these cells.

**Response: With respect to THP-1 cells, we included that data in the manuscript to advocate the use of hamster’s cells as a better cell model. Due to the low rates of infection in THP-1 cells and the high costs of the CBA flex kits, we were not encouraged to ascertain the cytokines levels in those cells nor to discuss it more deeply. For this reason, we sent that data as supplementary. We also shortened the discussion as requested and added a final paragraph focusing on the main objectives of the study as also requested by reviewer #3.**

12, The Discussion section is overly exhaustive. Ensure that it is focused and directly addresses the main research question. The paragraphs are excessively long.

14, The Discussion redundantly revisits all results and includes unnecessary literature reviews. Streamline this section and focus directly on discussing the findings.

15, The conclusion primarily discusses L. (V.) lainsoni, which does not align with the stated major aim of the study. Please revise accordingly.

**Response: We agree with the reviewer, and we removed the excessive background information and excessive references. This resulted in reducing the Discussion to 5.5 pages. Regarding *L. lainsoni*, we reduced this part drastically and added one more paragraph as main conclusionsrequested by reviewer #3.**

Minor comments throughout the manuscript:

Define ATL in the Introduction as well as in the abstract. **OK (line 87).**

Line 99: There is no need to capitalize "leishmaniasis."**OK.**

Lines 144-145: The mention of a wider project on the immunopathology of Leishmania parasites is unnecessary. **OK.**

Line 179: Adjust to "Cells were exposed to L. (Viannia) species. **OK.**

Line 181: Correct "Panoptic" usage. **OK.**

Asterisks in Figures 4, 5, and 6 should be explained in the figure legends. **OK.**

Line 316: The statement "During interaction with macrophages LPG and GIPLs had different levels of stimulation" is unclear.

**Response: We changed to: LPG and GIPLs differentially stimulated murine macrophages (line 308).**

Reviewer #3: The objectives of the study are unclear in the introduction, but they were specified in the abstract and in the author's summary and are articulated with a testable hypothesis. The study design is appropriate to address the stated objectives and the population is appropriate for the hypothesis tested, with the sample size being sufficient to ensure adequate power to address the hypothesis. Statistical analysis is adequate and ethical requirements were met.

**Response: We shortened the Introduction as requested by reviewer #1 and added more specific aims at the end (lines 132-135).**

Line 185–187 - Were the negative and positive controls placed after treating the macrophages with INF-gamma? If yes, this should be clear.

**Response: We clarified this part as requested as follows: (lines 174-176).**

**Negative control was RPMI medium only. Positive controls were primed with IFN-γ (3U/mL) and included LPS (100 ng/mL, TLR4 agonist) and *Staphylococcus aureus* extract (100 ng/mL, TLR2 agonist).**

Reviewer #3: The results are presented clearly and completely, with figures and tables of sufficient quality to understand the data. However, some points deserve attention:

Table 2 - Please, put the species and not just the strains in the first column.

**Response: Species names were included as requested.**

Figure 2, 12-16 - The relationship between the letters and the P value is unclear. Please describe this relationship in each subtitle.

**Response: to clarify this we included the following sentences:**

**The same letters above bars do not indicate statistical differences (P>0.05). Different letters indicate statistical differences (P<0.05).**

Figure 3 - To know when the lesion began to be significant, it would be interesting to compare the size of the paw edema on the days of infection with the size of the paw before infection. Was this comparison carried out? If yes, indicate in the figure and clarify in the subtitle.

**Response: In fact, the infection is only on the right foot pad, the left pad is not injected with parasites. The comparisons of the paw edema sizes are within the same animal.**

The hamster is known to be a good experimental model used for L. braziliensis. However, according to our experience in the laboratory, the lesion usually becomes significant around the sixth/seventh week after infection. It is possible that 5 weeks of infection (40 days PI) was not enough time for the lesion to be macroscopically visible to other species, since not even L. braziliensis the lesion became visible. This subject must be present in the discussion.

**Response: This is a very good point raised by the reviewer and we agree that this discussion falls into the intraspecies variability in *L. braziliensis*. Some strains can infect better and cause lesions in hamsters. It seems that strain Ba788 is not very pathogenic, though it caused a heavy infection in the dermis during our period. We contacted our colleague Dr. Camila Indiana (Fiocruz Bahia) that was performing ear infections with the same strain but without SGE. She observed a mild thickening of the ear (4-6 weeks PI) with no ulceration either. In conclusion, we think our interval was within a range that we could at least see a thickening of the footpad for *L. braziliensis*. But since this was in another part of the hamster, we will include that in the discussion. For this strain we should have followed for a longer period up to 8-10 weeks PI (lines 405-408):**

**However, no visible dermotropic lesions appeared in this species during the time proposed (up to 7 weeks PI). We therefore suggest that for this strain (BA788), a longer period of 8-10 weeks should be performed to circumvent the possible lack of apparent lesions.**

Figure 13A - Why does the scale continue up to 100,000 if the values end at 20,000? Consider finishing at 20,000.

**Response: We apologize for this, and we corrected as requested.**

The conclusions are supported by the data presented, and the limitations of the analysis have been described. The authors discuss how these data can be useful in advancing our understanding of the topic under study, and also address the relevance of the study to public health. However, some points could be better explored, such as, for example, the importance of establishing experimental models for the Leishmania species covered in the study.

**Response: This is another good point raised by the reviewer. After we finished our experiments on hamsters, we thought about which animal model we could try in future assays. Since most of the wild reservoirs are mammals and marsupials, to have an animal facility that could rear those animals is difficult. Currently, in Brazil, and in other countries, ethical committees on animal handling have become very harsh, even for mice protocols. Based on our data, and to the fact that 5 out 6 species caused infection in the dermis, we should try to improve the hamster model. For example, those strains were recovered from animals during our limiting dilution assays and are supposed to increase virulence. Those are reference strains that were kept in culture for many years and might have lost their full capacity. If I had to it all over again, I would use recovered strains and follow them up to 8-10 weeks. I think the best way would be to improve the hamster model by using more infective strains for a longer period.**

Establishing an experimental model that allows us to unravel the mechanisms of the complex host-parasite relationship is essential. This knowledge can serve as a basis for, for example, developing new, more effective, and less toxic, chemotherapy drugs for leishmaniasis treatment. This could be one of the biases to be explored in the introduction, since the working points to immune mechanisms present in the parasite-host relationship, which could serve, in subsequent studies, as possible targets for immunomodulators to treat these parasitoses.

**Yes, we agree. The establishment of an animal model would be important for drug screening during chemotherapeutic studies. Also, LPGs and GIPLs are potent inducers of immune responses. However, carbohydrates are poor inducers of memory and should be coupled with a protein to favor the presentation to immune cells. The use of an LPG/GIPLs for *L. lainsoni* for example could be a candidate for such a purpose.**

**We included these points in the last part of discussion as requested (lines 481-409).**

Reviewer #3: The article is interesting, and the topic is quite relevant. However, important changes need to be made. The introduction does not make the objective of the article clear. However, both the abstract and the author summary suggest that the objective would be to establish an experimental model for Amazonian *Leishmania (Viannia)* species. It was unclear to me whether this was specifically the objective of the work, principally when we observe its title. I suggest that the authors rethink whether the article title could contain the possibility of establishing an experimental model for these species.

**Response: We changed the title as follows: Unveiling the Enigmatic nature of six neglected Amazonian *Leishmania (Viannia)* species using the hamster model: Virulence, Histopathology and prospection of LRV1. Also, as suggested by reviewer #1, we included changes in the abstract and introduction to clarify this.**

The introduction is disjointed and confusing. It needs to be reviewed. The objective of the work must be clear. The introduction needs to provide information so that the reader understands the importance of this objective.

**Response: The Introduction was shortened as requested by reviewer #1 to become more objective.**

Required modifications

Introduction:

Line 94 - One sentence ends with the expression "depending on the region", and the subsequent sentence starts with the same expression. It is better to change the expression in the subsequent sentence.

**Response. Not applicable anymore since Introduction was shortened.**

Lines 96-98 - Consider rewriting the sentence: “Recently, urbanization of ATL was reported in the Southeast Brazil, where L. (V.) braziliensis proven vectors Migonemyia migonei, Ny. intermedia and Ny. whitmani were detected in urban areas [9,10]. **OK.**

Lines 101-102 - Consider rewriting the sentence: “Most LCL cases are in the north and northeast of Brazil, where L. (Viannia) parasites are endemic, the disease being a zoonosis and occupational hazard [12]. **OK. (now lines 97-98).**

Lines 107-108 - The sentence “A distinguished feature of this species is that it is often infected with an endosymbiont virus from the Totiviridae family named Leishmania RNA virus 1 (LRV1), known to increase disease severity.” is confusing. Consider exchanging for “The frequent infection of this species by Leishmania RNA virus 1 (LRV1), a Totiviridae virus known to increase disease severity, is a distinguished characteristic of L. guyanensis.” **OK.**

Line 118 - Consider changing “At that time” to “Again”. **Not applicable anymore.**

Lines 123-126 - Consider rewriting the sentence: “A distinguished feature of L. (V.) lainsoni compared to the other L. (Viannia) species is that it possesses several biological, biochemical, and molecular characteristics that hinder its positioning within the same subgenus [25]. **OK (now lines 113-116).**
